# Chemoenzymatic Synthesis of ABC-Type Enantiostructured Triacylglycerols by the Use of the *p*-Methoxybenzyl Protective Group

**DOI:** 10.3390/molecules29071633

**Published:** 2024-04-05

**Authors:** Hafdis Haraldsdottir, Haraldur G. Gudmundsson, Kaisa M. Linderborg, Baoru Yang, Gudmundur G. Haraldsson

**Affiliations:** 1 Chemistry Department, Science Institute, University of Iceland, Dunhaga 3, 107 Reykjavik, Iceland; hah118@hi.is (H.H.); hgg@hi.is (H.G.G.); 2 Food Sciences, Department of Life Technologies, University of Turku, 20520 Turku, Finland; kaisa.linderborg@utu.fi (K.M.L.); bayang@utu.fi (B.Y.)

**Keywords:** asymmetric synthesis, enantiostructured triacylglycerols, lipase, *p*-methoxybenzyl (PMB) protective group, enantiospecific TAG analysis

## Abstract

This report demonstrates the first asymmetric synthesis of enantiopure structured triacylglycerols (TAGs) of the ABC type presenting three non-identical fatty acids, two of which are unsaturated. The unsaturated fatty acids included monounsaturated oleic acid (C18:1 n-9) and polyunsaturated linoleic acid (C18:2 n-6). This was accomplished by a six-step chemoenzymatic approach starting from (*R*)- and (*S*)-solketals. The highly regioselective immobilized *Candida antarctica* lipase (CAL-B) played a crucial role in the regiocontrol of the synthesis. The synthesis also benefited from the use of the *p*-methoxybenzyl (PMB) ether protective group, which enabled the incorporation of two different unsaturated fatty acids into the glycerol skeleton. The total of six such TAGs were prepared, four constituting the unsaturated fatty acids in the *sn*-1 and *sn*-2 positions, with a saturated fatty acid in the remaining *sn*-3 position of the glycerol backbone. In the two remaining TAGs, the different unsaturated fatty acids accommodated the *sn*-1 and *sn*-3 end positions, with the saturated fatty acid present in the *sn*-2 position. Enantiopure TAGs are urgently demanded as standards for the enantiospecific analysis of intact TAGs in fats and oils.

## 1. Introduction

This report focuses on the synthesis of enantiopure structured triacylglycerols (TAGs), which we have named enantiostructured TAGs. Glycerol is prochiral, and the prerequisite for a TAG to become chiral is that the fatty acids accommodating the terminal 1,3-positions are different, regardless of the fatty acid occupying the central 2-position. Accordingly, chiral TAGs may be classified as AAB- and ABC-type TAGs, constituting two or three different fatty acids, respectively. A prefix, *sn*-, pertains to a stereospecific numbering that is used to distinguish between the two enantiotopic terminal carbons of the glycerol backbone in TAGs [1,2]. The *pro-S* hydroxycarbon group refers to the *sn*-1 position, the *pro-R* group to the *sn*-3 position, and the central carbon to the *sn*-2 position.

Our interest relates to synthetic challenges as well as opening an access to a library of pure enantiostructured TAGs intended as standards for the enantiospecific analysis of chiral TAGs present in natural fats and oils. A major obstacle in such analyses performed by chiral HPLC is the lack of enantiopure TAGs as reference compounds to confirm their elution behavior and retention order [3,4,5,6,7,8]. Ultimately, this may enable a more comprehensive analysis of complex natural TAG mixtures, which frequently contain remarkably high proportions of chiral ABC-type TAGs [9,10,11]. The resulting TAGs may also find use in serving as model compounds to study enzyme activities and biological functions.

Recently, we have succeeded in the synthesis of enantiostructured TAGs of various types. This includes AAB-type TAGs, possessing only two different types of fatty acids. AAB-type TAGs are covered by four AAB subclasses. Our synthetic focus has been on the SSU category, constituting two identical saturated fatty acids (S) and one unsaturated fatty acid (U), and the SUU category, with two identical unsaturated fatty acids and one saturated fatty acid [7,12]. The remaining subclasses include the SSS’ category, possessing both fatty acids as saturated, and the UUU’ category, with both fatty acids as unsaturated. Likewise, we have also reported the synthesis of ABC-type enantiostructured TAGs possessing three different fatty acids with two different saturated fatty acids and one unsaturated fatty acid [13]. These TAGs belong to two (out of six) ABC-type TAG subclasses, namely, the SS’U and SUS’ categories.

**Figure 1:**
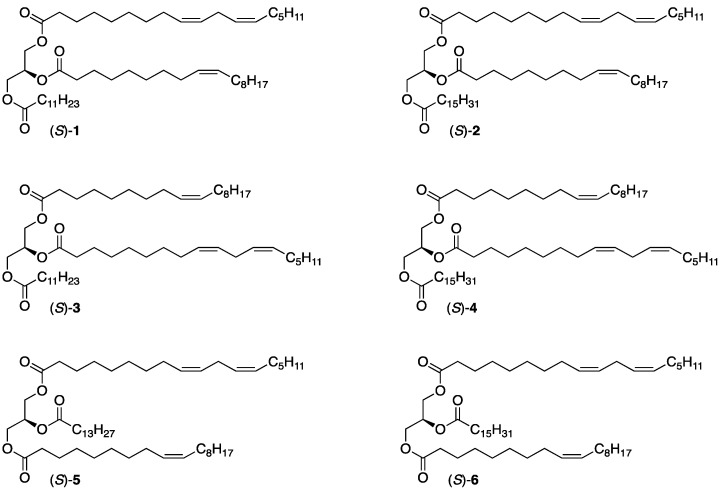
The structures of the six enantiostructured ABC-type TAG products synthesized: (*S*)-**1**, (*S*)-**2**, (*S*)-**3**, and (*S*)-**4** belong to the UU’S subclass category and (*S*)-**5** and (*S*)-**6** to the USU’ subclass category.

In the work described herein, our attention is focused on the synthesis of ABC-type TAGs constituting two different unsaturated fatty acids and one saturated fatty acid. Accordingly, these TAGs belong to the UU’S and USU’ subclass categories. The unsaturated fatty acids involved in this work are monounsaturated oleic acid (C18:1 n-9) and polyunsaturated linoleic acid (C18:2 n-6). Saturated fatty acids include lauric acid (C12:0), myristic acid (C14:0), and palmitic acid (16:0). The synthetic target included a total of six TAG molecules, all having the (*S*)-configuration, four of which belong to the UU’S category, (*S*)-**1**–**4**, and two to the SUS’ category, (*S*)-**5** and **6**. Their structures are revealed in Figure 1.

To execute the synthesis of ABC-type enantiostructured TAGs possessing two different unsaturated fatty acids, our previous strategy, which was based on the use of a benzyl ether as a protective group for the glycerol skeleton, needed a revision. With benzyl ether, we can easily deal with TAGs possessing one unsaturated fatty acid and two different saturated fatty acids. This is no longer an option when dealing with two different unsaturated fatty acids. Our modification is based on replacing the benzyl group with a *p*-methoxybenzyl protective group. The results are described in the current report.

The work described herein clearly reflects a great deal of novelty not only in terms of the TAG products accomplished but also their extensive utilities. The primary role of enantiostructured TAGs as standards for the enantiospecific analysis of chiral TAGs commonly present in natural fats and oils has already been demonstrated [4,7], and similar analyses of the TAGs obtained from the current work are underway to be reported within a few months. Furthermore, the enantiostructured TAGs have also found use in various important biological activities that include bioavailability [14,15,16,17] and oxidative stability [17,18,19] studies of bioactive n-3 polyunsaturated fatty acids, with a special emphasis on their location in terms of the stereospecific positions within the TAGs.

## 2. Results and Discussion

All these syntheses were dependent on enantiopure (*R*)- and (*S*)-solketals as chiral precursors and a highly regioselective *Candida antarctica* lipase (CAL-B) to incorporate fatty acids exclusively into the primary alcohol 1,3-positions of the glycerol backbone to control the regiochemistry. The syntheses were also dependent on the use of a benzyl ether protective group to maintain the chirality of the glycerol skeleton after the removal of the original isopropylidene protective moiety. The deprotection of the benzyl ether requires catalytic hydrogenolysis, under which conditions unsaturated fatty acids that are present obviously will not survive. This means that all the manipulations involving unsaturated fatty acids must be brought about after such deprotection and that the introduction of unsaturated fatty acids must take place in the final step(s) of the synthesis.

This is not a problem when dealing with the synthesis of the above AAB- and ABC-type enantiostructured TAGs substituted with only one type of unsaturated fatty acid. But once dealing with the introduction of two unsaturated fatty acids of different types, which is the case with the TAGs belonging to the SUU’ and USU’ subclass categories, the task becomes more challenging. That task requires an alternative protective group to the benzyl group, for which removal is tolerated by the unsaturated fatty acids. In that context, we came up with the idea for using a *p*-methoxybenzyl (PMB) ether to protect the end position of the glyceryl backbone, which may be cleaved under mild oxidative conditions by the use of 2,3-dichloro-5,6-dicyanobenzoquinone (DDQ) [20,21]. The question remains here as to whether not only mono- but also di- or even higher polyunsaturated fatty acids (PUFAs) might possibly survive such an oxidative treatment. It should also be emphasized that challenges related to acyl migration promoted by parameters including temperature, the presence of an acid or base, and the use of silica gel in relation to chromatography treatments must also be kept under control to maintain the regiocontrol of the synthesis [22,23,24].

### 2.1. Chemoenzymatic Synthesis of the SUU’ Subclass Category TAGs, (S)-***1*** and ***2***

**Figure 2:**
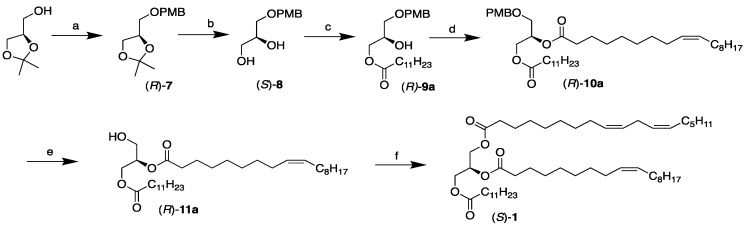
Chemoenzymatic synthesis of the SUU’ subclass category TAGs, (*S*)-**1**, **2**, **3**, and **4** (shown for (*S*)-**1**). Reagents and conditions: (**a**) NaH, THF, then PMB-Cl, reflux (76%); (**b**) I_2_, H_2_O, acetonitrile r.t. (97%); (**c**) vinyl dodecanoate, CAL, CH_2_Cl_2_, r.t. (>99%); (**d**) oleic acid, EDCI, DMAP, CH_2_Cl_2_, r.t. (99%); (**e**) DDQ, CH_2_Cl_2_, H_2_O, from 0 °C to r.t. (91%); (**f**) linoleic acid, EDCI, DMAP, CH_2_Cl_2_, r.t. (85%).

A six-step chemoenzymatic approach was designed for the synthesis of the SUU’ subclass category TAGs, (*S*)-**1**–**4**, which is depicted in Figure 2 (as shown for (*S*)-**1**). It is based on the use of (*R*)-solketal as a chiral precursor of which the *sn*-1 position is protected as a PMB ether in the first step. This is followed by the removal of the isopropylidene protective moiety and the subsequent lipase-promoted regioselective acylation of the *sn*-3 hydroxyl group in the resulting diol with a saturated fatty acid. The fourth step involves the introduction of the first unsaturated fatty acid to the remaining *sn*-2 position. This is followed by the removal of the PMB protective group, and in the final step, the second unsaturated fatty acid is incorporated into the *sn*-3 position of the glycerol backbone to complete the synthesis.

In the first step, the PMB protective group was attached to the free *sn*-1 hydroxyl group of (*R*)-solketal by treating it with *p*-methoxybenzyl chloride (PMB-Cl) in THF under reflux for 22 h, using sodium hydride as a base. The PMB-protected solketal, (*R*)-**7**, was obtained in a 76% yield. The reaction required a significantly longer reaction time than the corresponding reaction with benzyl chloride (4 h) under identical conditions [7,12,13], which relates to the electron-donating properties of the *p*-methoxyl group. That electron-donating effect also created challenges in the subsequent removal of the isopropylidene protective moiety from the (*R*)-**7** product.

In our previous TAG synthesis cases involving the benzyl group protection of the solketal, the isopropylidene group deprotection was smoothly brought about by hydrolysis using aqueous 1 M HCl in ethanol [7,12,13]. However, when the PMB-protected solketal (*R*)-**7** was gently refluxed with aqueous HCl in ethanol, no PMB-protected glycerol, (*S*)-**8**, was obtained. The reaction was attempted several times under milder conditions at room temperature, with differing reaction times and acid concentrations. That resulted in the desired diol in poor yields (20–35%). It appears that the electron-donating properties of the *p*-methoxyl group were strong enough to induce an S_N_1-type cleavage of the PMB group, causing the reaction to yield mostly a free glycerol. Hence, it was clear that a different approach was needed.

Molecular iodine in the presence of water in acetonitrile as a solvent has been used to cleave isopropylidene acetals in the presence of several acid-sensitive protecting groups, including PMB [25,26]. This iodine–water-based method was adopted to remove the isopropylidene group from the solketal in the above synthesis to give excellent results, with the PMB-protected diol, (*S*)-**8**, being obtained in 97% yields.

Having the chemoselective acetal protective group’s removal successfully sorted out and the resulting PMB-ether-protected glycerol, (*S*)-**8**, in hand, the next reaction involved the introduction of fatty acids to the free end position. The selected fatty acids included the three saturated lauric (C12:0), myristic (C14:0), and palmitic (C16:0) acids. All three saturated fatty acids were commercially available as activated vinyl esters. Their activation ensures a fast reaction with the enol leaving group undergoing tautomerization to form acetaldehyde, securing an irreversible process. A fast irreversible process favors the excellent regioselectivity of the lipase [27,28].

The highly regioselective *Candida antarctica* lipase B (CAL-B) was used to exclusively acylate the primary *sn*-3 rather than the secondary *sn*-2 position of the glycerol backbone. Mildness in terms of the temperature is another essential parameter that is offered by the lipase to avoid the acyl migration [22,23,24] of the fatty acid once it has been introduced to a predetermined position of the glycerol backbone. We have demonstrated that under the above mild conditions offered by the lipase, no such acyl migration took place to disturb the regiocontrol of the acylation, and that was also the case in the current reactions [27,28].

In addition to that, in all the previous and current acylation steps involving the lipase and when dealing with all the intermediates possessing an acyl group adjacent to a free hydroxyl group in the glycerol skeleton, extra care had to be taken to avoid acyl migration. That includes when performing the reactions, during their work-ups and the purification of the products by the use of silica-gel-based chromatography. The use of silica gel is known to promote acyl migration, and to get around that, a silica gel impregnated with 4% boric acid was used [29,30].

The lipase-promoted reactions of the vinyl esters were performed in dry dichloromethane at room temperature and were completed in 4 h to afford the (*R*)-**9a–c** PMB-protected monoacylglycerols (MAGs) in excellent quantitative yields. The (*R*)-**9a** product with lauric acid was obtained as a colorless oil, while those of myristic and palmitic acids, (*R*)-**9b** and (*R*)-**9c**, respectively, precipitated as white lightweight powders. Table 1 shows the identities, yields, and specific optical activities of these PMB-protected *sn*-3-MAG derivatives. The corresponding (*R*)-**14** from the USU’ subclass category TAG synthesis (see Section 2.2 below) has been included in the table.

The structures of the PMB-protected MAGs were confirmed by the characteristic pattern for the glycerol region (δ 5.40–3.60 ppm) in their ^1^H-NMR spectra. Figure S1 in the Supplementary Materials shows a comparison of the glycerol region of the PMB-protected solketal, (*R*)-**7**, the PMB-protected glycerol, (*S*)-**8**, and the PMB-protected *sn*-3-MAG, (*R*)-**9c**. No sign of acyl migration was observed in the case of the PMB-protected *sn*-3-MAG derivatives. Acyl migration side reactions would distort the peak pattern and give additional peaks in their glycerol proton region.

The *sn*-2 mid-position of the PMB-protected *sn*-3-MAGs, (*R*)-**9a**–**c**, was acylated by the use of 1-ethyl-3-(3-dimethylaminopropyl)carbodiimide (EDCI) as a coupling agent in the presence of 4-dimethylaminopyridine (DMAP) as a base and a catalyst. The reactions were performed under conditions identical to those previously described in our syntheses of structured and enantiostructured TAGs in dichloromethane at room temperature, under which no acyl migration took place [12,13,27,28]. The participating fatty acids included the unsaturated oleic acid (18:1 n-9) and linoleic acid (18:2 n-6) in the case of the SUU’ subclass category TAGs.

All the PMB-protected diacylglycerol (DAG) products, (*R*)-**10a**–**e**, were obtained as colorless oils in very high to excellent yields (88–99%), in the same range as those previously reported for the benzyl-protected acylglycerols [13]. All these PMB-protected *sn*-2,3-DAG derivatives showed specific rotation values between −6.0 and −7.0. Table 2 shows a summary of the identities, the yields obtained, and the specific optical activities of these intermediates. The corresponding (*R*)-**15a**,**b** from the USU’ subclass category TAG synthesis (see Section 2.2 below) have also been included in the table.

Again, the structures of the PMB-protected DAGs were confirmed by the characteristic pattern for the protons belonging to the glycerol region of their ^1^H-NMR spectra. Figure S2 in the Supplementary Materials shows a comparison of the glycerol regions of the PMB-protected *sn*-3-MAG, (*R*)-**9b**, and the PMB-protected *sn*-2,3-DAG, (*R*)-**10c**. It is of interest to notice the dramatic changes taking place in the spectrum upon the acylation of the *sn*-2 position, confirming a successful reaction.

#### 2.1.1. DDQ Deprotection with an Incorporated MUFA Present

The most challenging key step in the proposed synthetic approach was the removal of the PMB-protecting group by the use of DDQ in a water–dichloromethane medium. DDQ is a mild oxidant capable for removing the *p*-methoxybenzyl protective group from alcohols and other derivatives under neutral conditions [20,21,31]. The first important question as to whether a monounsaturated fatty acid would survive the oxidative treatment would already be an important achievement in the synthesis of the SUU’ and USU’ subclass categories of the enantiostructured ABC-type TAGs. A more critical question is related to whether a more unsaturated fatty acid, such as a diene fatty acid, would survive such an oxidation, or perhaps the longer-chain PUFAs.

It is believed that the PMB group and the oxidizing benzoquinone form a charge-transfer complex before reacting in an oxidation–reduction-type reaction involving the transfer of single electrons [21]. The reduced hydroquinone (DDQ-H_2_) is insoluble in both water and organic solvents and is, therefore, easily removed in the work-up. Finally, the now-oxidized PMB group is vulnerable to a nucleophilic attack by water at the benzyl position and ends up cleaving off as *p*-methoxybenzaldehyde.

First, the compounds listed in Table 2 and containing the monounsaturated oleic acid along with a saturated fatty acid, were introduced to the deprotection, that is, not only (*R*)-**10a**–**c** but also (*R*)-**15a**,**b** (see Section 2.2 below). Those PMB-protected DAGs possessing linoleic acid, (*R*)-**10d**,**e**, were looked at separately. This was intended to test the compatibility of a single double bond in oleic acid to the reaction conditions. The reaction was highly successful, with all the compounds being deprotected in very high to excellent yields with no indication of any deterioration of the double bond present in the monounsaturated fatty acid. The identity, obtained yield, along with the specific optical rotation of each of the *sn*-2,3-DAG products, (*R*)-**11a**–**c** and (*R*)-**16a**,**b**, (Section 2.2) are revealed in Table 3.

The formation of a charge-transfer complex turned the solution a dark color, usually dark green or brown, which slowly faded until the reaction solution had become colorless, with a bright red aqueous phase. At that time, the reaction proceeded to completion, as indicated by TLC monitoring. For some reason, care had to be taken during the work-up of the reaction. The reaction product mixture appeared to be quite vulnerable, even after extraction in dichloromethane. It could not be kept in solution for more than a couple of hours or be exposed to temperatures above 20 °C without a clear deterioration in the product, as indicated by ^1^H NMR spectroscopy. Treatment on a rotary evaporator to remove the solvent had to be performed at room temperature without heating. Once the product had been isolated and purified, it remained stable, and there were no signs of any acyl migration taking place, as is evident from Figure S3 in the Supplementary Materials displaying the glyceryl proton region of the ^1^H NMR spectrum of (*R*)-**16a** as a typical example for 1,2-DAGs [12,13].

#### 2.1.2. DDQ Deprotection with an Incorporated PUFA Present

Having confirmed that a single double bond present in oleic acid was unaffected by the oxidative cleavage conditions for the PMB protective group, we wanted to investigate further whether fatty acids of higher unsaturation would survive. Two of the PMB-protected diacylglycerols in Table 2 constitute linoleic acid, possessing two methylene-interrupted double bonds, namely, (*R*)-**10d** and (*R*)-**10e**. Both adducts contain linoleic acid (C18:2 n-6) in their *sn*-2 position, with saturated lauric (C12:0) and palmitic (C16:0) acids in the sn-3 position. They serve as intermediates in the intended syntheses of (*S*)-**3** and (*S*)-**4**, respectively, and belong to the SUU’ subclass category TAGs. They were prepared by the chemoenzymatic approach shown in Figure 2. We were particularly interested in finding out whether dienes of that type might possibly survive the deprotection conditions involving the DDQ oxidation. They are far more prone to undergo oxidation as compared to an isolated double bond present in monounsaturated fatty acids [32,33,34,35]. For that investigation, the (*R*)-**10e**-constituting palmitic acid was chosen to experiment on.

First, the DDQ reaction was performed under the exact conditions as those when deprotecting the monounsaturated (*R*)-**10a**–**c**, as described above, using 1.3 equivalents of the DDQ oxidant stirred with a solution of the PMB-protected diacylglycerols in water–dichloromethane for 6 h at room temperature. The results were disappointing because the desired deprotected (*R*)-**11e** was isolated at only a 26% yield along with some unreacted starting material and a highly polar fraction. The polar fraction comprised oxidized breakdown products of linoleic acid, as indicated by ^1^H NMR spectroscopy.

Most likely, the oxidizer DDQ was destroying the double bond system. Skipped polyenes are much more sensitive to oxidation than the single double bonds of MUFAs [32,33,34,35]. To confirm this breakdown, free linoleic acid was stirred with DDQ, and the solution was monitored with TLC. As time passed, the single spot of the linoleic acid started to break down into more polar spots, indicating that, indeed, the diene system was being destroyed. A ^1^H NMR spectrum of linoleic acid before and after exposure to DDQ supported the postulate, with the olefin peaks being diminished after the experiment.

Several attempts were made to try to adjust the reaction conditions for the more vulnerable diene system. The amount of DDQ was reduced to one equivalent in hopes that less free oxidizer in the solution would yield more product. Additionally, DDQ was dissolved in dichloromethane and added slowly to the solution via a dropping funnel, instead of all at once, to further minimize the free DDQ, and the reaction time was decreased. These adjustments had some limited success, resulting in increased yields from 26% to 36%, and more unreacted starting material (43%) was obtained than oxidized side products. However, it was clear that the reaction was far from ideal. The product, (*R*)-**11e**, obtained from these experiments was acylated with lauric acid to accomplish the TAG product, (*R*)-**12f**, which has been included in Table 4 (see Section 2.2).

#### 2.1.3. Chemical Coupling of the Final Fatty Acid

To complete the synthesis of the intended enantiostructured TAGs, (*S*)-**1** and (*S*)-**2**, the final step involved the chemical coupling of the third and final fatty acid into the remaining *sn*-1 position of the *sn*-2,3-DAG precursors. In accordance with the scheme in Figure 2, this was brought about by EDCI and DMAP, as described in previous steps using free fatty acids. The final TAGs were obtained as colorless oils in very good to excellent yields. Table 4 outlines the identities, yields, and optical activities of the final products. The corresponding (*S*)-**5** and (*S*)-**6** from the USU’ subclass category TAG synthesis (see Section 2.2 below) have also been included in the table.

As can be further noticed from Table 4, six enantiostructured ABC-type TAGs belonging to the SUS’ subclass category have also been included there. Earlier, we had reported the synthesis of TAGs of that subclass by the use of the benzyl protective group, which involved two separate lipase steps for vinyl esters of different saturated fatty acids [13]. Alternatively, the additional TAGs included were prepared using the PMB protective group approach described herein and, thus, involved only one lipase step for the saturated fatty acid vinyl esters. In addition to that, the TAGs included are the enantiomers of those previously prepared, i.e., (*R*)-**12a**, **b**, **d**, and **f**, whereas the (*R*)-**12c** and (*S*)-**12e** TAGs had not been synthesized before. The TAG (*R*)-**12f** was prepared by the acylation of diacylglycerol (*R*)-**11e**, as obtained in the previous section.

Figure S4 in the Supplementary Materials depicts the glyceryl proton region in the ^1^H NMR spectrum of (*S*)-**5**, which is characteristic of TAGs [27,28]. The optical activities of all the TAGs were extremely low, such that it was rather difficult to measure the exact values. Frequently, when a sample was measured, the rotation angle of the polarized light would bounce up and down around the zero value, and a high concentration of the sample (40–60 mg per 1 mL) was needed to obtain a good, stable measurement. This observation can be explained by a phenomenon known as cryptoactivity or cryptochirality [36,37,38,39]. Compounds that show cryptoactivity are chiral, but the stereogenic center possesses two moieties that are so similar that optical rotation becomes non-measurable. The carbon chains of the fatty acids at the *sn*-1 and *sn*-3 positions in TAGs, which give rise to the molecule’s chirality, are essentially too alike, such that the TAGs are functionally optically inactive. Although the TAGs showed very low optical rotation, it is without a doubt that they are indeed chiral because all the intermediates in the synthesis retained their enantiopurities throughout, and the final reaction would not cause racemization.

### 2.2. Chemoenzymatic Synthesis of the USU’ Subclass Category TAGs, (S)-***5*** and ***6***

**Figure 3:**
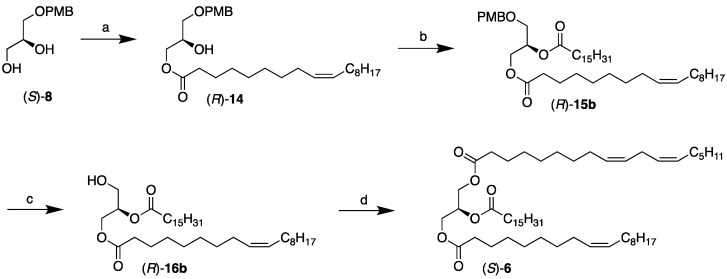
Chemoenzymatic synthesis of the USU’ subclass category TAGs, (*S*)-**5** and (*S*)-**6** (shown for (*S*)-**6**). Reagents and conditions: (**a**) oleic acid acetoxime ester (**13**), CAL-B, vacuum, r.t. (87%); (**b**) hexadecanoic acid, EDCI, DMAP, CH_2_Cl_2_, r.t. (91%); (**c**) DDQ, CH_2_Cl_2_, H_2_O, from 0 °C to r.t. (91%); (**d**) linoleic acid, EDCI, DMAP, CH_2_Cl_2_, r.t. (95%).

Like the SUU’ subclass category TAG synthesis, a six-step chemoenzymatic approach was designed for the two intended USU’ subclass category TAGs, (*S*)-**5** and (*S*)-**6**, with both syntheses sharing the (*S*)-**8** PMB-protected glycerol intermediate. The proposed approach is depicted in the scheme in Figure 3. It involves the lipase-promoted regioselective acylation of oleic acid in the *sn*-3 position of the protected glycerol. This is followed by the introduction of saturated fatty acids to the *sn*-2 position by chemical coupling, the deprotection of the PMB group, and, finally, the introduction of linoleic acid to the *sn*-1 position by a second chemical coupling to complete the synthesis.

Because vinyl esters of unsaturated fatty acids, including oleic acid, are not readily available, we were dependent on a different activated ester form that when dealing with such fatty acids in regioselective lipase biotransformations. Previously, we have successfully activated polyunsaturated fatty acids as acetoxime (acetone oxime) esters, including EPA (20:5 n-3) and DHA (22:6 n-3) [40], to obtain excellent regioselectivity in their lipase reactions involving CAL-B with glycerol to prepare symmetrically structured TAGs [40,41]. However, the oximes are clearly less reactive than the vinyl esters, and the irreversibility is not as explicit as when using the enol esters so that we have depended on the use of a vacuum to retard the reversibility of the system [40].

Oleic acid, in its reaction with acetone oxime, was converted to its acetoxime ester (**13**) in a quantitative yield by the use of the EDCI coupling agent in the presence of DMAP in dichloromethane at room temperature. At first, the reaction of the acetoxime ester (**13**) with the PMB-protected glycerol, (*S*)-**8**, promoted by CAL-B, only gave about a 70% yield for (R)-**14** when performed under the same conditions as the vinyl esters this time in the presence of activated molecular sieves. However, when the reaction was performed without a solvent, under vacuum to rid the reaction environment of the acetone oxime co-product, the yield jumped up to 87%, with the product, (*R*)-**14**, obtained as a colorless oil (see Table 1).

The saturated myristic (14:0) and palmitic (16:0) acids were introduced to the *sn*-2 position of (*R*)-**14** by the use of the EDCI coupling reaction described above to accomplish the PMB-protected DAG products (R)-**15a** and (*R*)-**15b** as colorless oils in excellent yields (94 and 91%, respectively; Table 2). Their PMB protective group was successfully removed under the DDQ reaction conditions identical to those described above to accomplish the *sn*-2,3-DAG intermediates (*R*)-**16a** and (*R*)-**16b** in excellent yields (95 and 91%, respectively; Table 3). As before, they were obtained in excellent regiopurities, with no signs of acyl migration taking place.

To complete the synthesis of the intended enantiostructured TAGs, (*S*)-**5** and (*S*)-**6**, the final step, like before, involved the chemical coupling of the third and final fatty acid, linoleic acid, to the remaining *sn*-1 position of the *sn*-2,3-DAGs precursors. This was brought about by EDCI and DMAP under identical conditions to those described in previous steps using free linoleic acid. The final TAGs, (*S*)-**5** and (*S*)-**6**, were obtained as colorless oils in excellent yields (94 and 95%, respectively; Table 4).

Table 1, Table 2, Table 3 and Table 4 outline the yields and optical activities of all the acylated intermediates and the final products in the synthesis of the intended SUU’ and USU’ subclass category TAGs obtained so far. The total yields from the (*R*)-solketal starting material to complete the TAGs over the six steps were 48–56%. This means that the average yield for each of the six individual steps of the synthesis ranged roughly between 88.5 and 90.5%, which is quite high. The introduction of the PMB protective group to the (*R*)-solketal offered the lowest yield of 76% and contributed the most to lowering the overall yield.

### 2.3. Chemoenzymatic Synthesis of the Remaining SUU’ Subclass Category TAGs, (S)-***3*** and ***4***

Returning to the problem of the polyenes of PUFAs being slowly destroyed by DDQ in the deprotection of the PMB ethers, for the remaining two SUU’ subclass category TAGs, (*S*)-**3** and (*S*)-**4**, that were to be synthesized by the approach based on the PMB protective group, linoleic acid is attached to the mid position. Because the DDQ reaction could not be adequately adapted to include linoleic acid, an adjustment was clearly needed. Instead of coupling in the first two fatty acids, a saturated fatty acid, followed by linoleic acid, and then deprotecting the PMB ether, it was opted to deprotect with only the saturated fatty acid present at the end position, leaving the mid position open. Then, after deprotection, CAL-B might be used a second time to regioselectively acylate the now-free *sn*-1 position, and, finally, the linoleic acid could be chemically coupled to the *sn*-2 position.

#### 2.3.1. Deprotection with an Open *sn*-2 Position

**Figure 4:**
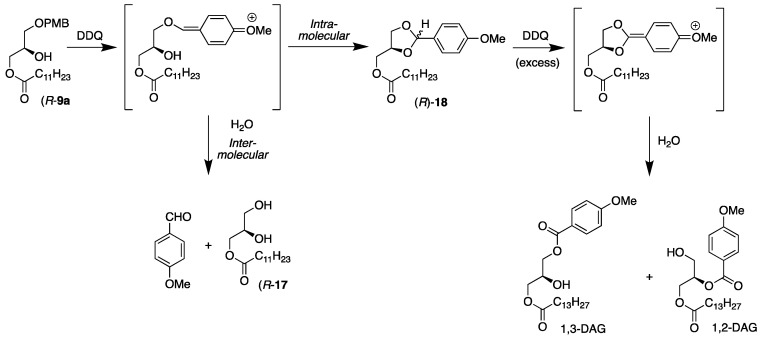
Intermediates and products obtained when (*R*)-**9a** was treated with DDQ to deprotect the PMB–ether moiety in dichloromethane with water at room temperature.

To investigate this option further, the previously described DDQ reaction conditions were applied to the (*R*)-**9a** derivative possessing the saturated lauric acid (C12:0) at the *sn*-3 position (see Figure 4). This reaction yielded quite interesting results. Four compounds were isolated from the reaction mixture, and their structures were elucidated by ^1^H NMR spectroscopy and accurate mass spectrometry studies. The desired monoacylglycerol (MAG) product, (*R*)-**17**, was obtained in a 42% yield, a cyclic acetal, (*R*)-**18**, with the protective group still attached in a 26% yield, a small fraction of *p*-anisaldehyde, and, finally, an unknown compound in a 30% yield.

The unknown product turned out to be a mixture of two *sn*-2,3- and *sn*-1,3-DAG regioisomers in ratios of 70% and 30%, respectively, with a *p*-methoxybenzoate ester present in the molecule. The acetal, (*R*)-**18**, was contaminated with *p*-anisaldehyde, and they were not completely separable in a flash column despite several attempts. Figure 4 outlines the reaction, all the products that had formed, and a rationalization of what happened in the reaction.

As indicated in Figure 4, the reaction began as anticipated by DDQ oxidizing the PMB group to transform it to a quinone-like moiety that is vulnerable to a nucleophilic attack. This time, however, unlike the previous cases, where two fatty acyl groups were present, there were two options for the reaction to proceed further. The reaction can continue as wanted by external water attacking in an intermolecular reaction mode. Second, the free hydroxyl group on the glycerol can also perform an intramolecular nucleophilic attack to form a cyclic acetal. The second option is usually considered as a more likely scenario because of the proximity of the hydroxyl group to the reactive benzyl position.

Like in the previous reactions, 30% excess DDQ was used and, therefore, there was plenty of DDQ present to continue oxidation of the cyclic acetal. This resulted in a second quinone-like moiety that underwent an intermolecular nucleophilic attack with water to form a benzoate attached to the glycerol backbone. It was of interest that the resulting diacylglycerols were formed in a ratio of 70% 1,2-DAG and 30% 1,3-DAG, which is opposite to the thermodynamic composition of 70% 1,3-DAG at an equilibrium involving an acyl migration [22,23,24], indicating that the overall DAG formation was somewhat under a kinetic control. It should be emphasized that under the DDQ deprotection conditions, no acyl migration was observed to take place.

Armed with the newfound knowledge of the process, the reaction was adjusted to minimize the undesired side products. First, the amount of DDQ was lowered to exactly one equivalent to retard the formation of the double-oxidized benzoate DAG regioisomers. Second, to enhance the intermolecular reaction with water over the intramolecular reaction of the free hydroxyl group, the ratio of the water to the dichloromethane in the reaction medium was increased from 1:10 to 1:3. Additionally, the reaction was performed with the magnetic stirrer on a full spin to increase the accessibility of the water in the reaction.

When the reaction was repeated with the new adjustments, the deprotected (*R*)-**17** was isolated in a 72% yield with a minor fraction of the acetal, (*R*)-**18**, which is quite acceptable. Moreover, it was demonstrated that the acetal, (*R*)-**18**, could be converted to the desired (*R*)-**17** in a 91% yield, using elemental iodine along with water in acetonitrile to further improve the yield of the modified reaction. This is the same reaction as was applied to remove the isopropylidene group from the solketal in the second step of the total synthesis.

#### 2.3.2. TAG Synthesis via a Double-Lipase-Step Method

Now, when the MAG (*R*)-**17** had been obtained, the next step involved the acylation of oleic acid as an oxime ester (**13**) to the *sn*-1 end position utilizing CAL-B to form the intended 1,3-DAG intermediate. However, the results from that reaction were disappointing. Because oxime esters are less reactive than vinyl esters, the enzymatic reaction was slow, and stirring overnight was needed for the reaction to proceed to completion. The problem is that the lipase, unlike the previous case, was in a dynamic system with the acylated glycerol. And given enough time, CAL-B started acting on the saturated ester already present in the monoacylglycerol starting material, thus interfering with the intended reaction. The consequences were losses in the regioselectivity of the reaction, lower yields of the desired product, and unreacted starting material present. In other words, the increased reaction time that was needed to introduce the oxime, allowed the lipase to slowly start destroying the desired compound and the regiocontrol of the reaction.

**Figure 5:**
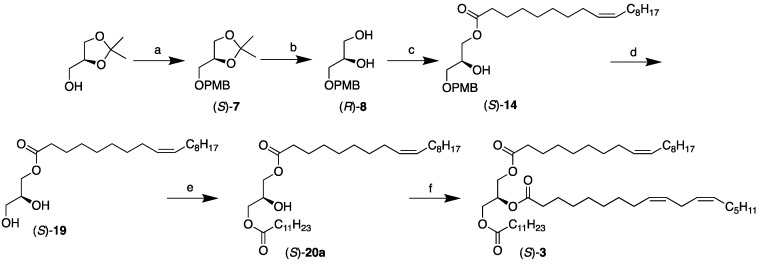
Chemoenzymatic synthesis of the SUU’ subclass category TAGs, (*S*)-**3** and **4** (shown for (*S*)-**3**). Reagents and conditions: (**a**) NaH, THF, then PMB-Cl, reflux (76%); (**b**) I_2_, H_2_O, acetonitrile, r.t. (92%); (**c**) oleic acid acetoxime ester (**13**), CAL-B, vacuum, r.t. (74%); (**d**) DDQ, CH_2_Cl_2_, H_2_O, from 0 °C to r.t. (70%); (**e**) vinyl dodecanoate, CAL-B, CH_2_Cl_2_, r.t. (83%); (**f**) Linoleic acid, EDCI, DMAP, CH_2_Cl_2_, r.t. (97%).

The double-lipase-step method has been successfully used before to synthesize ABC-type TAGs belonging to the SUS’ subclass categories [13]. However, in that case, the lipase reactions involved two saturated fatty acids to be introduced to the end positions as the much faster vinyl esters in two lipase steps, each taking no more than four hours. Therefore, it became evident that if the double-lipase-step method was to be utilized, involving a saturated and a monounsaturated fatty acid to be incorporated into the end positions, the monounsaturated one would need to be introduced first and then the saturated one in the second lipase step. Consequently, the synthesis would need to be initiated from the opposite (*S*)-solketal enantiomer starting material to secure the intended stereochemistry of the resulting TAGs. The accordingly modified approach is outlined in Figure 5.

The first three reactions of the synthesis were identical to those of the opposite enantiomers shown in Figure 2 and Figure 3. Once oleic acid had been introduced to the *sn*-1 position of (*S*)-**8** in a 74% yield, the PMB group of the resulting (*S*)-**14** was deprotected to accomplish the MAG, (*S*)-**19**, in a 70% yield, following the modifications described above. The second lipase step was performed with vinyl esters of lauric acid and palmitic acid to accomplish the resulting 1,3-DAGs, (*S*)-**20a** and (*S*)-**20b**, in 83 and 87% yields, respectively. The reactions only required 4 h to proceed to completion, with no observed acyl migration taking place, as was to be expected. Finally, linoleic acid was incorporated into the *sn*-2 position by the usual chemical coupling involving EDCI and DMAP to complete the desired target molecules, (*S*)-**3** and (*S*)-**4**, that were obtained as colorless oils in 97 and 96% yields, respectively. Table 5 shows the identities, yields, and specific rotations for the final two TAGs and all their intermediates, starting from (*S*)-solketal, via the double-lipase-step approach.

The double-lipase-step method, based on the introduction of a PUFA to the *sn*-2 position with a monounsaturated and a saturated fatty acid present at each of the end positions, afforded the two TAGs belonging to the SUU’ subclass category in 30% overall yields from (*S*)-solketal over six steps. This is significantly lower than the average of 52% overall yields obtained from the PMB-based strategy described above in Figure 2 and Figure 4. However, it must be kept in mind that the average yield for the individual steps of the six-step synthesis is still close to 82%, which cannot be considered as bad. The lower total yields can mainly be attributed to the lipase-promoted oleic acid coupling reaction, which we may be able to improve, and the less efficient deprotection, with the *sn*-2 position being free. Nevertheless, the target compounds were obtained in excellent regiopurities, with no acyl migration occurring during the synthesis. This was established through studies of the quite characteristic glyceryl proton region from the ^1^H NMR spectra of all the individual 1-MAGs, 1,3- and 1,2-DAGs, and TAGs featured in the synthesis. This is demonstrated in Figure S4 of the Supporting Information.

## 3. Materials and Methods

### 3.1. General Information

The ^1^H- and ^13^C-NMR spectra were recorded on a 400 MHz Bruker Avance NEO 400 spectrometer (Bruker Switzerland AG, Faellanden, Switzerland). Chemical shifts (δ) are reported in parts per million (ppm) from tetramethylsilane, with the solvent resonance being used as an internal standard. In all the cases, the solvent was deuterochloroform, which had been filtered through aluminum oxide to eliminate acidic contamination. The coupling constants (J) are given in Hertz (Hz). The following abbreviations are used to describe the multiplicities: s, singlet; d, doublet; t, triplet; q, quartet; p, pentet; dd, doublet of doublets; dt, doublet of triplets; ABq, AB-quartet; m, multiplet. For ^13^C-NMR, the number of carbon nuclei contributing to each signal is indicated in parentheses after the chemical shift value. Infrared spectra were recorded on a Nicolet IS 10 FT-IR spectrometer (Thermo Scientific, Madison, WI, USA), using either sodium chloride windows (NaCl) for liquid compounds, potassium bromide pellets (KBr) for solids, or a diamond ATR crystal that was used for both liquids and solids. The following abbreviations are used to describe the peaks: s, strong; vs, very strong; m, medium; w, weak; br, broad. High-resolution mass spectra (HMRS) were recorded on a Bruker OTOF-Q Compact ESI mass spectrometer (Bruker Daltonic, Bremen, Germany). Optical activities were measured on an Autopol V automatic Polarimeter from Rudolph Research Analytical (Hacketstown, NJ, USA) using a 40T-2.5-100-0.7 Temp Trol polarimetric cell with a 2.5 mm inside diameter, a 100 mm optical length, and a 0.7 mL volume, with c (concentration) referring to g sample/100 mL. Melting points were determined using a Büchi m-560 melting point apparatus. TLC monitoring was conducted on silica plates from SiliCycle, and the plates were developed in 4% PMA solution in methanol. Boric-acid-impregnated silica gel was prepared by dissolving 4 g of boric acid in 100 mL of methanol and then adding 55 g of silica and swirling the resulting slurry for a few minutes. The methanol was then evaporated off, and the silica was dried in vacuo for 6 h at 40 °C.

All the chemicals and solvents were used without further purification unless otherwise stated. Most solvents used, deuterated chloroform (99.8% D), diethyl ether (99.8%), chloroform (≥99.5%), dichloromethane (99.8%), ethanol (≥99.8%), hexane (>97%), methanol (99.9%), and tetrahydrofuran (99.9%) were from Sigma-Aldrich (Steinheim, Germany). Tetrahydrofuran and dichloromethane were dried over molecular sieves and stored under nitrogen. Ethyl acetate and petroleum ether (special boiling point 60–95 °C) were purchased from Brenntag (Essen, Germany) in barrels. The petroleum ether was purified by distillation in a rotary evaporator. All the following chemicals: *p*-anisaldehyde (98%), boric acid (≥99.5%), hydrochloric acid (37%), magnesium sulfate (≥99.5%), phosphomolybdic acid, sodium bicarbonate (≥99.0%), sodium hydride (60% dispersion in mineral oil), sodium sulfate (≥99%), sodium thiosulfate (≥98.5%), (*R*)-solketal (98%, 98% ee), (*S*)-solketal (98%, 99% ee), stearic acid (≥99%), vinyl caprate (>95%), and vinyl laurate (≥99%) were obtained from Sigma-Aldrich. Capric acid (>99%), lauric acid (>99.5%), myristic acid (>99.5%), oleic alcohol, and tetrabutylammonium bromide (99%) were from Acros Organics (Geel, Belgium). 2,3-Dichloro-5,6-dicyano-1,4-benzoquinone (>97%), 1-ethyl-3-(3-dimethylamino propyl) carbodiimide (>98%), *p*-methoxybenzyl chloride (>98%), vinyl myristate (>99%), vinyl palmitate (>96%), and vinyl stearate (>95%) were purchased from TCI Europe (Zwinderecht, Belgium). Arachidic acid, linoleic acid, oleic acid, and palmitic acid were all from Larodan Fine Chemicals (Malmö, Sweden). Acetoxime (98%), 4-dimethylaminopyridine (≥99%), elemental iodine, potassium hydroxide, and sodium chloride (≥99.8%) were obtained from Merck (Darmstadt, Germany). The immobilized *Candida antarctica* lipase B (CAL-B, Novozym 435) was obtained as a gift from Novozymes A/S (Bagsvaerd, Denmark).

### 3.2. Synthesis of the SUU’ Subclass Category TAGs, (S)-***1*** and ***2***

#### 3.2.1. Synthesis of 2,3-*O*-Isopropylidene-1-*O*-(*p*-methoxybenzyl)-*sn*-glycerol, (*R*)-**7**

Sodium hydride (60% mineral oil dispersion, 490 mg, 20.43 mmol) was added to a 250 mL flame-dried two-necked round-bottom flask with a magnetic stirrer and rinsed three times with dry THF (10 mL portions) under a nitrogen atmosphere. After that, a fresh portion of dry THF (15 mL) was added, and the solution was cooled to 0 °C. (*R*)-Solketal (900 mg, 6.81 mmol) was added dropwise to the solution in dry THF, and the mixture was then allowed to reach room temperature and stirred for 1.5 h. After that time, the solution was again cooled to 0 °C, and *p*-methoxybenzyl chloride (1226 mg, 7.83 mmol) was added. Finally, the solution was refluxed for 22 h, after which the mixture had a deep orange color. The reaction was carefully quenched with water and extracted three times with dichloromethane. The combined organic extracts were washed with water and brine, then dried over MgSO_4_ and concentrated in vacuo. The crude concentrate was then purified by flash column chromatography using ethyl acetate:petroleum ether (2:8) as the eluent, affording the product, (*R*)-**7**, as a slightly yellow liquid (1303 mg, 76% yield). TLC (silica, ethyl acetate:petroleum ether, 2:8): Rf = 0.39. [α]^20^_D_ = −1.24 (c. 2.16, CH_2_Cl_2_). IR (NaCl, ν_max_/cm^−1^): 2935 (s), 2865 (s), 1613 (m), 1248 (vs), 1037 (s). ^1^H NMR (400 MHz, CDCl_3_) δ_H_: 7.26 (d, *J* = 8.8 Hz, 2H, Ph-H), 6.88 (d, *J* = 8.8 Hz, 2H, Ph-H), 4.49 (m, 2H, PhCH**_2_**), 4.27 (m, 1H, CH *sn*-2), 4.04 (dd, *J* = 8.3, 6.4 Hz, 1H, CH_2_ *sn*-3), 3.80 (s, 3H, OCH**_3_**), 3.72 (dd, *J* = 8.3, 6.3 Hz, 1H, CH_2_ *sn*-3), 3.52 (dd, *J* = 9.8, 5.7 Hz, 1H, CH_2_ *sn*-1), 3.44 (dd, *J* = 9.8, 5.6 Hz, 1H, CH_2_ *sn*-1), 1.42 (s, 3H, C(CH**_3_**)_2_), 1.36 (s, 3H, C(CH**_3_**)_2_) ppm. ^13^C{H} NMR (101 MHz, CDCl_3_) δ_C_: 159.3, 130.0, 129.4 (2), 113.8 (2), 109.4, 74.7, 73.2, 70.8, 66.9, 55.3, 26.8, 25.4 ppm. HRMS (ESI) *m*/*z*: [M + Na]^+^ calcd. for C_14_H_20_O_4_Na 275.1254; found, 275.1255.

#### 3.2.2. Synthesis of 1-*O*-(*p*-Methoxybenzyl)-*sn*-glycerol, (*S*)-**8**

PMB–solketal, (*R*)-**7** (1300 mg, 5.15 mmol), in acetonitrile (25 mL) was added to a 50 mL round-bottom flask equipped with a magnetic stirrer. Subsequently, elemental iodine (392 mg, 1.54 mmol) and water (1 mL) were added to the solution, and it allowed to stir for 22 h at room temperature under a nitrogen atmosphere. After that time, the solution was quenched with 50 mL of Na_2_S_2_O_3_ (20% *w*/*w* aqueous solution) and extracted three times with ethyl acetate. The combined organic layers were dried over Na_2_SO_4_ and concentrated in vacuo. The crude concentrate was then purified by flash column chromatography, first using ethyl acetate/petroleum ether (3:7) as the eluent and then gradually increasing the proportion of ethyl acetate until the eluent was pure ethyl acetate. That afforded the product, (*S*)-**8**, which solidified upon drying under vacuum into a slightly yellow solid. It was then recrystallized in hexane, which afforded colorless fine needles (1092 mg, 97% yield). TLC (silica, ethyl acetate:petroleum ether, 30:70): Rf = 0.11. Mp. 43.1–43.6 °C. [α]^20^_D_ = +2.48 (c. 1.73, CH_2_Cl_2_). IR (NaCl, ν_max_/cm^−1^): 3389 (br), 2935 (s), 2837 (vs), 1612 (m), 1463 (m), 1247 (s), 1033 (vs). ^1^H NMR (400 MHz, CDCl_3_) δ_H_: 7.25 (d, *J* = 8.7 Hz, 2H, Ph-H), 6.89 (d, *J* = 8.7 Hz, 2H, Ph-H), 4.49 (s, 2H, PhCH**_2_**), 4.88 (m, 1H, CH *sn*-2), 3.81 (s, 3H, OCH**_3_**), 3.71 (dd, *J* = 11.5, 3.8 Hz, 1H, CH_2_ *sn*-3), 3.63 (dd, *J* = 11.5, 5.6 Hz, 1H, CH_2_ *sn*-3), 3.57 (dd, *J* = 9.6, 3.6 Hz, 1H, CH_2_ *sn*-1), 3.52 (dd, *J* = 9.6, 3.5 Hz, 1H, CH_2_ *sn*-1), 2.59 (br s, 1H, OH), 2.10 (br s, 1H, OH) ppm. ^13^C{H} NMR (101 MHz, CDCl_3_) δ_C_: 159.4, 129.8, 129.5 (2), 113.9 (2), 73.4, 71.5, 70. 6, 63.9, 55.3 ppm. HRMS (ESI) *m*/*z*: [M + Na]^+^ calcd. for C_11_H_16_O_4_Na 235.0941; found, 235.0943.

#### 3.2.3. Synthesis of 3-Dodecanoyl-1-*O*-(*p*-methoxybenzyl)-*sn*-glycerol, (*R*)-**9a**

PMB-protected glycerol, (*S*)-**8** (130 mg, 0.55 mmol), and vinyl laurate (160 mg, 0.71 mmol) dissolved in dichloromethane (3 mL) were added under slow magnetic stirring to a 10 mL round-bottom flask. Subsequently, immobilized *Candida antarctica* lipase (CAL-B) (28 mg, 10% *w*/*w*) was added, and the atmosphere was replaced with nitrogen gas. The mixture was allowed to stir for 4 h while being monitored by TLC. After that time, the reaction was complete, and the lipase was filtered off. The solvent was removed in vacuo, and the crude concentrate was washed with a 15 mg/mL NaHCO_3_/methanol (1:1) solution and then extracted twice with hexane. The combined organic extracts were dried over Na_2_SO_4_ and concentrated in vacuo. The crude product was then purified by flash column chromatography with 4% boric-acid-impregnated silica gel, using ethyl acetate:hexane (2:8) as the eluent, affording the product, (I)-**9a**, as a colorless liquid (231 mg, quantitative yield). TLC (silica, ethyl acetate:petroleum ether, 20:80): Rf = 0.27. [α]^20^_D_ = −1.28 (c. 2.50, CH_2_Cl_2_). IR (NaCl, ν_max_/cm^−1^): 3449 (br), 2925 (s), 2854 (vs), 1736 (vs), 1612 (m), 1466 (m), 1377 (m), 1248 (s), 1037 (m). ^1^H NMR (400 MHz, CDCl_3_) δ_H_: 7.25 (d, *J* = 9.0 Hz, 2H, Ph-H), 6.88 (d, *J* = 8.7 Hz, 2H, Ph-H), 4.49 (s, 2H, PhCH**_2_**), 4.17 (dd, *J* = 11.5, 4.4 Hz, 1H, CH_2_ *sn*-3), 4.12 (dd, *J* = 11.5, 6.0 Hz, 1H, CH_2_ *sn*-3), 4.01 (m, 1H, CH *sn*-2), 3.81 (s, 3H, OCH**_3_**), 3.52 (dd, *J* = 9.6, 4.3 Hz, 1H, CH_2_ *sn*-1), 3.46 (dd, *J* = 9.6, 6.2 Hz, 1H, CH_2_ *sn*-1), 2.49 (br s, 1H, OH), 2.32 (t, *J* = 7.6 Hz, 2H, CH**_2_**COO), 1.61 (m, 2H, C*H***_2_**CH_2_COO), 1.29–1.24 (m, 16H, CH**_2_**), 0.88 (t, *J* = 6.8 Hz, 3H, CH_2_C*H***_3_**) ppm. ^13^C{H} NMR (101 MHz, CDCl_3_) δ_C_: 174.1, 159.5, 129.9, 129.6 (2), 114.0 (2), 73.3, 70.7, 69.1, 65.5, 55.4, 34.3, 32.1, 29.7, 29.6, 29.45, 29.4 (2), 29.3, 25.1, 22.8, 14.3 ppm. HRMS (ESI) *m*/*z*: [M + Na]^+^ calcd. for C_23_H_38_O_5_Na 417.2611; found, 417.2610.

#### 3.2.4. Synthesis of (*R*)-**9b** and (*R*)-**9c**

See the full experimental details in the Supplementary Materials.

#### 3.2.5. Synthesis of 3-Dodecanoyl-1-*O*-(*p*-methoxybenzyl)-2-[(9*Z*)-octadec-9-enoyl)]-*sn*-glycerol, (*R*)-**10a**

Monoacylglycerol, (*R*)-**9a** (200 mg, 0.51 mmol), and oleic acid (165 mg, 0.58 mmol) in dry dichloromethane (5 mL) were added to a flame-dried 10 mL round-bottom flask equipped with a magnetic stirrer. Then, 4-dimethylaminopyridine (DMAP) (50 mg, 0.41 mmol) and 1-ethyl-3-(3-dimethylaminopropyl)carbodiimide (EDCI) (117 mg, 0.61 mmol) were added to the solution and stirred at room temperature under nitrogen for 20 h. After that time, the reaction mixture was passed through a short column packed with silica gel using ethyl acetate. The solvent was removed in vacuo, and the crude concentrate was then purified by flash column chromatography using ethyl acetate/hexane (1:9) as the eluent, affording the product, (*R*)-**10a**, as a colorless liquid (188mg, 99% yield). TLC (silica, ethyl acetate:petroleum ether, 20:80): Rf = 0.60. [α]^20^_D_ = −6.77 (c. 1.92, CH_2_Cl_2_). IR (NaCl, ν_max_/cm^−1^): 3003 (m), 2925 (s), 2854 (s), 1743 (s), 1613 (m), 1464 (m), 1248 (s), 1172 (s), 1038 (m). ^1^H NMR (400 MHz, CDCl_3_) δ_H_: 7.23 (d, *J* = 8.8 Hz, 2H, Ph-H), 6.87 (d, *J* = 8.8 Hz, 2H, Ph-H), 5.34 (m, 2H, =CH), 5.23 (m, 1H, CH *sn*-2), 4.45 (ABq, Δδ_AB_ = 0.04, *J* = 11.8, 2H, PhCH**_2_**), 4.33 (dd, *J* = 11.9, 3.9 Hz, 1H, CH_2_ *sn*-3), 4.17 (dd, *J* = 11.9, 6.5 Hz, 1H, CH_2_ *sn*-3), 3.80 (s, 3H, OCH**_3_**), 3.55 (dd, *J* = 5.2, 1.3 Hz, 2H, CH_2_ *sn*-1), 2.31 (t, *J* = 7.6 Hz, 2H, CH**_2_**COO SFA), 2.27 (t, *J* = 7.6 Hz, 2H, CH**_2_**COO MUFA), 2.01 (m, 4H, C*H***_2_**CH=), 1.65–1.56 (m, 4H, C*H***_2_**CH_2_COO), 1.36–1.22 (m, 36H, CH**_2_**), 0.88 (t, *J* = 6.7 Hz, 6H, CH_2_C*H***_3_**) ppm. ^13^C{H} NMR (101 MHz, CDCl_3_) δ_C_: 173.6, 173.2, 159.5, 130.2, 129.92, 129.87, 129.5 (2), 114.0 (2), 73.1, 70.2, 68.1, 62.8, 55.4, 34.5, 34.3, 32.1 (2), 29.92, 29.87, 29.8, 29.7, 29.6 (2), 29.49 (2), 29.47 (2), 29.44, 29.36, 29.3 (2), 29.2, 27.4, 27.3, 25.1, 25.0, 22.8, 14.3 (2) ppm. [M + Na]^+^ calcd. for C_41_H_70_O_6_Na 681.5065; found, 681.5050.

#### 3.2.6. Synthesis of (*R*)-**10b**, (*R*)-**10c**, (*R*)-**10d**, and (*R*)-**10e**

See the full experimental details in the Supplementary Materials.

#### 3.2.7. Synthesis of 3-Dodecanoyl-2-[(9*Z*)-octadec-9-enoyl)]-*sn*-glycerol, (*R*)-**11a**

Diacylglycerol, (*R*)-**10a** (306 mg, 0.465 mmol), and dichloromethane (6 mL) were added to a 25 mL round-bottom flask equipped with a magnetic stirrer. Water (1 mL) was pipetted into the solution, and it was cooled down to 0 °C. Subsequently 2,3-dichloro-5,6-dicyano-1,4-benzoquinone (DDQ) (137 mg, 0.60 mmol) was added, which turned the solution to a dark green color. The mixture was stirred under nitrogen for an hour and then the cooling bath was removed. It was allowed to stir for an additional three hours at room temperature, during which the color slowly changed to colorless with a bright red aqueous phase. When all the dark color had vanished, the reaction was complete, as indicated by TLC monitoring. The reaction mixture was extracted three times with dichloromethane, and the combined organic layers were washed with NaHCO_3_, water, and brine. Then, they were dried over MgSO_4_ and concentrated in vacuo, and the crude concentrate was then purified by flash column chromatography with 4% boric-acid-impregnated silica gel, using ethyl acetate:hexane (1:9) as the eluent, affording the product, (*R*)-**11a**, as a colorless liquid (227 mg, 91% yield). TLC (silica, ethyl acetate:petroleum ether, 20:80): Rf = 0.41. [α]^20^_D_ = +2.43 (c. 4.24, CH_2_Cl_2_). IR (NaCl, ν_max_/cm^−1^): 3480 (br), 3004 (m), 2925 (vs), 2854 (vs), 1744 (s), 1466 (m), 1352 (m), 1167 (s). ^1^H NMR (400 MHz, CDCl_3_) δ_H_: 5.34 (m, 2H, =CH), 5.08 (p, *J* = 4.9 Hz, 1H, CH *sn*-2), 4.32 (dd, *J* = 12.0, 4.6 Hz, 1H, CH_2_ *sn*-3), 4.23 (dd, *J* = 12.0, 5.7 Hz, 1H, CH_2_ *sn*-3), 3.72 (br s, 2H, CH_2_ *sn*-1), 2.34 (t, *J* = 7.5 Hz, 2H, CH**_2_**COO SFA), 2.32 (t, *J* = 7.6 Hz, 2H, CH**_2_**COO MUFA), 2.09 (br s, 1H, OH), 2.01 (q, *J* = 6.6 Hz, 4H, C*H***_2_**CH=), 1.62 (m, 4H, C*H***_2_**CH_2_COO), 1.39–1.22 (m, 36H, CH**_2_**), 0.88 (t, *J* = 6.7, 6H, CH_2_C*H***_3_**) ppm. ^13^C{H} NMR (101 MHz, CDCl_3_) δ_C_: 173.9, 173.5, 130.2, 129.8, 72.3, 62.1, 61.7, 34.4, 34.2, 32.0 (2), 29.9, 29.84, 29.75 (2), 29.7, 29.6, 29.5 (2), 29.4, 29.32, 29.29, 29.25 (2), 29.2, 27.4, 27.3, 25.1, 25.0, 22.8 (2), 14.3 (2) ppm. [M + Na]^+^ calcd. for C_33_H_62_O_5_Na 561.4489; found, 561.4470.

#### 3.2.8. Synthesis of 2-[(9*Z*)-Octadec-9-enoyl)]-3-tetradecanoyl-*sn*-glycerol, (*R*)-**11b** and (*R*)-**11c**

See the full experimental details in the Supplementary Materials.

#### 3.2.9. Synthesis of 3-Hexadecanoyl-2-[(9*Z*,12*Z*)-octadeca-9,12-dienoyl)]-*sn*-glycerol, (*R*)-**11e**

Diacylglycerol, (*R*)-**10e** (1297 mg, 1.82 mmol), and dichloromethane (5 mL) were added to a 50 mL round-bottom flask equipped with a magnetic stirrer. Then, water (3 mL) was pipetted into the solution. Subsequently, 2,3-dichloro-5,6-dicyano-1,4-benzoquinone (412 mg, 1.82 mmol) was dissolved in dichloromethane (10 mL) and slowly added dropwise to the solution over a 30 min period. The mixture was stirred vigorously for additional 1.5 h until the characteristic dark color had vanished. The reaction mixture was extracted three times with dichloromethane, and the combined organic layers were washed with NaHCO_3_, water, and brine. Then, they were dried over Na_2_SO_4_ and concentrated in vacuo, and the crude concentrate was then purified by flash column chromatography with 4% boric-acid-impregnated silica gel, using ethyl acetate:hexane (1:9) as the eluent, affording the product, (*R*)-**11e**, as a colorless liquid (390 mg, 36% yield) along with unreacted (*R*)-**10e** (562 mg, 43% recovery). TLC (silica, ethyl acetate:petroleum ether, 20:80): Rf = 0.40. [α]^20^_D_ = +2.45 (c. 2.57, CH_2_Cl_2_). IR (ATR, ν_max_/cm^−1^): 3213 (br), 3009 (m), 2925 (vs), 2853 (vs), 1713 (vs), 1465 (m), 1349 (m), 1162 (s). ^1^H NMR (400 MHz, CDCl_3_) δ_H_: 5.36 (m, 4H, =CH), 5.08 (m, 1H, CH *sn*-2), 4.32 (dd, *J* = 11.9, 4.6 Hz, 1H, CH_2_ *sn*-3), 4.23 (dd, *J* = 11.9, 5.6 Hz, 1H, CH_2_ *sn*-3), 3.73 (m, 2H, CH_2_ *sn*-1), 2.77 (t, *J* = 6.6 Hz, 2H, =CHC*H***_2_**CH=), 2.32 (t, *J* = 7.7 Hz, 2H, CH**_2_**COO PUFA), 2.31 (t, *J* = 7.6 Hz, 4H, CH**_2_**COO SFA), 2.05 (q, *J* = 6.8 Hz, 4H, C*H***_2_**CH=), 1.65–1.58 (m, 4H, C*H***_2_**CH_2_COO), 1.33–1.25 (m, 38H, CH**_2_**), 0.89 (t, *J* = 6.9, 3H, CH_2_C*H***_3_** PUFA), 0.88 (t, *J* = 7.0, 6H, CH_2_C*H***_3_**) ppm. ^13^C{H} NMR (101 MHz, CDCl_3_) δ_C_: 173.9, 173.5, 130.4, 130.1, 128.2, 128.0, 72.3, 62.1, 61.7, 34.4, 34.3, 32.1, 31.7, 29.9 (3), 29.81 (2), 29.76, 29.6, 29.51, 29.50, 29.4, 29.33, 29.27 (2), 29.2, 27.4 (2), 25.8 (2), 25.1, 25.0, 22.8, 22.7, 14.3, 14.2 ppm. [M + Na]^+^ calcd. for C_37_H_68_O_5_Na 615.4964; found, 615.5067.

#### 3.2.10. Synthesis of 3-Dodecanoyl-1-[(9*Z*,12*Z*)-octadeca-9,12-dienoyl)]-2-[(9*Z*)-octadec-9-enoyl)]-*sn*-glycerol, (*S*)-**1**

Diacylglycerol, (*R*)-**11a** (72 mg, 0.13 mmol), and linoleic acid (43 mg, 0.15 mmol) in dry dichloromethane (3 mL) were added to a 10 mL flame-dried round-bottom flask equipped with a magnetic stirrer. Then, 4-dimethylaminopyridine (DMAP) (13 mg, 0.11 mmol) and 1-ethyl-3-(3-dimethylaminopropyl)carbodiimide (EDCI) (31 mg, 0.16 mmol) were added to the solution and stirred at room temperature under nitrogen for 20 h. After that time, the reaction mixture was passed through a short column packed with silica gel, using ethyl acetate. The solvent was removed in vacuo, and the crude concentrate was then purified by flash column chromatography using ethyl acetate:hexane (1:19) as the eluent, affording the product, (*S*)-**1**, as a colorless liquid (91 mg, 85% yield). TLC (silica, ethyl acetate:petroleum ether, 20:80): Rf = 0.75. [α]^20^_D_ = +0.03 (c. 2.96, CH_2_Cl_2_). IR (NaCl, ν_max_/cm^−1^): 3008 (s), 2926 (vs), 2855 (s), 1747 (s), 1464 (m), 1378 (m), 1161 (s). ^1^H NMR (400 MHz, CDCl_3_) δ_H_: 5.35 (m, 6H, =CH), 5.26 (m, 1H, CH *sn*-2), 4.29 (dd, *J* = 11.9, 4.3 Hz, 2H, CH_2_ *sn*-1/*sn*-3), 4.14 (dd, *J* = 11.9, 6.0 Hz, 2H, CH_2_ *sn*-1/*sn*-3), 2.77 (t, *J* = 6.6 Hz, 2H, =CHC*H***_2_**CH=), 2.32 (t, *J* = 7.5 Hz, 2H, CH**_2_**COO), 2.31 (t, *J* = 7.6 Hz, 4H, CH**_2_**COO), 2.04 (q, *J* = 6.8 Hz, 4H, C*H***_2_**CH= MUFA), 2.01 (q, *J* = 6.6 Hz, 4H, C*H***_2_**CH= PUFA), 1.65–1.57 (m, 6H, C*H***_2_**CH_2_COO), 1.38–1.21 (m, 50H, CH**_2_**), 0.89 (t, *J* = 6.9, 3H, CH_2_C*H***_3_** PUFA), 0.88 (t, *J* = 6.7, 6H, CH_2_C*H***_3_**) ppm. ^13^C{H} NMR (101 MHz, CDCl_3_) δ_C_: 173.43, 173.38, 173.0, 130.4, 130.17, 130.15, 129.8, 128.2, 128.0, 69.0, 62.2 (2), 34.3, 34.20, 34.17, 32.1 (2), 31.7, 29.91, 29.87, 29.8 (3), 29.7, 29.6, 29.49 (2), 29.47 (2), 29.41, 29.35, 29.32, 29.28, 29.27 (2), 29.23, 29.20, 27.4, 27.34 (2), 27.32, 25.8, 25.03, 25.01, 24.98, 22.8 (2), 22.7, 14.3 (2), 14.2 ppm. [M + Na]^+^ calcd. for C_51_H_92_O_6_Na 823.6786; found, 823.6766.

#### 3.2.11. Synthesis of (*S*)-**2**, (*R*)-**12a**, (*R*)-**12b**, (*R*)-**12c**, (*R*)-**12d**, (*R*)-**12e**, and (*R*)-**12f**

See the full experimental details in the Supplementary Materials.

### 3.3. Synthesis of the USU’ Subclass Category TAGs, (S)-***5*** and ***6***

#### 3.3.1. Synthesis of Oleic Acid Acetoxime Ester, **13**

Oleic acid (500 mg, 1.77 mmol) and dry dichloromethane (8 mL) were added to a 100 mL flame-dried two-necked round-bottom flask equipped with a magnetic stirrer. Acetone oxime (130 mg, 1.77 mmol), DMAP (43 mg, 0.35 mmol), and EDCI (407 mg, 2.12 mmol) were added to the solution, and it was stirred at room temperature under nitrogen for 20 h. After that time, the reaction mixture was flushed through a short column packed with silica gel, using ethyl acetate. The solvent was removed in vacuo, and the crude concentrate was then purified by flash column chromatography using ethyl acetate:hexane (1:9) as the eluent, affording the product (**13**) as a colorless liquid (596 mg, quantitative yields). TLC (silica, ethyl acetate:petroleum ether, 30:70): Rf = 0.59. IR (NaCl, ν_max_/cm^−1^): 2925 (s), 2854 (vs), 1765 (vs), 1654 (w), 1460 (m), 1377 (m), 1271 (m), 1136(s). ^1^H NMR (400 MHz, CDCl_3_) δ_H_: 5.34 (m, 2H, =CH), 2.40 (t, *J* = 7.6 Hz, 2H, CH_2_COO), 2.05 (s, 3H, (CH_3_)_2_C=N), 2.01 (m, 4H, C*H*_2_CH=), 1.99 (s, 3H, (CH_3_)_2_C=N), 1.69 (p, *J* = 7.5 Hz, 2H, C*H*_2_CH_2_COO), 1.38–1.23 (m, 20H, CH_2_), 0.87 (t, *J* = 6.7 Hz, 3H, CH_2_C*H*_3_) ppm. ^13^C{H} NMR (101 MHz, CDCl_3_) δ_C_: 171.4, 163.8, 130.2, 129.9, 33.2, 32.0, 29.9, 29.8, 29.7, 29.5 (2), 29.3, 29.2 (2), 27.4, 27.3, 25.1, 22.8, 22.2, 17.1, 14.3 ppm. [M + Na]^+^ calcd. for C_21_H_39_O_2_NNa 360.2873; found, 360.2879.

#### 3.3.2. Synthesis of 1-*O*-(*p*-Methoxybenzyl)-3-[(9*Z*)-octadec-9-enoyl)]-*sn*-glycerol, (*R*)-**14**

PMB-protected glycerol, (*S*)-**8** (300 mg, 1.41 mmol), and oleic acid acetoxime ester (**13**) (573 mg, 1.70 mmol) were added to a flame-dried 5 mL round-bottom flask. Subsequently, immobilized *Candida antarctica* lipase B (CAL-B) (70 mg, 8% *w*/*w*) was added; the flask was connected to a vacuum pump system (10^−2^ mmHg), and the resulting mixture was stirred at room temperature for 6 h. After that time, the vacuum was disconnected, and additional CAL-B (10 mg) along with dried dichloromethane (1.5 mL) were added to the flask. The mixture was allowed to stir under a nitrogen atmosphere overnight. Then, the reaction was complete, and the lipase was filtered off. The solvent was removed in vacuo, and the crude concentrate was then purified by flash column chromatography with 4% boric-acid-impregnated silica gel, using ethyl acetate:hexane (2:8) as the eluent, affording the product, (*R*)-**14**, as a colorless liquid (585 mg, 87% yield). TLC (silica, ethyl acetate:petroleum ether, 30:70): Rf = 0.50. [α]^20^_D_ = −0.72 (c. 1.67, CH_2_Cl_2_). IR (NaCl, ν_max_/cm^−1^): 3449 (br), 2925 (s), 2854 (vs), 1739 (vs), 1612 (m), 1463 (m), 1377 (m), 1248 (s). ^1^H NMR (400 MHz, CDCl_3_) δ_H_: 7.25 (d, *J* = 9.1 Hz, 2H, Ph-H), 6.88 (d, *J* = 8.8 Hz, 2H, Ph-H), 5.34 (m, 2H, =CH), 4.49 (s, 2H, PhCH_2_), 4.17 (dd, *J* = 11.5, 4.5 Hz, 1H, CH_2_ *sn*-3), 4.12 (dd, *J* = 11.5, 6.1 Hz, 1H, CH_2_ *sn*-3), 4.01 (m, 1H, CH *sn*-2), 3.81 (s, 3H, OCH_3_), 3.52 (dd, *J* = 9.6, 4.3 Hz, 1H, CH_2_ *sn*-1), 3.46 (dd, *J* = 9.6, 6.1 Hz, 1H, CH_2_ *sn*-1), 2.48 (d, *J* = 4.9 Hz, 1H, OH), 2.32 (t, *J* = 7.6 Hz, 2H, CH_2_COO), 2.00 (m, 4H, C*H*_2_CH=), 1.61 (m, 2H, C*H*_2_CH_2_COO), 1.38–1.23 (m, 20H, CH_2_), 0.88 (t, *J* = 6.7 Hz, 3H, CH_2_C*H*_3_) ppm. ^13^C{H} NMR (101 MHz, CDCl_3_) δ_C_: 174.1, 159.5, 130.2, 129.89, 129.87, 129.6 (2), 114.0 (2), 73.3, 70.7, 69.1, 65.5, 55.4, 34.3, 32.0, 29.9, 29.8, 29.7, 29.5 (2), 29.31, 29.25 (2), 27.4, 27.3, 25.0, 22.8, 14.3 ppm. [M + Na]^+^ calcd. for C_29_H_48_O_5_Na 499.3394; found, 499.3353.

#### 3.3.3. Synthesis of (*R*)-**15a**, (*R*)-**15b**, (*R*)-**16a**, (*R*)-**16b**, (*S*)-**5**, and (*S*)-**6**

See the full experimental details in the Supplementary Materials.

### 3.4. Synthesis of the SUU’ Subclass Category TAGs, (S)-***3*** and ***4***

#### 3.4.1. Synthesis of 3-Dodecanoyl-*sn*-glycerol, (*R*)-**17**

Acylglycerol, (*R*)-**9a** (200 mg, 0.51 mmol), dissolved in dichloromethane (3 mL) was added to a 10 mL round-bottom flask equipped with a magnetic stirrer. Water (1 mL) was pipetted into the solution, which was cooled down to 0 °C. Subsequently, DDQ (115 mg, 0.51 mmol) was added, which turned the solution to a dark green color. The cooling bath was removed after 30 min. The solution was stirred under a nitrogen atmosphere overnight with the magnetic stirrer on full speed. After that time, the reaction mixture was extracted three times with dichloromethane, and the combined organic layers were washed with water, aqueous NaHCO_3_, and brine. Then, they were dried over Na_2_SO_4_, concentrated in vacuo, and the crude concentrate was then purified by flash column chromatography with 4% boric-acid-impregnated silica gel, using a gradient solvent system from ethyl acetate:hexane (1:9) to ethyl acetate:hexane (1:1) as the eluents, affording the product, (*R*)-**17**, as a white, waxy solid (139 mg, 72% yield). TLC (silica, ethyl acetate:petroleum ether, 50:50): Rf = 0.18. Mp. 53.8–54.3 °C. [α]^20^_D_ = −1.62 (c. 1.11, CH_2_Cl_2_). IR (ATR, ν_max_/cm^−1^): 3300 (br), 2956 (m), 2918 (vs), 2849 (vs), 1733 (s), 1463 (m), 1175 (s). ^1^H NMR (400 MHz, CDCl_3_) δ_H_: δ 4.21 (dd, *J* = 11.8, 4.6 Hz, 1H, CH_2_ *sn*-3), 4.15 (dd, *J* = 11.6, 6.1 Hz, 1H, CH_2_ *sn*-3), 3.93 (m, 1H, CH *sn*-2), 3.70 (ddd, *J* = 10.4, 6.4, 4.1 Hz, 1H, CH_2_ *sn*-1), 3.60 (dd, *J* = 11.4, 5.6 Hz, 1H, CH_2_ *sn*-1), 2.51 (d, *J* = 5.1 Hz, 1H, CHO*H*), 2.35 (t, *J* = 6.1, 2H, C*H***_2_**COO), 2.07 (t, *J* = 6.1 Hz, 1H, CH_2_O*H*),1.64 (p, *J* = 7.6 Hz, 2H, C*H***_2_**CH_2_COO), 1.32–1.22 (m, 16H, CH**_2_**), 0.88 (t, *J* = 6.9, 3H, CH_2_C*H***_3_**) ppm. ^13^C{H} NMR (101 MHz, CDCl_3_) δ_C_: 174.5, 70.4, 65.3, 63.5, 34.3, 32.1, 29.7 (2), 29.6, 29.5, 29.4, 29.3, 25.1, 22.8, 14.3 ppm. [M + Na]^+^ calcd. for C_15_H_30_O_4_Na 297.2036; found, 297.2023.

#### 3.4.2. Synthesis of [2-(*p*-methoxyphenyl)-1,3-dioxolan-4-yl]methyl Dodecanoate, (*R*)-**18**

Acylglycerol, (*R*)-**9a** (100 mg, 0.25 mmol), and dichloromethane (3 mL) were added to a 10 mL round-bottom flask equipped with a magnetic stirrer. Water (0.5 mL) was pipetted into the solution, and it was cooled down to 0 °C. Subsequently DDQ (75 mg, 0.33 mmol) was added, which turned the solution to a dark green color. The mixture was stirred under nitrogen for 30 min and then the cooling bath was removed. It was allowed to stir for an additional five hours at room temperature, during which the color slowly changed to bright red. When the dark color had vanished, the reaction was finished, and the mixture was extracted three times with dichloromethane. The combined organic layers were washed with aqueous NaHCO_3_, water, and brine. Then, they were dried over Na_2_SO_4_, concentrated in vacuo, and the crude concentrate was then purified by flash column chromatography with 4% boric-acid-impregnated silica gel, using ethyl acetate:petroleum ether (1:9) as the eluent, affording the product, (*R*)-**18**, as a colorless liquid (38 mg, 54% yield). TLC (silica, ethyl acetate:petroleum ether, 50:50): Rf = 0.71. [α]^20^_D_ = +5.63 (c. 1.92, CH_2_Cl_2_). IR (ATR, ν_max_/cm^−1^): 2923 (s), 2853 (s), 1737 (s), 1614 (w), 1464 (m), 1248 (vs), 1160 (s), 1032 (s). ^1^H NMR (400 MHz, CDCl_3_) δ_H_: 7.40 (dd, *J* = 8.7, 6.2 Hz, 2H, Ph-H), 6.90 (d, *J* = 7.8 Hz, 2H, Ph-H), 5.89 (s, 0.45H, OCH**-**Ph), 5.78 (s, 0.55H, OCH**-**Ph), 4.48 (m, 0.45H, CH *sn*-2), 4.43 (m, 0.55H, CH *sn*-2), 4.28 (dd, *J* = 6.8, 1.8 Hz, 0.5H, CH_2_ *sn*-1), 4.23 (m, 2H, CH_2_ *sn*-3), 4.10 (dd, *J* = 8.5, 7.1 Hz, 0.5H, CH_2_ *sn*-1), 3.95 (dd, *J* = 8.4, 5.1 Hz, 0.5H, CH_2_ *sn*-1), 3.81 (s, 3H, OCH**_3_**), 3.77 (dd, *J* = 8.5, 6.8 Hz, 0.5H, CH_2_ *sn*-1), 2.35 (m, 2H, CH**_2_**COO), 1.63 (p, *J* = 7.2 Hz, 2H, C*H***_2_**CH_2_COO), 1.33–1.24 (m, 16H, CH**_2_**), 0.88 (t, *J* = 6.9 Hz, 3H, CH_2_C*H***_3_**) ppm. ^13^C{H} NMR (101 MHz, CDCl_3_) δ_C_: 173.82, 173.79, 160.8, 160.6, 129.7, 129.2, 128.3 (2), 128.1 (2), 113.93 (2), 113.91 (2), 104.8, 100.0, 74.2, 74.0, 67.6, 67.4, 64.6, 64.2, 55. 5 (2), 34.3 (2), 32.1 (2), 29.7 (4), 29.6 (2), 29.5 (2), 29.4 (2), 29.3 (2), 25.1 (2), 22.8 (2), 14.3 (2) ppm. [M + Na]^+^ calcd. for C_23_H_36_O_5_Na 415.2455; found, 415.2445.

#### 3.4.3. Conversion from Acetal, (*R*)-**18**, to (*R*)-**17**

Acetal, (*R*)-**18** (32 mg, 0.08 mmol), in acetonitrile (1 mL) was added to a 10 mL round-bottom flask equipped with a magnetic stirrer. Subsequently, elemental iodine (6 mg, 0.02 mmol) and water (20 μL) were added to the solution, and it was allowed to stir for 22 h at room temperature under a nitrogen atmosphere. After that time, the solution was quenched with Na_2_S_2_O_3_ (20% *w*/*w* aqueous solution) and extracted three times with ethyl acetate. The combined organic layers were dried over Na_2_SO_4_ and concentrated in vacuo. The crude concentrate was then purified by flash column chromatography with 4% boric acid-impregnated-silica gel, using a gradient solvent system from ethyl acetate:hexane (1:9) to ethyl acetate:hexane (1:1) as the eluents. That afforded the product, (*R*)-**17**, as a slightly yellow solid (20 mg, 91% yield).

#### 3.4.4. Synthesis of (*R*)-**8**, (*S*)-**14**, (*S*)-**19**, (*S*)-**20a**, (*S*)-**20b**, (*S*)-**3**, and (*S*)-**4**.

See the full experimental details in the Supplementary Materials.

## 4. Conclusions

The lack of enantiopure TAGs as reference standards has been a major barrier in the enantiospecific analysis of intact chiral TAGs in natural fats and oils to determine the separation order of TAG enantiomers. In recent years, we have contributed various types of enantiostructured TAGs for that purpose, including AAB- and ABC-type TAG enantiomers. In an attempt to expand the existing focused-compound library of such standards, the current work was undertaken and succeeded in the synthesis of ABC-type TAG enantiomers belonging to both subclass categories of TAGs, constituting two non-identical unsaturated fatty acids (oleic acid and linoleic acid) along with a saturated fatty acid. Herein, we reported the synthesis of six enantiostructured ABC-type TAGs. Four of them belong to the SUU’ subclass category, having unsaturated fatty acids located at the *sn*-1 and *sn*-2 positions, with a saturated fatty acid at the *sn*-3 position of the glycerol skeleton. The two remaining TAGs belong to the USU’ subclass category, with different unsaturated fatty acids present at the *sn*-1 and *sn*-3 end positions and the saturated fatty acid at the *sn*-2 position.

As before, the highly regioselective immobilized *Candida antarctica* lipase (CAL-B) played a crucial role in the regiocontrol of the six-step chemoenzymatic synthesis that was started from enantiopure solketals. The use of the ether PMB protective group, indeed, allowed the incorporation of two different unsaturated fatty acids into the glycerol backbone. However, its use was limited to the presence of only a monounsaturated fatty acid in the deprotection step because the higher unsaturation did not survive the mild oxidative treatment. That created challenges when dealing with the TAGs belonging to the SUU’ subclass category, which involved the PUFA at the *sn*-2 position, causing the overall yields to drop from an average of 52% over the six steps down to 30%, which is still quite acceptable.

This is, however, an important achievement because we were unable to prepare TAGs possessing two dissimilar unsaturated fatty acids by the previous strategy based on the use of the benzyl-protecting moiety. This highly efficient synthetic strategy should prove to be of high use to synthesize a variety of similar and related enantiopure ABC-type structured TAGs constituting two unsaturated fatty acids, with one of them monounsaturated and the other polyunsaturated. It should be emphasized that PUFAs are not limited to linoleic acid and will most certainly suit bioactive n-6 and n-3 long-chain PUFAs, such as arachidonic acid, EPA, and DHA, which may be incorporated in the final step by the aid of chemical coupling, as has been demonstrated previously. Our continuing activities aim at modifying the current strategies to enable the synthesis of enantiostructured TAGs constituting two different types of PUFAs by investigating alternative protective groups.

## Figures and Tables

**Table 1:** Summary of yields and specific rotations of intermediates (*R*)-**9a–c** and (*R*)-**14**.

Compound	*sn*-1	*sn*-2	*sn*-3	Yield	[α]^20^_D_
(*R*)-**9a**	PMB	OH	12:0	>99%	−1.28
(*R*)-**9b**	PMB	OH	14:0	91%	−1.93
(*R*)-**9c**	PMB	OH	16:0	94%	−1.18
(*R*)-**14**	PMB	OH	18:1	87%	−0.72

**Table 2:** Summary of the yields and specific rotations of the intermediates (*R*)-**10a**–**c** and (*R*)-**15a**,**b**.

Compound	*sn*-1	*sn*-2	*sn*-3	Yield	[α]^20^_D_
(*R*)-**10a**	PMB	18:1	12:0	99%	−6.77
(*R*)-**10b**	PMB	18:1	14:0	99%	−6.61
(*R*)-**10c**	PMB	18:1	16:0	88%	−6.24
(*R*)-**10d**	PMB	18:2	12:0	88%	−6.05
(*R*)-**10e**	PMB	18:2	16:0	89%	−6.80
(*R*)-**15a**	PMB	14:0	18:1	94%	−6.86
(*R*)-**15b**	PMB	16:0	18:1	91%	−6.75

**Table 3:** Summary of yields and specific rotations of intermediates (*R*)-**11a**–**c** and (*R*)-**16a**,**b**.

Compound	*sn*-1	*sn*-2	*sn*-3	Yield	[α]^20^_D_
(*R*)-**11a**	OH	18:1	12:0	91%	+2.43
(*R*)-**11b**	OH	18:1	14:0	92%	+2.09
(*R*)-**11c**	OH	18:1	16:0	85%	+2.10
(*R*)-**16a**	OH	14:0	18:1	95%	+2.41
(*R*)-**16b**	OH	16:0	18:1	91%	+2.39

**Table 4:** Summary of yields and specific rotations of TAGs (*S*)-**1**, **2**, **5**, and **6** and (*R*)-**12a**–**d**, (*S*)-**12e**, and (*R*)-**12f**.

Compound	*sn*-1	*sn*-2	*sn*-3	Yield	[α]^20^_D_
(*S*)-**1**	18:2	18:1	12:0	85%	+0.03
(*S*)-**2**	18:2	18:1	16:0	92%	−0.01
(*S*)-**5**	18:2	14:0	18:1	94%	+0.05
(*S*)-**6**	18:2	16:0	18:1	95%	−0.02
(*R*)-**12a**	12:0	18:1	14:0	90%	+0.04
(*R*)-**12b**	10:0	18:1	16:0	98%	+0.05
(*R*)-**12c**	12:0	18:1	16:0	99%	+0.02
(*R*)-**12d**	14:0	18:1	16:0	96%	−0.02
(*S*)-**12e**	20:0	18:1	16:0	97%	+0.01
(*R*)-**12f**	12:0	18:2	16:0	86%	+0.09

**Table 5:** Summary of yields and specific optical rotations for all the intermediates and SUU’ subclass category TAGs (*R*)-**3** and (*R*)-**4** synthesized via the two-lipase-step method.

Compound	*sn*-1	*sn*-2	*sn*-3	Yield	[α]^20^_D_
(*S*)-**7**	isopropylidene		PMB	76%	+0.03
(*R*)-**8**	OH	OH	PMB	92%	−1.84
(*S*)-**14**	18:1	OH	PMB	74%	+0.79
(*S*)-**19**	18:1	OH	OH	70%	+1.05
(*S*)-**20a**	18:1	OH	12:0	83%	−1.28
(*S*)-**20b**	18:1	OH	16:0	87%	−0.76
(*S*)-**3**	18:1	18:2	12:0	97%	+0.08
(*S*)-**4**	18:1	18:2	16:0	96%	+0.09

## Data Availability

The data underlying this study are available in the published article and its online Supplementary Materials.

## Supplementary Materials

The following supporting information can be downloaded at https://www.mdpi.com/article/10.3390/molecules29071633/s1: Figure S1: Comparison of the glycerol regions of the PMB-protected solketal (*R*)-**7**, the PMB-protected glycerol (*S*)-**8**, and the PMB-protected *sn*-3-MAG (*R*)-**9c** (p*S1*); Figure S2: Comparison of the glycerol regions of the PMB-protected *sn*-3-MAG (*R*)-**9b** and the PMB-protected *sn*-2,3-DAG (*R*)-**10c** (p*S2*); Figure S3: Comparison of the glyceryl proton regions in the ^1^H NMR spectra for the diacylglycerol (*R*)-**16a** and the triacylglycerol (*S*)-**5** (p*S3*); Figure S4: Comparison of the glycerol proton regions in the ^1^H NMR spectra for the 1-MAG (S)-**19**, 1,3-DAG (S)-**20b**, 1,2-DAG (R)-**15a**, and TAG (S)-**5** (p*S4*); Experimental Information: p*S5*-*S20*; NMR spectra: p*S21*-*S106*.
