# Supplementary material for: Chemoenzymatic Synthesis of ABC-Type Enantiostructured Triacylglycerols by the Use of the p-Methoxybenzyl Protective Group
Source: Molecules. 2024 Apr 5;29(7):1633. doi: 10.3390/molecules29071633 (PMC11013301; doi:10.3390/molecules29071633)

Supplementary Materials

Chemoenzymatic synthesis of ABC-type enantiostructured triacylglycerols by the use of the *p*-methoxybenzyl protective group

Hafdis Haraldsdottir[1], Haraldur G. Gudmundsson[1], Kaisa M. Linderborg[2], Baoru Yang[2] and Gudmundur G. Haraldsson[1]*

[1)] Science Institute, Chemistry Department, University of Iceland, Dunhaga 3, 107 Reykjavik, Iceland

[2)] Food Sciences, Department of Life Technologies, University of Turku, Turku, Finland

Table of Contents

**Figures** ***S1 – S4***	*S1-S4*
**Experimental Information**	*S5-S20*
**NMR Spectra**	
Compound (*R*)-**7**	*S21*
Compound (*S*)-**8**	*S24*
Compound (*R*)-**9a**	*S27*
Compound (*R*)-**9b**	*S30*
Compound (*R*)-**9c**	*S32*
Compound (*R*)-**10a**	*S34*
Compound (*R*)-**10b**	*S37*
Compound (*R*)-**10c**	*S39*
Compound (*R*)-**10d**	*S41*

Compound (*R*)-**10e** *S43*

Compound (*R*)-**11a** *S45*

Compound (*R*)-**11b** *S47*

Compound (*R*)-**11c** *S49*

Compound (*R*)-**11e** *S51*

Compound (S)-**1** *S53*

Compound (S)-**2** *S55*

Compound (*R*)-**12a** *S57*

Compound (*R*)-**12b** *S59*

Compound (*R*)-**12c** *S61*

Compound (*R*)-**12d** *S63*

Compound (*R*)-**12e** *S65*

Compound (*R*)-**12f** *S67*

Compound **13** *S69*

Compound (*R*)-**14** *S71*

Compound (*R*)-**15a** *S73*

Compound (*R*)-**15b** *S75*

Compound (*R*)-**16a** *S77*

Compound (*R*)-**16b** *S79*

Compound (S)-**5** *S81*

Compound (S)-**6** *S83*

Compound (*R*)-**17** *S85*

Compound (*R*)-**18** *S87*

Compound (*S*)-**7** *S90*

Compound (*R*)-**8** *S92*

Compound (S)-**14** *S94*

Compound (S)-**19** *S96*

Compound (S)-**20a** *S98*

Compound (S)-**20b** *S101*

Compound (S)-**3** *S103*
Compound (S)-**4** *S105*

Figures *S1* – *S4*

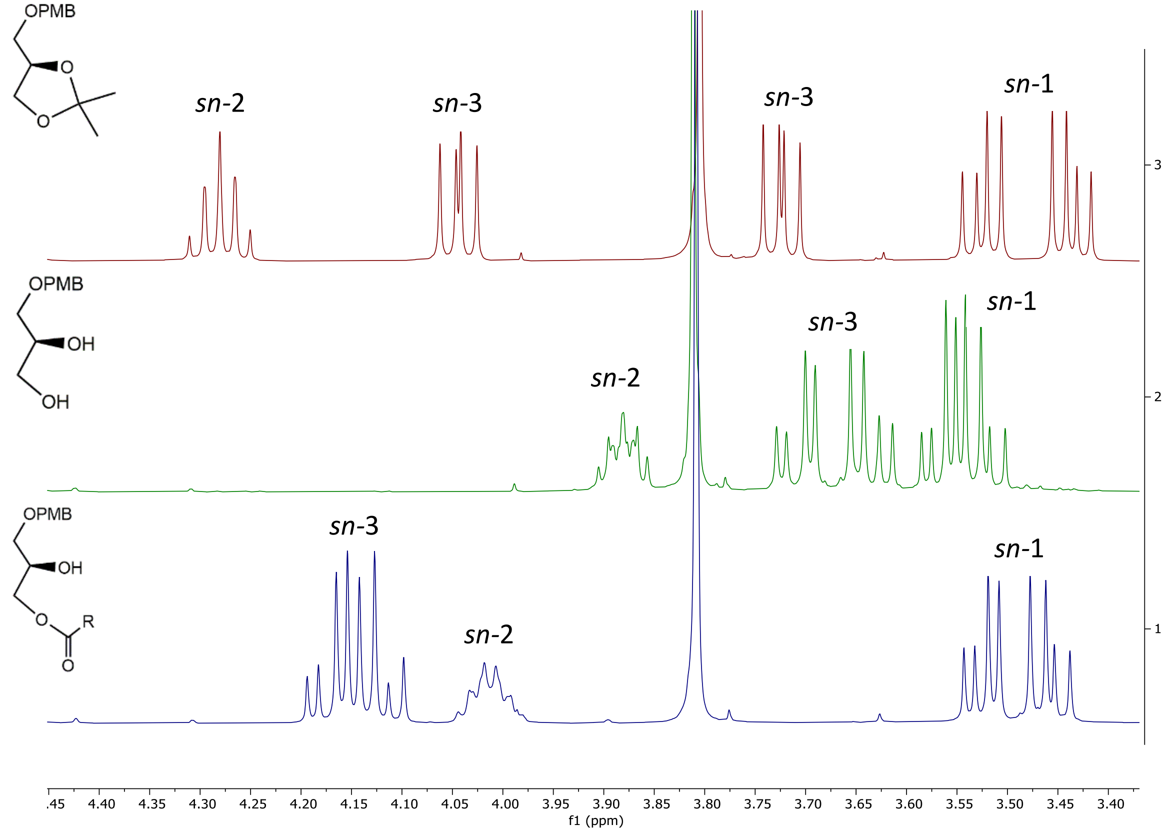

Figure *S1*. Comparison of the glycerol proton region of the ^{1}H NMR spectra for the PMB-protected solketal (*R*)-**7** (red), the PMB-protected glycerol (*S*)-**8** (green) and the PMB-protected monoacylglycerol (*R*)-**9c** (blue).

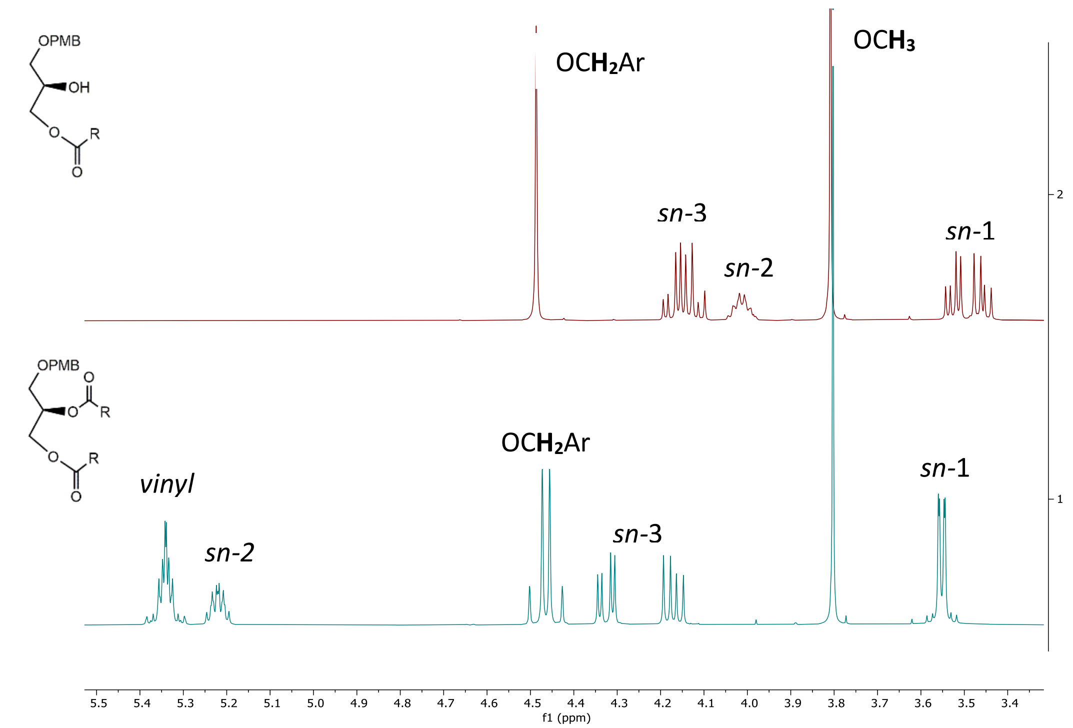

Figure *S*2. Comparison of the glycerol proton region of the ^{1}H NMR spectra for PMB-protected compounds (*R*)-**9b** (top) and (*R*)-**10c** (bottom).

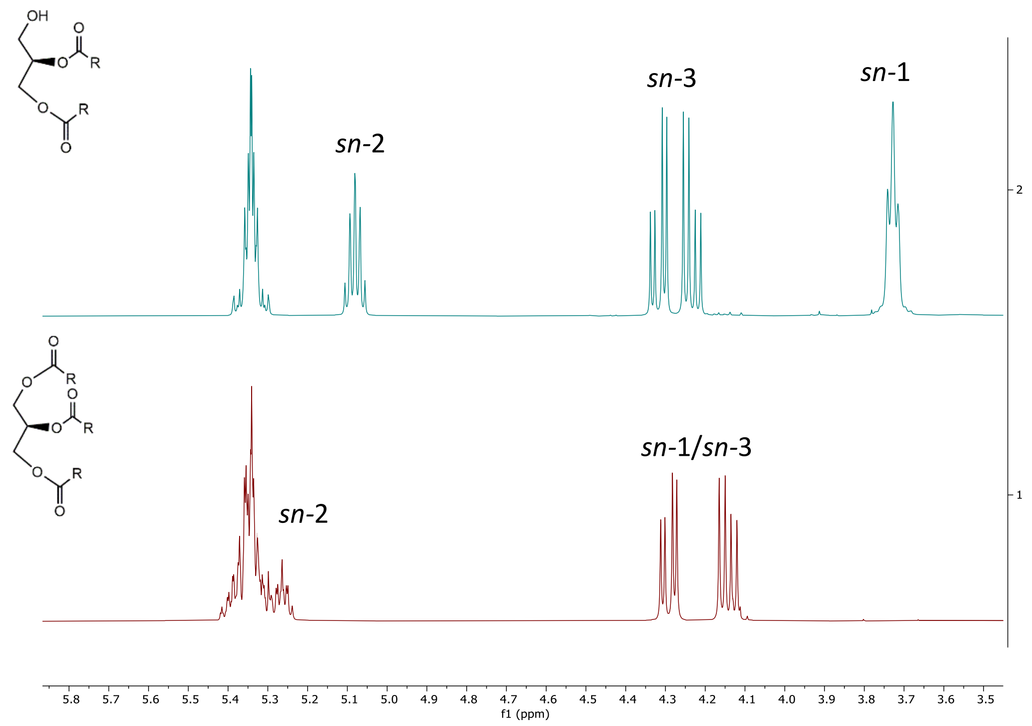

Figure *S*3. Comparison of the glycerol proton region of the ^{1}H NMR spectra for the diacylglycerol (*R*)-**15a** (top) and the triacylglycerol (*S*)-**5** (bottom).

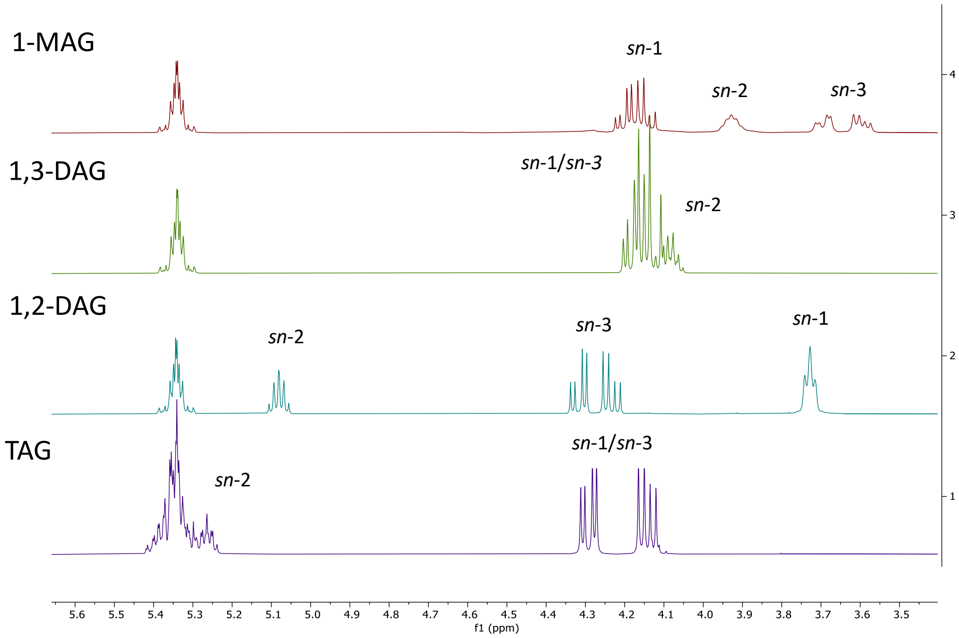

Figure *S4*. Comparison of the glycerol proton region of the ^{1}H NMR spectra for the 1-MAG (*S*)-**19**, 1,3-DAG (*S*)-**20b**, 1,2-DAG (*R*)-**15a** and TAG (*S*)-**5**.

Experimental Information

3.1 General Information

The ^{1}H- and ^{13}C-NMR spectra were recorded on a 400 MHz Bruker Avance NEO 400 spectrometer (Bruker Switzerland AG, Faellanden, Switzerland). Chemical shifts (δ) are reported in parts per million (ppm) from tetramethylsilane with the solvent resonance used as an internal standard. In all cases the solvent was deuterochloroform which had been filtered through aluminium oxide to get rid of acid contamination. The coupling constants (J) are given in Hertz (Hz). The following abbreviations are used to describe the multiplicity: s, singlet; d, doublet; t, triplet; q, quartet; p, pentet; dd, doublet of doublets; dt, doublet of triplets; ABq, AB-quartet; m, multiplet. For ^{13}C-NMR, the number of carbon nuclei contributing to each signal is indicated in parentheses after the chemical shift value. Infrared spectra were recorded on a Nicolet IS 10 FT-IR spectrometer (Thermo Scientific, Madison, Wisconsin, USA) using either sodium chloride windows (NaCl) for liquid compounds, in potassium bromide pellets (KBr) for solids or a diamond ATR crystal that was used for both liquids and solids. The following abbreviations are used to describe the peaks: s, strong; vs, very strong; m, medium; w, weak; br, broad. The high-resolution mass spectra (HMRS) were recorded on a Bruker OTOF-Q Compact ESI mass spectrometer (Bruker Daltonic, Bremen, Germany). Optical activities were measured on an Autopol V automatic Polarimeter from Rudolph Research Analytical (Hacketstown, New Jersey, USA) using a 40T-2.5-100-0.7 Temp Trol polarimetric cell with 2.5 mm inside diameter, 100 mm optical length and 0.7 mL volume with c (concentration) referring to g sample/100mL. Melting points were determined using a Büchi m-560 melting point apparatus. TLC monitoring was done on silica plates from SiliCycle and the plates were developed in 4% PMA solution in methanol. Boric acid impregnated silica gel was prepared by dissolving 4g of boric acid in 100 mL methanol then adding 55g of silica and swirling the resulting slurry for a few minutes. The methanol was then evaporated off and the silica dried in vacuo for 6h at 40°C.

All chemicals and solvents were used without further purification unless otherwise stated. Most solvents used, deuterated chloroform (99.8% D), diethyl ether (99.8%), chloroform (≥99.5%), dichloromethane (99.8%), ethanol (≥99.8%), hexane (>97%), methanol (99.9%) and tetrahydrofuran (99.9%), were from Sigma-Aldrich (Steinheim, Germany). Tetrahydrofuran and dichloromethane were dried over molecular sieves and stored under nitrogen. Ethyl acetate and petroleum ether (special boiling point 60-95°C) were purchased from from Brenntag (Essen, Germany) in barrels. The petroleum ether was purified by distillation on rotary evaporator. All the following chemicals: p-anisaldehyde (98%), boric acid (≥99.5%), hydrochloric acid (37%), magnesium sulfate (≥99.5%), phosphomolybdic acid, sodium bicarbonate (≥99.0%), sodium hydride (60% dispersion in mineral oil), sodium sulfate (≥99%), sodium thiosulfate (≥98.5%), (R)-solketal (98%, 98% ee), (S)-solketal (98%, 99% ee), stearic acid (≥99%), triphenylphosphine (99%), vinyl caprate (>95%) and vinyl laurate (≥99%) were obtained from Sigma-Aldrich. Capric acid (>99%), lauric acid (>99.5%), myristic acid (>99.5%), oleic alcohol and tetrabutylammonium bromide (99%) were from Acros Organics (Geel, Belgium). 2,3-Dichloro-5,6-dicyano-1,4-benzoquinone (>97%), 1-Ethyl-3-(3-dimethylamino propyl) carbodiimide (>98%), p-methoxybenzyl chloride (>98%), vinyl myristate (>99%), vinyl palmitate (>96%) and vinyl stearate (>95%) were purchased from TCI Europe (Zwinderecht, Belgium). Arachidic acid, linoleic acid, oleic acid and palmitic acid were all from Larodan Fine Chemicals (Malmö, Sweden). Acetoxime (98%), 4-Dimethylaminopyridine (≥99%), elemental iodine, potassium hydroxide and sodium chloride (≥99.8%) were obtained from Merck (Darmstadt, Germany). The immobilized *Candida antarctica* lipase B (CAL-B, Novozym 435) was obtained as a gift from Novozymes A/S (Bagsvaerd, Denmark).

*3.2. Synthesis of the SUU′ subclass category TAGs (S)-**1** and **2***

3.2.1. Synthesis of 2,3-*O*-isopropylidene-1-*O*-(*p*-methoxybenzyl)-*sn*-glycerol, (*R*)-**7**

Sodium hydride (60% mineral oil dispersion, 490mg, 20.43 mmol) was added to a 250 mL flame-dried two-necked round bottom flask with a magnetic stirrer and rinsed three times with dry THF (10 mL portions) under nitrogen atmosphere. After that, a fresh portion of dry THF (15 mL) was added and the solution cooled to 0°C. (*R*)-Solketal (900mg, 6.81 mmol) was added dropwise to the solution in dry THF, the mixture was then allowed to reach room temperature and stirred for 1.5 h. After that time, the solution was again cooled to 0°C and *p*-methoxybenzyl chloride (1226mg, 7.83 mmol) was added. Finally, the solution was refluxed at for 22h after which the mixture had a deep orange colour. The reaction was carefully quenched with water and extracted three times with dichloromethane. The combined organic extracts were washed with water and brine, then dried over $MgSO_4$ and concentrated *in vacuo.* The crude concentrate was then purified by flash column chromatography using ethyl acetate:petroleum ether (2:8) as eluent affording the product (*R*)-**7** as a slightly yellow liquid (1303mg, 76% yield). TLC (Silica, ethyl acetate:petroleum ether, 2:8): Rf = 0.39. $[\alpha]^{20}_D$ = -1.24 (c. 2.16, CH_2Cl_2). IR (NaCl, ν_{max} / cm^{-1}): 2935 (s), 2865 (s), 1613 (m), 1248 (vs), 1037 (s). ^1H NMR (400 MHz, $CDCl_3$) δ_H: 7.26 (d, *J*=8.8 Hz, 2H, Ph-H), 6.88 (d, *J*=8.8 Hz, 2H, Ph-H), 4.49 (m, 2H, $PhCH_2$), 4.27 (m, 1H, CH *sn*-2), 4.04 (dd, *J*=8.3, 6.4 Hz, 1H, CH_2 *sn*-3), 3.80 (s, 3H, OCH_3), 3.72 (dd, *J*=8.3, 6.3 Hz, 1H, CH_2 *sn*-3), 3.52 (dd, *J*=9.8, 5.7 Hz, 1H, CH_2 *sn*-1), 3.44 (dd, *J*=9.8, 5.6 Hz, 1H, CH_2 *sn*-1), 1.42 (s, 3H, $C(CH_3)_2$), 1.36 (s, 3H, $C(CH_3)_2$) ppm. $^{13}C\{H\}$ NMR (101 MHz, $CDCl_3$) δ_C: 159.3, 130.0, 129.4 (2), 113.8 (2), 109.4, 74.7, 73.2, 70.8, 66.9, 55.3, 26.8, 25.4 ppm. HRMS (ESI) *m*/*z*: $[M + Na]^+$ calcd for $C_{14}H_{20}O_4Na$ 275.1254; found, 275.1255.

3.2.2. Synthesis of 1-*O*-(*p*-methoxybenzyl)-*sn*-glycerol, (*S*)-**8**

PMB-solketal (*R*)-**7** (1300mg, 5.15 mmol) in acetonitrile (25 mL) was added to a 50 mL round bottom flask equipped with a magnetic stirrer. Subsequently, elemental iodine (392mg, 1.54 mmol) and water (1 mL) were added to the solution, and it allowed to stir for 22h at room temperature under nitrogen atmosphere. After that time, the solution was quenched with 50 mL $Na_2S_2O_3$ (20% w/w aqueous solution) and extracted three times with ethyl acetate. The combined organic layers were dried over Na_2SO_4 and concentrated *in vacuo.* The crude concentrate was then purified by flash column chromatography, first using ethyl acetate/petroleum ether (3:7) as eluent, and then gradually increasing the proportion of ethyl acetate until the eluent was pure ethyl acetate. That afforded the product (*S*)-**8**, which solidified upon drying under vacuum into a slightly yellow solid. It was then recrystallized in hexane which afforded colourless fine needles (1092mg, 97% yield). TLC (Silica, ethyl acetate:petroleum ether, 30:70): Rf = 0.11. Mp. 43.1-43.6°C. $[\alpha]^{20}_D$ = +2.48 (c. 1.73, CH_2Cl_2). IR (NaCl, ν_{max} / cm^{-1}): 3389 (br), 2935 (s), 2837 (vs), 1612 (m), 1463 (m), 1247 (s), 1033 (vs). ^1H NMR (400 MHz, $CDCl_3$) δ_H: 7.25 (d, *J*=8.7 Hz, 2H, Ph-H), 6.89 (d, *J*=8.7 Hz, 2H, Ph-H), 4.49 (s, 2H, $PhCH_2$), 4.88 (m, 1H, CH *sn*-2), 3.81 (s, 3H, OCH_3), 3.71 (dd, *J*=11.5, 3.8 Hz, 1H, CH_2 *sn*-3), 3.63 (dd, *J*=11.5, 5.6 Hz, 1H, CH_2 *sn*-3), 3.57 (dd, *J*=9.6, 3.6 Hz, 1H, CH_2 *sn*-1), 3.52 (dd, *J*=9.6, 3.5 Hz, 1H, CH_2 *sn*-1), 2.59 (br s, 1H, OH), 2.10 (br s, 1H, OH) ppm. $^{13}C\{H\}$ NMR (101 MHz, $CDCl_3$) δ_C: 159.4, 129.8, 129.5 (2), 113.9 (2), 73.4, 71.5, 70. 6, 63.9, 55.3 ppm. HRMS (ESI) *m*/*z*: $[M + Na]^+$ calcd for $C_{11}H_{16}O_4Na$ 235.0941; found, 235.0943.

3.2.3. Synthesis of 3-dodecanoyl-1-*O*-(*p*-methoxybenzyl)-*sn*-glycerol, (*R*)-**9a**

PMB-protected glycerol (*S*)-**8** (130mg, 0.55 mmol) and vinyl laurate (160mg, 0.71 mmol) dissolved in dichloromethane (3 mL) were added under slow magnetic stirring to a 10 mL round bottom flask. Subsequently, an immobilized *Candida antarctica* lipase (CAL-B) (28mg, 10% w/w) was added, and the atmosphere replaced with nitrogen gas. The mixture was allowed to stir for 4h while being monitored by

TLC. After that time, the reaction was complete, and the lipase was filtered off. The solvent was removed *in vacuo,* the crude concentrate washed with a 15mg/mL $NaHCO_3$ / methanol (1:1) solution and then extracted twice with hexane. The combined organic extracts were dried over Na_2SO_4 and concentrated *in vacuo.* The crude product was then purified by flash column chromatography with 4% boric acid impregnated silica gel using ethyl acetate:hexane (2:8) as eluent affording the product (I)-**9a** as a colourless liquid (231mg, quantitative yield). TLC (Silica, ethyl acetate:petroleum ether, 20:80): Rf = 0.27. $[\alpha]^{20}_D$ = -1.28 (c. 2.50, CH_2Cl_2). IR (NaCl, ν_{max} / cm^{-1}): 3449 (br), 2925 (s), 2854 (vs), 1736 (vs), 1612 (m), 1466 (m), 1377 (m), 1248 (s), 1037 (m). ^1H NMR (400 MHz, $CDCl_3$) δ_H: 7.25 (d, *J*=9.0 Hz, 2H, Ph-H), 6.88 (d, *J*=8.7 Hz, 2H, Ph-H), 4.49 (s, 2H, $PhCH_2$), 4.17 (dd, *J*=11.5, 4.4 Hz, 1H, CH_2 *sn*-3), 4.12 (dd, *J*=11.5, 6.0 Hz, 1H, CH_2 *sn*-3), 4.01 (m, 1H, CH *sn*-2), 3.81 (s, 3H, OCH_3), 3.52 (dd, *J*=9.6, 4.3 Hz, 1H, CH_2 *sn*-1), 3.46 (dd, *J*=9.6, 6.2 Hz, 1H, CH_2 *sn*-1), 2.49 (br s, 1H, OH), 2.32 (t, *J*=7.6 Hz, 2H, CH_2COO), 1.61 (m, 2H, CH_2CH_2COO), 1.29-1.24 (m, 16H, CH_2), 0.88 (t, *J*=6.8 Hz, 3H, CH_2CH_3) ppm. $^{13}C\{H\}$ NMR (101 MHz, $CDCl_3$) δ_C: 174.1, 159.5, 129.9, 129.6 (2), 114.0 (2), 73.3, 70.7, 69.1, 65.5, 55.4, 34.3, 32.1, 29.7, 29.6, 29.45, 29.4 (2), 29.3, 25.1, 22.8, 14.3 ppm. HRMS (ESI) *m*/*z*: $[M + Na]^+$ calcd for $C_{23}H_{38}O_5Na$ 417.2611; found, 417.2610.

3.2.4. Synthesis of 1-*O*-(*p*-methoxybenzyl)-3-tetradecanoyl-*sn*-glycerol, (*R*)-**9b**

The same procedure was followed as described for (*R*)-**9a** using the diol (*S*)-**8** (100mg, 0.43mmol), vinyl myristate (132mg, 0.52 mmol) and immobilized *Candida antarctica* lipase (CAL-B) (23mg, 10% w/w) in dichloromethane (3 mL). The crude product was then purified by flash column chromatography with 4% boric acid impregnated silica gel using ethyl acetate/hexane (2:8) as eluent. When drying the product *in vacuo*, it became a white solid. It was then recrystallized in hexane which afforded the product (*R*)-**9b** as a white lightweight powder (181mg, 91% yield). TLC (Silica, ethyl acetate:petroleum ether, 20:80): Rf = 0.49. Mp. 30.0-30.9°C. $[\alpha]^{20}_D$ = -1.93 (c. 1.97, CH_2Cl_2). IR (NaCl, ν_{max} / cm^{-1}): 3460 (br), 2924 (s), 2854 (vs), 1739 (vs), 1613 (m), 1466 (m), 1249 (s), 1038 (vs). ^1H NMR (400 MHz, $CDCl_3$) δ_H: 7.25 (d, *J*=8.9 Hz, 2H, Ph-H), 6.88 (d, *J*=8.8 Hz, 2H, Ph-H), 4.49 (s, 2H, $PhCH_2$), 4.17 (dd, *J*=11.5, 4.4 Hz, 1H, CH_2 *sn*-3), 4.12 (dd, *J*=11.5, 6.1 Hz, 1H, CH_2 *sn*-3), 4.01 (m, 1H, CH *sn*-2), 3.81 (s, 3H, OCH_3), 3.52 (dd, *J*=9.6, 4.3 Hz, 1H, CH_2 *sn*-1), 3.46 (dd, *J*=9.6, 6.2 Hz, 1H, CH_2 *sn*-1), 2.47 (br s, 1H, OH), 2.32 (t, *J*=7.6 Hz, 2H, CH_2COO), 1.61 (m, 2H, CH_2CH_2COO), 1.32-1.25 (m, 20H, CH_2), 0.88 (t, *J*=6.8 Hz, 3H, CH_2CH_3) ppm. $^{13}C\{H\}$ NMR (101 MHz, $CDCl_3$) δ_C: 174.1, 159.5, 129.9, 129.6 (2), 114.0 (2), 73.3, 70.7, 69.1, 65.5, 55.4, 34.3, 32.1, 29.82, 29.79 (2), 29.75, 29.6, 29.5, 29.4, 29.3, 25.1, 22.83, 14.3 ppm. HRMS (ESI) *m*/*z*: $[M + Na]^+$ calcd for $C_{25}H_{42}O_5Na$ 445.2924; found, 445.2919.

3.2.5 Synthesis of 3-hexadecanoyl-1-*O*-(*p*-methoxybenzyl)-*sn*-glycerol, (*R*)-**9c**

The same procedure was followed as described for (*R*)-**9a** using diol (*S*)-**8** (132mg, 0.56 mmol), vinyl palmitate (200mg, 0.71 mmol) and immobilized *Candida antarctica* lipase B (CAL-B) (30mg, 10% w/w) in dichloromethane (3 mL). The crude product was then purified by flash column chromatography with 4% boric acid impregnated silica gel using ethyl acetate/hexane (2:8) as eluent. When drying the product *in vacuo*, it became a white solid. It was then recrystallized in hexane which afforded the product (R)-**9c** as a white lightweight powder (250mg, 94% yield). TLC (Silica, ethyl acetate:petroleum ether, 30:70): Rf = 0.38. Mp. 40.3-41.3°C. $[\alpha]^{20}_D$ = -1.18 (c. 1.44, CH_2Cl_2). IR (NaCl, ν_{max} / cm^{-1}): 3242 (br), 2919 (s), 2851 (vs), 1738 (vs), 1614 (m), 1467 (m), 1249 (s), 1036 (vs). ^1H NMR (400 MHz, $CDCl_3$) δ_H: 7.25 (d, *J*=8.6 Hz, 2H, Ph-H), 6.88 (d, *J*=8.6 Hz, 2H, Ph-H), 4.49 (s, 2H, $PhCH_2$), 4.17 (dd, *J*=11.5, 4.4 Hz, 1H, CH_2 *sn*-3), 4.12 (dd, *J*=11.5, 6.0 Hz, 1H, CH_2 *sn*-3), 4.01 (m, 1H, CH *sn*-2), 3.81 (s, 3H, OCH_3), 3.53 (dd, *J*=9.6, 4.3 Hz, 1H, CH_2 *sn*-1), 3.46 (dd, *J*=9.6, 6.1 Hz, 1H, CH_2 *sn*-1), 2.46 (d, *J*=4.8, 1H, OH), 2.32 (t, *J*=7.6 Hz, 2H, CH_2COO), 1.61 (p, *J*=7.1 Hz, 2H, CH_2CH_2COO), 1.34-1.22 (m, 24H, CH_2), 0.88 (t, *J*=6.8 Hz, 3H, CH_2CH_3) ppm. $^{13}C\{H\}$ NMR (101 MHz, $CDCl_3$) δ_C: 173.8, 159.228, 129.6, 129.3 (2), 113.7 (2), 73.01, 70.4, 68.8, 65.2, 55.1, 34.0, 31.8, 29.53, 29.49, 29.4, 29.3, 29.2, 29.1, 29.0, 24.8, 22.5, 14.0 ppm. HRMS (ESI) *m*/*z*: $[M + Na]^+$ calcd for $C_{27}H_{46}O_5Na$ 473.3243; found, 473.3222.

3.2.6. Synthesis of 3-dodecanoyl-1-*O*-(*p*-methoxybenzyl)-2-[(9*Z*)-octadec-9-enoyl)]-*sn*-glycerol, (*R*)-**10a**

Monoacylglycerol (*R*)-**9a** (200mg, 0.51 mmol) and oleic acid (165mg, 0.58 mmol) in dry dichloromethane (5mL) were added to a flame-dried 10 mL round bottom flask equipped with a magnetic stirrer. Then 4-dimethylaminopyridine (DMAP) (50 mg, 0.41 mmol) and 1-ethyl-3-(3-dimethylaminopropyl)carbodiimide (EDCI) (117mg, 0.61 mmol) were added to the solution and stirred at room temperature, under nitrogen for 20h. After that time, the reaction mixture was passed through a short column packed with silica gel by using ethyl acetate. The solvent was removed *in vacuo,* and the crude concentrate was then purified by flash column chromatography using ethyl acetate/hexane (1:9) as eluent affording the product (*R*)-**10a** as a colourless liquid (188mg, 99% yield). TLC (Silica, ethyl acetate:petroleum ether, 20:80): Rf = 0.60. $[\alpha]^{20}_{D}$ = -6.77 (c. 1.92, CH_2Cl_2). IR (NaCl, ν_{max} / cm^{-1}): 3003 (m) ,2925 (s), 2854 (s), 1743 (s), 1613 (m), 1464 (m), 1248 (s), 1172 (s), 1038 (m). ^1H NMR (400 MHz, $CDCl_3$) δ_H: 7.23 (d, *J*=8.8 Hz, 2H, Ph-H), 6.87 (d, *J*=8.8 Hz, 2H, Ph-H), 5.34 (m, 2H, =CH), 5.23 (m, 1H, CH *sn*-2), 4.45 (ABq, $\Delta\delta_{AB}$=0.04, *J*=11.8, 2H, $PhCH_2$), 4.33 (dd, *J*=11.9, 3.9 Hz, 1H, CH_2 *sn*-3), 4.17 (dd, *J*=11.9, 6.5 Hz, 1H, CH_2 *sn*-3), 3.80 (s, 3H, OCH_3), 3.55 (dd, *J*=5.2, 1.3 Hz, 2H, CH_2 *sn*-1), 2.31 (t, *J*=7.6 Hz, 2H, CH_2COO SFA), 2.27 (t, *J*=7.6 Hz, 2H, CH_2COO MUFA), 2.01 (m, 4H, C*H*$_2$CH=), 1.65-1.56 (m, 4H, C*H*$_2$$CH_2COO$), 1.36-1.22 (m, 36H, CH_2), 0.88 (t, *J*=6.7 Hz, 6H, CH_2C*H*$_3$) ppm. $^{13}C\{H\}$ NMR (101 MHz, $CDCl_3$) δ_C: 173.6, 173.2, 159.5, 130.2, 129.92, 129.87, 129.5 (2), 114.0 (2), 73.1, 70.2, 68.1, 62.8, 55.4, 34.5, 34.3, 32.1 (2), 29.92, 29.87, 29.8, 29.7, 29.6 (2), 29.49 (2), 29.47 (2), 29.44, 29.36, 29.3 (2), 29.2, 27.4, 27.3, 25.1, 25.0, 22.8, 14.3 (2) ppm. $[M + Na]^+$ calcd for $C_{41}H_{70}O_6Na$ 681.5065; found, 681.5050.

3.2.7. Synthesis of 1-*O*-(*p*-methoxybenzyl)-2-[(9*Z*)-octadec-9-enoyl)]-3-tetradecanoyl-*sn*-glycerol, (*R*)-**10b**

The same procedure was followed as described for (*R*)-**10a**, using (*R*)-**9b** (170mg, 0.40 mmol), oleic acid (131mg, 0.46 mmol), DMAP (39mg, 0.32 mmol) and EDCI (93mg, 0.48 mmol) in dry dichloromethane (4mL). The crude product was purified by flash column chromatography using ethyl acetate/hexane (1:9) as eluent affording the product (*R*)-**10b** as a colourless liquid (274mg, 99% yield). TLC (Silica, ethyl acetate:petroleum ether, 20:80): Rf = 0.56. $[\alpha]^{20}_{D}$ = -6.61 (c. 1.68, CH_2Cl_2). IR (NaCl, ν_{max} / cm^{-1}): 3003 (m), 2925 (s), 2854 (s), 1742 (s), 1613 (w), 1465 (m), 1248 (s), 1172(s), 1038 (vs). ^1H NMR (400 MHz, $CDCl_3$) δ_H: 7.23 (d, *J*=8.6 Hz, 2H, Ph-H), 6.87 (d, *J*=8.8 Hz, 2H, Ph-H), 5.34 (m, 2H, =CH), 5.22 (m, 1H, CH *sn*-2), 4.46 (ABq, $\Delta\delta_{AB}$=0.04, *J*=11.8, 2H, $PhCH_2$), 4.33 (dd, *J*=11.9, 3.8 Hz, 1H, CH_2 *sn*-3), 4.17 (dd, *J*=11.9, 6.4 Hz, 1H, CH_2 *sn*-3), 3.80 (s, 3H, OCH_3), 3.55 (dd, *J*=5.2, 1.3 Hz, 2H, CH_2 *sn*-1), 2.31 (t, *J*=7.5 Hz, 2H, CH_2COO SFA), 2.27 (t, *J*=7.7 Hz, 2H, CH_2COO MUFA), 2.00 (m, 4H, C*H*$_2$CH=), 1.66-1.57 (m, 4H, C*H*$_2$$CH_2COO$), 1.34–1.21 (m, 40H, CH_2), 0.88 (t, *J*=6.9 Hz, 6H, CH_2C*H*$_3$) ppm. $^{13}C\{H\}$ NMR (101 MHz, $CDCl_3$) δ_C: 173.6, 173.3, 159.5, 130.2, 129.91, 129.86, 129.5 (2), 114.0 (2), 73.1, 70.2, 68.1, 62.8, 55.4, 34.5, 34.3, 32.07, 32.05, 29.92, 29.87, 29.84, 29.81 (2), 29.78, 29.7, 29.6 (2), 29.51, 29.47 (2), 29.44, 29.35, 29.3 (2), 29.2, 27.4, 27.3, 25.1, 25.0, 22.8, 14.3 (2) ppm. $[M + Na]^+$ calcd for $C_{43}H_{74}O_6Na$ 709.5378; found, 709.5390.

3.2.8. Synthesis of 3-hexadecanoyl-1-*O*-(*p*-methoxybenzyl)-2-[(9*Z*)-octadec-9-enoyl)]-*sn*-glycerol, (*R*)-**10c**

The same procedure was followed as described for (*R*)-**10a**, using (*R*)-**9c** (135mg, 0.30 mmol), oleic acid (97mg, 0.34 mmol), DMAP (29mg, 0.24 mmol) and EDCI (69mg, 0.36 mmol) in dry dichloromethane (3mL). The crude product was purified by flash column chromatography using ethyl acetate:hexane (1:9) as eluent affording the product (*R*)-**10c** as a colourless liquid (188mg, 88% yield). TLC (Silica, ethyl acetate:petroleum ether, 20:80): Rf = 0.55. $[\alpha]^{20}_{D}$ = -6.24 (c. 1.41, CH_2Cl_2). IR (NaCl, ν_{max} / cm^{-1}): 2925 (s), 2854 (s), 1742 (s), 1613 (w), 1466 (m), 1248 (s), 1172(s), 1038 (vs). ^1H NMR (400 MHz, $CDCl_3$) δ_H: 7.23 (d, *J*=8.8 Hz, 2H, Ph-H), 6.87 (d, *J*=8.8 Hz, 2H, Ph-H), 5.34 (m, 2H, =CH), 5.22 (m, 1H, CH *sn*-2), 4.46 (ABq, $\Delta\delta_{AB}$=0.04, *J*=11.7, 2H, $PhCH_2$), 4.33 (dd, *J*=11.9, 3.8 Hz, 1H, CH_2 *sn*-3), 4.17 (dd, *J*=11.9, 6.4 Hz, 1H, CH_2 *sn*-3), 3.80 (s, 3H, OCH_3), 3.55 (dd, *J*=5.2, 1.0 Hz, 2H, CH_2 *sn*-1), 2.31 (t, *J*=7.5 Hz, 2H, CH_2COO SFA), 2.27 (t, *J*=7.5 Hz, 2H, CH_2COO MUFA), 2.01 (q, *J*=6.4 Hz, 4H, C*H*$_2$CH=), 1.63-1.55 (m, 4H, C*H*$_2$$CH_2COO$), 1.38-1.20 (m, 44H, CH_2), 0.88 (t, *J*=6.8 Hz, 6H, CH_2C*H*$_3$) ppm. $^{13}C\{H\}$ NMR (101 MHz, $CDCl_3$) δ_C: 173.6, 173.2, 159.5, 130.2, 129.91, 129.86, 129.4 (2), 114.0 (2), 73.1, 70.2, 68.0, 62.8, 55.4 34.5, 34.3, 32.07, 32.05, 32.0, 29.91, 29.87, 29.84 (2), 29.81 (2), 29.78, 29.7,

29.6, 29.51, 29.47 (2), 29.44, 29.35, 29.3 (2), 29.2, 29.1, 27.4, 27.3, 25.1, 25.0, 22.8, 14.4, 14.3 ppm. $[M + Na]^+$ calcd for $C_{45}H_{78}O_6Na$ 737.5696; found, 737.5689.

3.2.9. Synthesis of 3-dodecanoyl-1-*O*-(*p*-methoxybenzyl)-2-[(9Z,12Z)-octadeca-9,12-dienoyl)]-*sn*-glycerol, (*R*)-**10d**

The same procedure was followed as described for (*R*)-**10a**, using ***(R)*-9a** (300mg, 0.76 mmol), linoleic acid (245mg, 0.87 mmol), DMAP (74mg, 0.61 mmol) and EDCI (175mg, 0.91 mmol) in dry dichloromethane (4mL). The crude product was purified by flash column chromatography using ethyl acetate/hexane (1:9) as eluent affording the product (*R*)-**10d** as a colourless liquid (441mg, 88% yield). TLC (Silica, ethyl acetate:petroleum ether, 20:80): Rf = 0.59. $[\alpha]^{20}_D$ = -6.05 (c. 1.72, CH_2Cl_2). IR (ATR, ν_{max} / cm^{-1}): 3008 (m) ,2926 (s), 2855 (s), 1747 (s), 1464 (m), 1161 (s), 1038 (m). ^1H NMR (400 MHz, $CDCl_3$) δ_H: 7.23 (d, *J*=8.8 Hz, 2H, Ph-H), 6.87 (d, *J*=8.8 Hz, 2H, Ph-H), 5.35 (m, 4H, =CH), 5.22 (m, 1H, CH *sn*-2), 4.45 (ABq, $\Delta\delta_{AB}$=0.04, *J*=11.7, 2H, $PhCH_2$), 4.33 (dd, *J*=11.9, 3.8 Hz, 1H, CH_2 *sn*-3), 4.17 (dd, *J*=11.9, 6.4 Hz, 1H, CH_2 *sn*-3), 3.80 (s, 3H, OCH_3), 3.55 (dd, *J*=5.2, 1.3 Hz, 2H, CH_2 *sn*-1), 2.77 (t, *J*=6.1, 2H, =CHCH_2CH=), 2.31 (t, *J*=7.6 Hz, 2H, CH_2COO SFA), 2.27 (t, *J*=7.6 Hz, 2H, CH_2COO PUFA), 2.04 (q, *J*=6.4, 4H, CH_2CH=), 1.65-1.58 (m, 4H, CH_2CH_2COO), 1.38-1.23 (m, 30H, CH_2), 0.89 (t, *J*=6.9, 3H, CH_2CH_3 PUFA), 0.88 (t, *J*=6.8, 6H, CH_2CH_3 SFA) ppm. $^{13}C\{H\}$ NMR (101 MHz, $CDCl_3$) δ_C: 173.6, 173.2, 159.5, 130.4, 130.2, 129.4 (2), 128.2, 128.0, 114.0 (2), 73.1, 70.2, 68.0, 62.8, 55.4, 34.5, 34.3, 32.1, 31.7, 29.8 (4), 29.6, 29.5 (2), 29.43, 29.35, 29.3 (2), 29.2, 27.4 (2), 25.8, 25.1, 25.0, 22.8, 22.7, 14.3, 14.2 ppm. $[M + Na]^+$ calcd for $C_{41}H_{68}O_6Na$ 679.4908; found, 679.4892.

3.2.10. Synthesis of 3-hexadecanoyl-1-*O*-(*p*-methoxybenzyl)-2-[(9Z,12Z)-octadeca-9,12-dienoyl)]-*sn*-glycerol, (*R*)-**10e**

The same procedure was followed as described for (*R*)-**10a**, using (*R*)-**9c** (106mg, 0.24 mmol), linoleic acid (76mg, 0.27 mmol), DMAP (23mg, 0.18 mmol) and EDCI (54mg, 0.28 mmol) in dry dichloromethane (4mL). The crude product was purified by flash column chromatography using ethyl acetate/hexane (1:9) as eluent affording the product (*R*)-**10e** as a colourless liquid (149mg, 89% yield). TLC (Silica, ethyl acetate:petroleum ether, 20:80): Rf = 0.63. $[\alpha]^{20}_D$ = -6.80 (c. 2.06, CH_2Cl_2). IR (NaCl, ν_{max} / cm^{-1}): 2925 (s), 2854 (s), 1742 (s), 1612 (w), 1466 (m), 1248 (s), 1172(s), 1038 (vs). ^1H NMR (400 MHz, $CDCl_3$) δ_H: δ 7.23 (d, *J*=8.6 Hz, 2H, Ph-H), 6.87 (d, *J*=8.8 Hz, 2H, Ph-H), 5.35 (m, 4H, =CH), 5.22 (m, 1H, CH *sn*-2), 4.46 (ABq, $\Delta\delta_{AB}$=0.04, *J*=11.6, 2H, $PhCH_2$), 4.33 (dd, *J*=11.9, 3.9 Hz, 1H, CH_2 *sn*-3), 4.17 (dd, *J*=11.9, 6.4 Hz, 1H, CH_2 *sn*-3), 3.80 (s, 3H, OCH_3), 3.55 (dd, *J*=5.2, 1.3 Hz, 2H, CH_2 *sn*-1), 2.77 (t, *J*=6.6 Hz, 2H, =CHCH_2CH=), 2.31 (t, *J*=7.4 Hz, 2H, CH_2COO SFA), 2.27 (t, *J*=7.6 Hz, 4H, CH_2COO PUFA), 2.05 (q, *J*=7.5 Hz, 4H, CH_2CH=), 1.66-1.54 (m, 4H, CH_2CH_2COO), 1.39-1.21 (m, 40H, CH_2), 0.89 (t, *J*=6.8 Hz, 3H, CH_2CH_3 PUFA), 0.88 (t, *J*=6.8 Hz, 3H, CH_2CH_3 SFA) ppm. $^{13}C\{H\}$ NMR (101 MHz, $CDCl_3$) δ_C: 173.6, 173.2, 159.5, 130.4, 130.2, 129.9, 129.4 (2), 128.2, 128.04, 114.0 (2), 73.1, 70.2, 68.1, 62.8, 55.4, 34.5, 34.3, 32.1, 31. 7, 29.9 (3), 29.81 (2), 29.78 (2), 29.6, 29.51, 29.49, 29.44, 29.35, 29.3 (2), 29.2, 27.4, 25.8, 25.1, 25.0, 22.8, 22.7, 14.3, 14.2 ppm. $[M + Na]^+$ calcd for $C_{45}H_{76}O_6Na$ 735.5540; found, 735.5489.

3.2.11. Synthesis of 3-dodecanoyl-2-[(9Z)-octadec-9-enoyl)]-*sn*-glycerol, (*R*)-**11a**

Diacylglycerol (*R*)-**10a** (306mg, 0.465 mmol) was added to a 25mL round bottom flask in dichloromethane (6 mL) equipped with a magnetic stirrer. Water (1 mL) was pipetted to the solution, and it was cooled down to 0°C. Subsequently 2,3-dichloro-5,6-dicyano-1,4-benzoquinone (DDQ) (137mg, 0.60 mmol) was added, which turned the solution to a dark green colour. The mixture was stirred under nitrogen for an hour and then the cooling bath was removed. It was allowed to stir for additional three hours at room temperature, during which the colour slowly changed to colourless with a bright red aqueous phase. When all the dark colour had vanished, the reaction was complete as indicated by TLC monitoring. The reaction mixture was extracted three times with dichloromethane and the combined

organic layers washed with $NaHCO_3$, water and brine. Then they were dried over $MgSO_4$ and concentrated *in vacuo*, and the crude concentrate then purified by flash column chromatography with 4% boric acid impregnated silica gel using ethyl acetate:hexane (1:9) as eluent affording the product (*R*)-**11a** as a colourless liquid (227mg, 91% yield). TLC (Silica, ethyl acetate:petroleum ether, 20:80): Rf = 0.41. $[\alpha]^{20}_D$ = +2.43 (c. 4.24, CH_2Cl_2). IR (NaCl, ν_{max} / cm^{-1}): 3480 (br), 3004 (m), 2925 (vs), 2854 (vs), 1744 (s), 1466 (m), 1352 (m), 1167 (s). ^1H NMR (400 MHz, $CDCl_3$) δ_H: 5.34 (m, 2H, =CH), 5.08 (p, *J*=4.9 Hz, 1H, CH *sn*-2), 4.32 (dd, *J* = 12.0, 4.6 Hz, 1H, CH_2 *sn*-3), 4.23 (dd, *J* = 12.0, 5.7 Hz, 1H, CH_2 *sn*-3), 3.72 (br s, 2H, CH_2 *sn*-1), 2.34 (t, *J*=7.5 Hz, 2H, CH_2COO SFA), 2.32 (t, *J*=7.6 Hz, 2H, CH_2COO MUFA), 2.09 (br s, 1H, OH), 2.01 (q, *J*=6.6 Hz, 4H, CH_2CH=), 1.62 (m, 4H, CH_2CH_2COO), 1.39– 1.22 (m, 36H, CH_2), 0.88 (t, *J*=6.7, 6H, CH_2CH_3) ppm. $^{13}C\{H\}$ NMR (101 MHz, $CDCl_3$) δ_C: 173.9, 173.5, 130.2, 129.8, 72.3, 62.1, 61.7, 34.4, 34.2, 32.0 (2), 29.9, 29.84, 29.75 (2), 29.7, 29.6, 29.5 (2), 29.4, 29.32, 29.29, 29.25 (2), 29.2, 27.4, 27.3, 25.1, 25.0, 22.8 (2), 14.3 (2) ppm. $[M + Na]^+$ calcd for $C_{33}H_{62}O_5Na$ 561.4489; found, 561.4470.

3.2.12. Synthesis of 2-[(9*Z*)-octadec-9-enoyl)]-3-tetradecanoyl-*sn*-glycerol, (*R*)-**11b**

The same procedure was followed as described for (*R*)-**11a**, using (*R*)-**10b** (258mg, 0.38 mmol) and DDQ (111mg, 0.49 mmol) in dichloromethane (6 mL) and water (1 mL). The crude product was purified by flash column chromatography with 4% boric acid impregnated silica gel using ethyl acetate:hexane (1:9) as eluent affording the product (*R*)-**11b** as a colourless liquid (195mg, 92% yield). TLC (Silica, ethyl acetate:petroleum ether, 20:80): Rf = 0.37. $[\alpha]^{20}_D$ = +2.09 (c. 2.73, CH_2Cl_2). IR (ATR, ν_{max} / cm^{-1}): 3492 (br), 2922 (vs), 2852 (vs), 1741 (s), 1465 (m), 1377 (m), 1162 (s). ^1H NMR (400 MHz, $CDCl_3$) δ_H: 5.34 (m, 2H, =CH), 5.08 (m, 1H, CH *sn*-2), 4.32 (dd, *J*=11.9, 4.6 Hz, 1H, CH_2 *sn*-3), 4.23 (dd, *J*=11.9, 5.6 Hz, 1H, CH_2 *sn*-3), 3.73 (br s, 2H, CH_2 *sn*-1), 2.34 (t, *J*=7.5 Hz, 2H, CH_2COO SFA), 2.32 (t, *J*=7.7 Hz, 2H, CH_2COO MUFA), 2.01 (q, *J*=6.5 Hz, 4H, CH_2CH=), 1.62 (m, 4H, CH_2CH_2COO), 1.36–1.21 (m, 40H, CH_2), 0.87 (t, *J*=6.7, 6H, CH_2CH_3) ppm. $^{13}C\{H\}$ NMR (101 MHz, $CDCl_3$) δ_C: 174.0, 173.6, 130.2, 129.8, 72.3, 62.1, 61.7, 34.4, 34.3, 32.07, 32.05, 29.91, 29.85, 29.83, 29.80 (2), 29.76, 29.7, 29.6, 29.50, 29.47, 29.46, 29.4, 29.33, 29.27 (2), 29.2, 27.4, 27.3, 25.1, 25.0, 22.8 (2), 14.3 (2) ppm. $[M + Na]^+$ calcd for $C_{35}H_{66}O_5Na$ 589.4802; found, 589.4712.

3.2.13. Synthesis of 3-hexadecanoyl-2-[(9*Z*)-octadec-9-enoyl)]-*sn*-glycerol, (*R*)-**11c**

The same procedure was followed as described for (*R*)-**11a**, using (*R*)-**10c** (238mg, 0.33 mmol) and DDQ (98mg, 0.43 mmol) in dichloromethane (6 mL) and water (1 mL). The crude product was purified by flash column chromatography with 4% boric acid impregnated silica gel using ethyl acetate:hexane (1:9) as eluent affording the product (*R*)-**11c** as a colourless liquid (169mg, 85% yield). TLC (Silica, ethyl acetate:petroleum ether, 20:80): Rf = 0.37. $[\alpha]^{20}_D$ = +2.10 (c. 4.48, CH_2Cl_2). IR (NaCl, ν_{max} / cm^{-1}): 3507 (br), 3004 (m), 2925 (vs), 2854 (vs), 1743 (s), 1466 (m), 1378 (m), 1165 (s). ^1H NMR (400 MHz, $CDCl_3$) δ_H: 5.34 (m, 2H, =CH), 5.08 (m, 1H, CH *sn*-2), 4.32 (dd, *J*=11.9, 4.5 Hz, 1H, CH_2 *sn*-3), 4.23 (dd, *J*=12.0, 5.6 Hz, 1H, CH_2 *sn*-3), 3.73 (m, 2H, CH_2 *sn*-1), 2.34 (t, *J*=7.5 Hz, 2H, CH_2COO SFA), 2.32 (t, *J*=7.6 Hz, 2H, CH_2COO MUFA), 2.11 (br s, 1H, OH), 2.01 (q, *J*=6.3 Hz, 4H, CH_2CH=), 1.63 (m, 4H, CH_2CH_2COO), 1.36–1.22 (m, 44H, CH_2), 0.88 (t, *J*=6.7, 6H, CH_2CH_3) ppm. $^{13}C\{H\}$ NMR (101 MHz, $CDCl_3$) δ_C: 174.0, 173.6, 130.2, 129.8, 72.3, 62.1, 61.7, 34.4, 34.3, 32.07, 32.05, 29.9, 29.84 (3), 29.83, 29.80 (2), 29.76, 29.7, 29.6, 29.51, 29.47 (2), 29.4, 29.32, 29.26 (2), 29.2, 27.4, 27.3, 25.1, 25.0, 22.8 (2), 14.3 (2) ppm. $[M + Na]^+$ calcd for $C_{37}H_{70}O_5Na$ 617.5115; found, 617.5118.

3.2.14. Synthesis of 3-hexadecanoyl-2-[(9*Z*,12*Z*)-octadeca-9,12-dienoyl)]-*sn*-glycerol, (*R*)-**11e**

Diacylglycerol (*R*)-**10e** (1297mg, 1.82 mmol) was added to a 50mL round bottom flask in dichloromethane (5mL) equipped with a magnetic stirrer. Then water (3mL) was pipetted to the solution. Subsequently 2,3-dichloro-5,6-dicyano-1,4-benzoquinone (412mg, 1.82 mmol) was dissolved in dichloromethane (10mL) and slowly added dropwise to the solution over a 30min period. The mixture was stirred vigorously for additional 1.5h until the characteristic dark colour had vanished. The reaction

mixture was extracted three times with dichloromethane and the combined organic layers washed with $NaHCO_3$, water and brine. Then they were dried over Na_2SO_4 and concentrated *in vacuo*, and the crude concentrate then purified by flash column chromatography with 4% boric acid impregnated silica gel using ethyl acetate:hexane (1:9) as eluent affording the product (*R*)-**11e** as a colourless liquid (390mg, 36% yield) along with unreacted (*R*)-**10e** (562mg, 43% recovery). TLC (Silica, ethyl acetate:petroleum ether, 20:80): Rf = 0.40. $[\alpha]^{20}_D$ = +2.45 (c. 2.57, CH_2Cl_2). IR (ATR, ν_{max} / cm^{-1}): 3213 (br), 3009 (m), 2925 (vs), 2853 (vs), 1713 (vs), 1465 (m), 1349 (m), 1162 (s). ^1H NMR (400 MHz, $CDCl_3$) δ_H: 5.36 (m, 4H, =CH), 5.08 (m, 1H, CH *sn*-2), 4.32 (dd, *J*=11.9, 4.6 Hz, 1H, CH_2 *sn*-3), 4.23 (dd, *J*=11.9, 5.6 Hz, 1H, CH_2 *sn*-3), 3.73 (m, 2H, CH_2 *sn*-1), 2.77 (t, *J*=6.6 Hz, 2H, =CHCH_2CH=), 2.32 (t, *J*=7.7 Hz, 2H, CH_2COO PUFA), 2.31 (t, *J*=7.6 Hz, 4H, CH_2COO SFA), 2.05 (q, *J*=6.8 Hz, 4H, CH_2CH=), 1.65–1.58 (m, 4H, CH_2CH_2COO), 1.33–1.25 (m, 38H, CH_2), 0.89 (t, *J*=6.9, 3H, CH_2CH_3 PUFA), 0.88 (t, *J*=7.0, 6H, CH_2CH_3) ppm. $^{13}C\{H\}$ NMR (101 MHz, $CDCl_3$) δ_C: 173.9, 173.5, 130.4, 130.1, 128.2, 128.0, 72.3, 62.1, 61.7, 34.4, 34.3, 32.1, 31.7, 29.9 (3), 29.81 (2), 29.76, 29.6, 29.51, 29.50, 29.4, 29.33, 29.27 (2), 29.2, 27.4 (2), 25.8 (2), 25.1, 25.0, 22.8, 22.7, 14.3, 14.2 ppm. $[M + Na]^+$ calcd for $C_{37}H_{68}O_5Na$ 615.4964; found, 615.5067.

3.2.15. Synthesis of 3-dodecanoyl-1-[(9*Z*,12*Z*)-octadeca-9,12-dienoyl)]-2-[(9*Z*)-octadec-9-enoyl)]-*sn*-glycerol, (*S*)-**1**

Diacylglycerol (*R*)-**11a** (72mg, 0.13 mmol) and linoleic acid (43mg, 0.15 mmol) in dry dichloromethane (3 mL) were added to a 10 mL flame-dried round bottom flask equipped with a magnetic stirrer. Then 4-dimethylaminopyridine (DMAP) (13mg, 0.11 mmol) and 1-ethyl-3-(3-dimethylaminopropyl)carbodiimide (EDCI) (31mg, 0.16 mmol) were added to the solution and stirred at room temperature, under nitrogen for 20 h. After that time, the reaction mixture was passed through a short column packed with silica gel by using ethyl acetate. The solvent was removed *in vacuo,* and the crude concentrate then purified by flash column chromatography using ethyl acetate:hexane (1:19) as eluent affording the product (*S*)-**1** as a colourless liquid (91mg, 85% yield). TLC (Silica, ethyl acetate:petroleum ether, 20:80): Rf = 0.75. $[\alpha]^{20}_D$ = +0.03 (c. 2.96, CH_2Cl_2). IR (NaCl, ν_{max} / cm^{-1}): 3008 (s), 2926 (vs), 2855 (s), 1747 (s), 1464 (m), 1378 (m), 1161 (s). ^1H NMR (400 MHz, $CDCl_3$) δ_H: 5.35 (m, 6H, =CH), 5.26 (m, 1H, CH *sn*-2), 4.29 (dd, *J*=11.9, 4.3 Hz, 2H, CH_2 *sn*-1/*sn*-3), 4.14 (dd, *J*=11.9, 6.0 Hz, 2H, CH_2 *sn*-1/*sn*-3), 2.77 (t, *J*=6.6 Hz, 2H, =CHCH_2CH=), 2.32 (t, *J*=7.5 Hz, 2H, CH_2COO), 2.31 (t, *J*=7.6 Hz, 4H, CH_2COO), 2.04 (q, *J*=6.8 Hz, 4H, CH_2CH= MUFA), 2.01 (q, *J*=6.6 Hz, 4H, CH_2CH= PUFA), 1.65-1.57 (m, 6H, CH_2CH_2COO), 1.38–1.21 (m, 50H, CH_2), 0.89 (t, *J*=6.9, 3H, CH_2CH_3 PUFA), 0.88 (t, *J*=6.7, 6H, CH_2CH_3) ppm. $^{13}C\{H\}$ NMR (101 MHz, $CDCl_3$) δ_C: 173.43, 173.38, 173.0, 130.4, 130.17, 130.15, 129.8, 128.2, 128.0, 69.0, 62.2 (2), 34.3, 34.20, 34.17, 32.1 (2), 31.7, 29.91, 29.87, 29.8 (3), 29.7, 29.6, 29.49 (2), 29.47 (2), 29.41, 29.35, 29.32, 29.28, 29.27 (2), 29.23, 29.20, 27.4, 27.34 (2), 27.32, 25.8, 25.03, 25.01, 24.98, 22.8 (2), 22.7, 14.3 (2), 14.2 ppm. $[M + Na]^+$ calcd for $C_{51}H_{92}O_6Na$ 823.6786; found, 823.6766.

3.2.16. Synthesis of 3-hexadecanoyl-1-[(9*Z*,12*Z*)-octadeca-9,12-dienoyl)]-2-[(9*Z*)-octadec-9-enoyl)]-*sn*-glycerol, (*S*)-**2**

The same procedure was followed as described for (*S*)-**1**, using (*R*)-**11c** (144mg, 0.24 mmol), linoleic acid (78mg, 0.28 mmol), DMAP (24mg, 0.19 mmol) and EDCI (56mg, 0.29 mmol) in dry dichloromethane (3mL). The crude product was purified by flash column chromatography using ethyl acetate:hexane (1:19) as eluent affording the product (*S*)-**2** as a colourless liquid (190mg, 92% yield). TLC (Silica, ethyl acetate:petroleum ether, 10:90): Rf = 0.54. $[\alpha]^{20}_D$ = -0.01 (c. 6.90, CH_2Cl_2). IR (NaCl, ν_{max} / cm^{-1}): 3008 (s), 2925 (s), 2854 (s), 1747 (s), 1466 (m), 1377 (m), 1163 (s). ^1H NMR (400 MHz, $CDCl_3$) δ_H: 5.35 (m, 6H, =CH), 5.26 (m, 1H, CH *sn*-2), 4.29 (dd, *J*=11.9, 4.3 Hz, 2H, CH_2 *sn*-1/*sn*-3), 4.14 (dd, *J*=11.9, 6.0 Hz, 2H, CH_2 *sn*-1/*sn*-3), 2.77 (t, *J*=6.6 Hz, 2H, =CHCH_2CH=), 2.32 (t, *J*=7.6 Hz, 2H, CH_2COO), 2.31 (t, *J*=7.6 Hz, 4H, CH_2COO), 2.04 (q, *J*=6.8 Hz, 4H, CH_2CH= MUFA), 2.01 (q, *J*=6.7 Hz, 4H, CH_2CH= PUFA), 1.64–1.58 (m, 6H, CH_2CH_2COO), 1.38–1.23 (m, 58H, CH_2), 0.89 (t, *J*=6.9, 3H, CH_2CH_3 PUFA), 0.88 (t, *J*=6.8, 6H, CH_2CH_3) ppm. $^{13}C\{H\}$ NMR (101 MHz, $CDCl_3$) δ_C: 173.5, 173.4, 173.0, 130.4, 130.17, 130.15, 129.8, 128.2, 128.0, 69.0, 62.2 (2), 34.3, 34.20,

34.17, 32.07, 32.05, 31.7, 29.91, 29.87, 29.84 (2), 29.83, 29.81 (2), 29.77, 29.76, 29.7, 29.63, 29.58, 29.51, 29.49, 29.47, 29.42, 29.35, 29.32, 29.28, 29.27 (2), 29.23, 29.20, 27.4, 27.34 (2), 27.32, 25.8, 25.03, 25.01, 24.98, 22.8 (2), 22.7, 14.3 (2), 14.2 ppm. $[M + Na]^+$ calcd for $C_{55}H_{100}O_6Na$ 879.7412; found, 879.7412.

3.2.17. Synthesis of 1-dodecanoyl-2-[(9Z)-octadec-9-enoyl)]-3-tetradecanoyl-*sn*-glycerol, (*R*)-**12a**

The same procedure was followed as described for (*S*)-**1**, using (*R*)-**11b** (178mg, 0.32 mmol), lauric acid (74mg, 0.37 mmol), DMAP (31mg, 0.26 mmol) and EDCI (74mg, 0.38 mmol) in dry dichloromethane (3mL). The crude product was purified by flash column chromatography using ethyl acetate:hexane (1:19) as eluent affording the product (*R*)-**12a** as a colourless liquid (216mg, 90% yield). TLC (Silica, ethyl acetate:petroleum ether, 20:80): Rf = 0.77. $[\alpha]^{20}_D$ = +0.04 (c. 2.84, CH_2Cl_2). IR (NaCl, ν_{max} / cm^{-1}): 2922 (vs), 2853 (s), 1743 (s), 1465 (m), 1377 (m), 1157 (s). ^1H NMR (400 MHz, $CDCl_3$) δ_H: 5.34 (m, 2H, =CH), 5.26 (m, 1H, CH *sn*-2), 4.29 (dd, *J*=11.9, 4.3 Hz, 2H, CH_2 *sn*-1/*sn*-3), 4.14 (dd, *J*=11.9, 6.0 Hz, 2H, CH_2 *sn*-1/*sn*-3), 2.32 (t, *J*=7.5 Hz, 2H, CH_2COO MUFA), 2.31 (t, *J*=7.7 Hz, 4H, CH_2COO SFA), 2.01 (q, *J*=6.6 Hz, 4H, C*H*$_2$CH=), 1.66-1.58 (m, 6H, C*H*$_2$$CH_2$COO), 1.36–1.23 (m, 56H, CH_2), 0.88 (t, *J*=6.9, 9H, CH_2C*H*$_3$) ppm. $^{13}C\{H\}$ NMR (101 MHz, $CDCl_3$) δ_C: 173.5 (2), 173.0, 130.2, 129.8, 69.0, 62.2 (2), 34.4, 34.2 (2), 32.07, 32.06 (2), 29.92, 29.87, 29.83, 29.80 (2), 29.77 (2), 29.76, 29.7, 29.6 (2), 29.51, 29.48 (2), 29.46, 29.42 (2), 29.35, 29.28, 29.27 (2), 29.2, 27.4, 27.3, 25.04, 25.01 (2), 22.8 (3), 14.3 (3) ppm. $[M + Na]^+$ calcd for $C_{47}H_{88}O_6Na$ 771.6473; found, 771.6463.

3.2.18. Synthesis of 1-decanoyl-3-hexadecanoyl-2-[(9Z)-octadec-9-enoyl)]-*sn*-glycerol, (*R*)-**12b**

The same procedure was followed as described for (*S*)-**1**, using (*R*)-**11c** (200mg, 0.34 mmol), capric acid (67mg, 0.39 mmol), DMAP (33mg, 0.27 mmol) and EDCI (77mg, 0.40 mmol) in dry dichloromethane (3mL). The crude product was purified by flash column chromatography using ethyl acetate:hexane (1:19) as eluent affording the product (*R*)-**12b** as a colourless liquid (247mg, 98% yield). TLC (Silica, ethyl acetate:petroleum ether, 20:80): Rf = 0.75. $[\alpha]^{20}_D$ = +0.05 (c. 3.02, CH_2Cl_2). IR (ATR, ν_{max} / cm^{-1}): 2925 (vs), 2854 (s), 1742 (s), 1467 (m), 1377 (m), 1156 (s). ^1H NMR (400 MHz, $CDCl_3$) δ_H: 5.34 (m, 2H, =CH), 5.26 (m, 1H, CH *sn*-2), 4.29 (dd, *J*=11.9, 4.3 Hz, 2H, CH_2 *sn*-1/*sn*-3), 4.14 (dd, *J*=11.9, 6.0 Hz, 2H, CH_2 *sn*-1/*sn*-3), 2.32 (t, *J*=7.6 Hz, 2H, CH_2COO MUFA), 2.31 (t, *J*=7.7 Hz, 4H, CH_2COO SFA), 2.01 (q, *J*=6.3 Hz, 4H, C*H*$_2$CH=), 1.63-1.52 (m, 6H, C*H*$_2$$CH_2$COO), 1.36–1.23 (m, 56H, CH_2), 0.88 (t, *J*=6.9, 9H, CH_2C*H*$_3$) ppm. $^{13}C\{H\}$ NMR (101 MHz, $CDCl_3$) δ_C: 173.5 (2), 173.0, 130.2, 129.8, 69.0, 62.2 (2), 34.4, 34.2 (2), 32.08, 32.06, 32.0, 29.92, 29.87, 29.85 (3), 29.81 (2), 29.78, 29.7, 29.63, 29.58, 29.52, 29.48 (2), 29.42 (3), 29.35, 29.29, 29.27, 29.26, 29.2, 27.4, 27.3, 25.04, 25.02 (2), 22.84 (2), 22.82, 14.3 (3) ppm. $[M + Na]^+$ calcd for $C_{47}H_{88}O_6Na$ 771.6473; found, 771.6421.

3.2.19. Synthesis of 1-dodecanoyl-3-hexadecanoyl-2-[(9Z)-octadec-9-enoyl)]-*sn*-glycerol, (*R*)-**12c**

The same procedure was followed as described for (*S*)-**1**, using (*R*)-**11c** (202mg, 0.34 mmol), lauric acid (77mg, 0.39 mmol), DMAP (33mg, 0.27 mmol) and EDCI (77mg, 0.40 mmol) in dry dichloromethane (3mL). The crude product was purified by flash column chromatography using ethyl acetate:hexane (1:19) as eluent affording the product (*R*)-**12c** as a colourless liquid (264mg, 99% yield). TLC (Silica, ethyl acetate:petroleum ether, 20:80): Rf = 0.72. $[\alpha]^{20}_D$ = +0.02 (c. 2.69, CH_2Cl_2). IR (ATR, ν_{max} / cm^{-1}): 2926 (vs), 2856 (s), 1745 (s), 1465 (m), 1378 (m), 1159 (s). ^1H NMR (400 MHz, $CDCl_3$) δ_H: 5.34 (m, 2H, =CH), 5.26 (m, 1H, CH *sn*-2), 4.29 (dd, *J*=11.9, 4.3 Hz, 2H, CH_2 *sn*-1/*sn*-3), 4.14 (dd, *J*=11.9, 5.9 Hz, 2H, CH_2 *sn*-1/*sn*-3), 2.32 (t, *J*=7.5 Hz, 2H, CH_2COO MUFA), 2.31 (t, *J*=7.6 Hz, 4H, CH_2COO SFA), 2.01 (q, *J*=5.5 Hz, 4H, C*H*$_2$CH=), 1.65-1.57 (m, 6H, C*H*$_2$$CH_2$COO), 1.34–1.23 (m, 60H, CH_2), 0.88 (t, *J*=6.8, 9H, CH_2C*H*$_3$) ppm. $^{13}C\{H\}$ NMR (101 MHz, $CDCl_3$) δ_C: 173.5 (2), 173.0, 130.2, 129.8, 69.0, 62.2 (2), 34.4, 34.2 (2), 32.08, 32.06 (2), 29.92, 29.88, 29.85 (3), 29.81 (2), 29.78, 29.77 (2), 29.7, 29.63, 29.58, 29.52, 29.48 (2), 29.42 (3), 29.35, 29.29, 29.27, 29.26, 29.2, 27.4, 27.3, 25.04, 25.02 (2), 22.8 (3), 14.3 (3) ppm. $[M + Na]^+$ calcd for $C_{49}H_{92}O_6Na$ 799.6786; found, 799.6748.

3.2.20. Synthesis of 3-hexadecanoyl-2-[(9Z)-octadec-9-enoyl)]-1-tetradecanoyl-*sn*-glycerol, (*R*)-**12d**

The same procedure was followed as described for (*S*)-**1**, using (*R*)-**11c** (200mg, 0.34 mmol), myristic acid (88mg, 0.39 mmol), DMAP (33mg, 0.27 mmol) and EDCI (77mg, 0.40 mmol) in dry dichloromethane (3mL). The crude product was purified by flash column chromatography using ethyl acetate:hexane (1:19) as eluent affording the product (*R*)-**12d** as a colourless liquid (259mg, 96% yield). TLC (Silica, ethyl acetate:petroleum ether, 20:80): Rf = 0.73. $[\alpha]^{20}_{D}$ = -0.02 (c. 3.78, CH_2Cl_2). IR (ATR, ν_{max} / cm^{-1}): 2925 (vs), 2855 (s), 1746 (s), 1466 (m), 1378 (m), 1161 (s). ^{1}H NMR (400 MHz, $CDCl_3$) δ_H: 5.34 (m, 2H, =CH), 5.26 (tt, *J*=5.9, 4.3 Hz, 1H, CH *sn*-2), 4.29 (dd, *J*=11.9, 4.3 Hz, 2H, CH_2 *sn*-1/*sn*-3), 4.14 (dd, *J*=11.9, 5.9 Hz, 2H, CH_2 *sn*-1/*sn*-3), 2.32 (t, *J*=7.6 Hz, 2H, CH_2COO MUFA), 2.31 (t, *J*=7.6 Hz, 4H, CH_2COO SFA), 2.01 (q, *J*=6.2 Hz, 4H, C*H*$_2$CH=), 1.66-1.57 (m, 6H, C*H*$_2$CH$_2$COO), 1.35–1.22 (m, 64H, CH_2), 0.88 (t, *J*=6.7, 9H, CH_2C*H*$_3$) ppm. $^{13}C\{H\}$ NMR (101 MHz, $CDCl_3$) δ_C: 173. 5 (2), 173.0, 130.2, 129.8, 69.0, 62.2 (2), 34.4, 34.2 (2), 32.08 (2), 32.06, 29.92, 29.88, 29.84 (3), 29.81 (4), 29.78 (3), 29.7, 29.6 (2), 29.52 (2), 29.48, 29.47, 29.43 (2), 29.35, 29.28, 29.27 (2), 29.2, 27.4, 27.3, 25.04, 25.02 (2), 22.8 (3), 14.3 (3) ppm. $[M + Na]^+$ calcd for $C_{51}H_{96}O_6Na$ 827.7099; found, 827.7049.

3.2.21. Synthesis of 3-hexadecanoyl-1-icosanoyl-2-[(9Z)-octadec-9-enoyl)]-*sn*-glycerol, (*R*)-**12e**

The same procedure was followed as described for (*S*)-**1**, using (*R*)-**11c** (200mg, 0.34 mmol), arachidic acid (121mg, 0.39 mmol), DMAP (33mg, 0.27 mmol) and EDCI (77mg, 0.40 mmol) in dry dichloromethane (3mL). The crude product was purified by flash column chromatography using ethyl acetate:hexane (1:19) as eluent affording the product (*R*)-**12e** as a colourless liquid (299mg, 97% yield). TLC (Silica, ethyl acetate:petroleum ether, 20:80): Rf = 0.89. $[\alpha]^{20}_{D}$ = +0.01 (c. 2.80, CH_2Cl_2). IR (ATR, ν_{max} / cm^{-1}): 2915 (s), 2848 (s), 1733 (s), 1464 (m), 1364 (w), 1161 (s). ^{1}H NMR (400 MHz, $CDCl_3$) δ_H: 5.34 (m, 2H, =CH), 5.26 (tt, *J*=6.0, 4.3 Hz, 1H, CH *sn*-2), 4.29 (dd, *J*=11.9, 4.3 Hz, 2H, CH_2 *sn*-1/*sn*-3), 4.14 (dd, *J*=11.9, 6.0 Hz, 2H, CH_2 *sn*-1/*sn*-3), 2.32 (t, *J*=7.5 Hz, 2H, CH_2COO MUFA), 2.31 (t, *J*=7.6 Hz, 4H, CH_2COO SFA), 2.01 (q, *J*=6.4 Hz, 4H, C*H*$_2$CH=), 1.65-1.57 (m, 6H, C*H*$_2$CH$_2$COO), 1.34–1.23 (m, 76H, CH_2), 0.88 (t, *J*=6.7, 9H, CH_2C*H*$_3$) ppm. $^{13}C\{H\}$ NMR (101 MHz, $CDCl_3$) δ_C: 173.5 (2), 173.01, 130.2, 129.8, 69.0, 62.2 (2), 34.4, 34.2 (2), 32.08 (2), 32.06, 29.92, 29.86 (10), 29.83, 29.82 (4), 29.78 (2), 29.7, 29.6 (2), 29.52 (2), 29.48, 29.47, 29.43 (2), 29.35, 29.28, 29.27 (2), 29.2, 27.4, 27.3, 25.04, 25.02 (2), 22.8 (3), 14.3 (3) ppm. $[M + Na]^+$ calcd for $C_{57}H_{108}O_6Na$ 911.8038; found, 911.7982.

3.2.22. Synthesis of 1-dodecanoyl-3-hexadecanoyl-2-[(9*Z*,12*Z*)-octadeca-9,12-dienoyl)]-*sn*-glycerol, (*R*)-**12f**

The same procedure was followed as described for (*S*)-**1**, using (*R*)-**11e** (150mg, 0.25 mmol), lauric acid (58mg, 0.29 mmol), DMAP (25mg, 0.20 mmol) and EDCI (58mg, 0.30 mmol) in dry dichloromethane (2mL). The crude product was purified by flash column chromatography using ethyl acetate:hexane (1:19) as eluent affording the product (*R*)-**12f** as a colourless liquid (168mg, 86% yield). TLC (Silica, ethyl acetate:petroleum ether, 20:80): Rf = 0.75. $[\alpha]^{20}_{D}$ = +0.09 (c. 6.92, CH_2Cl_2). IR (ATR, ν_{max} / cm^{-1}): 2919 (s), 2850 (s), 1745 (s), 1452 (w), 1378 (w), 1083 (s). ^{1}H NMR (400 MHz, $CDCl_3$) δ_H: 5.35 (m, 4H, =CH), 5.26 (m, 1H, CH *sn*-2), 4.29 (dd, *J*=11.9, 4.3 Hz, 2H, CH_2 *sn*-1/*sn*-3), 4.14 (dd, *J*=11.9, 6.0 Hz, 2H, CH_2 *sn*-1/*sn*-3), 2.77 (t, *J*=5.9 Hz, 2H, =CHC*H*$_2$CH=), 2.32 (t, *J*=7.6 Hz, 2H, CH_2COO), 2.31 (t, *J*=7.6 Hz, 4H, CH_2COO), 2.04 (q, *J*=6.9 Hz, 2H, C*H*$_2$CH=), 2.01 (q, *J*=6.7 Hz, 4H, C*H*$_2$CH=), 1.64–1.58 (m, 6H, C*H*$_2$CH$_2$COO), 1.34–1.23 (m, 54H, CH_2), 0.89 (t, *J*=6.9, 3H, CH_2C*H*$_3$ PUFA), 0.88 (t, *J*=6.8, 6H, CH_2C*H*$_3$ SFA) ppm. $^{13}C\{H\}$ NMR (101 MHz, $CDCl_3$) δ_C: 173.5 (2), 173.0, 130.4, 130.1, 128.2, 128.0, 69.0, 62.2 (2), 34.4, 34.2 (2), 32.1 (2), 31.7, 29.9 (3), 29.81 (3), 29.77 (2), 29.6 (2), 29.52, 29.49 (2), 29.42 (2), 29.35, 29.29, 29.27, 29.24 (2), 29.20, 27.4 (2), 25. 8, 25.0 (2), 22.8 (2), 22.7, 14.3 (2), 14.2 ppm. $[M + Na]^+$ calcd for $C_{49}H_{90}O_6Na$ 797.6630; found, 797.6620.

*3.3. Synthesis of the USU' subclass category TAGs (S)-**5** and **6***

3.3.1. Synthesis of oleic acid acetoxime ester, **13**

Oleic acid (500mg, 1.77 mmol) was added to a 100 mL flame-dried two-necked round bottom flask equipped with a magnetic stirrer in dry dichloromethane (8mL). Acetone oxime (130mg, 1.77 mmol), DMAP (43mg, 0.35 mmol) and EDCI (407mg, 2.12 mmol) were added to the solution and it stirred at room

temperature, under nitrogen for 20h. After that time, the reaction mixture was flushed through a short column packed with silica gel with ethyl acetate. The solvent was removed *in vacuo,* and the crude concentrate then purified by flash column chromatography using ethyl acetate:hexane (1:9) as eluent affording the product **13** as a colourless liquid (596mg, quantitative yields). TLC (Silica, ethyl acetate:petroleum ether, 30:70): Rf = 0.59. IR (NaCl, ν_{max} / cm^{-1}): 2925 (s), 2854 (vs), 1765 (vs), 1654 (w), 1460 (m), 1377 (m), 1271 (m), 1136(s). ^1H NMR (400 MHz, $CDCl_3$) δ_H: 5.34 (m, 2H, =CH), 2.40 (t, *J*=7.6 Hz, 2H, CH_2COO), 2.05 (s, 3H, $(CH_3)_2C$=N), 2.01 (m, 4H, CH_2CH=), 1.99 (s, 3H, $(CH_3)_2C$=N), 1.69 (p, *J*=7.5 Hz, 2H, CH_2CH_2COO), 1.38-1.23 (m, 20H, CH_2), 0.87 (t, *J*=6.7 Hz, 3H, CH_2CH_3) ppm. $^{13}C\{H\}$ NMR (101 MHz, $CDCl_3$) δ_C: 171.4, 163.8, 130.2, 129.9, 33.2, 32.0, 29.9, 29.8, 29.7, 29.5 (2), 29.3, 29.2 (2), 27.4, 27.3, 25.1, 22.8, 22.2, 17.1, 14.3 ppm. $[M + Na]^+$ calcd for $C_{21}H_{39}O_2NNa$ 360.2873; found, 360.2879.

3.3.2. Synthesis of 1-*O*-(*p*-methoxybenzyl)-3-[(9*Z*)-octadec-9-enoyl)]-*sn*-glycerol, (*R*)-**14**

PMB-protected glycerol (*S*)-**8** (300mg, 1.41 mmol) and oleic acid acetoxime ester **13** (573mg, 1.70 mmol) were added to a flame dried 5mL round bottom flask. Subsequently, immobilized *Candida antarctica* lipase B (CAL-B) (70mg, 8% w/w) was added, the flask connected to a vacuum pump system (10^{-2} mmHg) and the resulting mixture stirred at room temperature for 6h. After that time, the vacuum was disconnected and additional CAL-B (10mg) along with dried dichloromethane (1.5mL) were added to the flask. The mixture was allowed to stir under nitrogen atmosphere overnight. Then the reaction was complete, and the lipase was filtered off. The solvent was removed *in vacuo,* and the crude concentrate then purified by flash column chromatography with 4% boric acid impregnated silica gel using ethyl acetate:hexane (2:8) as eluent affording the product (*R*)-**14** as a colourless liquid (585mg, 87% yield). TLC (Silica, ethyl acetate:petroleum ether, 30:70): Rf = 0.50. $[\alpha]^{20}_D$ = -0.72 (c. 1.67, CH_2Cl_2). IR (NaCl, ν_{max} / cm^{-1}): 3449 (br), 2925 (s), 2854 (vs), 1739 (vs), 1612 (m), 1463 (m), 1377 (m), 1248 (s). ^1H NMR (400 MHz, $CDCl_3$) δ_H: 7.25 (d, *J*=9.1 Hz, 2H, Ph-H), 6.88 (d, *J*=8.8 Hz, 2H, Ph-H), 5.34 (m, 2H, =CH), 4.49 (s, 2H, $PhCH_2$), 4.17 (dd, *J*=11.5, 4.5 Hz, 1H, CH_2 *sn*-3), 4.12 (dd, *J*=11.5, 6.1 Hz, 1H, CH_2 *sn*-3), 4.01 (m, 1H, CH *sn*-2), 3.81 (s, 3H, OCH_3), 3.52 (dd, *J*=9.6, 4.3 Hz, 1H, CH_2 *sn*-1), 3.46 (dd, *J*=9.6, 6.1 Hz, 1H, CH_2 *sn*-1), 2.48 (d, *J*=4.9 Hz, 1H, OH), 2.32 (t, *J*=7.6 Hz, 2H, CH_2COO), 2.00 (m, 4H, CH_2CH=), 1.61 (m, 2H, CH_2CH_2COO), 1.38-1.23 (m, 20H, CH_2), 0.88 (t, *J*=6.7 Hz, 3H, CH_2CH_3) ppm. $^{13}C\{H\}$ NMR (101 MHz, $CDCl_3$) δ_C: 174.1, 159.5, 130.2, 129.89, 129.87, 129.6 (2), 114.0 (2), 73.3, 70.7, 69.1, 65.5, 55.4, 34.3, 32.0, 29.9, 29.8, 29.7, 29.5 (2), 29.31, 29.25 (2), 27.4, 27.3, 25.0, 22.8, 14.3 ppm. $[M + Na]^+$ calcd for $C_{29}H_{48}O_5Na$ 499.3394; found, 499.3353.

3.3.3. Synthesis of 1-*O*-(*p*-methoxybenzyl)-3-[(9*Z*)-octadec-9-enoyl)]-2-tetradecanoyl-*sn*-glycerol, (*R*)-**15a**

The same procedure was followed as described for (*R*)-**10a**, using (*R*)-**14** (100mg, 0.21 mmol), myristic acid (55mg, 0.24 mmol), DMAP (19mg, 0.17 mmol) and EDCI (48mg, 0.25 mmol) in dry dichloromethane (3mL). The crude product was purified by flash column chromatography using ethyl acetate:hexane (1:9) as eluent affording the product (*R*)-**15a** as a colourless liquid (135mg, 94% yield). TLC (Silica, ethyl acetate:petroleum ether, 20:80): Rf = 0.73. $[\alpha]^{20}_D$ = -6.86 (c. 1.59, CH_2Cl_2). IR (NaCl, ν_{max} / cm^{-1}): 2925 (s), 2854 (s), 1742 (s), 1612 (w), 1461 (s), 1377 (m), 1248 (m), 1172 (m), 1038 (w). ^1H NMR (400 MHz, $CDCl_3$) δ_H: 7.23 (d, *J*=8.8 Hz, 2H, Ph-H), 6.87 (d, *J*=8.8 Hz, 2H, Ph-H), 5.34 (m, 2H, =CH), 5.22 (m, 1H, CH *sn*-2), 4.45 (ABq, $\Delta\delta_{AB}$=0.04, *J*=11.6, 2H, $PhCH_2$), 4.32 (dd, *J*=11.9, 3.8 Hz, 1H, CH_2 *sn*-3), 4.17 (dd, *J*=11.9, 6.4 Hz, 1H, CH_2 *sn*-3), 3.80 (s, 3H, OCH_3), 3.55 (dd, *J*=5.1, 1.3 Hz, 2H, CH_2 *sn*-1), 2.31 (t, *J*=7.5 Hz, 2H, CH_2COO SFA), 2.27 (t, *J*=7.6 Hz, 2H, CH_2COO MUFA), 2.00 (m, 4H, CH_2CH=), 1.64-1.55 (m, 4H, CH_2CH_2COO), 1.35-1.22 (m, 40H, CH_2), 0.88 (t, *J*=6.8 Hz, 6H, CH_2CH_3) ppm. $^{13}C\{H\}$ NMR (101 MHz, $CDCl_3$) δ_C: 173.6, 173.3, 159.5, 130.2, 129.91, 129.86, 129.4 (2), 114.0 (2), 73.1, 70.2, 68.1, 62.9, 55.4, 34.5, 34.3, 32.07, 32.05, 29.91, 29.86, 29.84, 29.81 (2), 29.78, 29. 7, 29.6, 29.51, 29.47 (2), 29.4 (2), 29.34, 29.28, 29.2 (2), 27.4, 27.3, 25.1, 25.0, 22.8, 14.3 (2) ppm. $[M + Na]^+$ calcd for $C_{43}H_{74}O_6Na$ 709.5378; found, 709.5369.

3.3.4. Synthesis of 2-hexadecanoyl-1-*O*-(*p*-methoxybenzyl)-3-[(9*Z*)-octadec-9-enoyl)]-*sn*-glycerol, (*R*)-**15b**

The same procedure was followed as described for (*R*)-**10a**, using (*R*)-**14** (100mg, 0.21 mmol), palmitic acid (62mg, 0.24 mmol), DMAP (19mg, 0.17 mmol) and EDCI (48mg, 0.25 mmol) in dry dichloromethane (3mL). The crude product was purified by flash column chromatography using ethyl acetate:hexane (1:9) as eluent affording the product (*R*)-**15b** as a colourless liquid (137mg, 91% yield). TLC (Silica, ethyl acetate:petroleum ether, 20:80): Rf = 0.73. $[\alpha]^{20}_D$ = -6.75 (c. 1.91, CH_2Cl_2). IR (NaCl, ν_{max} / cm^{-1}): 2924 (s), 2854 (s), 1742 (s), 1613 (w), 1460 (s), 1378 (m), 1248 (m), 1172(m), 1038 (w). ^1H NMR (400 MHz, $CDCl_3$) δ_H: 7.23 (d, *J*=8.8 Hz, 2H, Ph-H), 6.87 (d, *J*=8.8 Hz, 2H, Ph-H), 5.34 (m, 2H, =CH), 5.22 (m, 1H, CH *sn*-2), 4.45 (ABq, $\Delta\delta_{AB}$=0.04, *J*=11.6, 2H, $PhCH_2$), 4.33 (dd, *J*=11.9, 3.8 Hz, 1H, CH_2 *sn*-3), 4.17 (dd, *J*=11.9, 6.5 Hz, 1H, CH_2 *sn*-3), 3.80 (s, 3H, OCH_3), 3.55 (dd, *J*=5.2, 1.3 Hz, 2H, CH_2 *sn*-1), 2.31 (t, *J*=7.5 Hz, 2H, CH_2COO SFA), 2.27 (t, *J*=7.5 Hz, 2H, CH_2COO MUFA), 2.00 (m, 4H, CH_2CH=), 1.63-1.55 (m, 4H, CH_2CH_2COO), 1.36-1.21 (m, 44H, CH_2), 0.88 (t, *J*=6.7 Hz, 6H, CH_2CH_3) ppm. $^{13}C\{H\}$ NMR (101 MHz, $CDCl_3$) δ_C: 173.5, 173.3, 159.5, 130.2, 129.91, 129.86, 129.4 (2), 114.0 (2), 73.1, 70.2, 68.1, 62.9, 55.4, 34.5, 34.3, 32.07, 32.05, 29.92, 29.86, 29.85 (3), 29.81 (2), 29.79, 29.67, 29.65, 29.6, 29.51, 29.47 (2), 29.45, 29.34, 29.28, 29.25 (2), 27.4, 27.3, 25.1, 25.0, 22.8, 14.3 (2) ppm. $[M + Na]^+$ calcd for $C_{45}H_{78}O_6Na$ 737.5691; found, 737.5641.

3.3.5. Synthesis of 3-[(9*Z*)-octadec-9-enoyl)]-2-tetradecanoyl-*sn*-glycerol, (*R*)-1**6a**

The same procedure was followed as described for (*R*)-**11a**, using (*R*)-**15a** (190mg, 0.28 mmol) and DDQ (82mg, 0.36 mmol) in dichloromethane (6mL) and water (1mL). The crude product was purified by flash column chromatography with 4% boric acid impregnated silica gel using ethyl acetate:petroleum ether (1:9) as eluent affording the product (*R*)-**16a** as a colourless liquid (146 mg, 95% yield). TLC (Silica, ethyl acetate:petroleum ether, 20:80): Rf = 0.36. $[\alpha]^{20}_D$ = +2.41 (c. 1.66, CH_2Cl_2). IR (NaCl, ν_{max} / cm^{-1}): 3449 (br), 3004 (m), 2924 (vs), 2854 (vs), 1743 (s), 1466 (m), 1377 (m), 1168 (s). ^1H NMR (400 MHz, $CDCl_3$) δ_H: 5.34 (m, 2H, =CH), 5.08 (m, 1H, CH *sn*-2), 4.32 (dd, *J*=11.9, 4.5 Hz, 1H, CH_2 *sn*-3), 4.23 (dd, *J*=11.9, 5.6 Hz, 1H, CH_2 *sn*-3), 3.73 (m, 2H, CH_2 *sn*-1), 2.34 (t, *J*=7.5 Hz, 2H, CH_2COO SFA), 2.32 (t, *J*=7.8 Hz, 2H, CH_2COO MUFA), 2.01 (q, *J*=5.8 Hz, 4H, CH_2CH=), 1.62 (m, 4H, CH_2CH_2COO), 1.38–1.21 (m, 40H, CH_2), 0.88 (t, *J*=6.7, 6H, CH_2CH_3) ppm. $^{13}C\{H\}$ NMR (101 MHz, $CDCl_3$) δ_C: 173.9, 173.6, 130.2, 129.9, 72.3, 62.1, 61.7, 34.4, 34.2, 32.07, 32.05, 29.92, 29.85, 29.83, 29.80 (2), 29.77, 29.7, 29.6, 29.51, 29.47 (2), 29.4, 29.32, 29.26, 29.2 (2), 27.4, 27.3, 25.1, 25.0, 22.8 (2), 14.3 (2) ppm. $[M + Na]^+$ calcd for $C_{35}H_{66}O_5Na$ 589.4802; found, 589.4797.

3.3.6. Synthesis of 2-hexadecanoyl-3-[(9*Z*)-octadec-9-enoyl)]-*sn*-glycerol, (*R*)-**16b**

The same procedure was followed as described for (*R*)-**11a**, using (*R*)-**15b** (200 mg, 0.28 mmol) and DDQ (83mg, 0.36 mmol) in dichloromethane (6mL) and water (1mL). The crude product was purified by flash column chromatography with 4% boric acid impregnated silica gel using ethyl acetate:petroleum ether (1:9) as eluent affording the product (*R*)-**16b** as a colourless liquid (151 mg, 91% yield). TLC (Silica, ethyl acetate:petroleum ether, 20:80): Rf = 0.35. $[\alpha]^{20}_D$ = +2.39 (c. 1.42, CH_2Cl_2). IR (NaCl, ν_{max} / cm^{-1}): 3475 (br), 3004 (m), 2924 (vs), 2854 (vs), 1744 (s), 1466 (m), 1377 (m), 1168 (s). ^1H NMR (400 MHz, $CDCl_3$) δ_H: 5.34 (m, 2H, =CH), 5.08 (m, 1H, CH *sn*-2), 4.32 (dd, *J*=11.9, 4.5 Hz, 1H, CH_2 *sn*-3), 4.23 (dd, *J*=11.9, 5.6 Hz, 1H, CH_2 *sn*-3), 3.73 (m, 2H, CH_2 *sn*-1), 2.34 (t, *J*=7.6 Hz, 2H, CH_2COO SFA), 2.32 (t, *J*=7.8 Hz, 2H, CH_2COO MUFA), 2.01 (q, *J*=6.6 Hz, 4H, CH_2CH=), 1.60 (m, 4H, CH_2CH_2COO), 1.35–1.22 (m, 44H, CH_2), 0.88 (t, *J*=6.7, 6H, CH_2CH_3) ppm. $^{13}C\{H\}$ NMR (101 MHz, $CDCl_3$) δ_C: 173.9, 173.6, 130.2, 129.9, 72.3, 62.1, 61.7, 34.4, 34.2, 32.08, 32.05, 29.92, 29.85 (3), 29.83, 29.81 (2), 29.77, 29.7, 29.6, 29.51, 29.47 (2), 29.4, 29.32, 29.26, 29.2 (2), 27.4, 27.3, 25.1, 25.0, 22.8 (2), 14.3 (2) ppm. $[M + Na]^+$ calcd for $C_{37}H_{70}O_5Na$ 617.5115; found, 617.5109.

3.3.7. Synthesis of 1-[(9*Z*,12*Z*)-octadeca-9,12-dienoyl)]-3-[(9*Z*)-octadec-9-enoyl)]-2-tetradecanoyl-*sn*-glycerol, (*S*)-**5**

The same procedure was followed as described for (*S*)-**1**, using (*R*)-**16a** (140mg, 0.25 mmol), linoleic acid (81mg, 0.29 mmol), DMAP (25mg, 0.20 mmol) and EDCI (58mg, 0.30 mmol) in dry dichloromethane

(3mL). The crude product was purified by flash column chromatography using ethyl acetate:hexane (1:19) as eluent affording the product (*S*)-**5** as a colourless liquid (195mg, 94% yield). TLC (Silica, ethyl acetate:petroleum ether, 20:80): Rf = 0.80. $[\alpha]^{20}_D$ = +0.05 (c. 5.60, CH_2Cl_2). IR (NaCl, ν_{max} / cm^{-1}): 3008 (m), 2926 (vs), 2856 (vs), 1745 (vs), 1465 (s), 1378 (w), 1163 (s). ^1H NMR (400 MHz, $CDCl_3$) δ_H: 5.35 (m, 6H, =CH), 5.26 (m, 1H, CH *sn*-2), 4.29 (dd, *J*=11.9, 4.3 Hz, 2H, CH_2 *sn*-1/*sn*-3), 4.14 (dd, *J*=11.9, 6.0 Hz, 2H, CH_2 *sn*-1/*sn*-3), 2.77 (t, *J*=6.6 Hz, 2H, =CHC*H*$_2$CH=), 2.32 (t, *J*=7.5 Hz, 2H, CH_2COO SFA), 2.31 (t, *J*=7.5 Hz, 4H, CH_2COO MUFA/PUFA), 2.03 (q, *J*=6.8 Hz, 4H, CH_2CH= MUFA), 2.01 (q, *J*=6.8 Hz, 4H, CH_2CH= PUFA), 1.65–1.57 (m, 6H, C*H*$_2$CH$_2$COO), 1.38–1.23 (m, 54H, CH_2), 0.89 (t, *J*=6.9, 3H, CH$_2$C*H*$_3$ PUFA), 0.88 (t, *J*=6.8, 6H, CH$_2$C*H*$_3$) ppm. $^{13}C\{H\}$ NMR (101 MHz, $CDCl_3$) δ_C: 173.42, 173.41, 173.0, 130.4, 130.2 (2), 129.9, 128.2, 128.1, 69.0, 62.3 (2), 34.4, 34.2 (2), 32.08, 32.06, 31.7, 29.92, 29.86, 29.85 (2), 29.82, 29.81 (2), 29.80 (2), 29.77, 29.68, 29.65, 29.52, 29.50, 29.47 (2), 29.45, 29.33 (2), 29.27 (2), 29.2 (2), 27.38, 27.35 (2), 27.3, 25.8, 25.11, 25.0 (2), 22.8 (2), 22.7, 14.3 (2), 14.2 ppm. $[M + Na]^+$ calcd for $C_{53}H_{96}O_6Na$ 851.7099; found, 851.7082.

3.3.8. Synthesis of 2-hexadecanoyl-1-[(9*Z*,12*Z*)-octadeca-9,12-dienoyl)]-3-[(9*Z*)-octadec-9-enoyl)]-*sn*-glycerol, (*S*)-**6**

The same procedure was followed as described for (*S*)-**1**, using (*R*)-**16b** (144mg, 0.24 mmol), linoleic acid (78mg, 0.28 mmol), DMAP (24mg, 0.19 mmol) and EDCI (56mg, 0.29 mmol) in dry dichloromethane (3mL). The crude product was purified by flash column chromatography using ethyl acetate:hexane (1:19) as eluent affording the product (*S*)-**6** as a colourless liquid (197mg, 95% yield). TLC (Silica, ethyl acetate:petroleum ether, 20:80): Rf = 0.76. $[\alpha]^{20}_D$ = -0.02 (c. 5.32, CH_2Cl_2). IR (NaCl, ν_{max} / cm^{-1}): 3008 (m), 2925 (vs), 2854 (vs), 1746 (vs), 1466 (s), 1378 (w), 1163 (s). ^1H NMR (400 MHz, $CDCl_3$) δ_H: 5.35 (m, 6H, =CH), 5.26 (m, 1H, CH *sn*-2), 4.29 (dd, *J*=11.9, 4.3 Hz, 2H, CH_2 *sn*-1/*sn*-3), 4.14 (dd, *J*=11.9, 6.0 Hz, 2H, CH_2 *sn*-1/*sn*-3), 2.77 (t, *J*=6.6 Hz, 2H, =CHC*H*$_2$CH=), 2.32 (t, *J*=7.6 Hz, 2H, CH_2COO SFA), 2.31 (t, *J*=7.6 Hz, 4H, CH_2COO MUFA/PUFA), 2.03 (q, *J*=6.9 Hz, 4H, C*H*$_2$CH= MUFA), 2.01 (q, *J*=6.8 Hz, 4H, C*H*$_2$CH= PUFA), 1.65–1.57 (m, 6H, C*H*$_2$CH$_2$COO), 1.38–1.23 (m, 58H, CH_2), 0.89 (t, *J*=6.9, 3H, CH$_2$C*H*$_3$ PUFA), 0.88 (t, *J*=6.9, 6H, CH$_2$C*H*$_3$) ppm. $^{13}C\{H\}$ NMR (101 MHz, $CDCl_3$) δ_C: 173.42, 173.41, 173.0, 130.4, 130.2 (2), 129.9, 128.2, 128.1, 69.0, 62.3 (2), 34.4, 34.2 (2), 32.08, 32.06, 31.7, 29.92, 29.86 (2), 29.85 (2), 29.83 (2), 29.82, 29.80 (2), 29.76, 29.68, 29.65, 29.52, 29.50, 29.47 (2), 29.45, 29.33 (2), 29.27 (2), 29.2 (2), 27.37, 27.35 (2), 27.3, 25.8, 25.1, 25.0 (2), 22.8 (2), 22.7, 14.3 (2), 14.2 ppm. $[M + Na]^+$ calcd for $C_{55}H_{100}O_6Na$ 879.7412; found, 879.7393.

*3.4. Synthesis of the SUU′ subclass category TAGs (S)-**3** and **4***

3.4.1. Synthesis of 3-dodecanoyl-*sn*-glycerol, (*R*)-**17**

Acylglycerol (*R*)-**9a** (200mg, 0.51 mmol) dissolved in dichloromethane (3mL) was added to a 10mL round bottom flask equipped with a magnetic stirrer. Water (1mL) was pipetted to the solution, which was cooled down to 0°C. Subsequently DDQ (115mg, 0.51 mmol) was added, which turned the solution to a dark green colour. The cooling bath was removed after 30min. The solution was stirred under nitrogen atmosphere overnight with the magnetic stirrer on a full speed. After that time the reaction mixture was extracted three times with dichloromethane and the combined organic layers washed with water, aqueous $NaHCO_3$ and brine. Then they were dried over Na_2SO_4, concentrated *in vacuo*, and the crude concentrate then purified by flash column chromatography with 4% boric acid impregnated silica gel using a gradient solvent system from ethyl acetate:hexane (1:9) to ethyl acetate:hexane (1:1) as eluent affording the product (*R*)-**17** as a white waxy solid (139mg, 72% yield). TLC (Silica, ethyl acetate:petroleum ether, 50:50): Rf = 0.18. Mp. 53.8-54.3°C. $[\alpha]^{20}_D$ = -1.62 (c. 1.11, CH_2Cl_2). IR (ATR, ν_{max} / cm^{-1}): 3300 (br), 2956 (m), 2918 (vs), 2849 (vs), 1733 (s), 1463 (m), 1175 (s). ^1H NMR (400 MHz, $CDCl_3$) δ_H: δ 4.21 (dd, *J*=11.8, 4.6 Hz, 1H, CH_2 *sn*-3), 4.15 (dd, *J*=11.6, 6.1 Hz, 1H, CH_2 *sn*-3), 3.93 (m, 1H, CH *sn*-2), 3.70 (ddd, *J*=10.4, 6.4, 4.1 Hz, 1H, CH_2 *sn*-1), 3.60 (dd, *J*=11.4, 5.6 Hz, 1H, CH_2 *sn*-1), 2.51 (d, *J*=5.1 Hz, 1H, CHO*H*), 2.35 (t, *J*=6.1, 2H, C*H*$_2$COO), 2.07 (t, *J*=6.1 Hz, 1H, CH$_2$O*H*),1.64 (p, *J*=7.6 Hz, 2H, C*H*$_2$CH$_2$COO), 1.32–1.22 (m, 16H, CH_2), 0.88 (t, *J*=6.9, 3H, CH$_2$C*H*$_3$) ppm.

$^{13}C\{H\}$ NMR (101 MHz, $CDCl_3$) δ_C: 174.5, 70.4, 65.3, 63.5, 34.3, 32.1, 29.7 (2), 29.6, 29.5, 29.4, 29.3, 25.1, 22.8, 14.3 ppm. $[M + Na]^+$ calcd for $C_{15}H_{30}O_4Na$ 297.2036; found, 297.2023.

3.4.2. Synthesis of [2-(*p*-methoxyphenyl)-1,3-dioxolan-4-yl]methyl dodecanoate, (*R*)-**18**

Acylglycerol (*R*)-**9a** (100mg, 0.25 mmol) was added to a 10mL round bottom flask in dichloromethane (3mL) equipped with a magnetic stirrer. Water (0.5mL) was pipetted to the solution, and it was cooled down to 0°C. Subsequently DDQ (75mg, 0.33 mmol) was added, which turned the solution to a dark green colour. The mixture was stirred under nitrogen for 30 min and then the cooling bath was removed. It was allowed to stir for additional five hours at room temperature, during which the colour slowly changed to bright red. When the dark colour had vanished, the reaction was finished, and it was extracted three times with dichloromethane. The combined organic layers were washed with aqueous $NaHCO_3$, water and brine. Then they were dried over Na_2SO_4, concentrated *in vacuo*, and the crude concentrate then purified by flash column chromatography with 4% boric acid impregnated silica gel using ethyl acetate:petroleum ether (1:9) as eluent affording the product (*R*)-**18** as a colourless liquid (38mg, 54% yield). TLC (Silica, ethyl acetate:petroleum ether, 50:50): Rf = 0.71. $[\alpha]^{20}_D$ = +5.63 (c. 1.92, CH_2Cl_2). IR (ATR, ν_{max} / cm^{-1}): 2923 (s), 2853 (s), 1737 (s), 1614 (w), 1464 (m), 1248 (vs), 1160 (s), 1032 (s). ^1H NMR (400 MHz, $CDCl_3$) δ_H: 7.40 (dd, *J*=8.7, 6.2 Hz, 2H, Ph-H), 6.90 (d, *J*=7.8 Hz, 2H, Ph-H), 5.89 (s, 0.45H, OCH-Ph), 5.78 (s, 0.55H, OCH-Ph), 4.48 (m, 0.45H, CH *sn*-2), 4.43 (m, 0.55H, CH *sn*-2), 4.28 (dd, *J*=6.8, 1.8 Hz, 0.5H, CH_2 *sn*-1), 4.23 (m, 2H, CH_2 *sn*-3), 4.10 (dd, *J*=8.5, 7.1 Hz, 0.5H, CH_2 *sn*-1), 3.95 (dd, *J*=8.4, 5.1 Hz, 0.5H, CH_2 *sn*-1), 3.81 (s, 3H, OCH_3), 3.77 (dd, *J*=8.5, 6.8 Hz, 0.5H, CH_2 *sn*-1), 2.35 (m, 2H, CH_2COO), 1.63 (p, *J*=7.2 Hz, 2H, CH_2CH_2COO), 1.33-1.24 (m, 16H, CH_2), 0.88 (t, *J*=6.9 Hz, 3H, CH_2CH_3) ppm. $^{13}C\{H\}$ NMR (101 MHz, $CDCl_3$) δ_C: 173.82, 173.79, 160.8, 160.6, 129.7, 129.2, 128.3 (2), 128.1 (2), 113.93 (2), 113.91 (2), 104.8, 100.0, 74.2, 74.0, 67.6, 67.4, 64.6, 64.2, 55. 5 (2), 34.3 (2), 32.1 (2), 29.7 (4), 29.6 (2), 29.5 (2), 29.4 (2), 29.3 (2), 25.1 (2), 22.8 (2), 14.3 (2) ppm. $[M + Na]^+$ calcd for $C_{23}H_{36}O_5Na$ 415.2455; found, 415.2445.

3.4.3. Conversion of acetal (*R*)-**18** into (*R*)-**17**

Acetal (*R*)-**18** (32mg, 0.08 mmol) in acetonitrile (1mL) was added to a 10 mL round bottom flask equipped with a magnetic stirrer. Subsequently, elemental iodine (6mg, 0.02 mmol) and water (20μL) were added to the solution, and it allowed to stir for 22h at room temperature under nitrogen atmosphere. After that time, the solution was quenched with $Na_2S_2O_3$ (20% w/w aqueous solution) and extracted three times with ethyl acetate. The combined organic layers were dried over Na_2SO_4 and concentrated *in vacuo*. The crude concentrate was then purified by flash column chromatography with 4% boric acid impregnated silica gel using gradient solvent system from ethyl acetate:hexane (1:9) to ethyl acetate:hexane (1:1) as eluent. That afforded the product (*R*)-**17** as a slightly yellow solid (20mg, 91% yield).

3.4.4. Synthesis of 1,2-*O*-isopropylidene-3-*O*-(*p*-methoxybenzyl)-*sn*-glycerol, (*S*)-**7**

The same procedure was followed as described for (*R*)-**7**, using (*S*)-solketal (900mg, 6.81 mmol), PMB-Cl (900mg, 6.81 mmol) and sodium hydride (60% mineral oil dispersion, 490mg, 20.43 mmol) in dry THF (15mL). The crude product was purified by flash column chromatography using ethyl acetate:petroleum ether (2:8) as eluent affording the product (*S*)-**7** as a colourless liquid (1308mg, 76% yield). TLC (Silica, ethyl acetate:petroleum ether, 3:7): Rf = 0.55. $[\alpha]^{20}_D$ = +1.27 (c. 2.96, CH_2Cl_2). IR (ATR, ν_{max} / cm^{-1}): 2934 (s,), 2861 (s), 1612 (m), 1244 (vs), 1034 (s). ^1H NMR (400 MHz, $CDCl_3$) δ_H: 7.26 (d, *J*=8.8 Hz, 2H, Ph-H), 6.88 (d, *J*=8.8 Hz, 2H, Ph-H), 4.49 (m, 2H, $PhCH_2$), 4.28 (p, *J*=6.0 Hz, 1H, CH *sn*-2), 4.04 (dd, *J*=8.3, 6.4 Hz, 1H, CH_2 *sn*-1), 3.80 (s, 3H, OCH_3), 3.72 (dd, *J*=8.3, 6.4 Hz, 1H, CH_2 *sn*-1), 3.52 (dd, *J*=9.8, 5.7 Hz, 1H, CH_2 *sn*-3), 3.44 (dd, *J*=9.9, 5.6 Hz, 1H, CH_2 *sn*-3), 1.42 (s, 3H, $C(CH_3)_2$), 1.36 (s, 3H, $C(CH_3)_2$) ppm. $^{13}C\{H\}$ NMR (101 MHz, $CDCl_3$) δ_C: 159.4, 130.2, 129.5 (2), 113.9 (2), 109.5, 74.9, 73.3, 71.6, 67.1, 55.4 (OCH_3), 26.9, 25.5 ppm. HRMS (ESI) *m*/*z*: $[M + Na]^+$ calcd for $C_{14}H_{20}O_4Na$ 275.1254; found, 275.1251.

3.4.5. Synthesis of 3-*O*-(*p*-methoxybenzyl)-*sn*-glycerol, (*R*)-**8**

The same procedure was followed as described for (*S*)-**8**, using (*S*)-**7** (1255mg, 4.97 mmol) and elemental iodine (379mg, 1.49 mmol) in acetonitrile (25mL) and water (1.5mL). The crude product was purified by flash column chromatography using ethyl acetate:petroleum ether (3:7) as eluent and then gradually increasing the proportion of ethyl acetate until the eluent was pure ethyl acetate. This afforded the product (*R*)-**8** as a slightly yellow solid (968mg, 92% yield). TLC (Silica, ethyl acetate:petroleum ether, 30:70): Rf = 0.13. Mp. 42.8-43.7°C. $[\alpha]^{20}_{D}$ = -1.84 (c. 2.06, CH_2Cl_2). IR (ATR, ν_{max} / cm^{-1}): 3268 (br), 2940 (s), 2839 (vs), 1611 (m), 1511 (m), 1242 (s), 1021 (vs). ^{1}H NMR (400 MHz, $CDCl_3$) δ_H: 7.24 (d, *J*=8.9 Hz, 2H, Ph-H), 6.88 (d, *J*=8.7 Hz, 2H, Ph-H), 4.47 (s, 2H, $PhCH_2$), 4.87 (m, 1H, CH *sn*-2), 3.80 (s, 3H, OCH_3), 3.68 (dd, *J*=11.4, 3.8 Hz, 1H, CH_2 *sn*-1), 3.60 (dd, *J*=11.4, 5.6 Hz, 1H, CH_2 *sn*-1), 3.53 (dd, *J*=9.6, 4.1 Hz, 1H, CH_2 *sn*-3), 3.49 (dd, *J*=9.7, 6.2 Hz, 1H, CH_2 *sn*-3), 2.91 (br s, 1H, OH), 2.48 (br s, 1H, OH) ppm. $^{13}C\{H\}$ NMR (101 MHz, $CDCl_3$) δ_C: 159.5, 129.9, 129.6 (2), 114.0 (2), 73.4, 71.6, 70.7, 64.2, 55.4 ppm. $[M + Na]^+$ calcd for $C_{11}H_{16}O_4Na$ 235.0941; found, 235.0935.

3.4.6. Synthesis of 3-*O*-(*p*-methoxybenzyl)-1-[(9Z)-octadec-9-enoyl)]-*sn*-glycerol, (*S*)-**14**

The same procedure was followed as described for (*R*)-**14**, using (*R*)-**8** (820mg, 3.87 mmol), oleic acid acetoxime ester **13** (1435mg, 4.25 mmol), and immobilized *Candida antarctica* lipase B (CAL-B) (338mg, 15% w/w). The crude product was purified by flash column chromatography with 4% boric acid impregnated silica gel using ethyl acetate/hexane (2:8) as eluent affording the product (*S*)-**14** as a colourless liquid (1592mg, 74% yield). TLC (Silica, ethyl acetate:petroleum ether, 30:70): Rf = 0.53. $[\alpha]^{20}_{D}$ = +0.79° (c. 1.65, CH_2Cl_2). IR (ATR, ν_{max} / cm^{-1}): 3448 (br), 2923 (s), 2853 (vs), 1736 (vs), 1612 (m), 1464 (m), 1247 (s). ^{1}H NMR (400 MHz, $CDCl_3$) δ_H: 7.25 (d, *J*=9.3 Hz, 2H, Ph-H), 6.88 (d, *J*=8.8 Hz, 2H, Ph-H), 5.34 (m, 2H, =CH), 4.49 (s, 2H, $PhCH_2$), 4.17 (dd, *J*=11.5, 4.4 Hz, 1H, CH_2 *sn*-1), 4.12 (dd, *J*=11.6, 6.1 Hz, 1H, CH_2 *sn*-1), 4.01 (m, 1H, CH *sn*-2), 3.81 (s, 3H, OCH_3), 3.52 (dd, *J*=9.6, 4.3 Hz, 1H, CH_2 *sn*-3), 3.46 (dd, *J*=9.6, 6.1 Hz, 1H, CH_2 *sn*-3), 2.49 (br s, 1H, OH), 2.32 (t, *J*=7.6 Hz, 2H, CH_2COO), 2.00 (m, 4H, CH_2CH=), 1.61 (m, 2H, CH_2CH_2COO), 1.34-1.25 (m, 20H, CH_2), 0.87 (t, *J*=6.7 Hz, 3H, CH_2CH_3) ppm. $^{13}C\{H\}$ NMR (101 MHz, $CDCl_3$) δ_C: 174.1, 159.5, 130.2, 129.89, 129.87, 129.6 (2), 114.0 (2), 73.3, 70.7, 69.1, 65.5, 55.4, 34.3, 32.0, 29.9, 29.8, 29.7, 29.5 (2), 29.3, 29.2 (2), 27.4, 27.3, 25.0, 22.8, 14.3 ppm. $[M + Na]^+$ calcd for $C_{29}H_{48}O_5Na$ 499.3394; found, 499.3392.

3.4.7. Synthesis of 1-[(9Z)-octadec-9-enoyl)]-*sn*-glycerol, (*S*)-**19**

The same procedure was followed as described for (*R*)-**17**, using (*S*)-**14** (1568mg, 3.29 mmol) and DDQ (747mg, 3.29 mmol) in dichloromethane (8mL) and water (2mL). The crude product was purified by flash column chromatography with 4% boric acid impregnated silica gel using ethyl acetate:hexane (1:9) as eluent affording the product (*S*)-**19** as a colourless liquid (816mg, 70% yield). TLC (Silica, ethyl acetate:petroleum ether, 20:80): Rf = 0.16. $[\alpha]^{20}_{D}$ = +1.05° (c. 2.48, CH_2Cl_2). IR (ATR, ν_{max} / cm^{-1}): 3419 (br), 3004 (s), 2956 (vs), 2854 (vs), 17339(s), 1463 (m), 1175 (s). ^{1}H NMR (400 MHz, $CDCl_3$) δ_H: 5.34 (m, 2H, =CH), 4.21 (dd, *J*=11.6, 4.6 Hz, 1H, CH_2 *sn*-1), 4.14 (dd, *J*=11.6, 6.1 Hz, 1H, CH_2 *sn*-1), 3.92 (m, 1H, CH *sn*-2), 3.69 (dd, *J*=10.5, 4.0 Hz, 1H, CH_2 *sn*-3), 3.60 (dd, *J*=11.4, 5.8 Hz, 1H, CH_2 *sn*-3), 2.51 (s, 1H, CHO*H*), 2.35 (t, *J*=7.6, 2H, CH_2COO), 2.07 (s, 1H, CH_2OH), 2.01 (q, *J*=5.8, 4H, CH_2CH=),1.63 (p, *J*=7.4 Hz, 2H, CH_2CH_2COO), 1.32–1.26 (m, 16H, CH_2), 0.88 (t, *J*=6.8, 3H, CH_2CH_3) ppm. $^{13}C\{H\}$ NMR (101 MHz, $CDCl_3$) δ_C: 174.5, 130.2, 129.9, 70.4, 65.3, 63.5, 34.3, 32.1, 29.9, 29.8, 29.7, 29.5 (2), 29.3, 29.2 (2), 27.4, 27.3, 25.0, 22.8, 14.3 ppm. $[M + Na]^+$ calcd for $C_{21}H_{40}O_4Na$ 356.2819; found, 356.2807.

3.4.8. Synthesis of 3-dodecanoyl-1-[(9Z)-octadec-9-enoyl)]-*sn*-glycerol, (*S*)-**20a**

The same procedure was followed as described for (*R*)-**9a**, using (*S*)-**19** (150mg, 0.42 mmol), vinyl laurate (142mg, 0.63 mmol) and the CAL-B (44mg, 15% w/w) in dichloromethane (4mL). The mixture was stirred for 3h under nitrogen. The crude product was purified by flash column chromatography with 4%

boric acid impregnated silica gel using ethyl acetate:petroleum ether (1:9) as eluent affording the product (*S*)-**20a** as a soft white wax (188mg, 83% yield). TLC (Silica, ethyl acetate:petroleum ether, 20:80): Rf = 0.36. Mp. 22.0-24.0°C. $[\alpha]^{20}_{D}$ = -1.28 (c. 4.42, CH_2Cl_2). IR (ATR, ν_{max} / cm^{-1}): 3492 (br), 2954 (), 2916 (vs), 2849 (s), 1730 (vs), 1470 (m), 1377 (m), 1182 (s). ^{1}H NMR (400 MHz, $CDCl_3$) δ_H: 5.34 (m, 2H, =CH), 4.16 (qd, J = 11.2, 4.9 Hz, 4H, CH_2 *sn*-1/*sn*-3), 4.08 (m, 1H, CH *sn*-2), 2.34 (t, *J*=7.6 Hz, 4H, CH_2COO), 2.00 (m, 4H, C*H*$_2$CH=), 1.63 (p, *J*=7.4 Hz, 4H, C*H*$_2$CH$_2$COO), 1.34–1.25 (m, 36H, CH_2), 0.88 (t, *J*=6.9, 6H, CH$_2$C*H*$_3$) ppm. $^{13}C\{H\}$ NMR (101 MHz, $CDCl_3$) δ_C: 173.9, 173.6, 130.2, 129.8, 72.3, 62.1, 61.7, 34.4, 34.2, 32.07, 32.05, 29.92, 29.85, 29.83, 29.80 (2), 29.77, 29.7, 29.6, 29.51, 29.47, 29.4, 29.32, 29.26, 29.2 (2), 27.4, 27.3, 25.1, 25.0, 22.8 (2), 14.3 (2) ppm. $[M + Na]^+$ calcd for $C_{33}H_{62}O_5Na$ 561.4489; found, 561.4482.

3.4.9. Synthesis of 3-hexadecanoyl-1-[(9*Z*)-octadec-9-enoyl)]-*sn*-glycerol, (*S*)-**20b**

The same procedure was followed as described for (*R*)-**9a**, using (*S*)-**19** (200mg, 0.56 mmol), vinyl palmitate (190mg, 0.67 mmol) and the CAL-B (59mg, 15% w/w) in dichloromethane (5mL). The mixture was stirred for 3h under nitrogen. The crude product was purified by flash column chromatography with 4% boric acid impregnated silica gel using ethyl acetate:petroleum ether (1:9) as eluent affording the product (*S*)-**20b** as a white waxy solid (263mg, 87% yield). TLC (Silica, ethyl acetate:petroleum ether, 20:80): Rf = 0.38. Mp. 42.6-43.3°C. $[\alpha]^{20}_{D}$ = -0.76 (c. 2.11, CH_2Cl_2). IR (ATR, ν_{max} / cm^{-1}): 3493 (br), 2954 (s), 2914 (vs), 2849 (s), 1731 (vs), 1471 (m), 1181 (s). ^{1}H NMR (400 MHz, $CDCl_3$) δ_H: 5.34 (m, 2H, =CH), 4.16 (m, 4H, CH_2 *sn*-1/*sn*-3), 4.08 (m, 1H, CH *sn*-2), 2.46 (br s, 1H, OH), 2.34 (t, *J*=7.6 Hz, 4H, CH_2COO), 2.02 (q, *J*=6.3 Hz, 4H, C*H*$_2$CH=), 1.63 (p, *J*=7.1 Hz, 4H, C*H*$_2$CH$_2$COO), 1.34–1.24 (m, 44H, CH_2), 0.88 (t, *J*=6.8, 6H, CH$_2$C*H*$_3$) ppm. $^{13}C\{H\}$ NMR (101 MHz, $CDCl_3$) δ_C: 174.09, 174.05, 130.2, 129.9, 68.6, 65.2 (2), 34.3, 34.2, 32.07, 32.05, 29.91, 29.84 (4), 29.80 (2), 29.75, 29.7, 29.6, 29.51, 29.47 (2), 29.4, 29.30, 29.27, 29.2 (2), 27.4, 27.3, 25.04, 25.03, 22.8 (2), 14.3 (2) ppm. $[M + Na]^+$ calcd for $C_{37}H_{70}O_5Na$ 617.5115; found, 617.5110.

3.4.10. Synthesis of 3-dodecanoyl-2-[(9*Z*,12*Z*)-octadeca-9,12-dienoyl)]-1-[(9*Z*)-octadec-9-enoyl)]-*sn*-glycerol, (*S*)-**3**

The same procedure was followed as described for (*S*)-**1**, using (*S*)-**20a** (124mg, 0.23 mmol), linoleic acid (74mg, 0.26 mmol), DMAP (23mg, 0.18 mmol) and EDCI (53mg, 0.28 mmol) in dry dichloromethane (3mL). The crude product was purified by flash column chromatography using ethyl acetate:hexane (1:19) as eluent affording the product (*S*)-**3** as a colourless liquid (179mg, 97% yield). TLC (Silica, ethyl acetate:petroleum ether, 20:80): Rf = 0.75. $[\alpha]^{20}_{D}$ = +0.08 (c. 1.24, CH_2Cl_2). IR (ATR, ν_{max} / cm^{-1}): 3008 (m), 2923 (vs), 2853 (s), 1741 (vs), 1464 (m), 1158 (s). ^{1}H NMR (400 MHz, $CDCl_3$) δ_H: δ 5.35 (m, 6H, =CH), 5.26 (m, 1H, CH *sn*-2), 4.29 (dd, *J*=11.9, 4.3 Hz, 2H, CH_2 *sn*-1/*sn*-3), 4.14 (dd, *J*=11.9, 6.0 Hz, 2H, CH_2 *sn*-1/*sn*-3), 2.77 (t, *J*=6.6 Hz, 2H, =CHC*H*$_2$CH=) 2.32 (t, *J*=7.6 Hz, 2H, CH_2COO PUFA), 2.31 (t, *J*=7.6 Hz, 4H, CH_2COO MUFA SFA), 2.03 (m, 8H, C*H*$_2$CH=), 1.64-1.58 (m, 6H, C*H*$_2$CH$_2$COO), 1.37–1.23 (m, 52H, CH_2), 0.89 (t, *J*=6.7, 3H, CH$_2$C*H*$_3$ PUFA), 0.88 (t, *J*=6.8, 6H, CH$_2$C*H*$_3$) ppm. $^{13}C\{H\}$ NMR (101 MHz, $CDCl_3$) δ_C: 173.5, 173.42, 173.0, 130.4, 130.2, 130.1, 129.9, 128.2, 128.0, 69.0, 62.2 (2), 35.4, 34.4, 34.3, 34.20, 34.18, 32.1 (2), 31.7, 29.92, 29.86, 29.8 (2), 29.71, 29.67, 29.64, 29.62, 29.49 (2), 29.47, 29.44, 29.42, 29.35, 29.33, 29.29, 29.27 (2), 29.24, 29.20, 27.37, 27.35, 27.3, 25.8, 25.0, 24.4, 22.8 (2), 22.7, 14.3 (2), 14.2 ppm. $[M + Na]^+$ calcd for $C_{51}H_{92}O_6Na$ 823.6786; found, 823.6772.

3.4.11. Synthesis of 3-hexadecanoyl-2-[(9*Z*,12*Z*)-octadeca-9,12-dienoyl)]-1-[(9*Z*)-octadec-9-enoyl)]-*sn*-glycerol, (*S*)-**4**

The same procedure was followed as described for (*S*)-**1**, using (*S*)-**20b** (150mg, 0.25 mmol), linoleic acid (81mg, 0.29 mmol), DMAP (25mg, 0.20 mmol) and EDCI (58mg, 0.30 mmol) in dry dichloromethane (3mL). The crude product was purified by flash column chromatography using ethyl acetate:hexane (1:19) as eluent affording the product (*S*)-**4** as a colourless liquid (207mg, 96% yield). TLC (Silica, ethyl

acetate:petroleum ether, 20:80): Rf = 0.77. $[\alpha]^{20}_{D}$ = +0.09 (c. 2.12, CH_2Cl_2). IR (ATR, ν_{max} / cm^{-1}): 3008 (m), 2922 (vs), 2853 (s), 1744 (vs), 1465 (m), 1159 (s). ^{1}H NMR (400 MHz, $CDCl_3$) δ_H: δ 5.35 (m, 6H, =CH), 5.26 (m, 1H, CH *sn*-2), 4.29 (dd, *J*=11.9, 4.3 Hz, 2H, CH_2 *sn*-1/*sn*-3), 4.14 (dd, *J*=11.9, 6.0 Hz, 2H, CH_2 *sn*-1/*sn*-3), 2.77 (t, *J*=6.5 Hz, 2H, =CHCH_2CH=) 2.32 (t, *J*=7.5 Hz, 2H, CH_2COO PUFA), 2.31 (t, *J*=7.6 Hz, 4H, CH_2COO MUFA SFA), 2.01 (m, 8H, CH_2CH=), 1.64-1.58 (m, 6H, C$H_2$$CH_2$COO), 1.33–1.24 (m, 58H, CH_2), 0.89 (t, *J*=6.9, 3H, CH_2CH_3 PUFA), 0.88 (t, *J*=6.8, 6H, CH_2CH_3) ppm. $^{13}C\{H\}$ NMR (101 MHz, $CDCl_3$) δ_C: 173.5, 173.4, 173.0, 130.4, 130.2, 130.1, 129.9, 128.2, 128.0, 69.0, 62.2, 34.3, 34.20, 34.18, 32.08, 32.06, 31.7, 29.92, 29.85 (5), 29.81 (4), 29.78 (2), 29.7, 29.6, 29.52, 29.50, 29.47 (2), 29.43, 29.35, 29.33, 29.29, 29.27 (2), 29.24, 29.20, 27.37, 27.35, 27.3, 25.8, 25.02, 24.99, 22.8 (2), 22.7, 14.3 (2), 14.2 ppm. $[M + Na]^+$ calcd for $C_{55}H_{100}O_6Na$ 879.7412; found, 879.7372.

NMR Spectra

^{1}H NMR (400 MHz, $CDCl_3$) of compound (*R*)-**7**

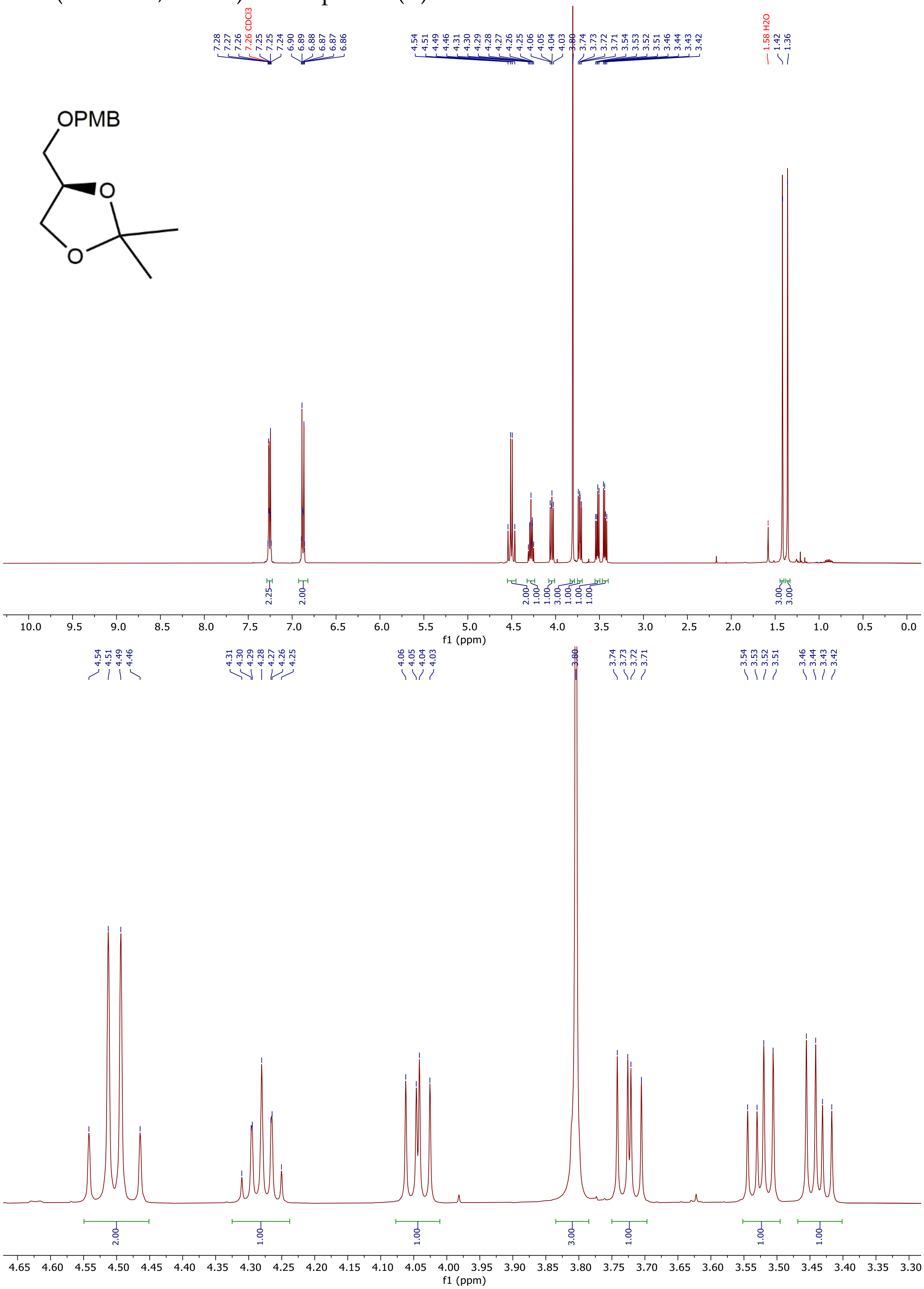

$^{13}C\{H\}$ NMR (101 MHz, $CDCl_3$) of compound (*R*)-**7**

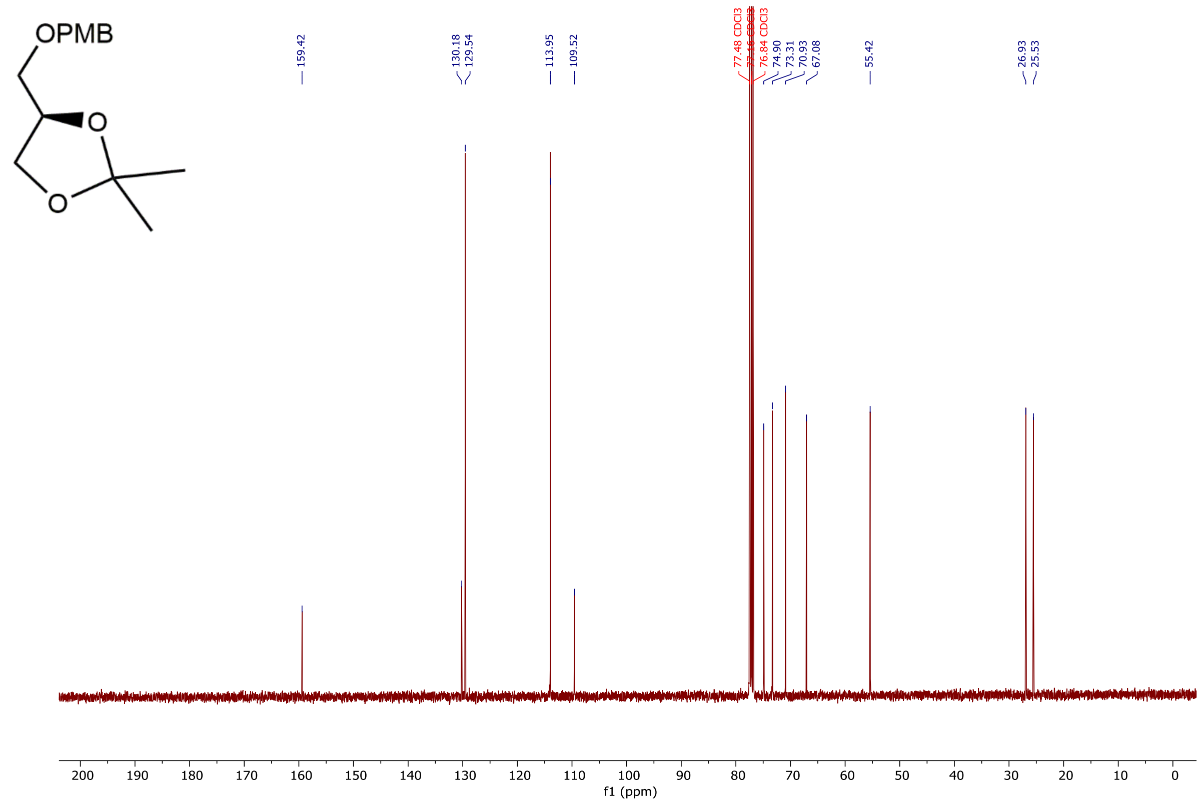

^1H-^1H COSY spectrum of compound (*R*)-**7**

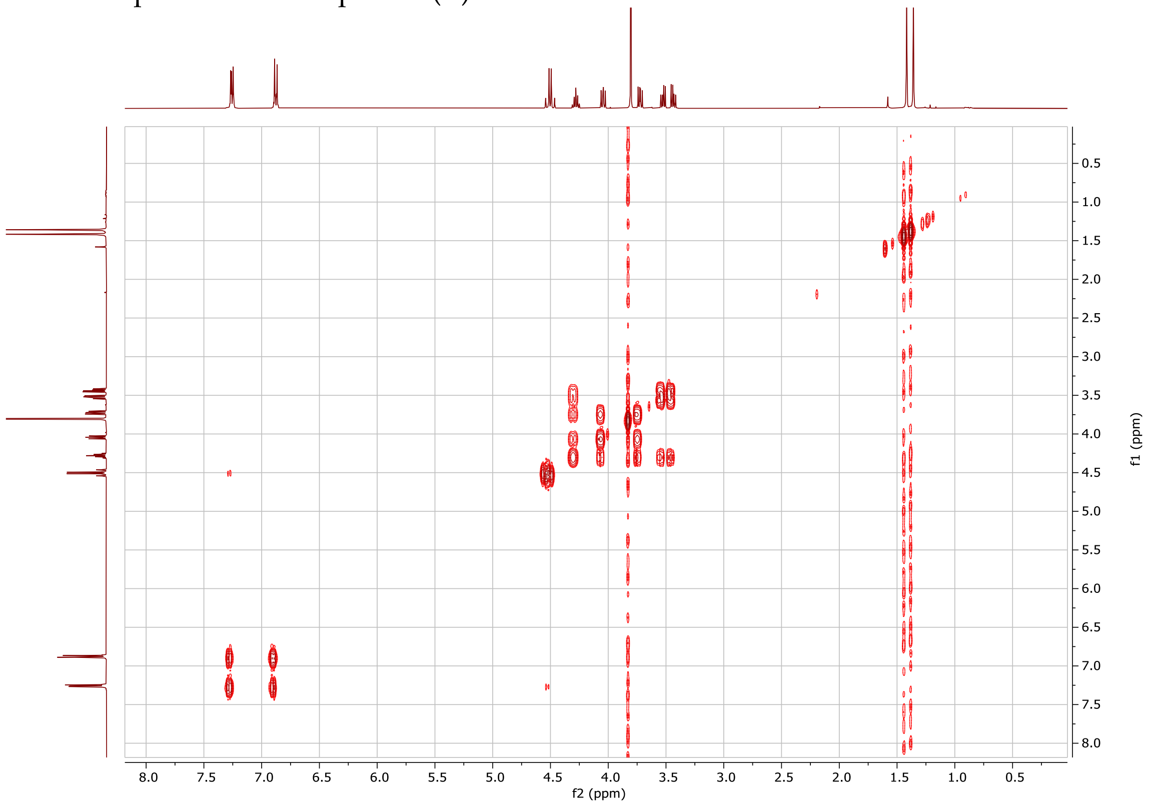

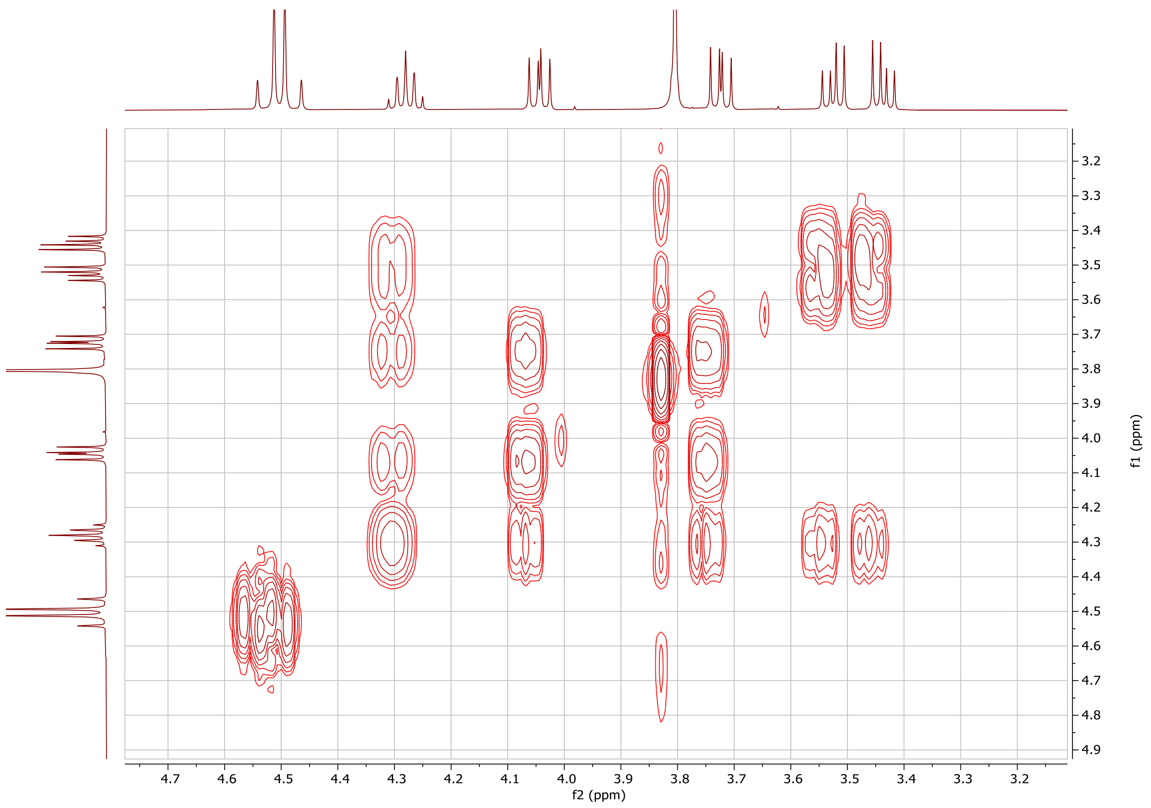

^{13}C-^{1}H HSQC spectrum of compound (*R*)-**7**

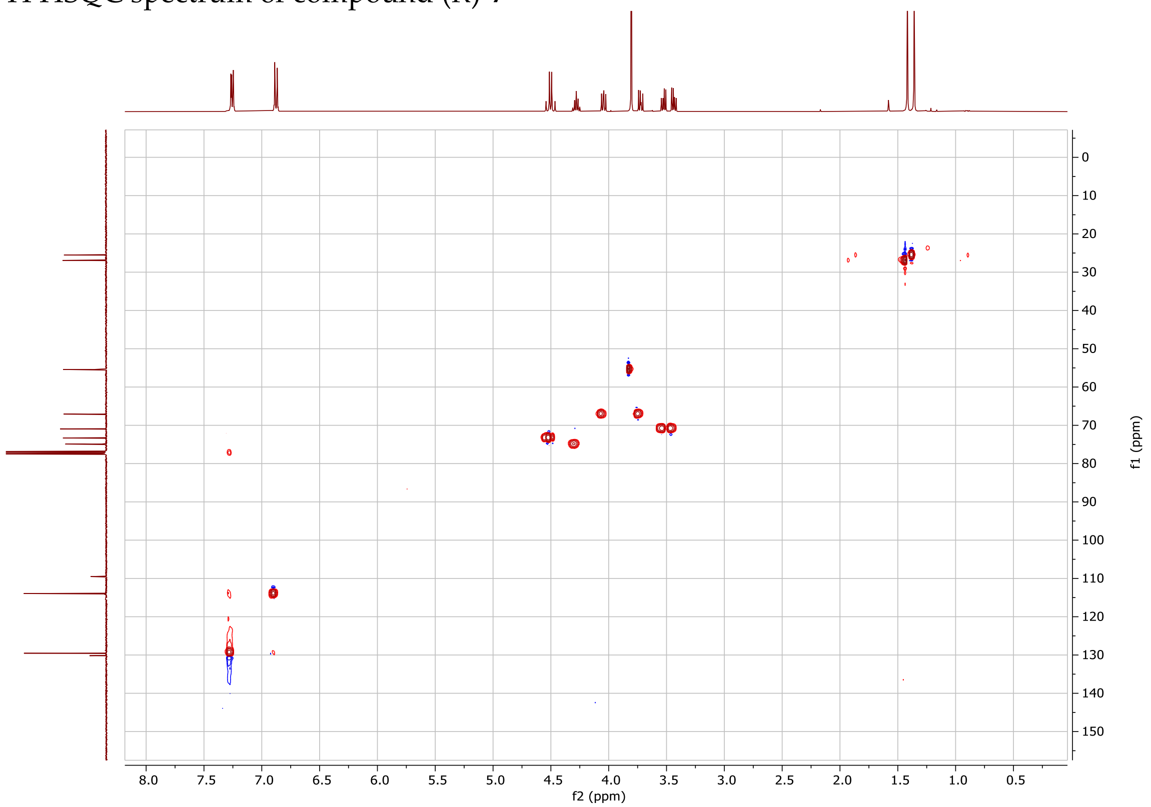

^{1}H NMR (400 MHz, $CDCl_3$) of compound (*S*)-**8**

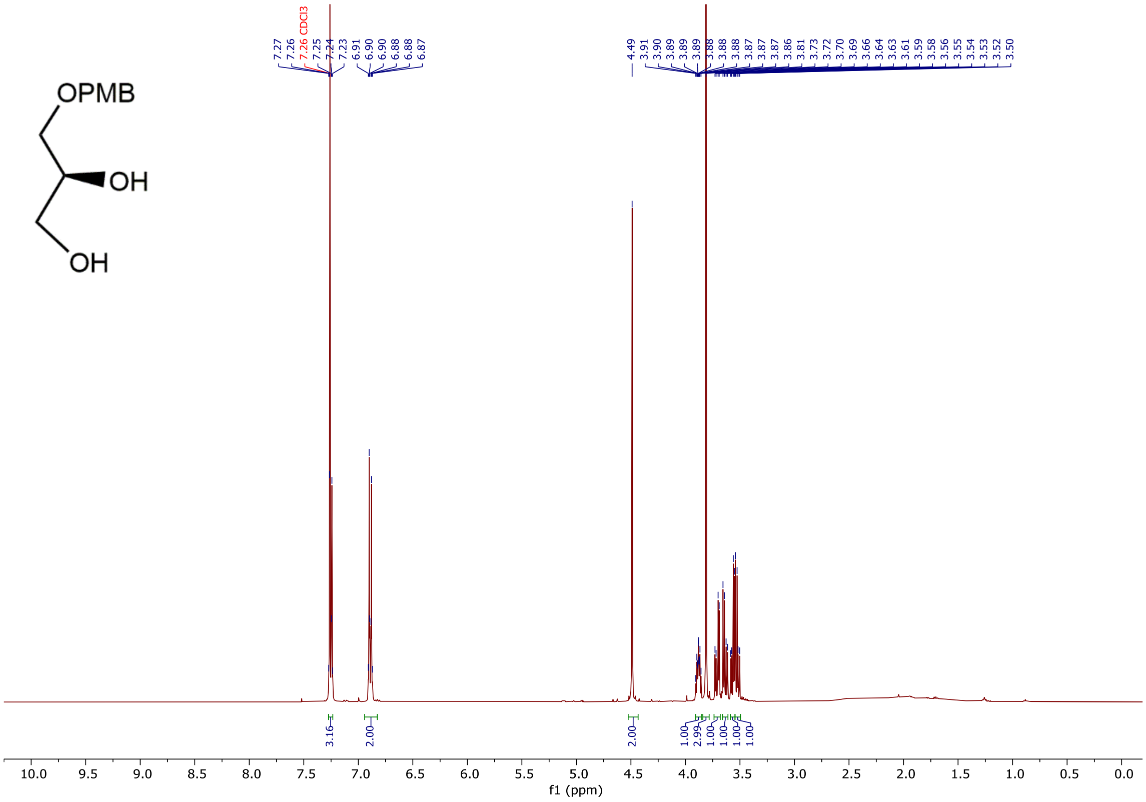

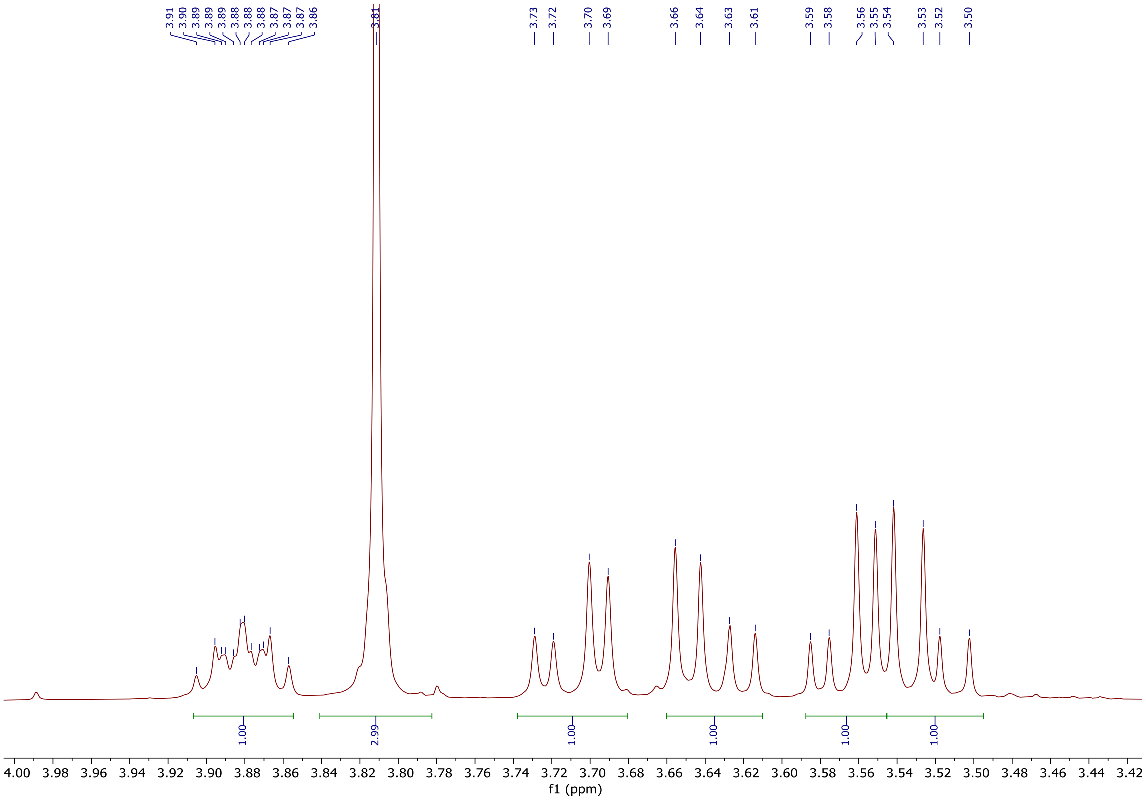

$^{13}C\{H\}$ NMR (101 MHz, $CDCl_3$) of compound (*S*)-**8**

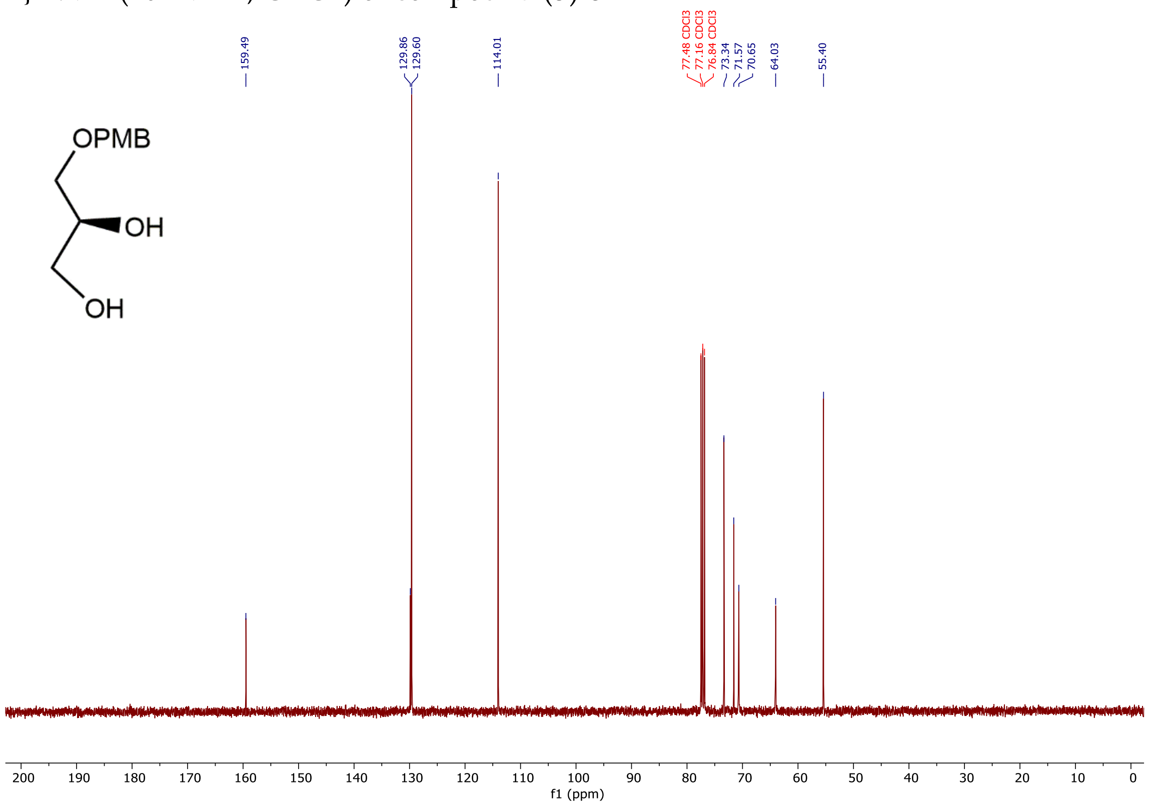

^{1}H-^{1}H COSY spectrum of compound (*S*)-**8**

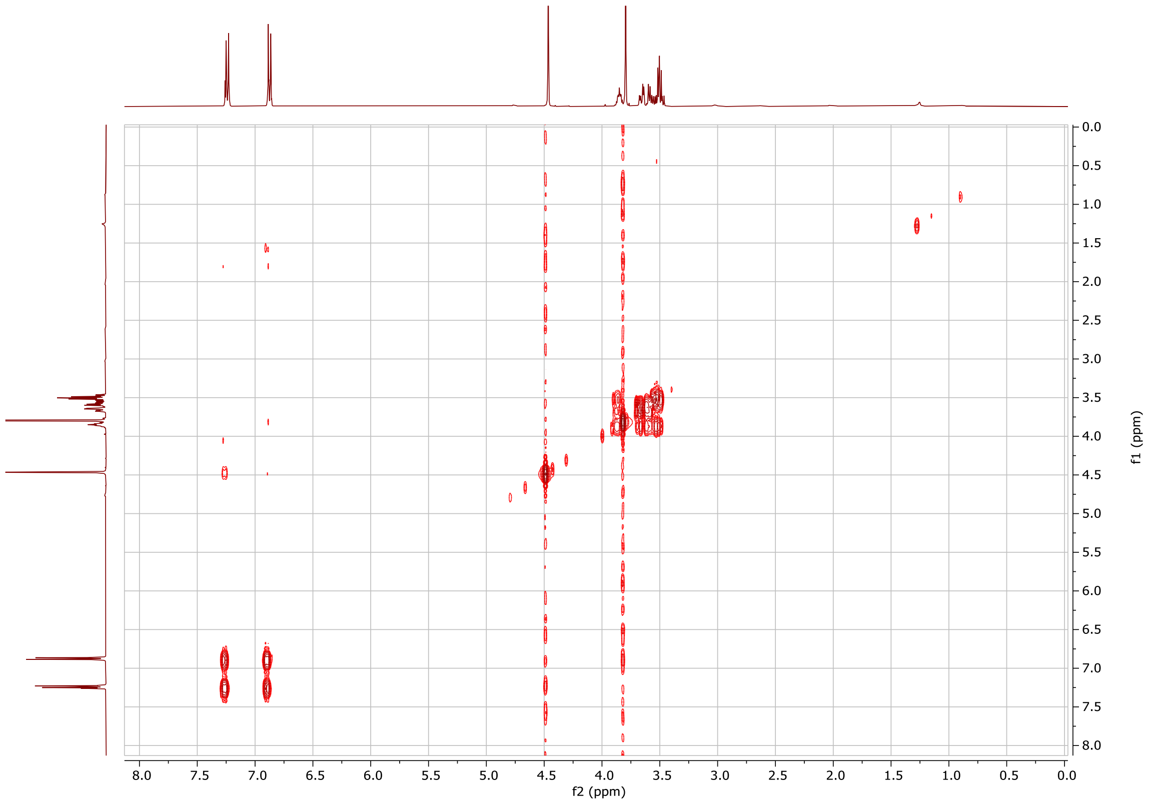

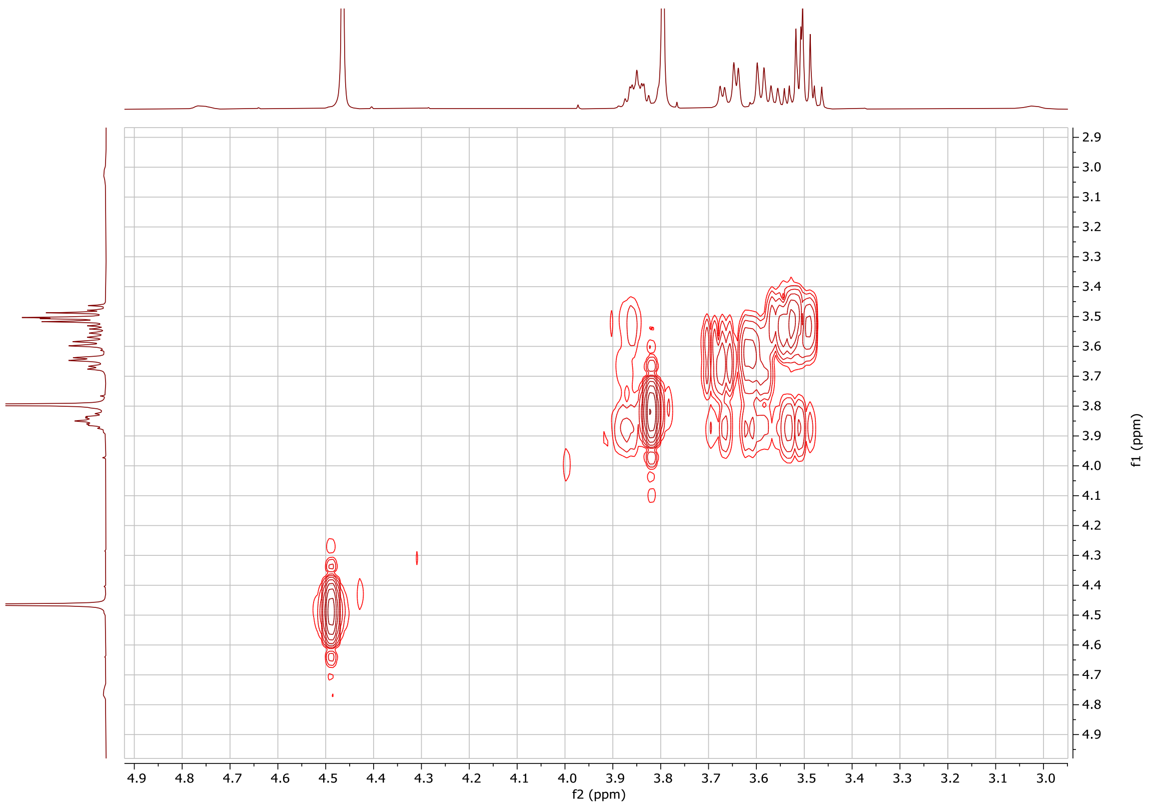

^{13}C-^{1}H HSQC spectrum of compound (*S*)-**8**

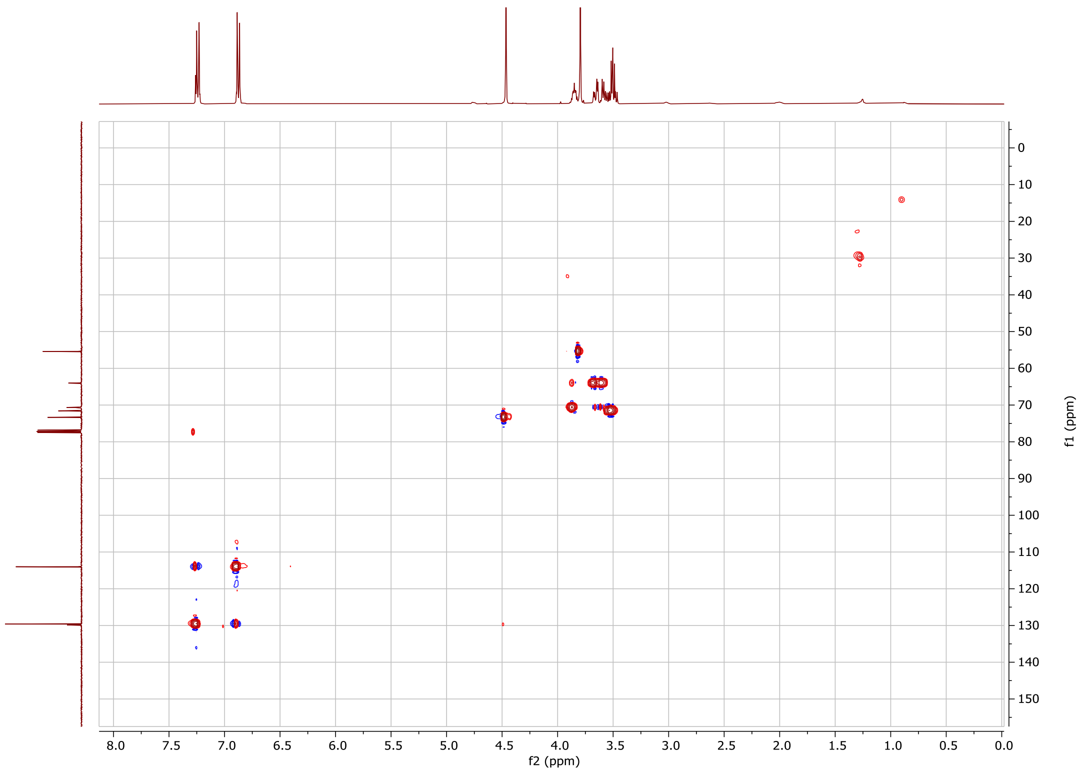

^{1}H NMR (400 MHz, $CDCl_3$) of compound (*R*)-**9a**

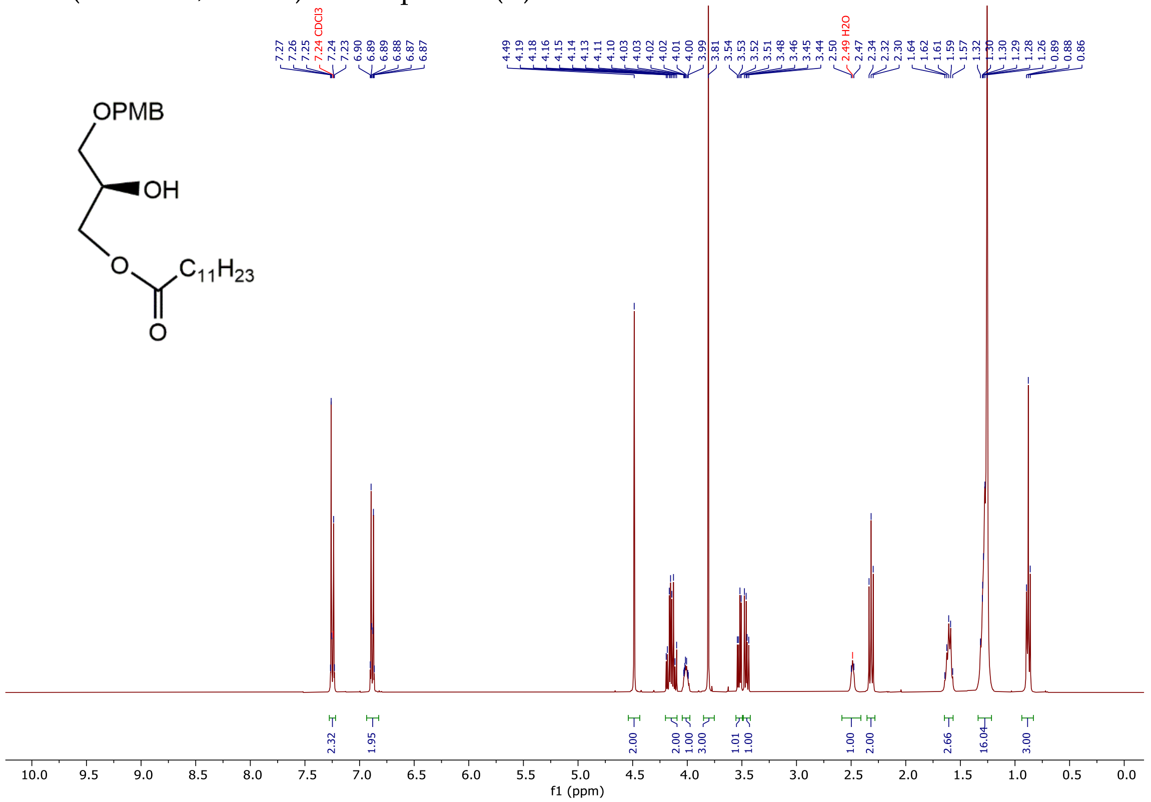

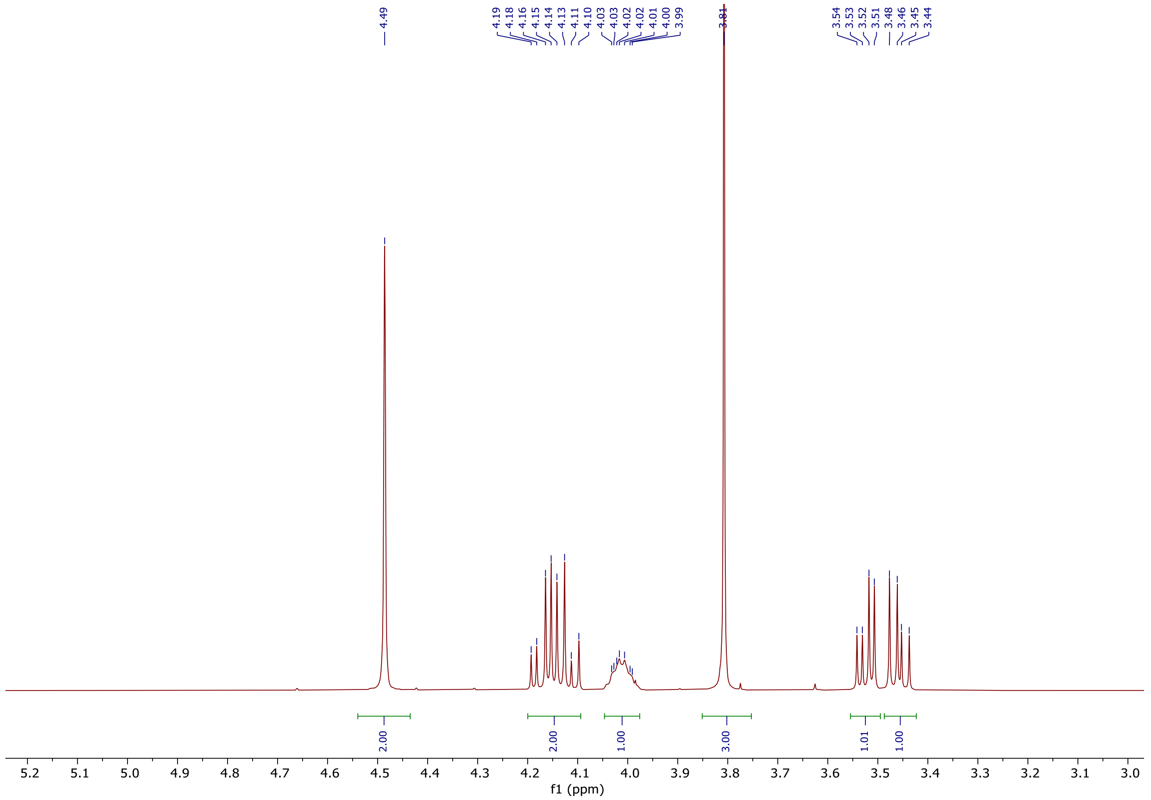

$^{13}C\{H\}$ NMR (101 MHz, $CDCl_3$) of compound (*R*)-**9a**

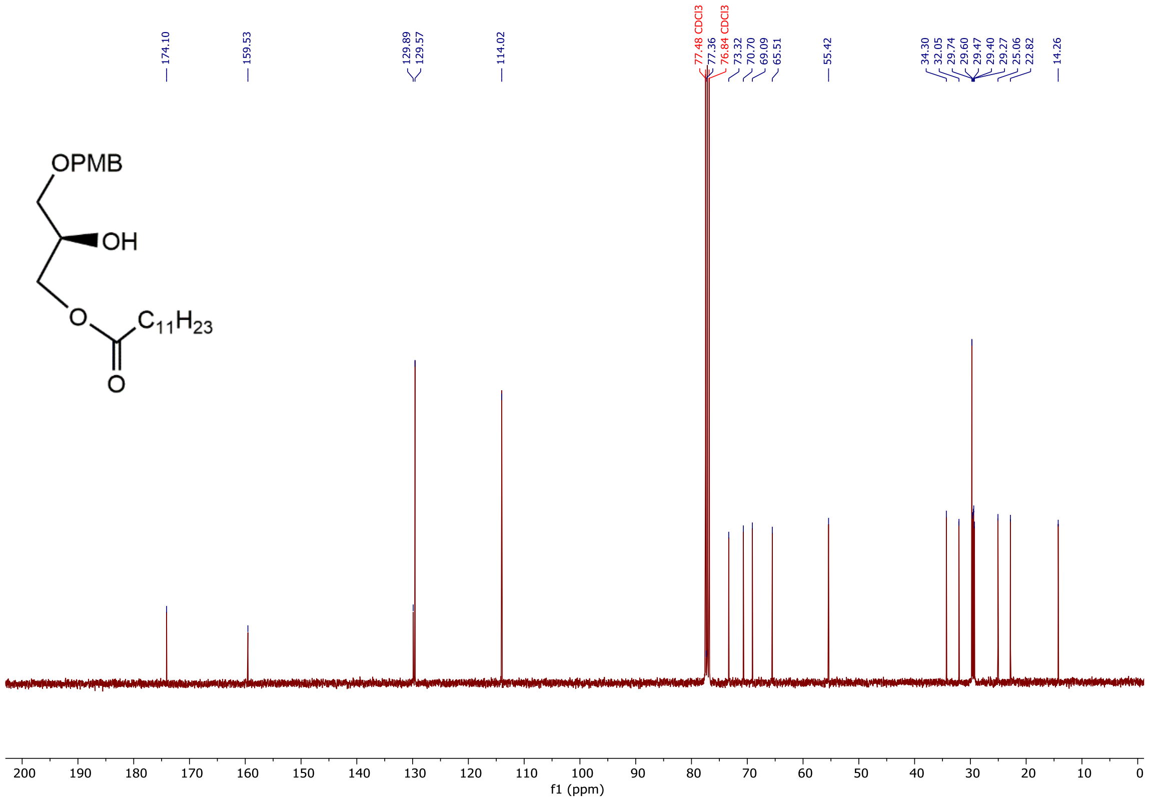

^1H-^1H COSY spectrum of compound (*R*)-**9a**

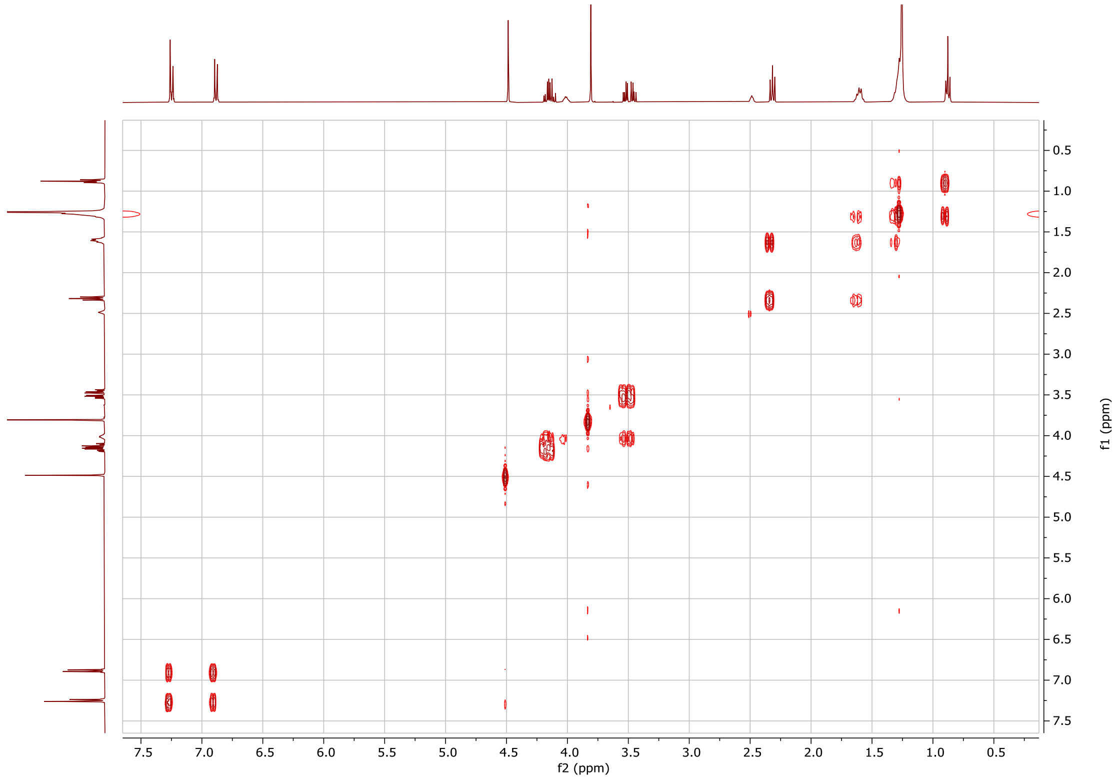

^{13}C-^{1}H HSQC spectrum of compound (*R*)-**9a**

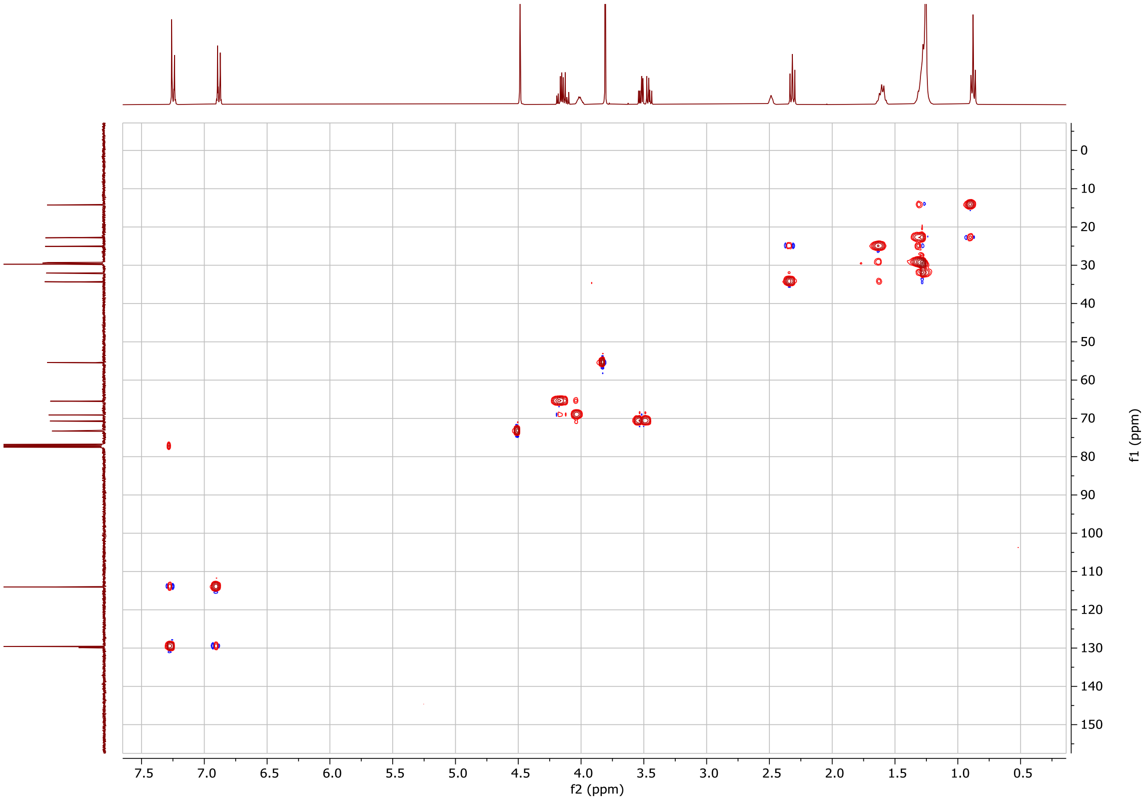

^{1}H NMR (400 MHz, $CDCl_3$) of compound (*R*)-**9b**

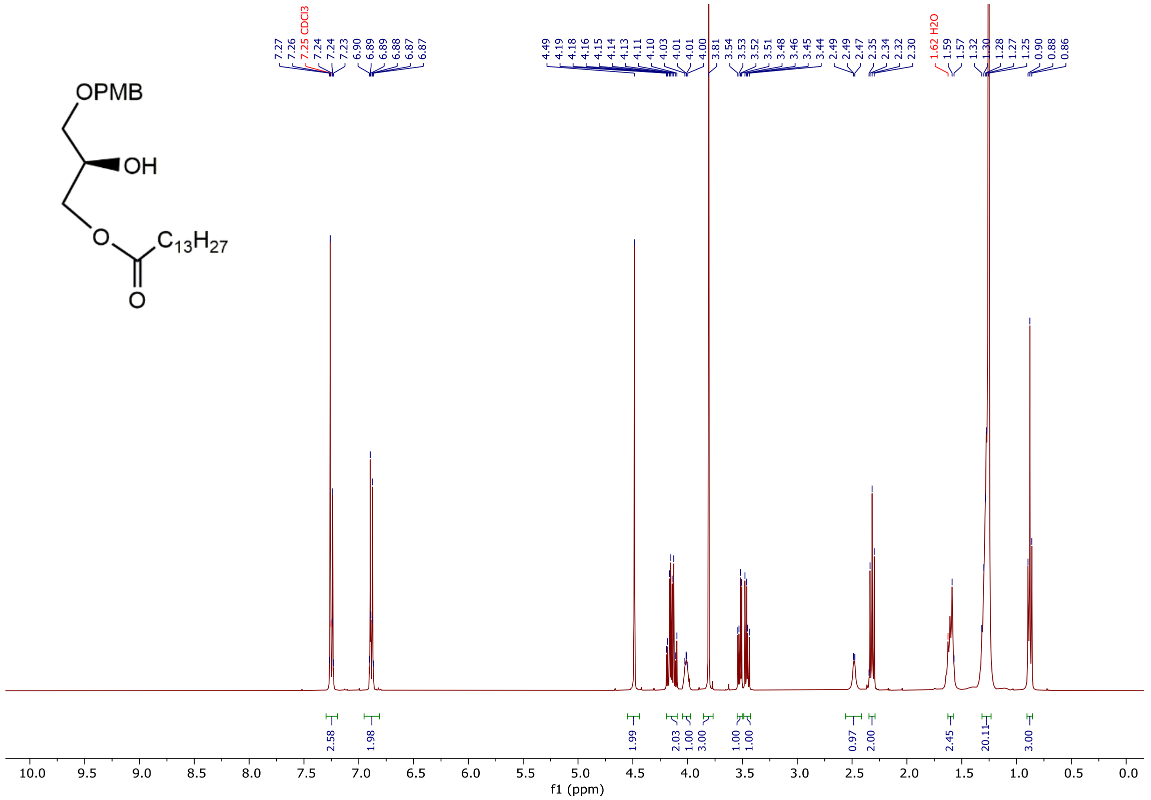

$^{13}C\{H\}$ NMR (101 MHz, $CDCl_3$) of compound (*R*)-**9b**

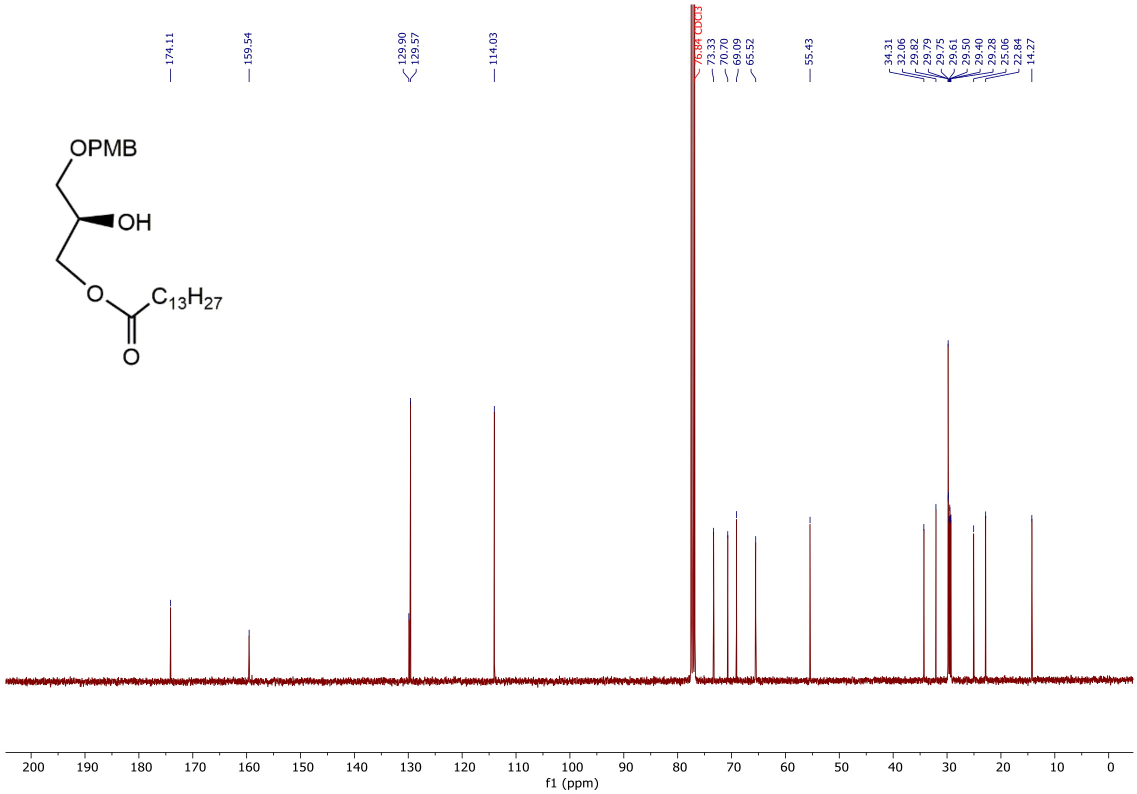

^{1}H-^{1}H COSY spectrum of compound (*R*)-**9b**

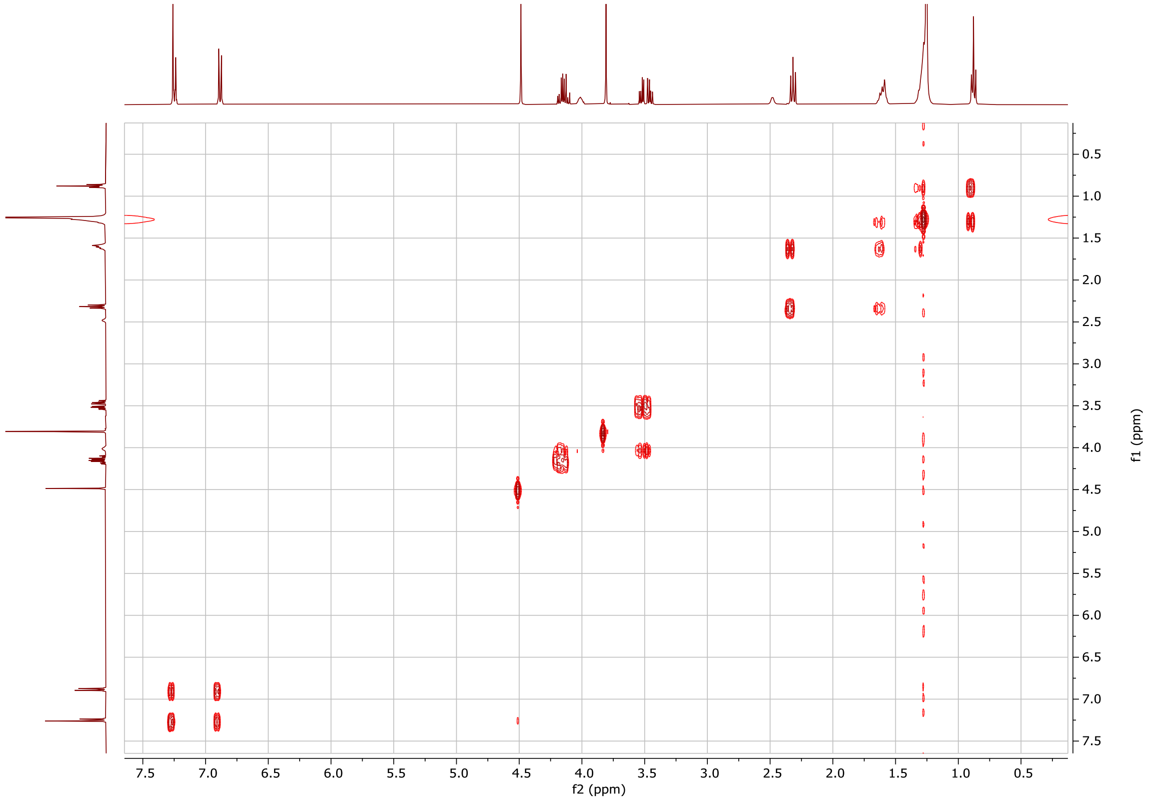

^{13}C-^{1}H HSQC spectrum of compound (*R*)-**9b**

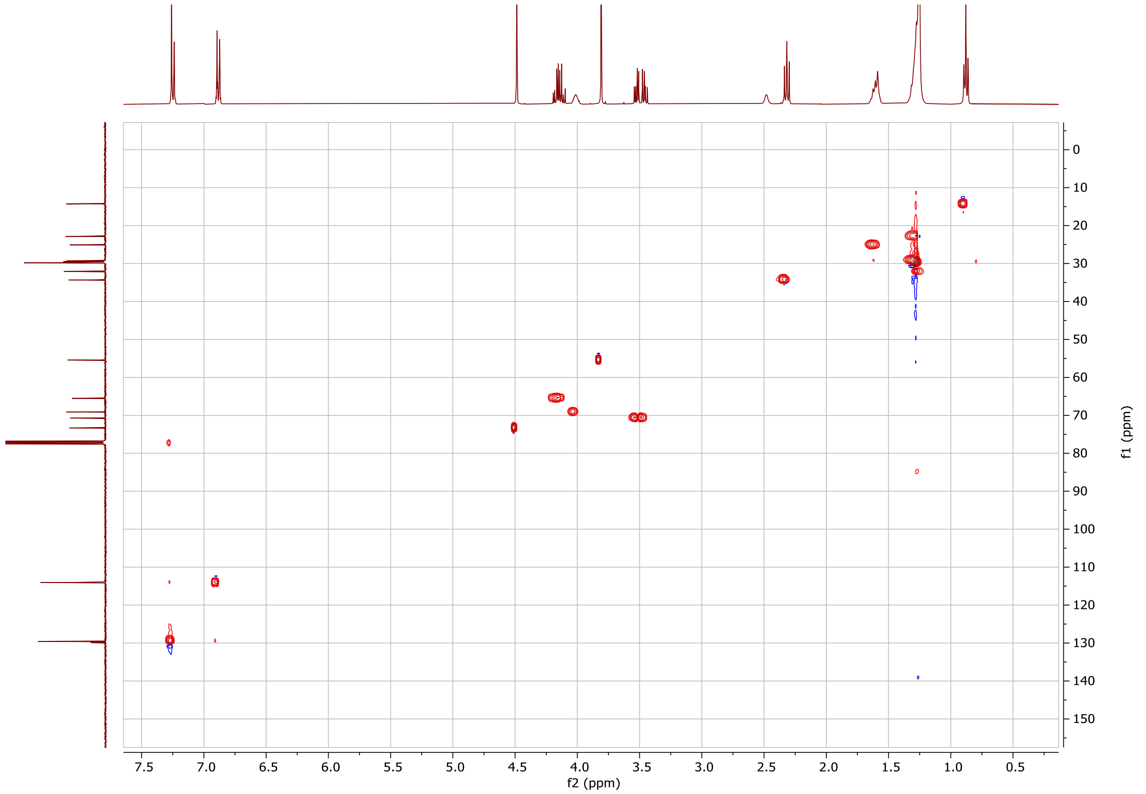

^{1}H NMR (400 MHz, $CDCl_3$) of compound (*R*)-**9c**

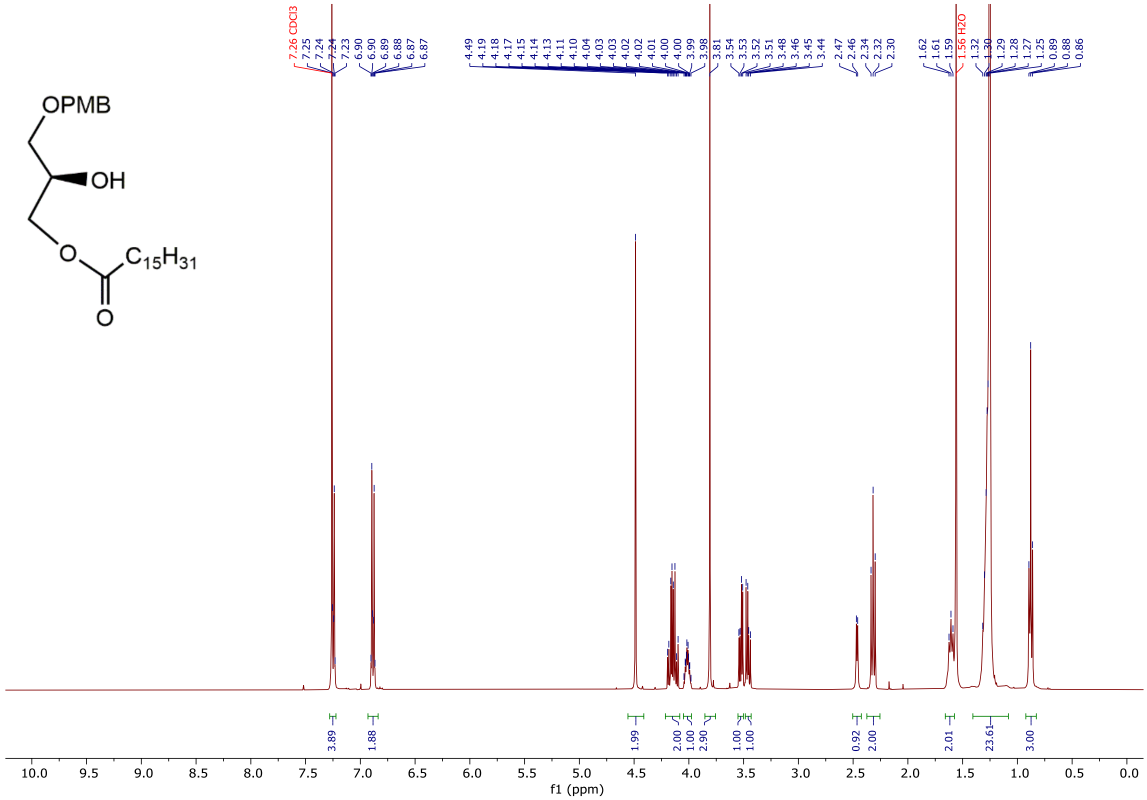

$^{13}C\{H\}$ NMR (101 MHz, $CDCl_3$) of compound (*R*)-**9c**

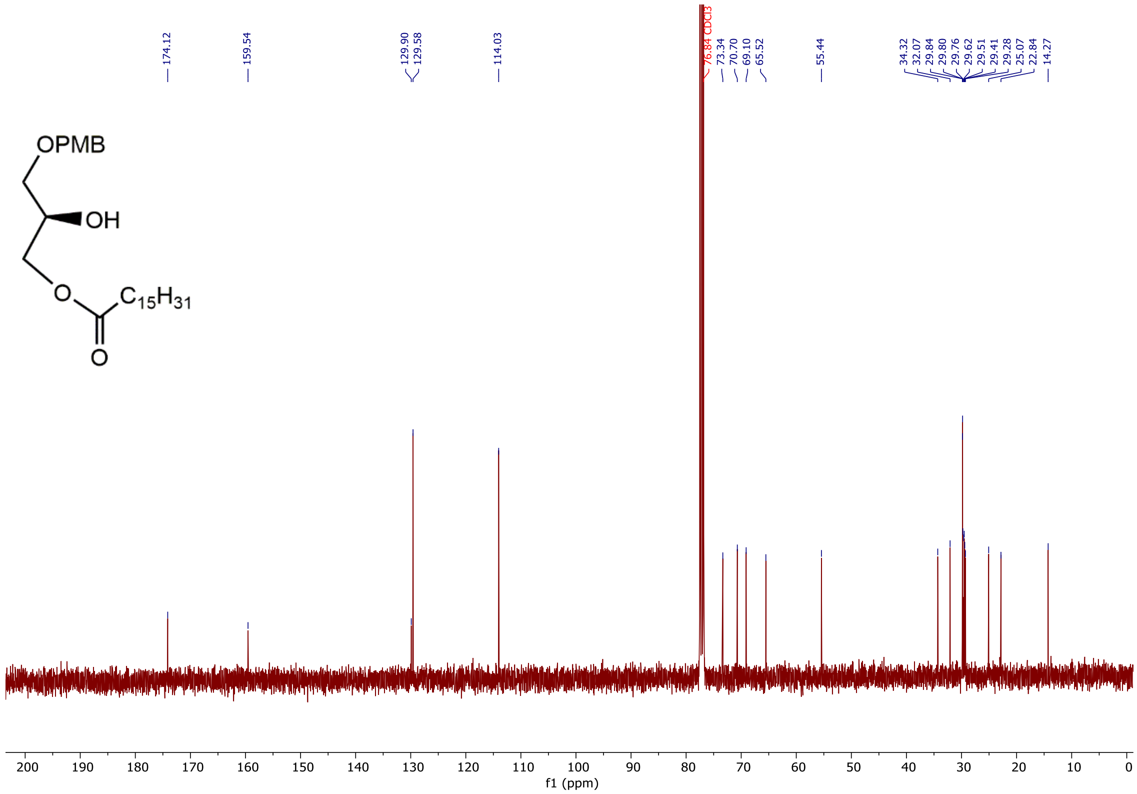

^{1}H-^{1}H COSY spectrum of compound (*R*)-**9c**

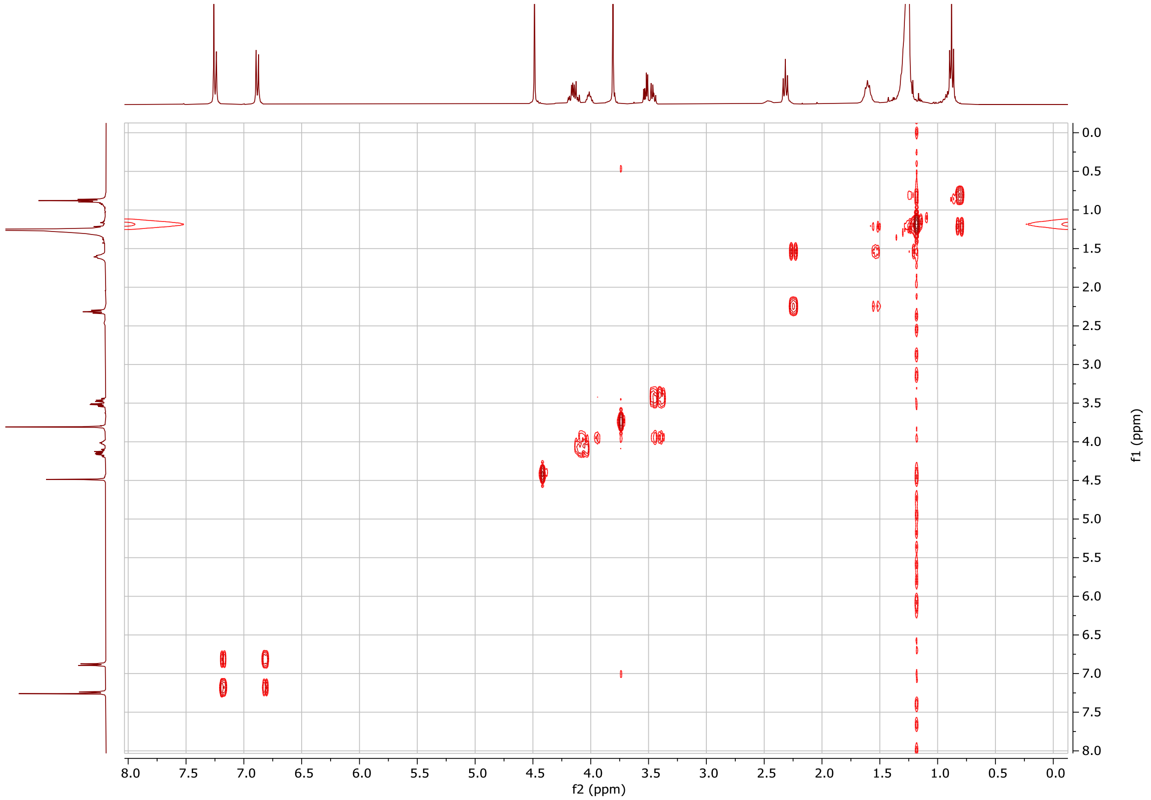

^{13}C-^{1}H HSQC spectrum of compound (*R*)-**9c**

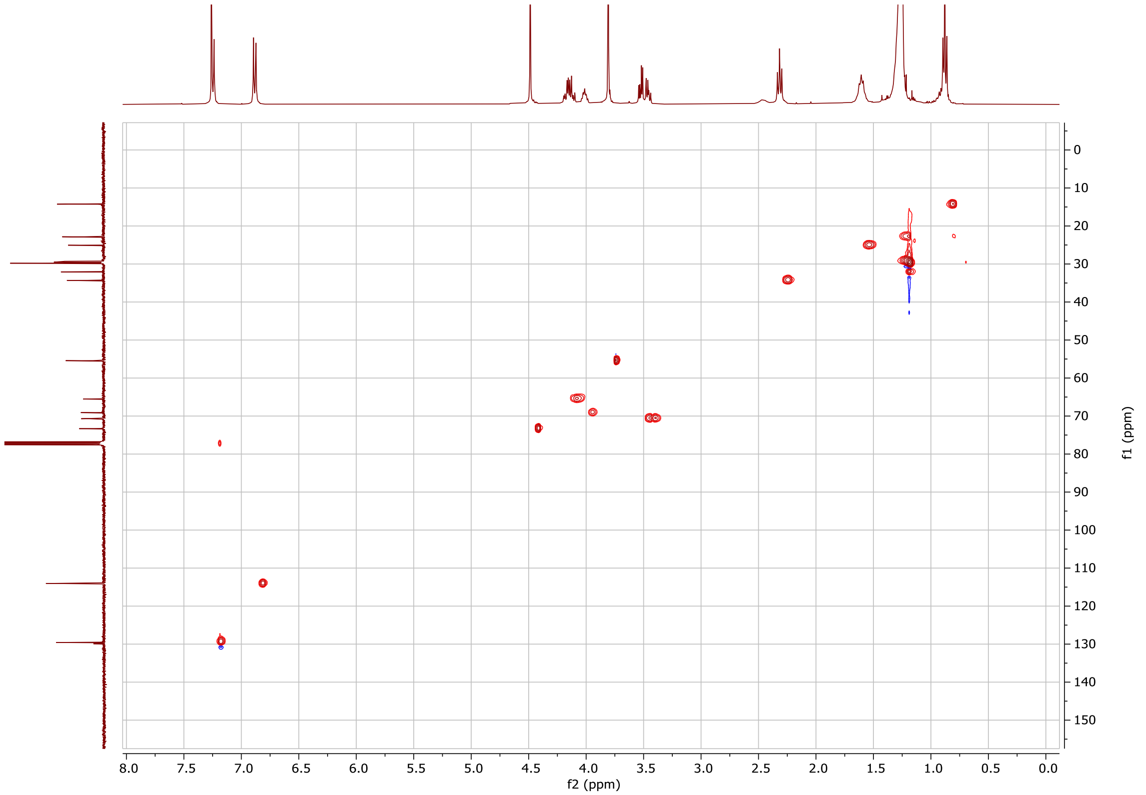

^{1}H NMR (400 MHz, $CDCl_3$) of compound (*R*)-**10a**

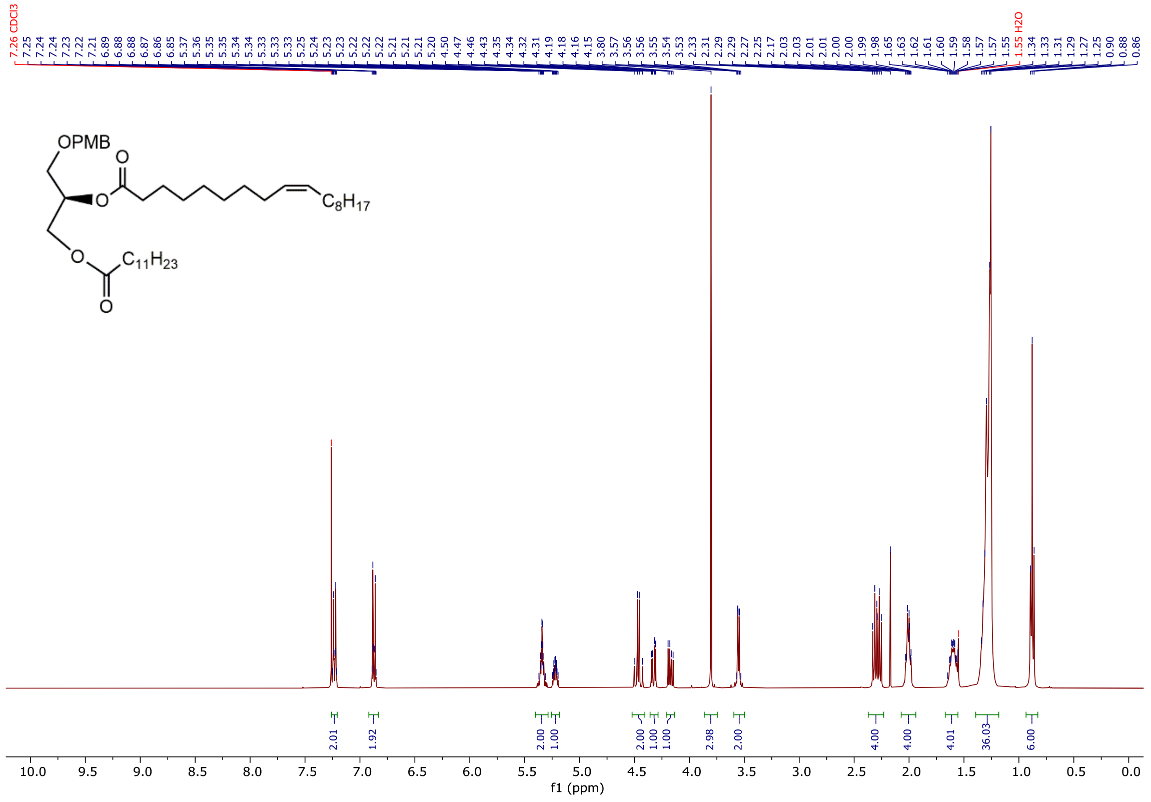

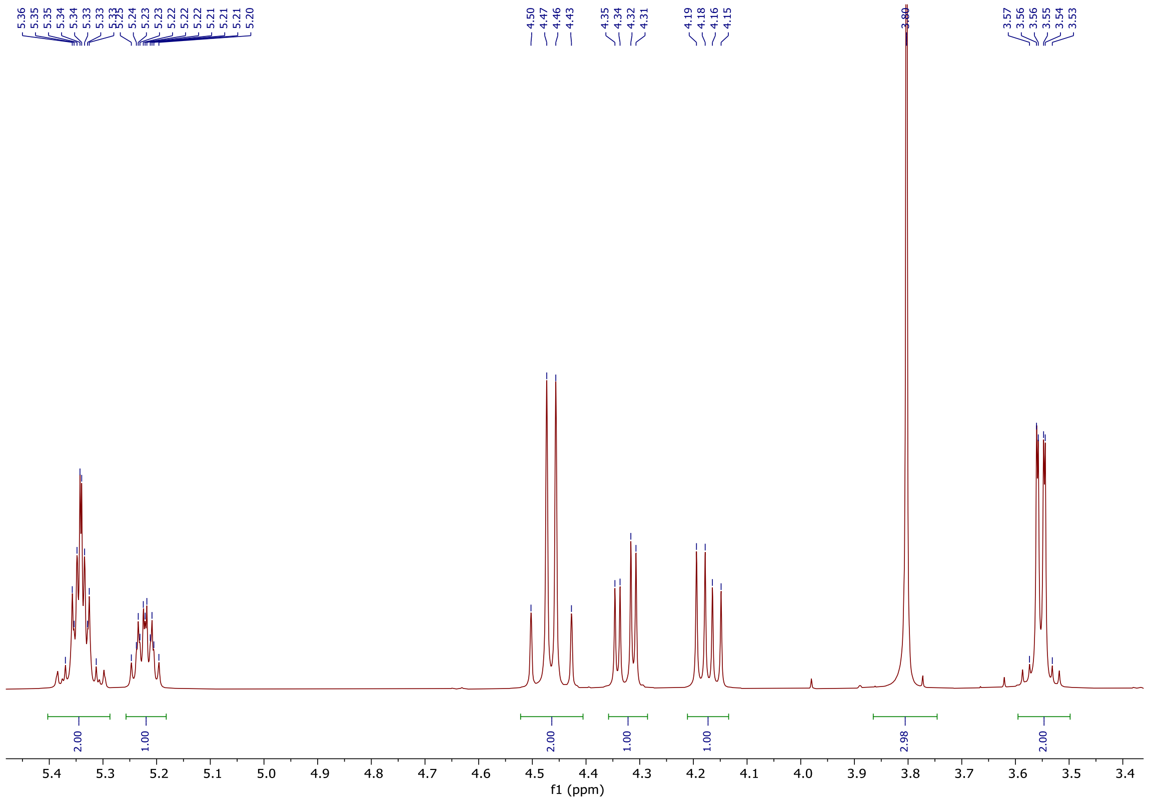

$^{13}C\{H\}$ NMR (101 MHz, $CDCl_3$) of compound (*R*)-**10a**

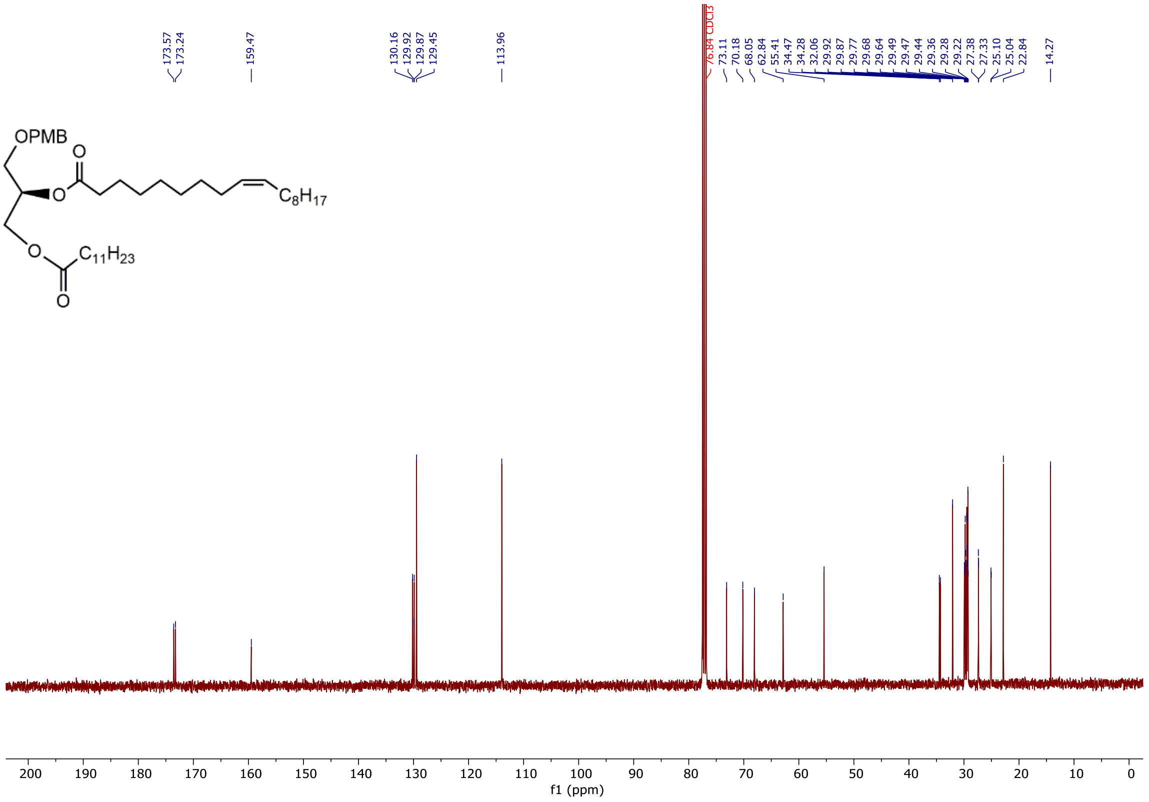

^1H-^1H COSY spectrum of compound (*R*)-**10a**

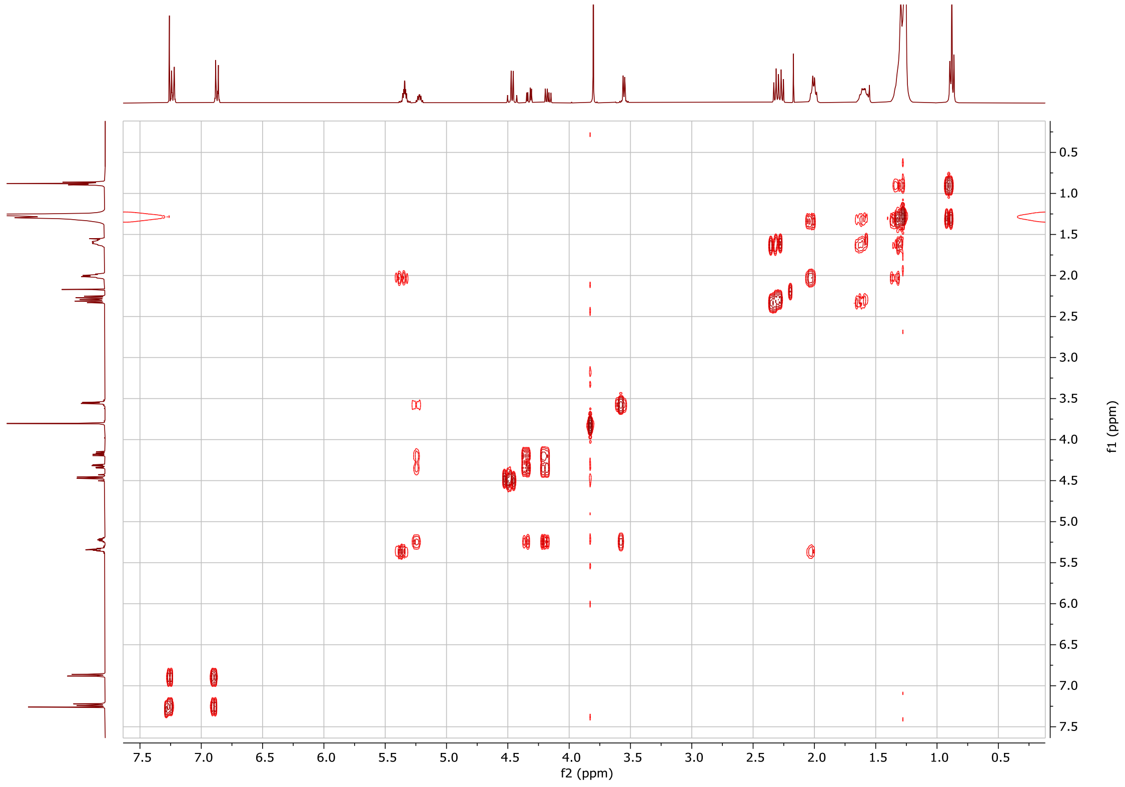

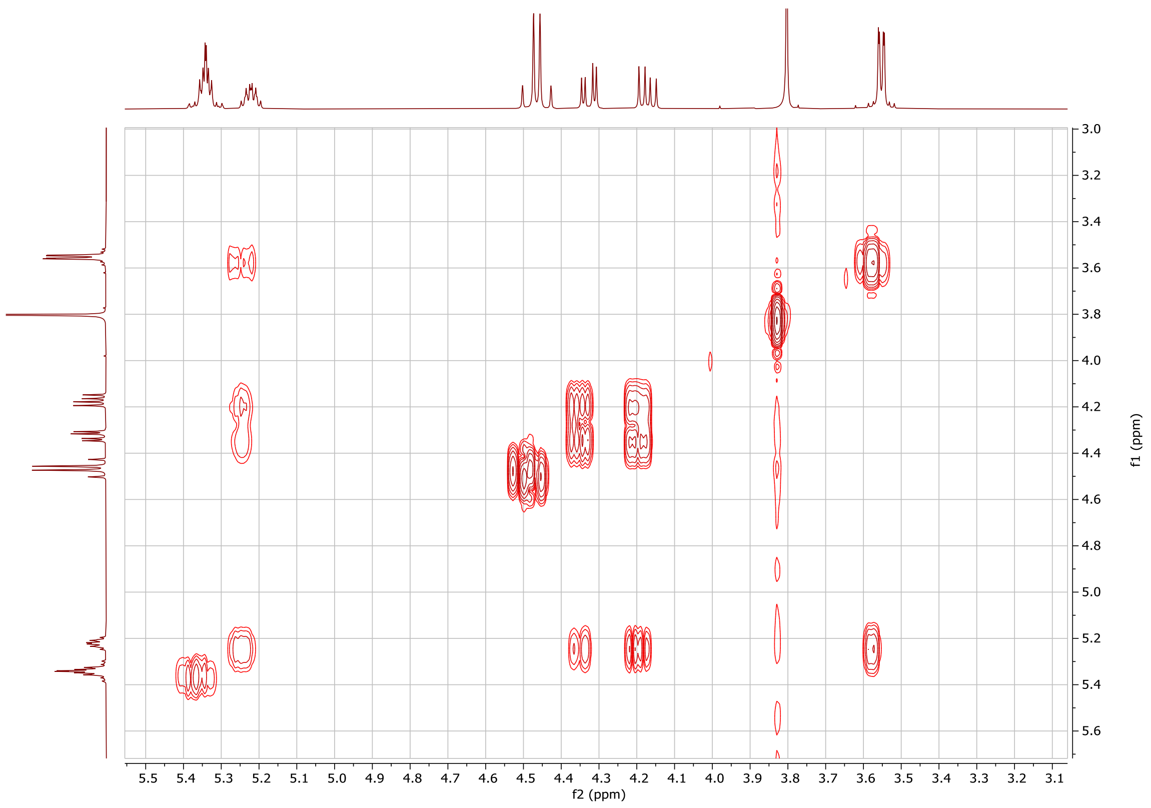

^{13}C-^{1}H HSQC spectrum of compound (*R*)-**10a**

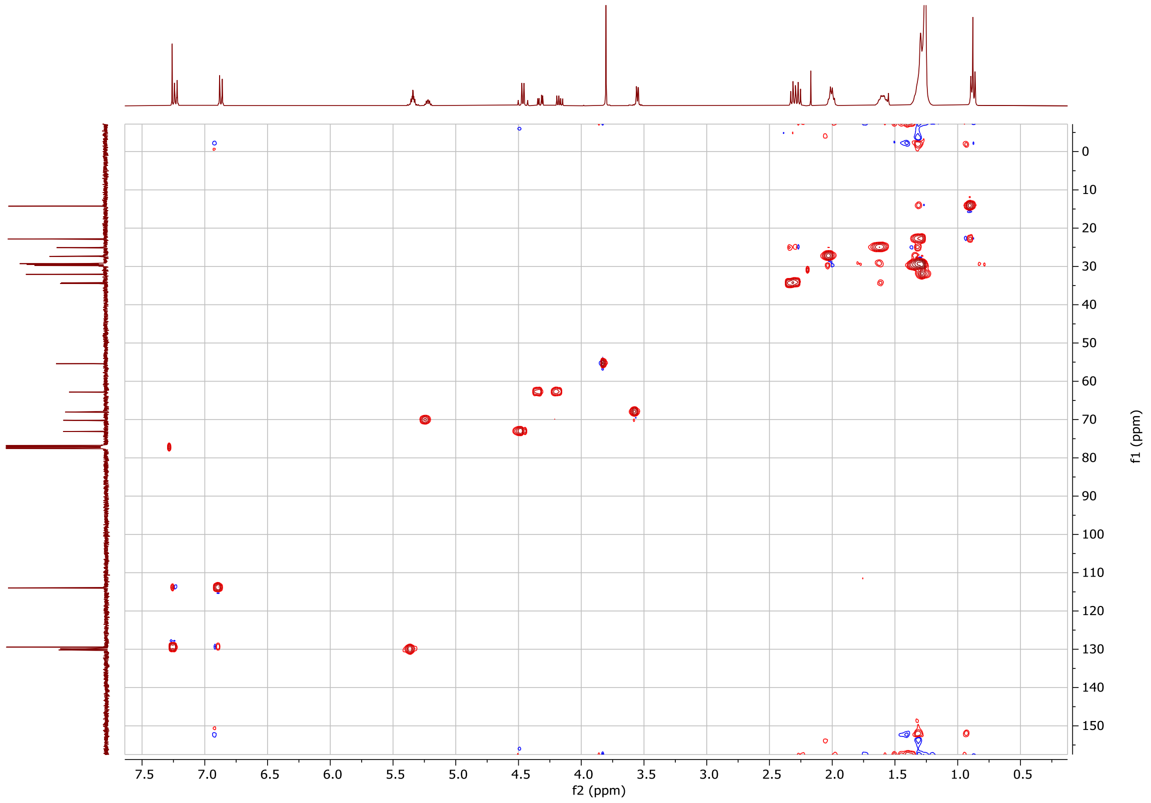

^{1}H NMR (400 MHz, $CDCl_3$) of compound (*R*)-**10b**

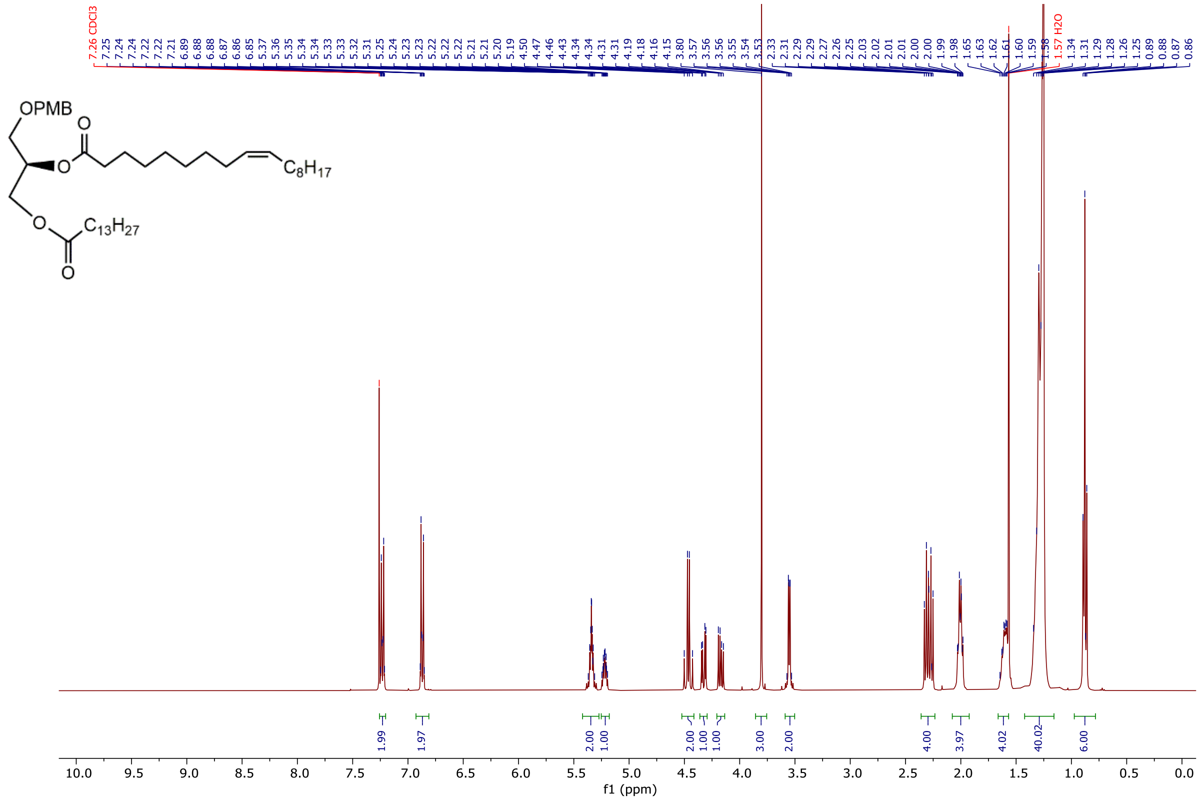

^{13}C{H} NMR (101 MHz, $CDCl_3$) of compound (*R*)-**10b**

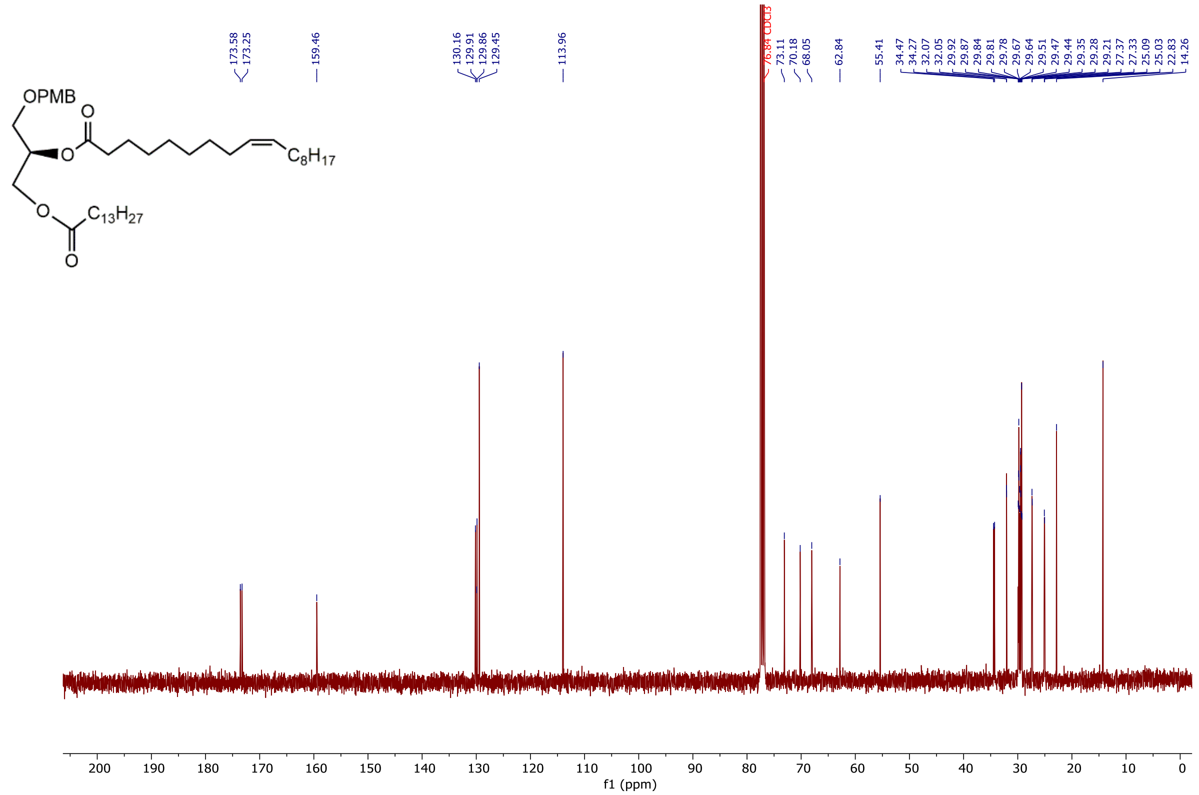

^{1}H-^{1}H COSY spectrum of compound (*R*)-**10b**

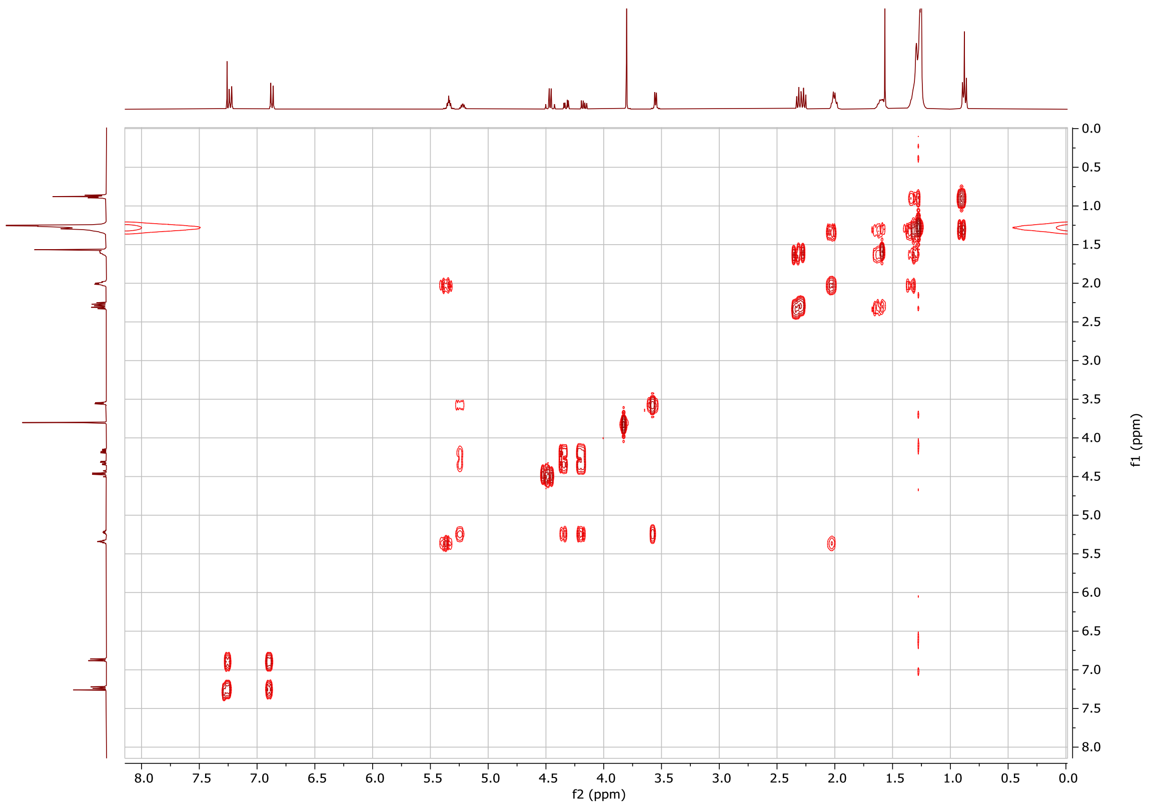

^{13}C-^{1}H HSQC spectrum of compound (*R*)-**10b**

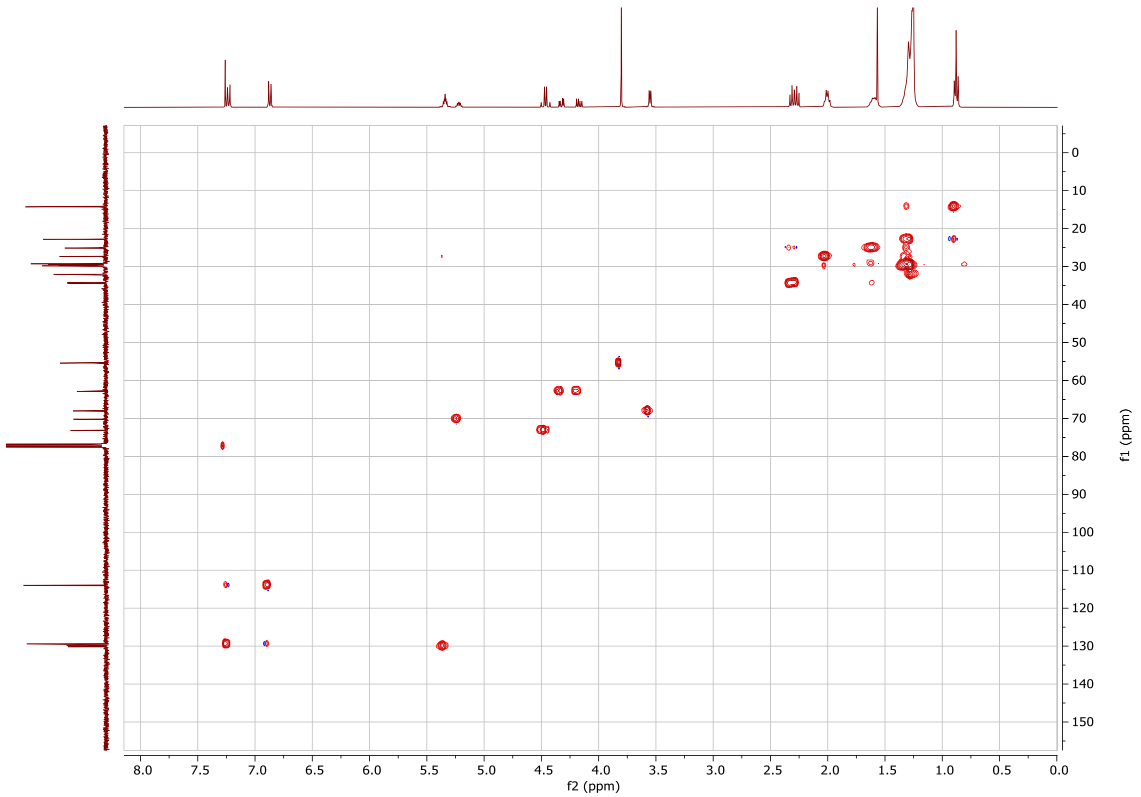

^{1}H NMR (400 MHz, $CDCl_3$) of compound (*R*)-**10c**

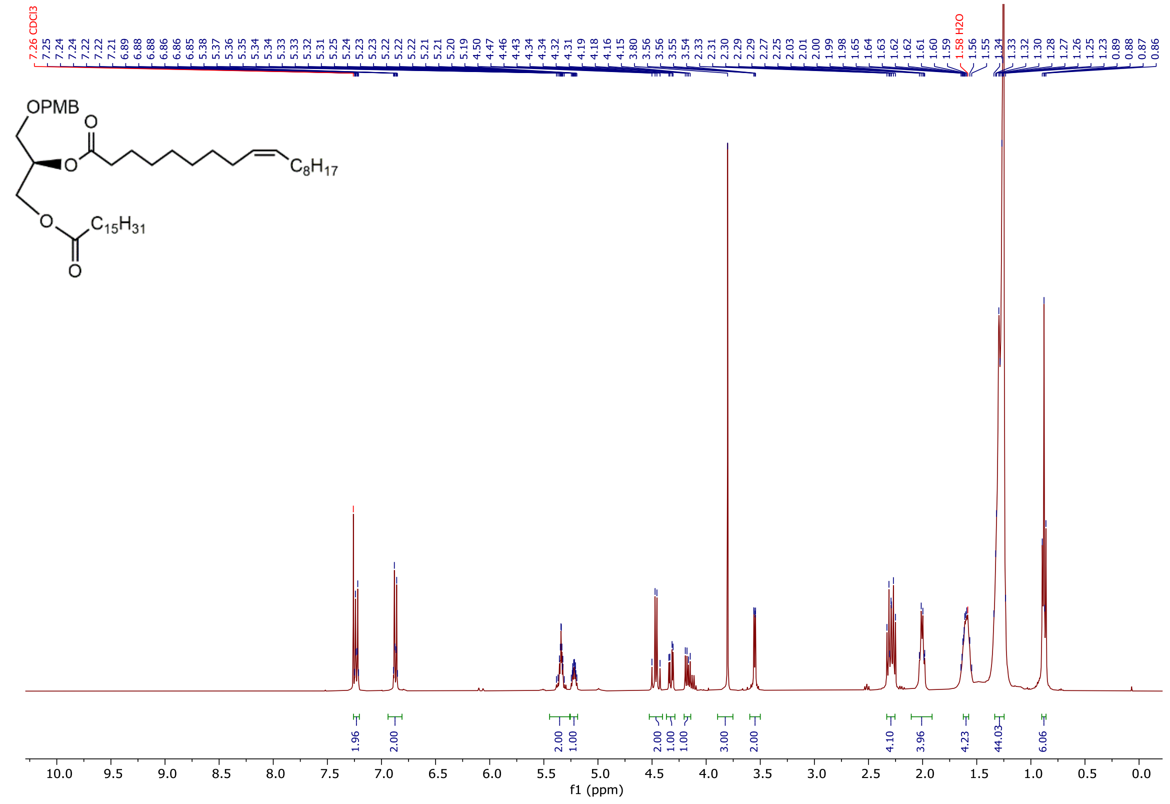

$^{13}C\{H\}$ NMR (101 MHz, $CDCl_3$) of compound (*R*)-**10c**

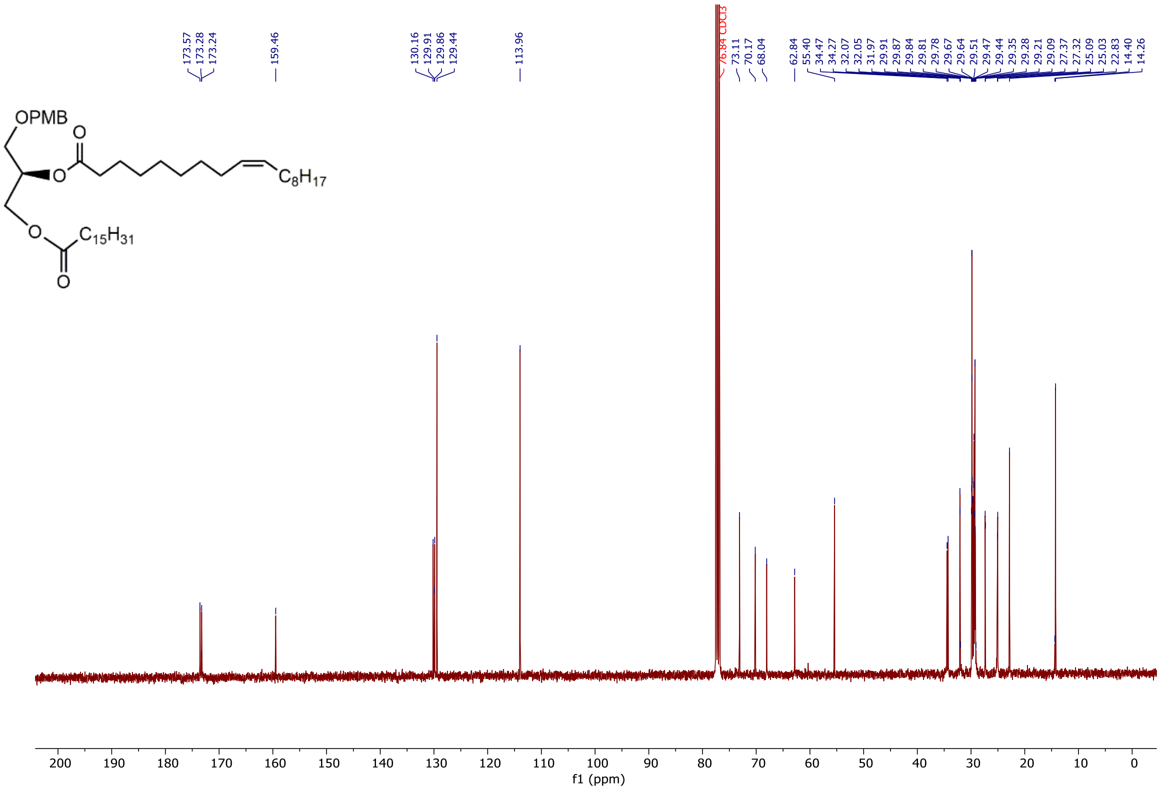

^{1}H-^{1}H COSY spectrum of compound (*R*)-**10c**

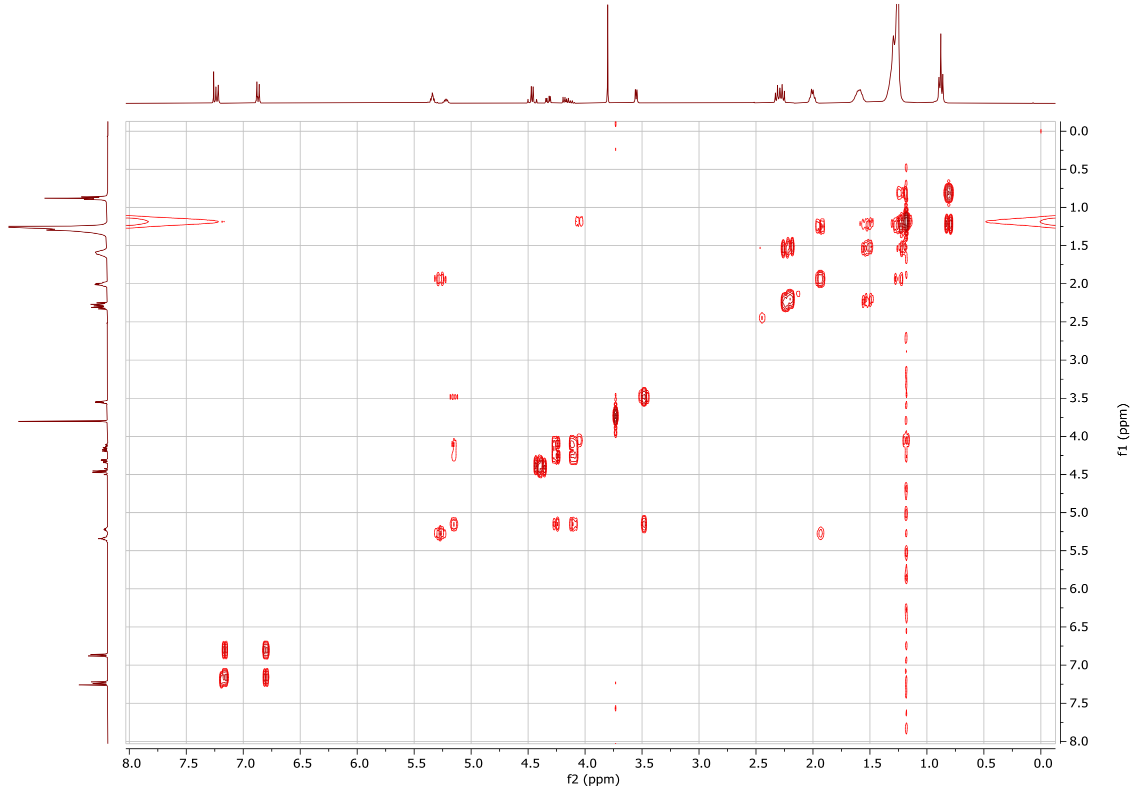

^{13}C-^{1}H HSQC spectrum of compound (*R*)-**10c**

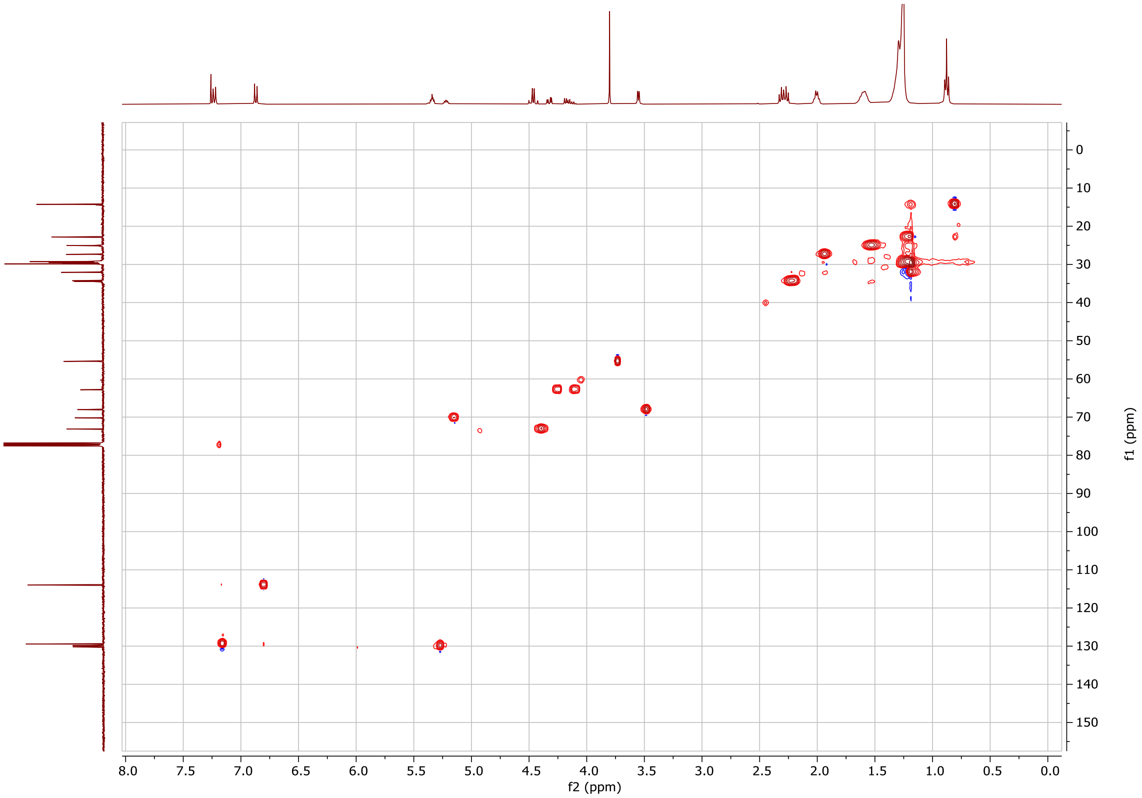

^{1}H NMR (400 MHz, $CDCl_3$) of compound (*R*)-**10d**

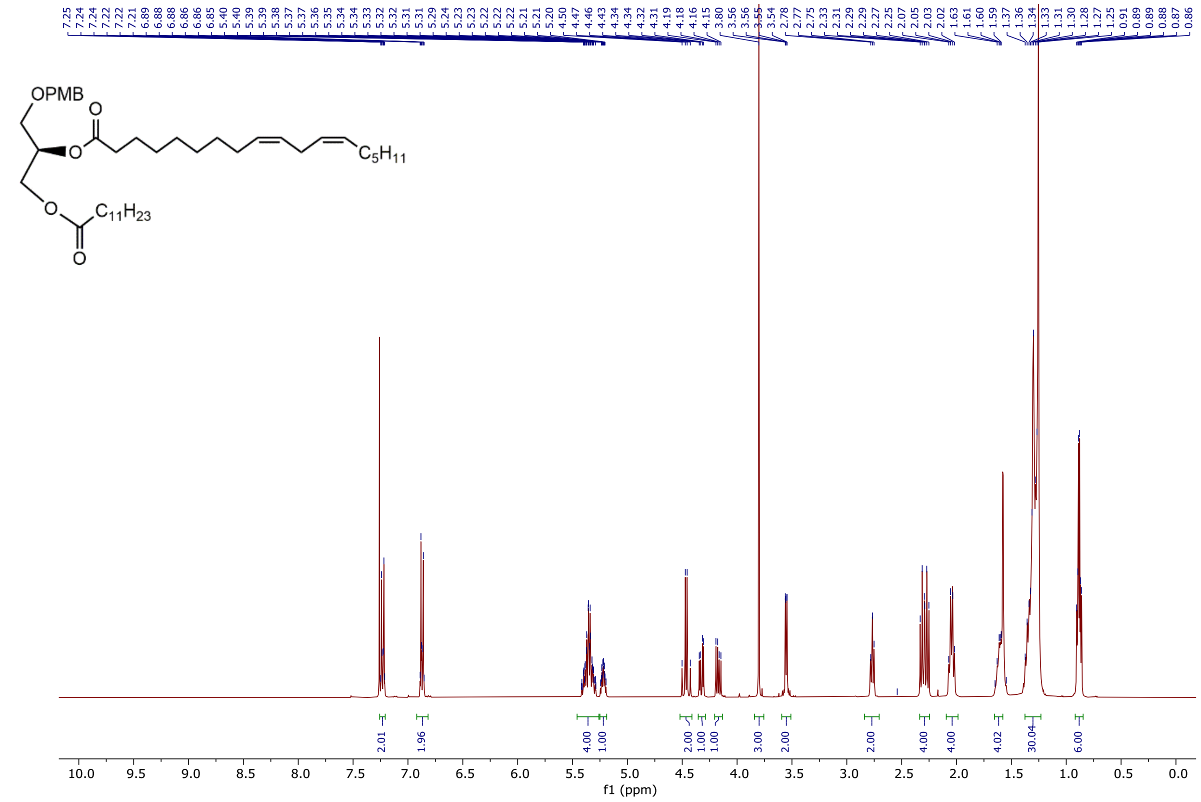

^{13}C{H} NMR (101 MHz, $CDCl_3$) of compound (*R*)-**10d**

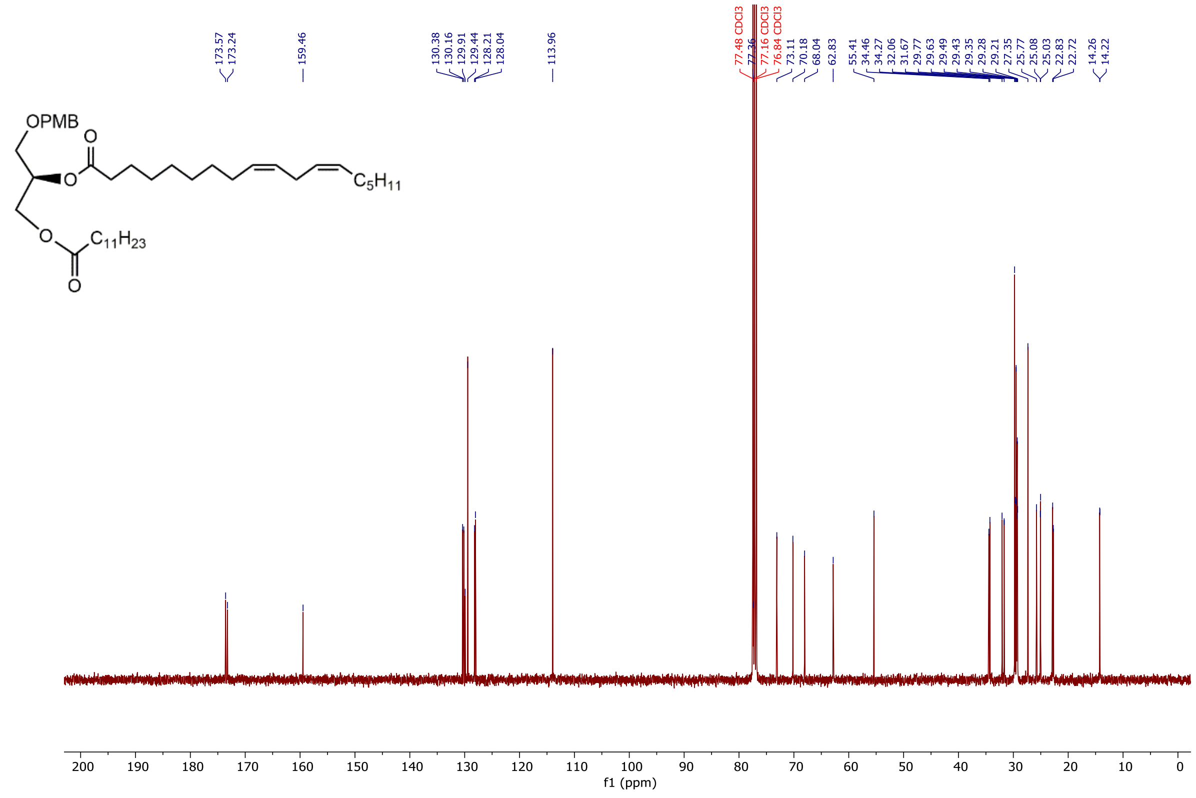

^{1}H-^{1}H COSY spectrum of compound (*R*)-**10d**

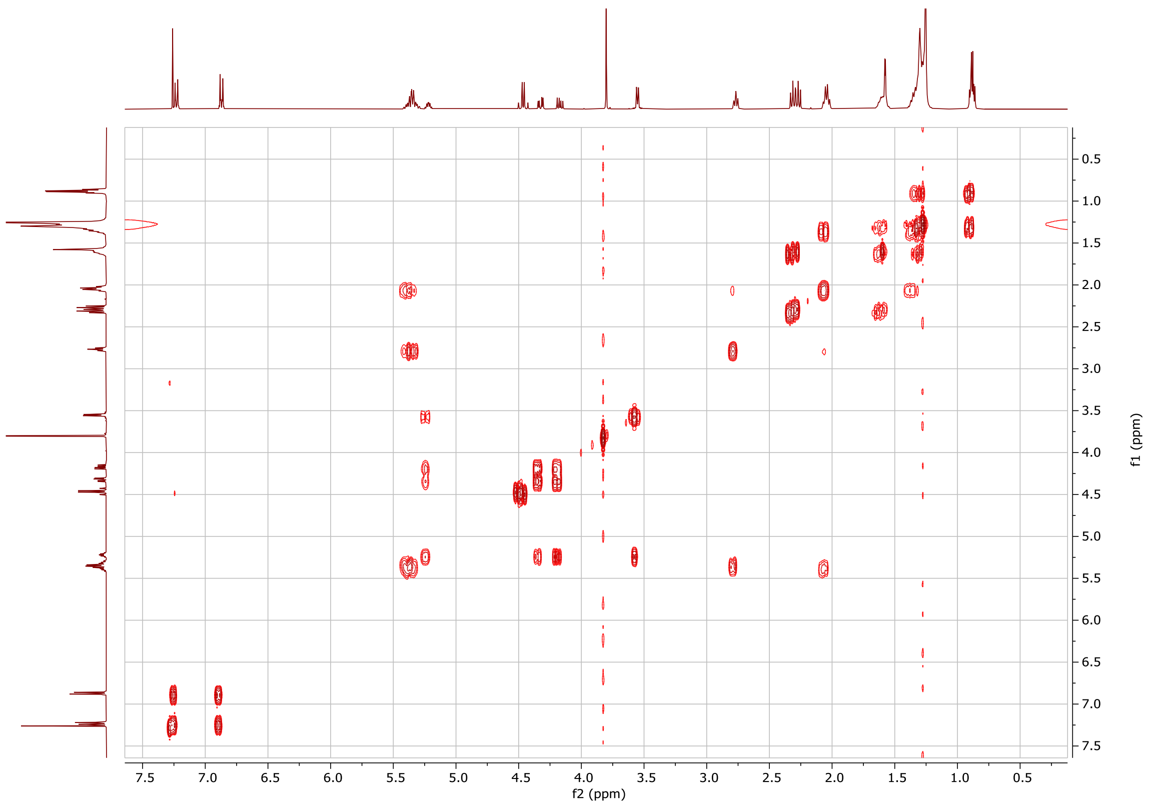

^{13}C-^{1}H HSQC spectrum of compound (*R*)-**10d**

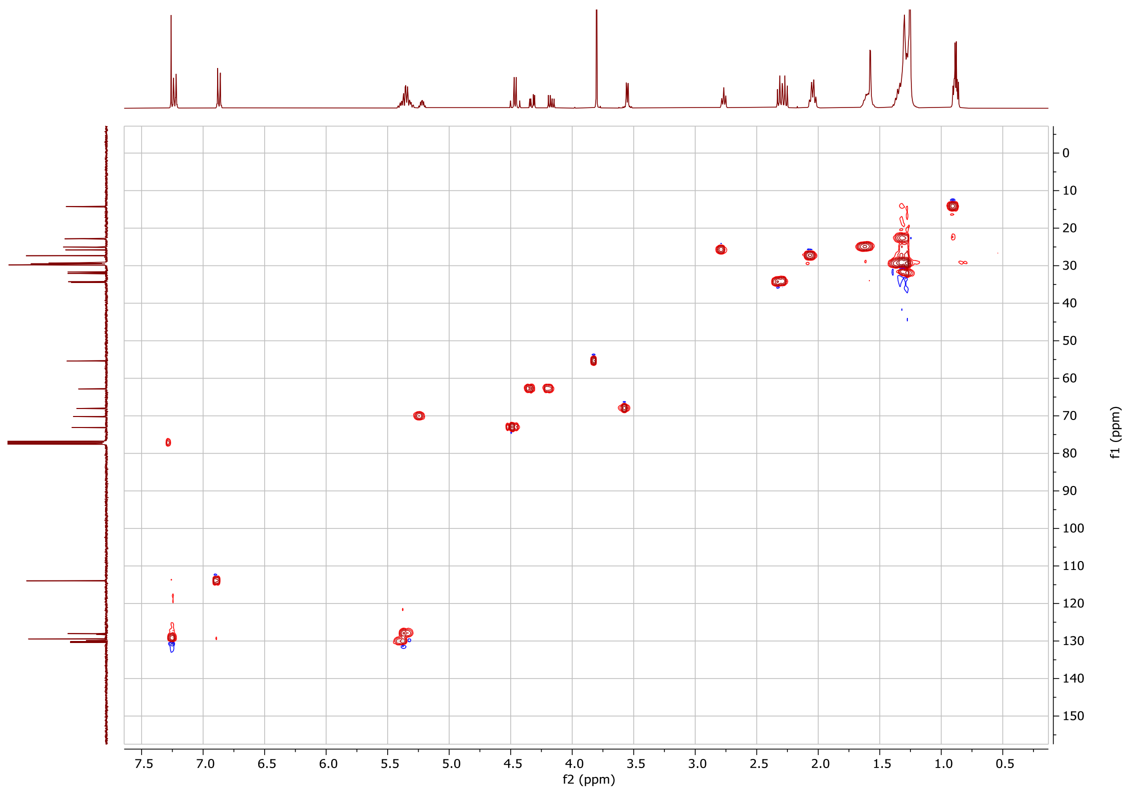

^{1}H NMR (400 MHz, $CDCl_3$) of compound (*R*)-**10e**

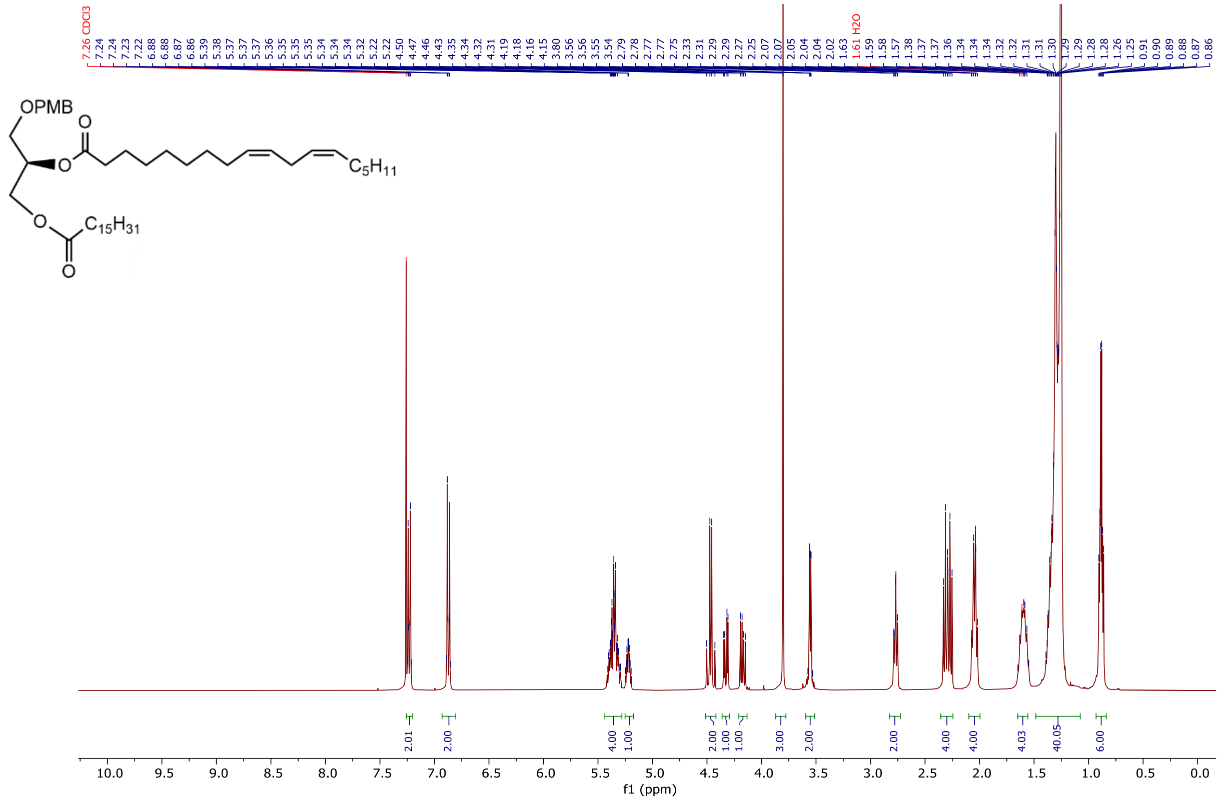

^{13}C{H} NMR (101 MHz, $CDCl_3$) of compound (*R*)-**10e**

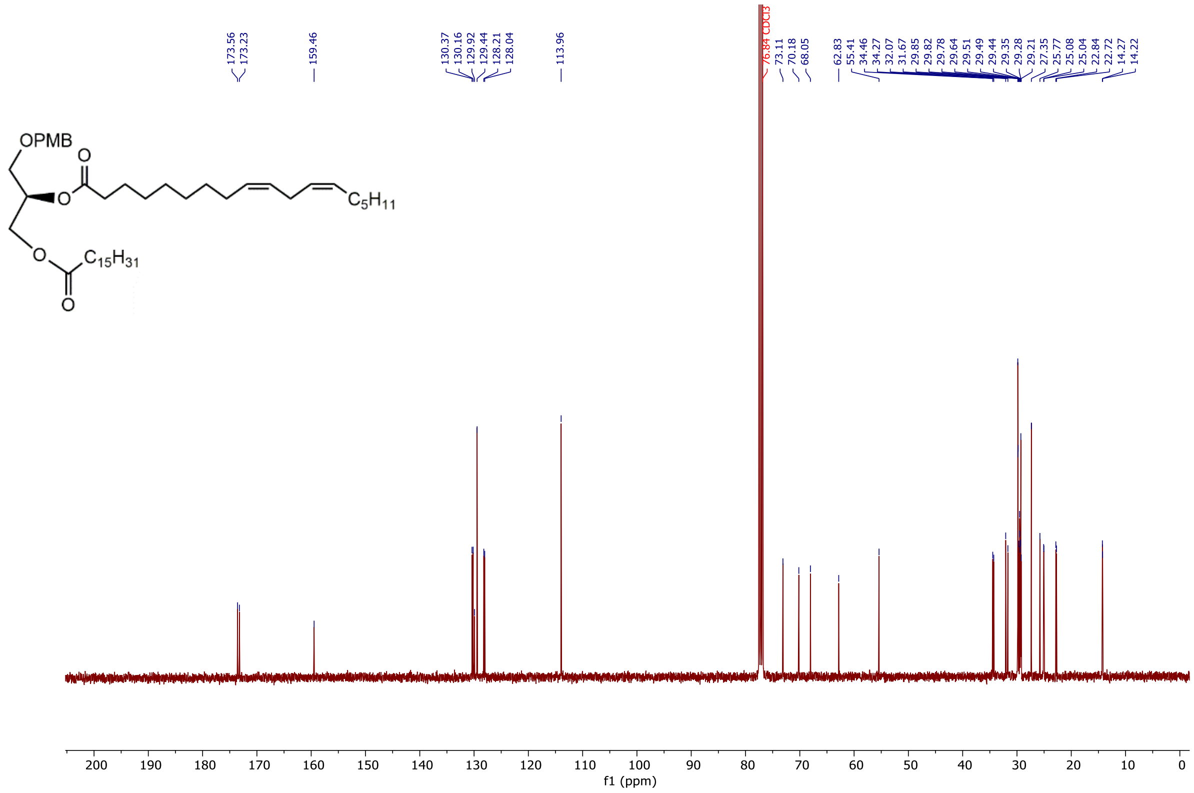

^{1}H-^{1}H COSY spectrum of compound (*R*)-**10e**

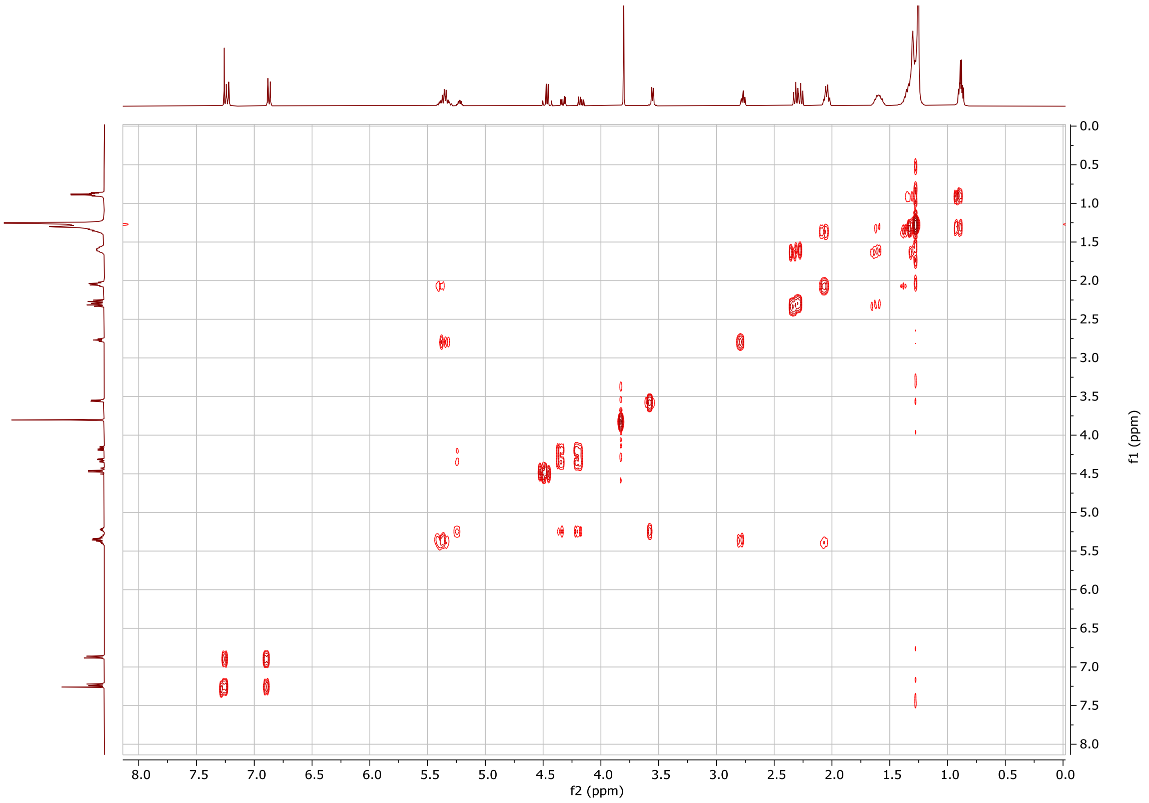

^{13}C-^{1}H HSQC spectrum of compound (*R*)-**10e**

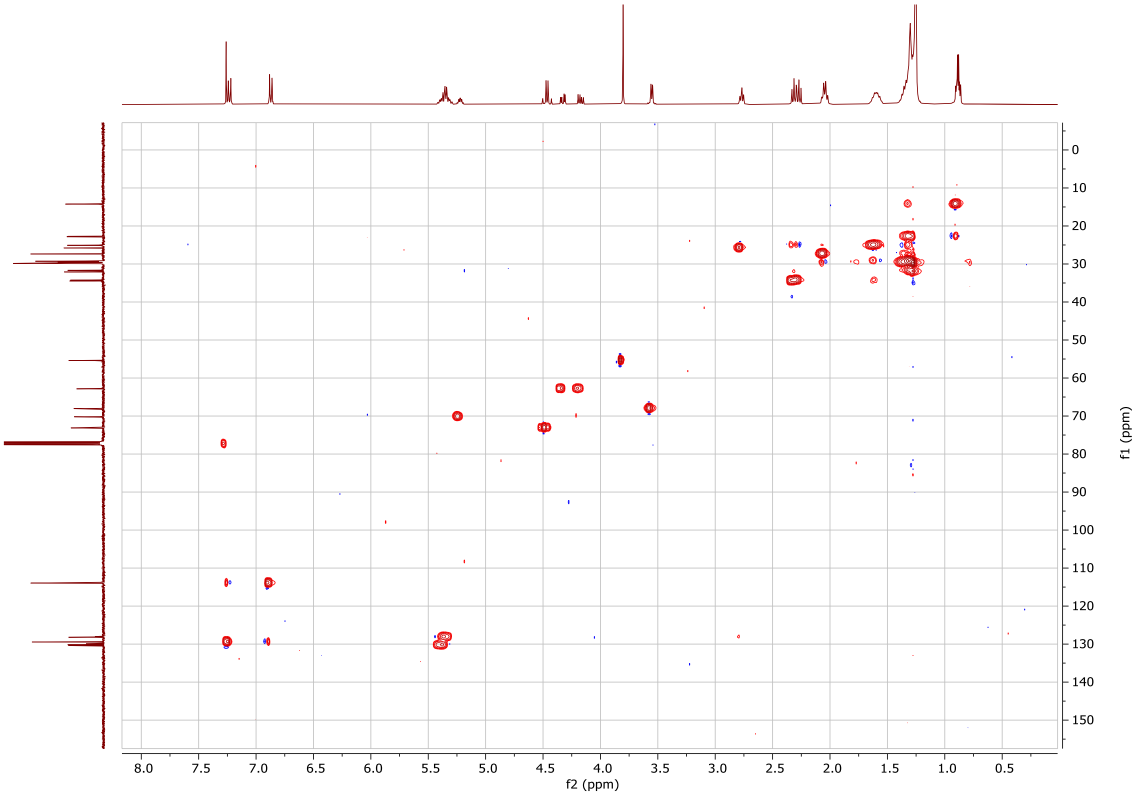

^{1}H NMR (400 MHz, $CDCl_3$) of compound (*R*)-**11a**

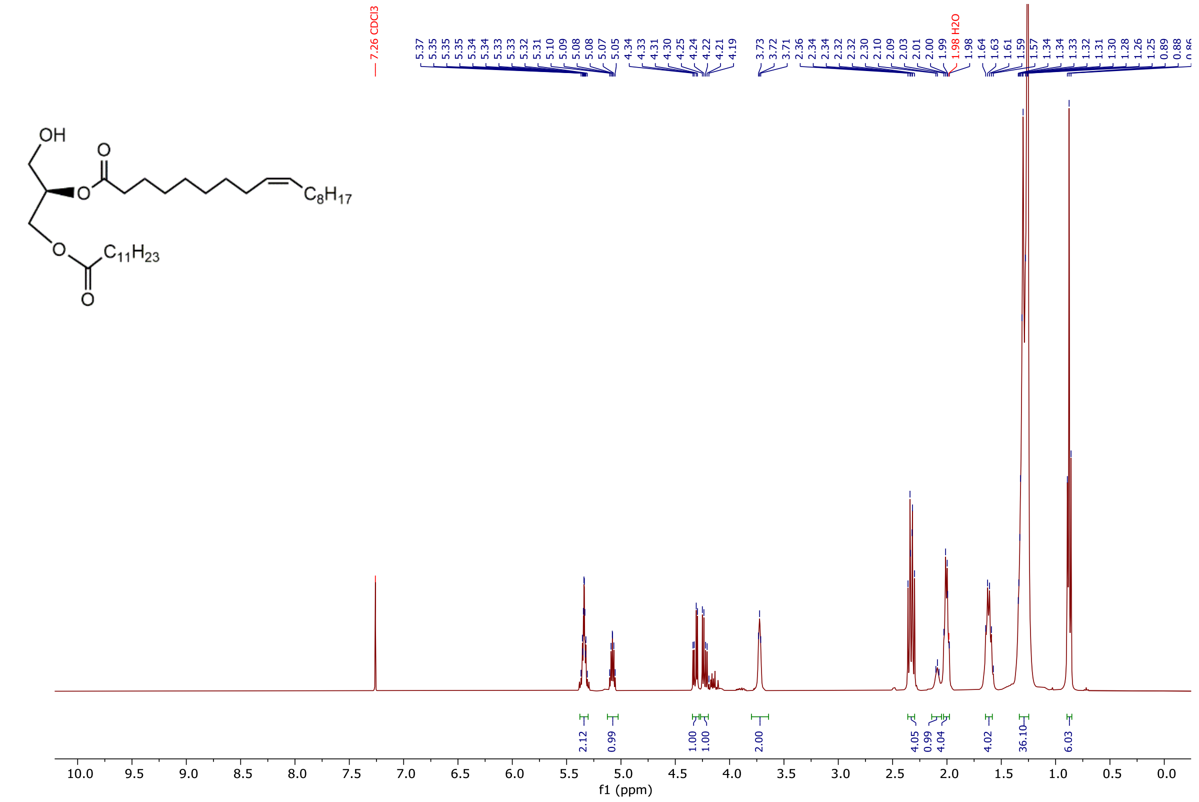

^{13}C{H} NMR (101 MHz, $CDCl_3$) of compound (*R*)-**11a**

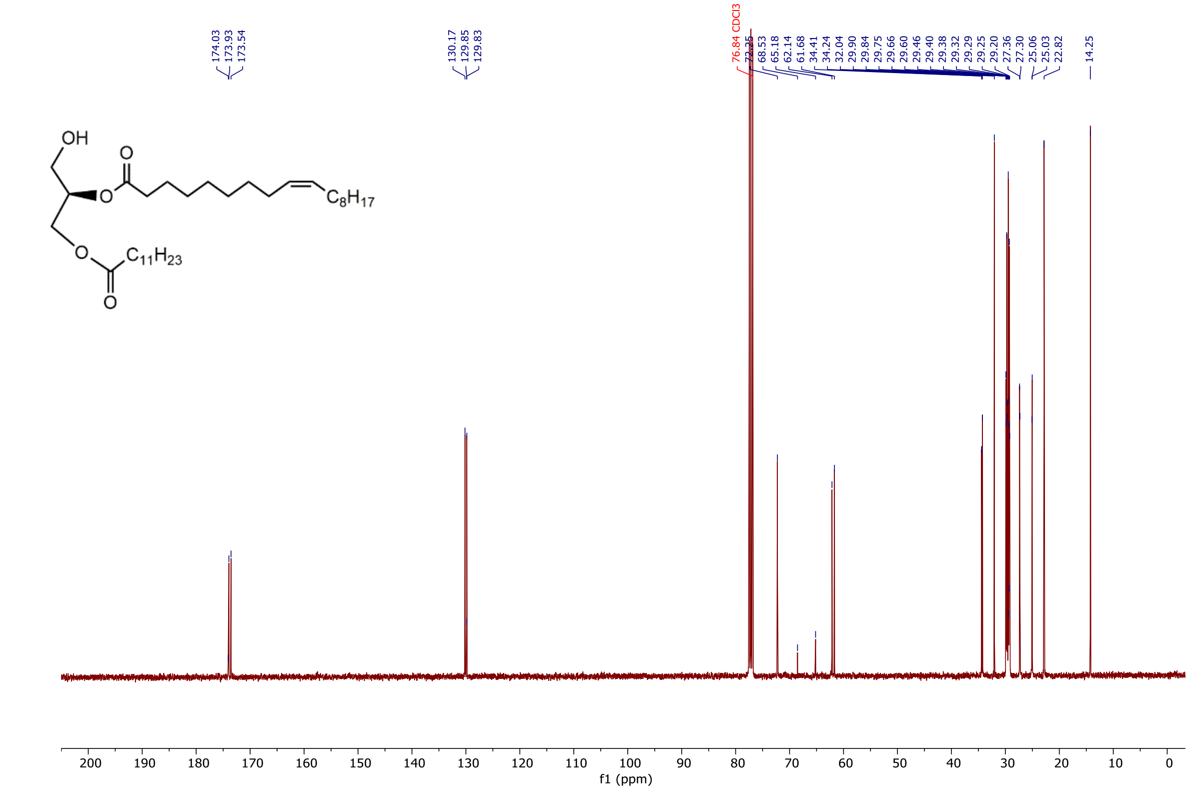

^{1}H-^{1}H COSY spectrum of compound (*R*)-**11a**

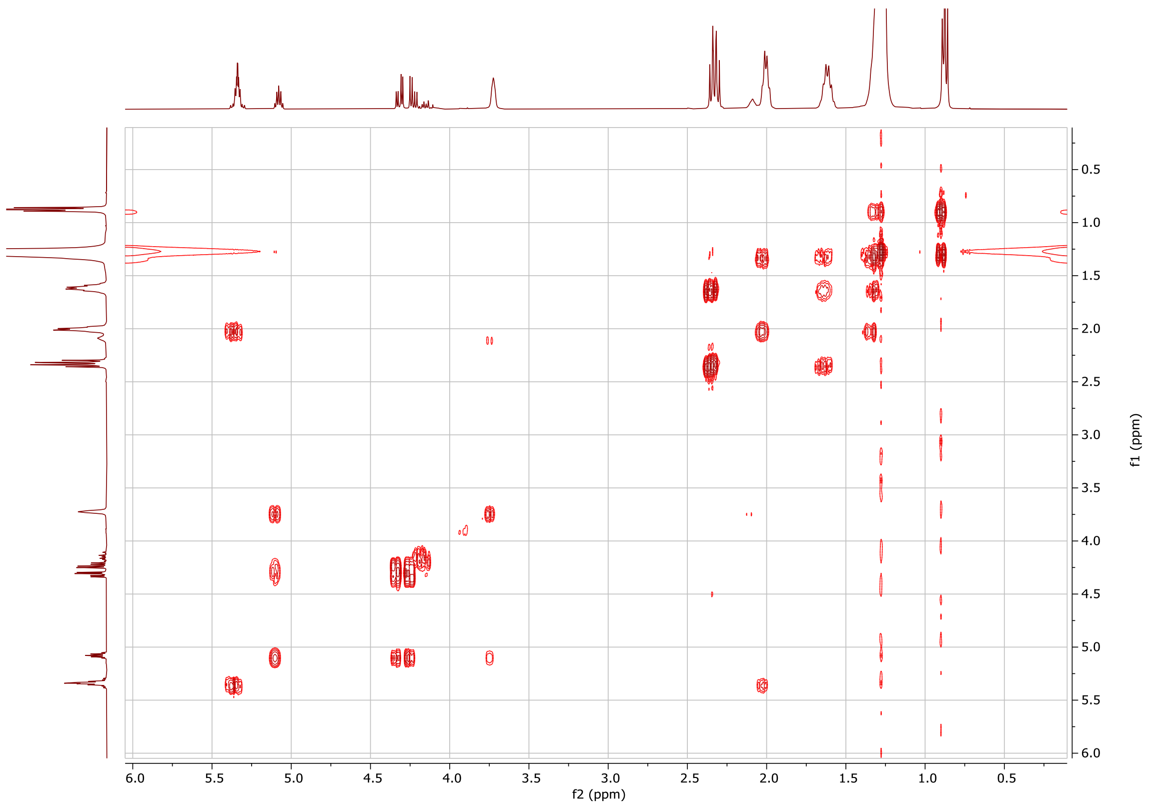

^{13}C-^{1}H HSQC spectrum of compound (*R*)-**11a**

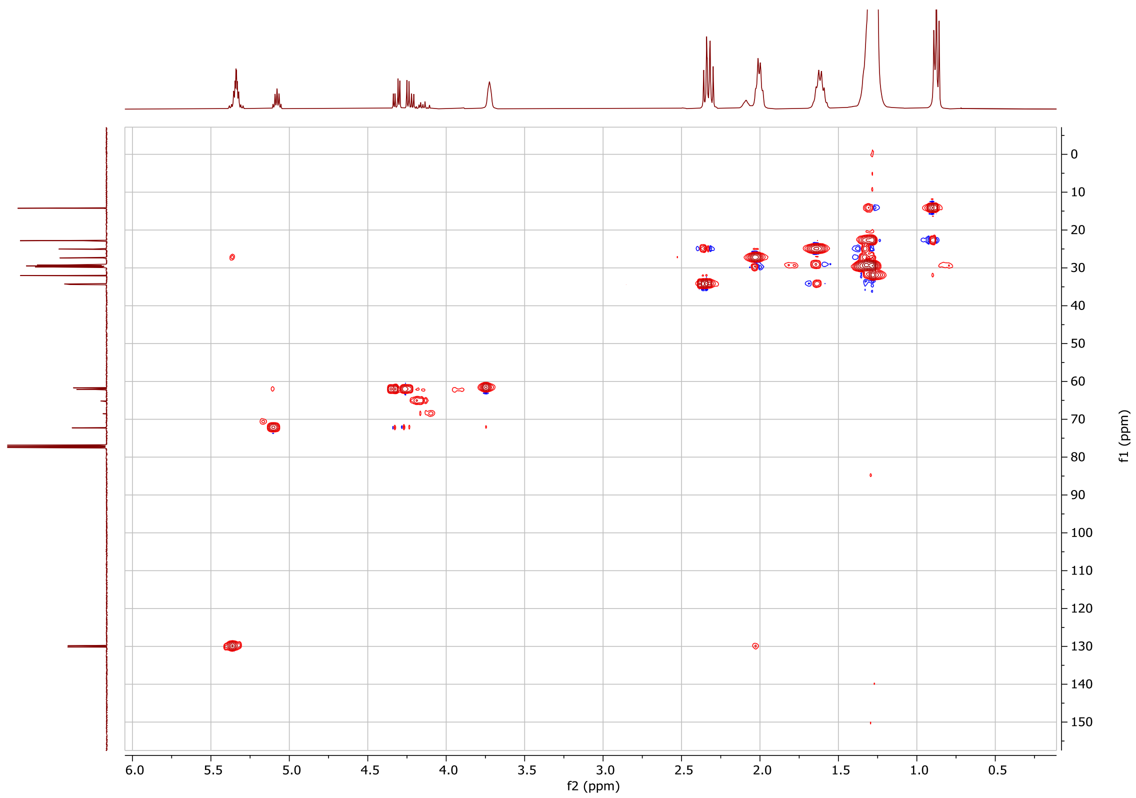

^{1}H NMR (400 MHz, $CDCl_3$) of compound (*R*)-**11b**

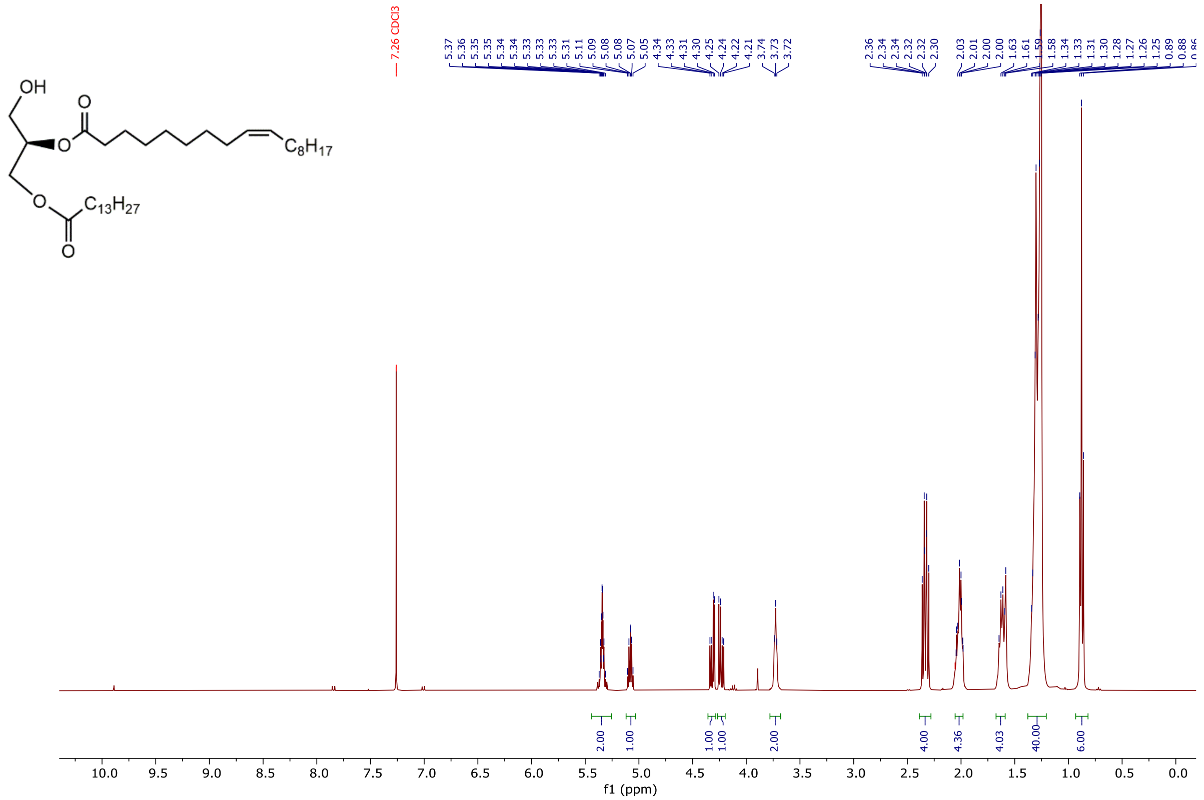

^{13}C{H} NMR (101 MHz, $CDCl_3$) of compound (*R*)-**11b**

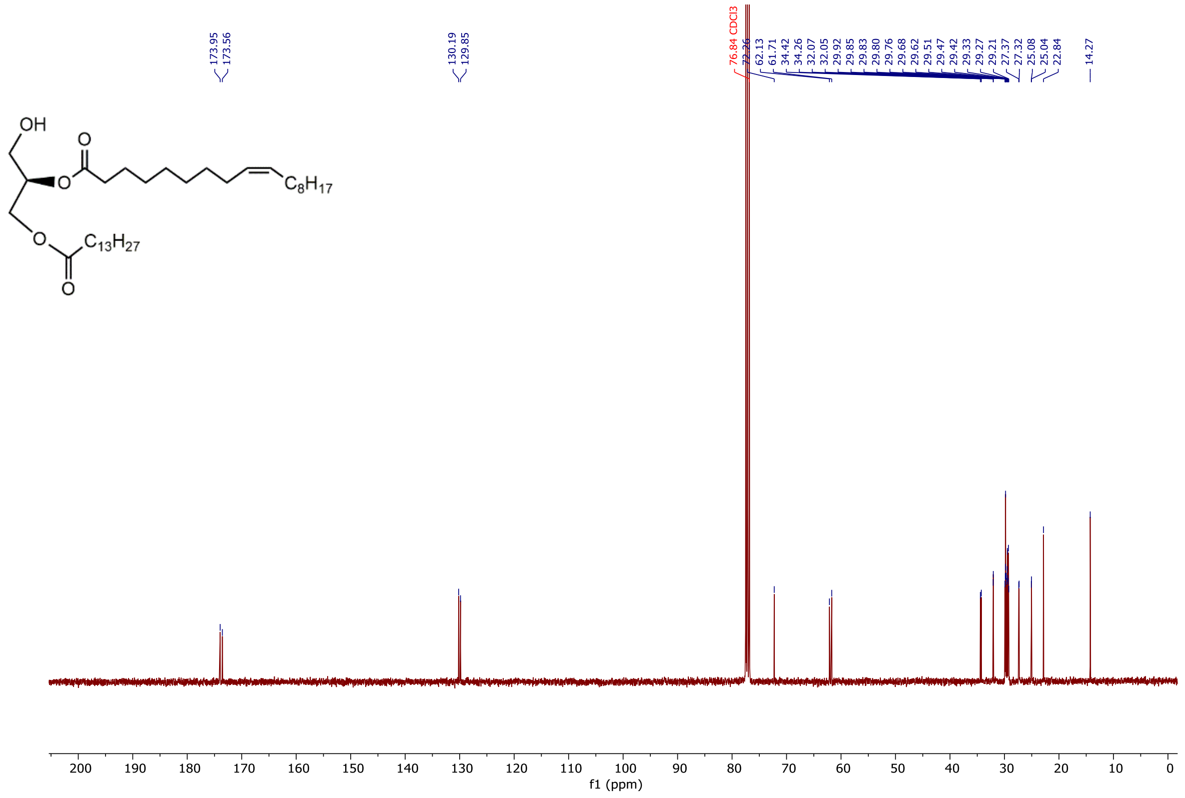

^{1}H-^{1}H COSY spectrum of compound (*R*)-**11b**

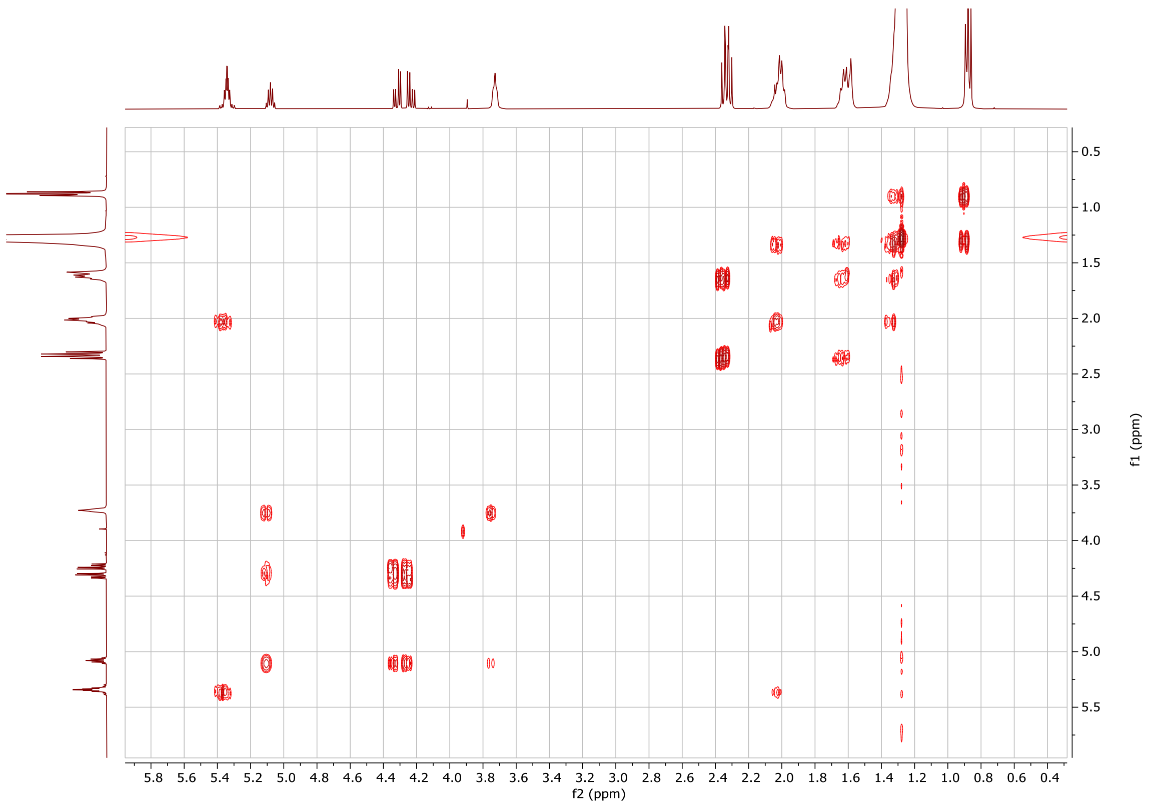

^{13}C-^{1}H HSQC spectrum of compound (*R*)-**11b**

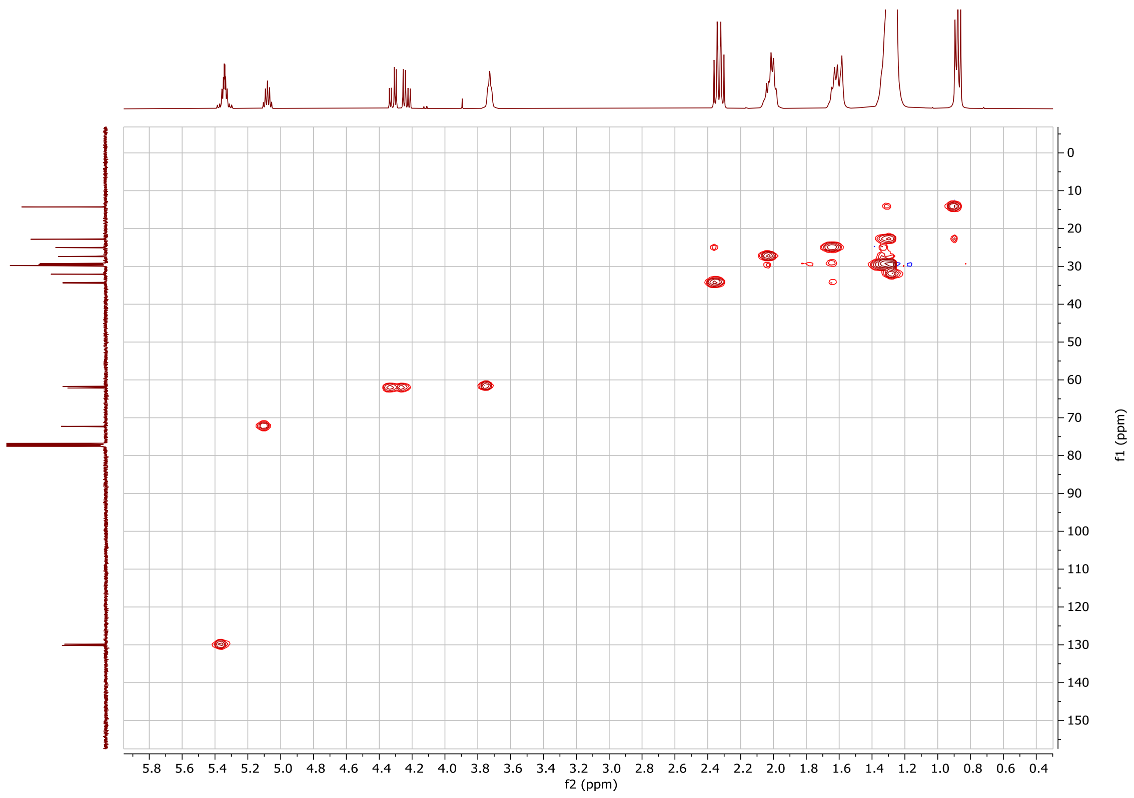

^{1}H NMR (400 MHz, $CDCl_3$) of compound (*R*)-**11c**

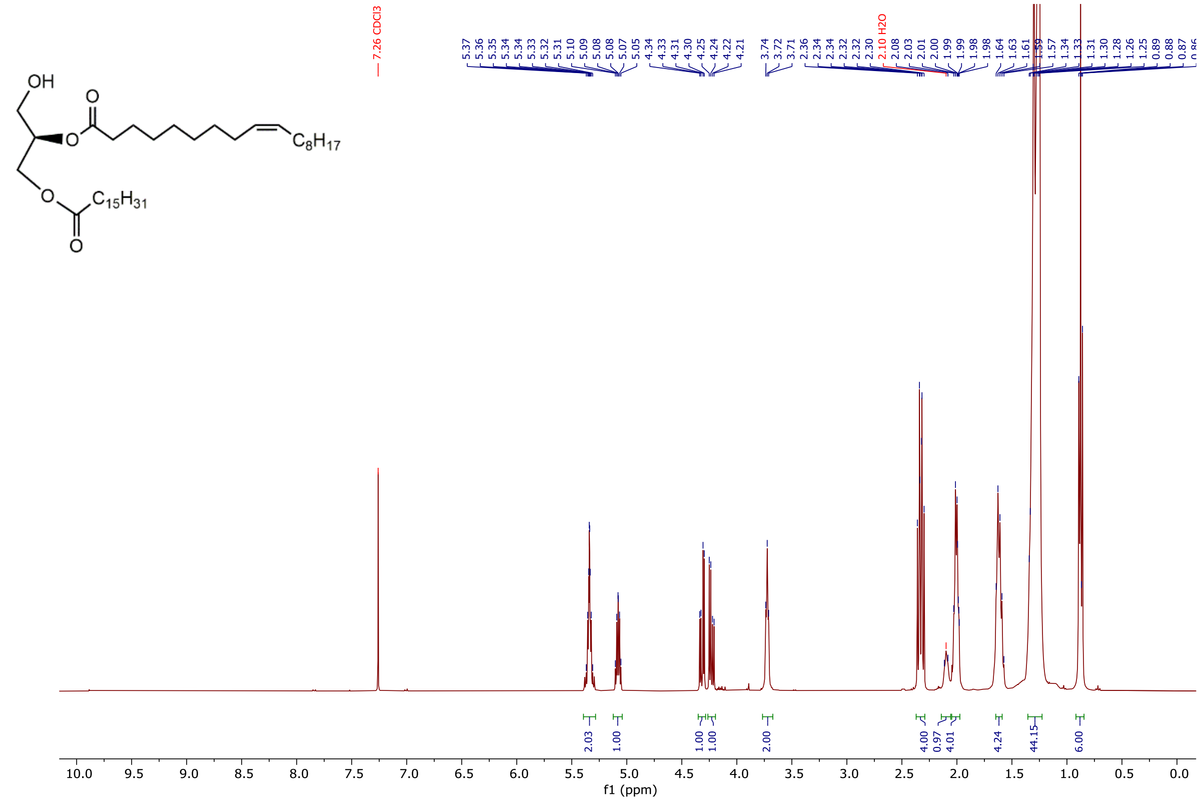

^{13}C{H} NMR (101 MHz, $CDCl_3$) of compound (*R*)-**11c**

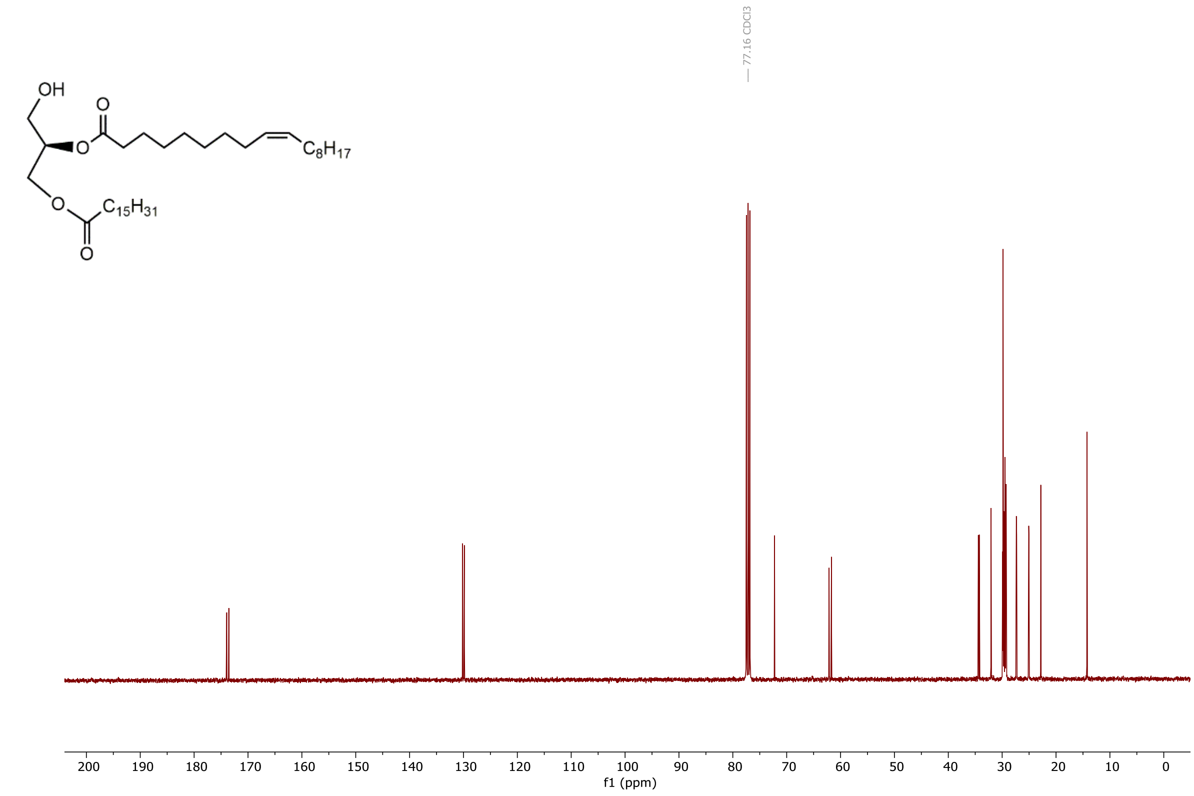

^{1}H-^{1}H COSY spectrum of compound (*R*)-**11c**

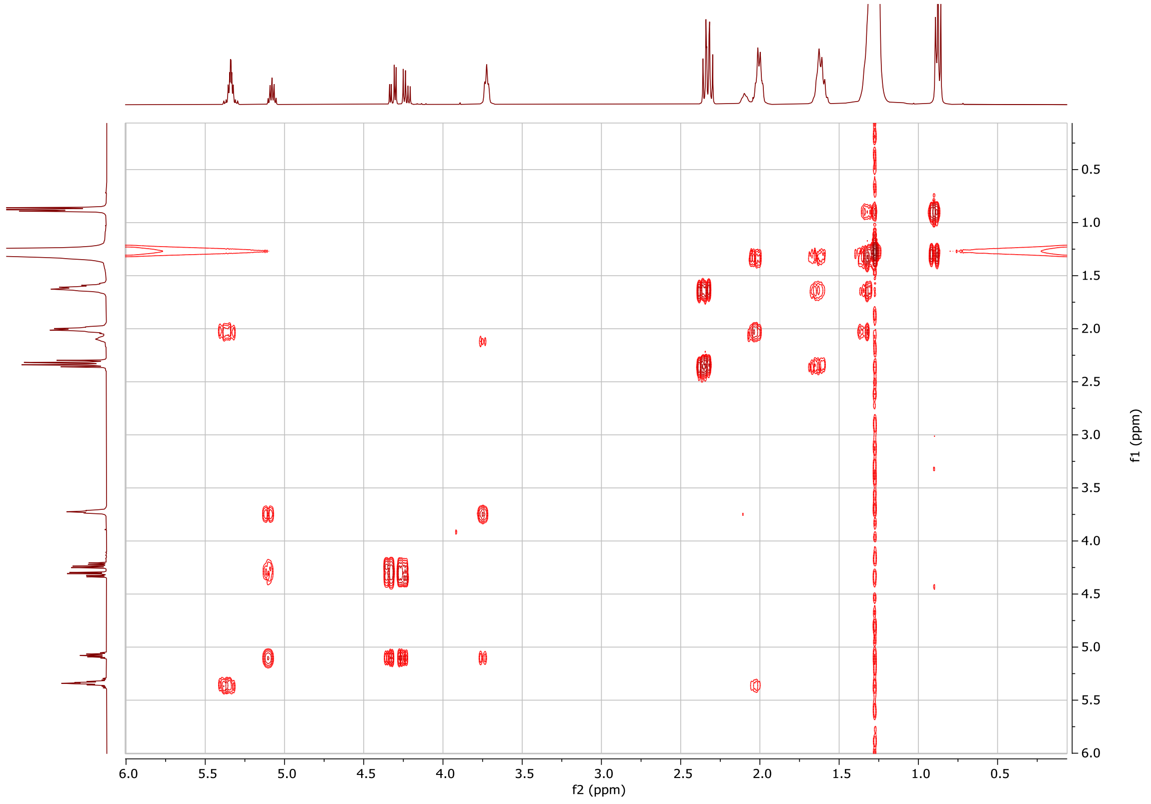

^{13}C-^{1}H HSQC spectrum of compound (*R*)-**11c**

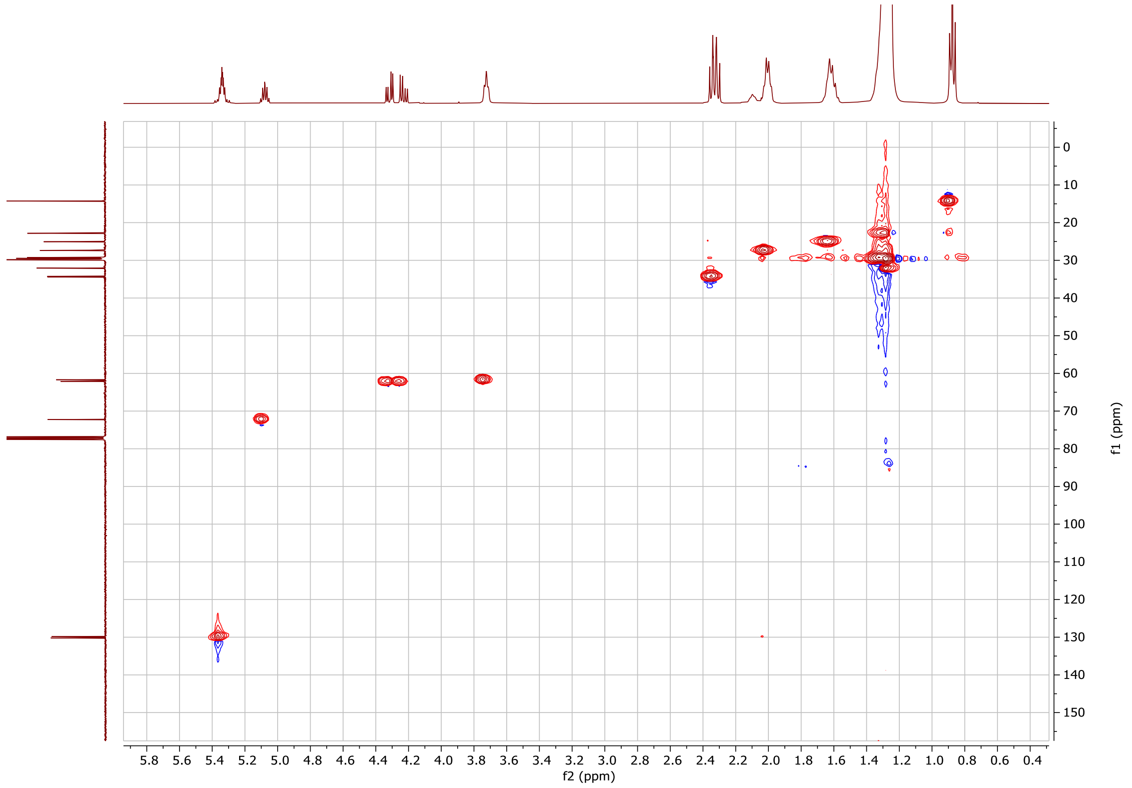

^{1}H NMR (400 MHz, $CDCl_3$) of compound *(R)*-**11e**

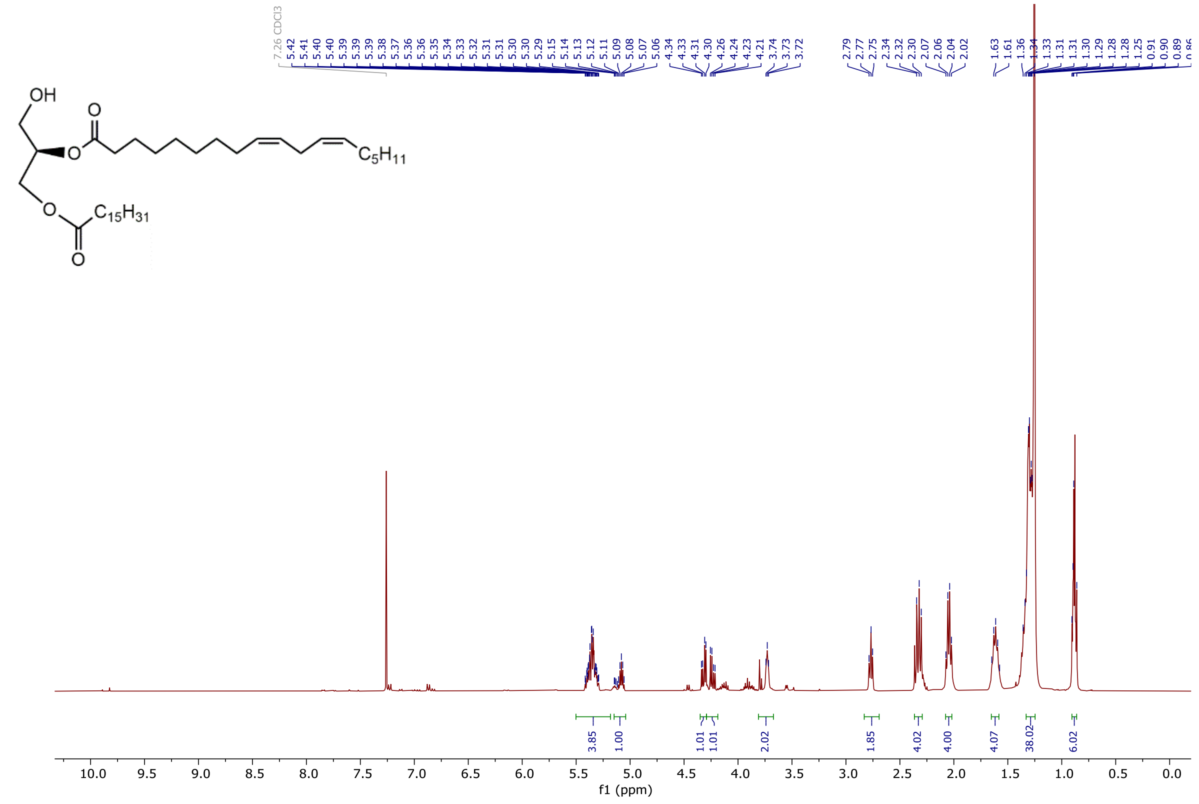

$^{13}C\{H\}$ NMR (101 MHz, $CDCl_3$) of compound *(R)*-**11e**

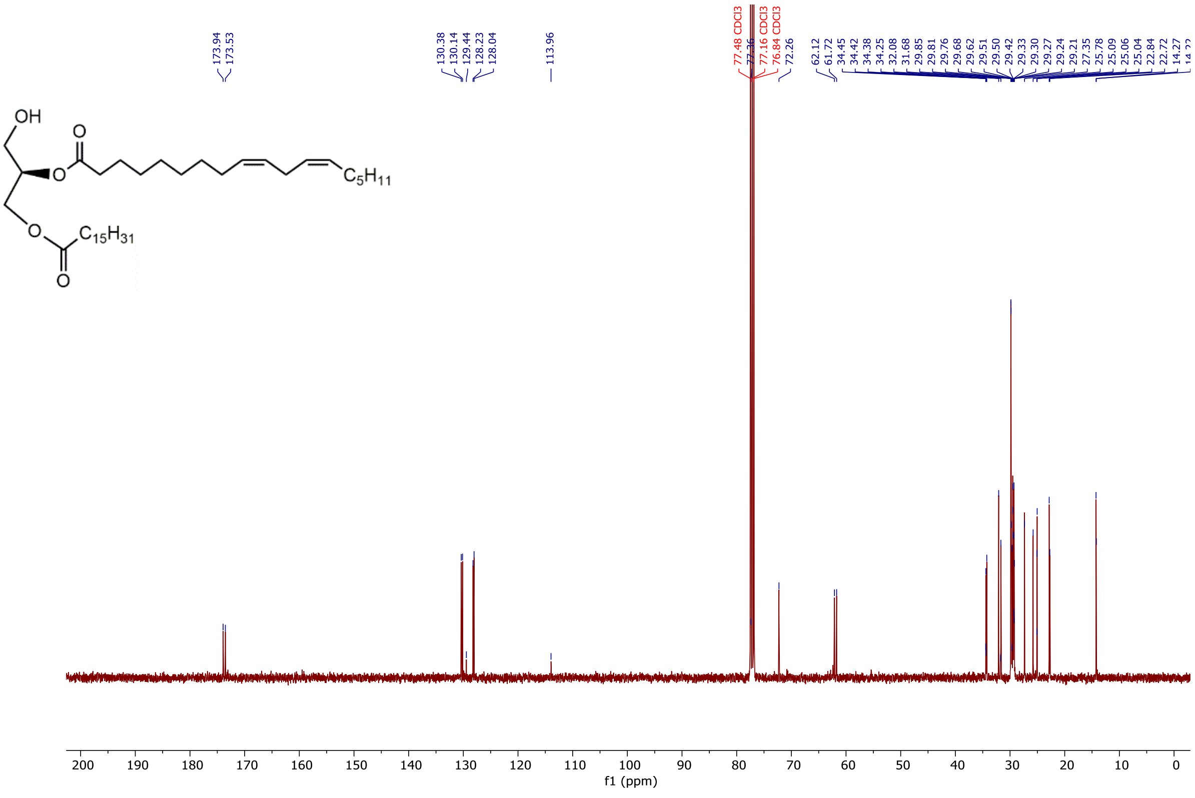

^{1}H-^{1}H COSY spectrum of compound (*R*)-**11e**

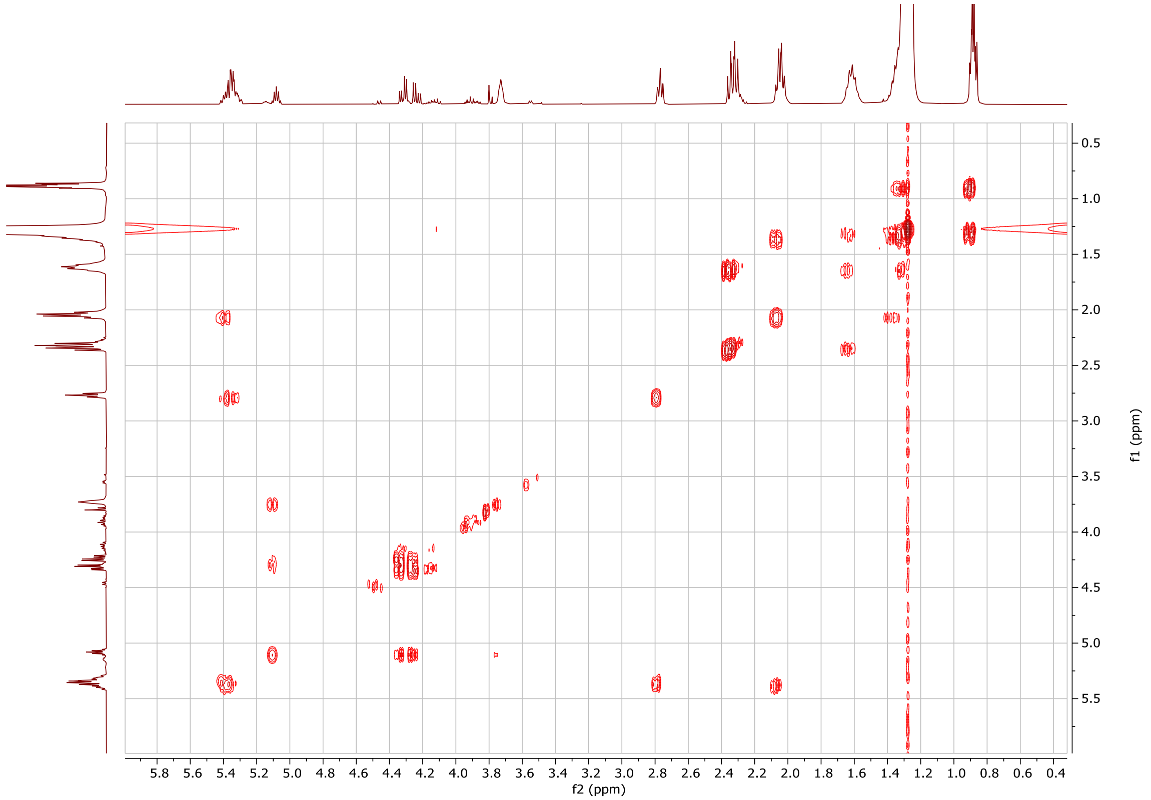

^{13}C-^{1}H HSQC spectrum of compound (*R*)-**11e**

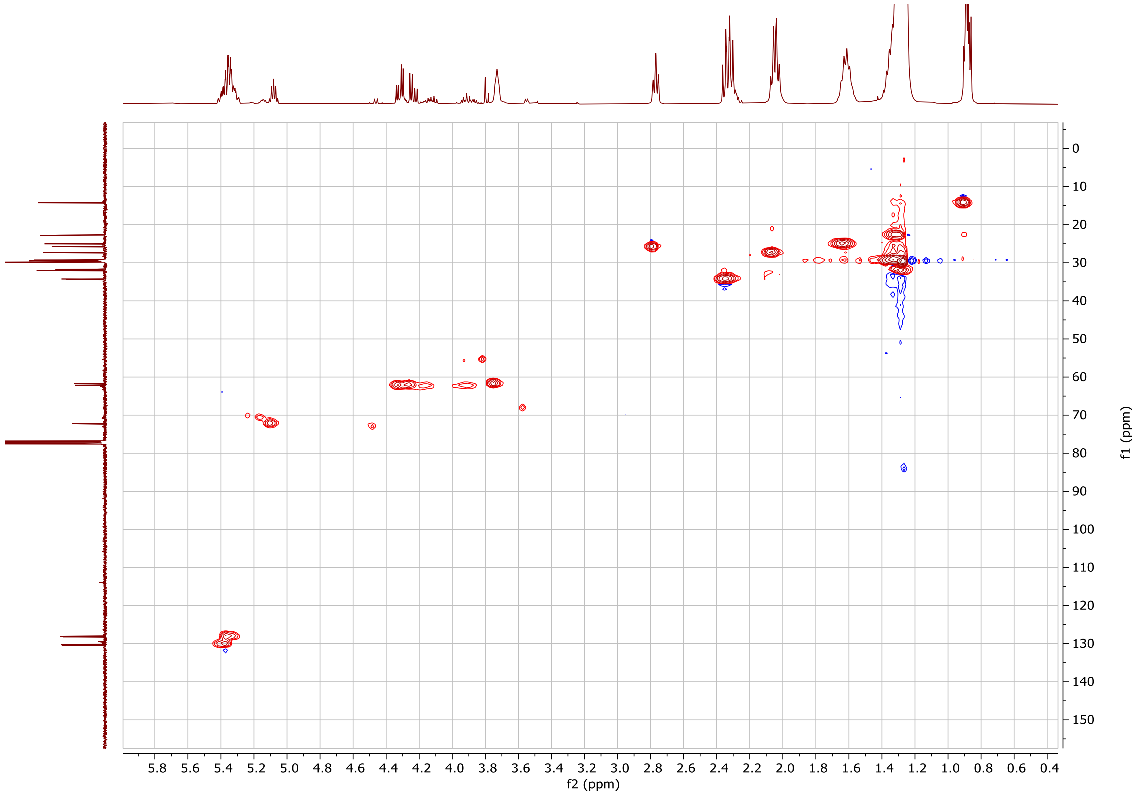

^1H NMR (400 MHz, $CDCl_3$) of compound (*S*)-**1**

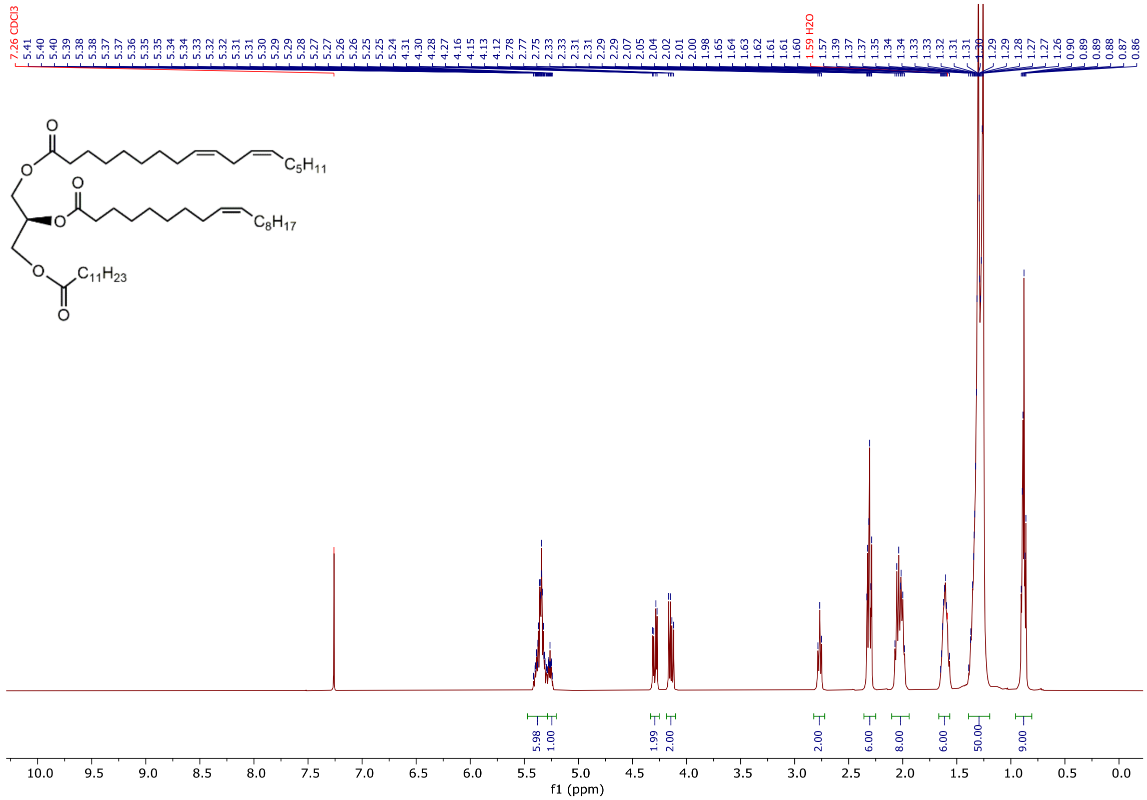

$^{13}C\{H\}$ NMR (101 MHz, $CDCl_3$) of compound (*S*)-**1**

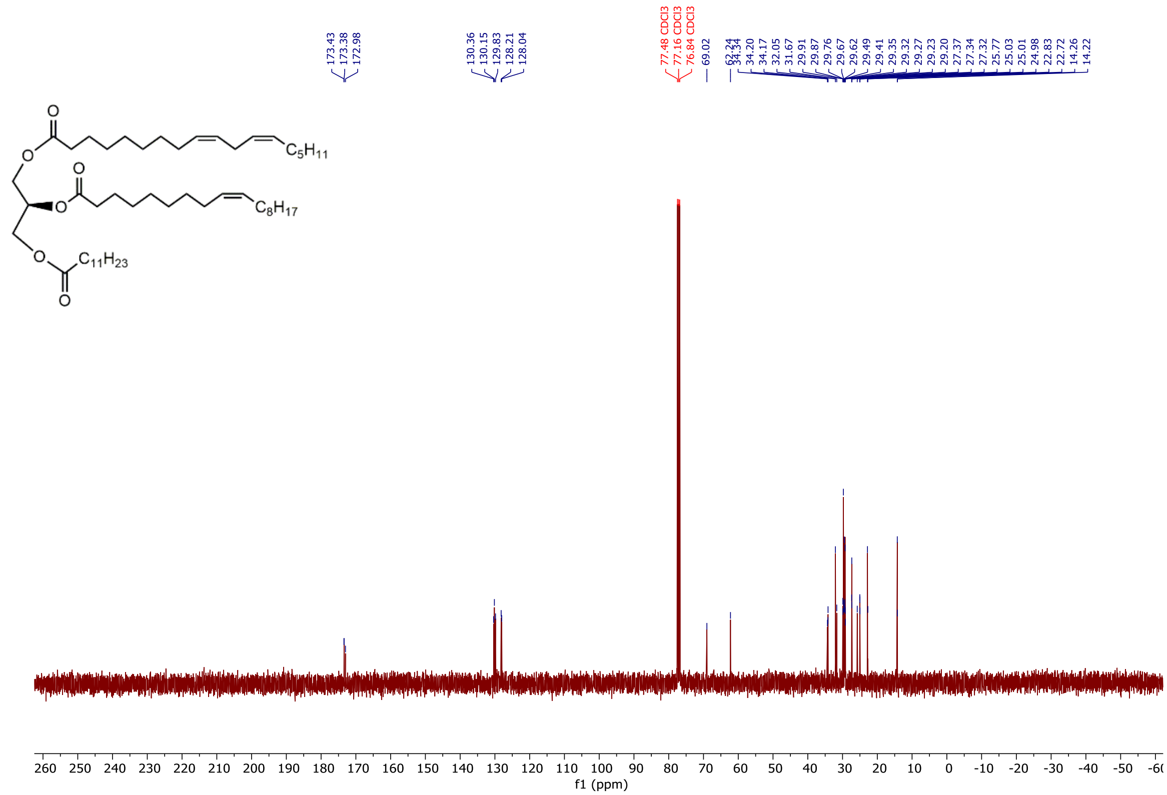

^{1}H-^{1}H COSY spectrum of compound (*S*)-**1**

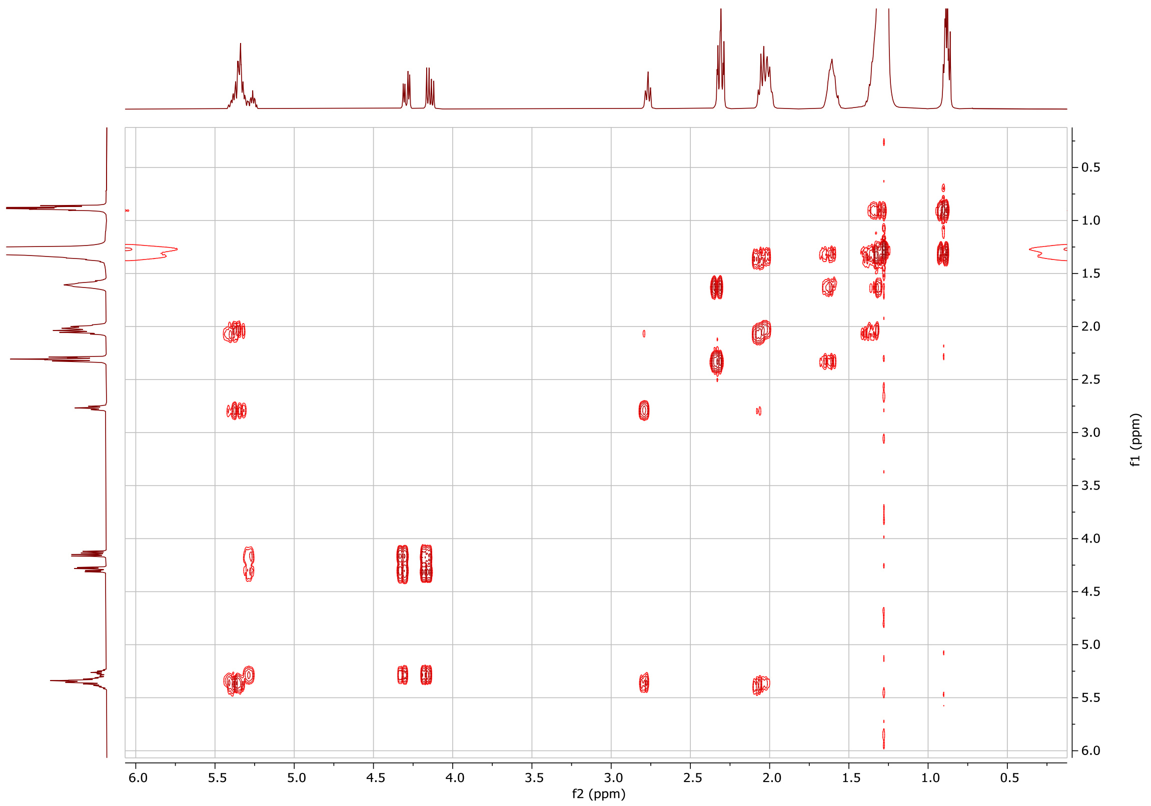

^{13}C-^{1}H HSQC spectrum of compound (*S*)-**1**

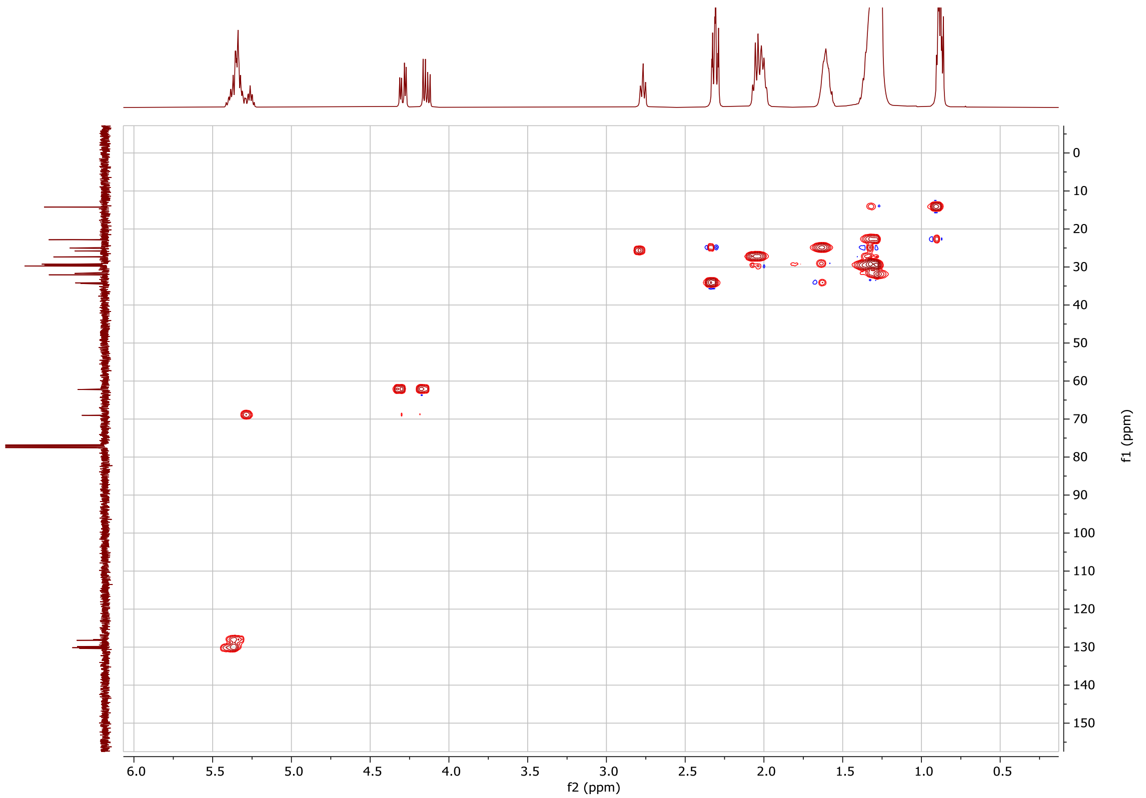

^{1}H NMR (400 MHz, $CDCl_3$) of compound (*S*)-**2**

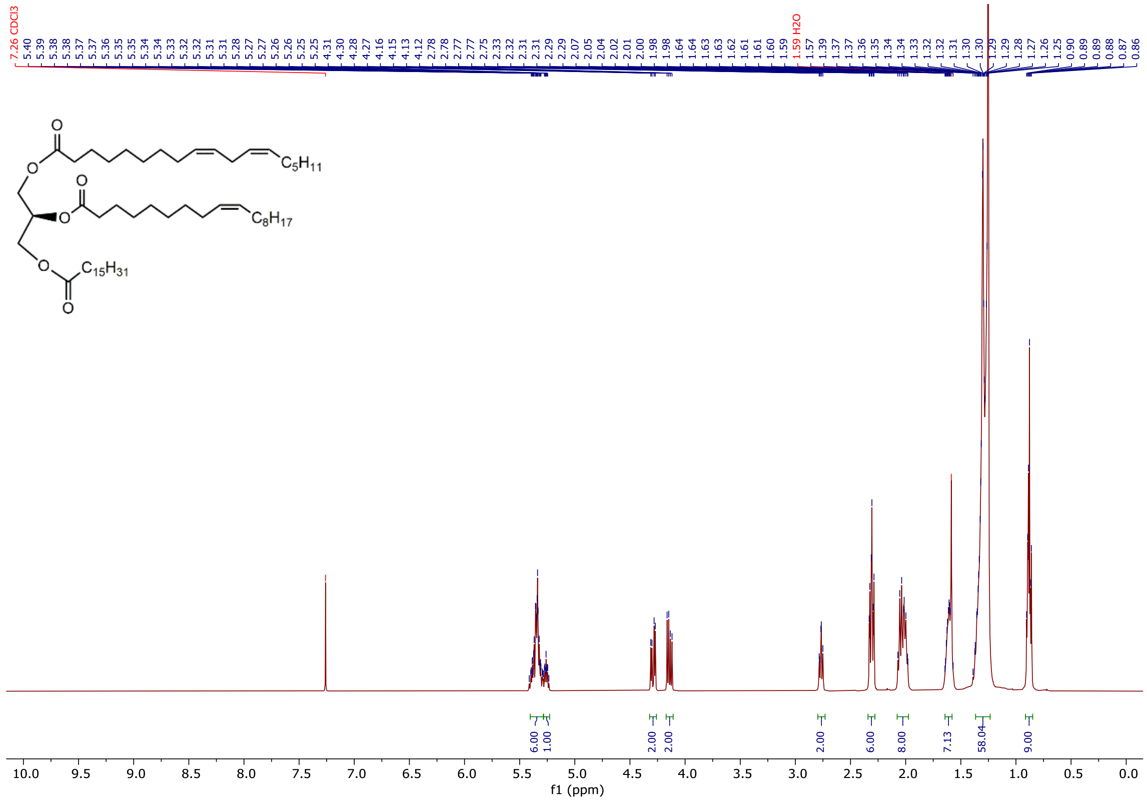

$^{13}C\{H\}$ NMR (101 MHz, $CDCl_3$) of compound (*S*)-**2**

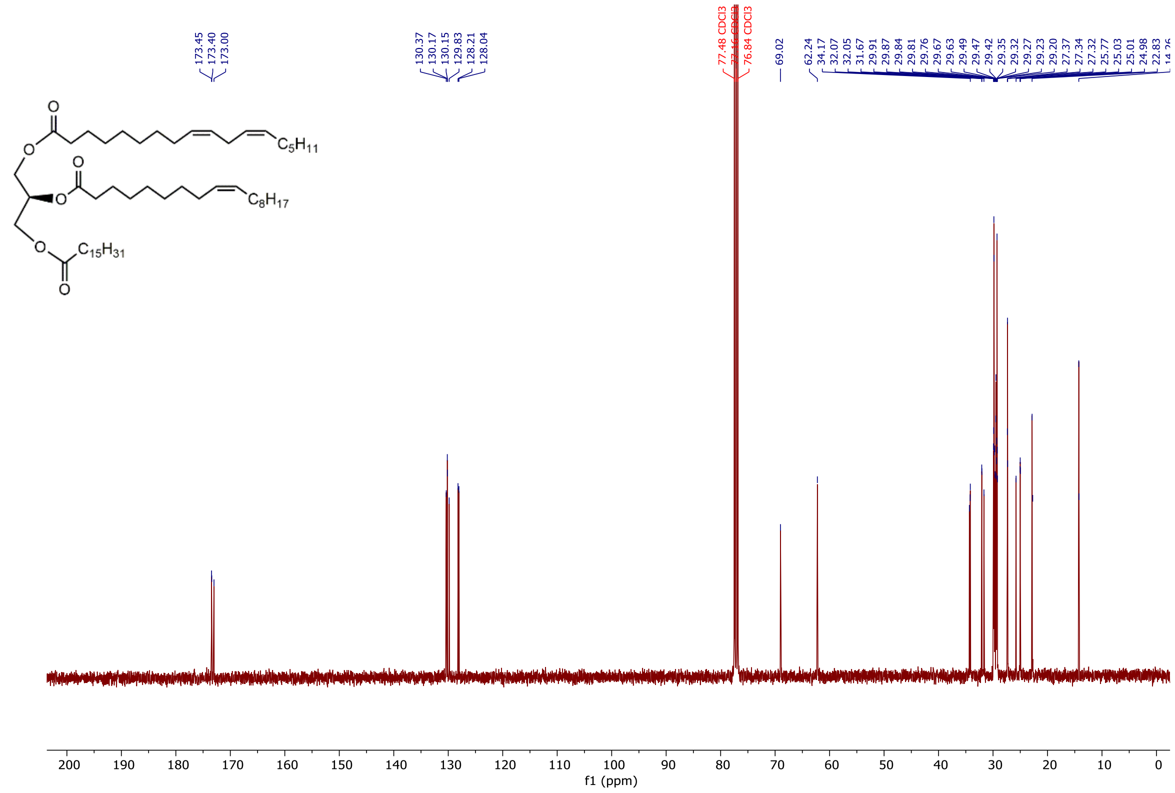

^{1}H-^{1}H COSY spectrum of compound (*S*)-**2**

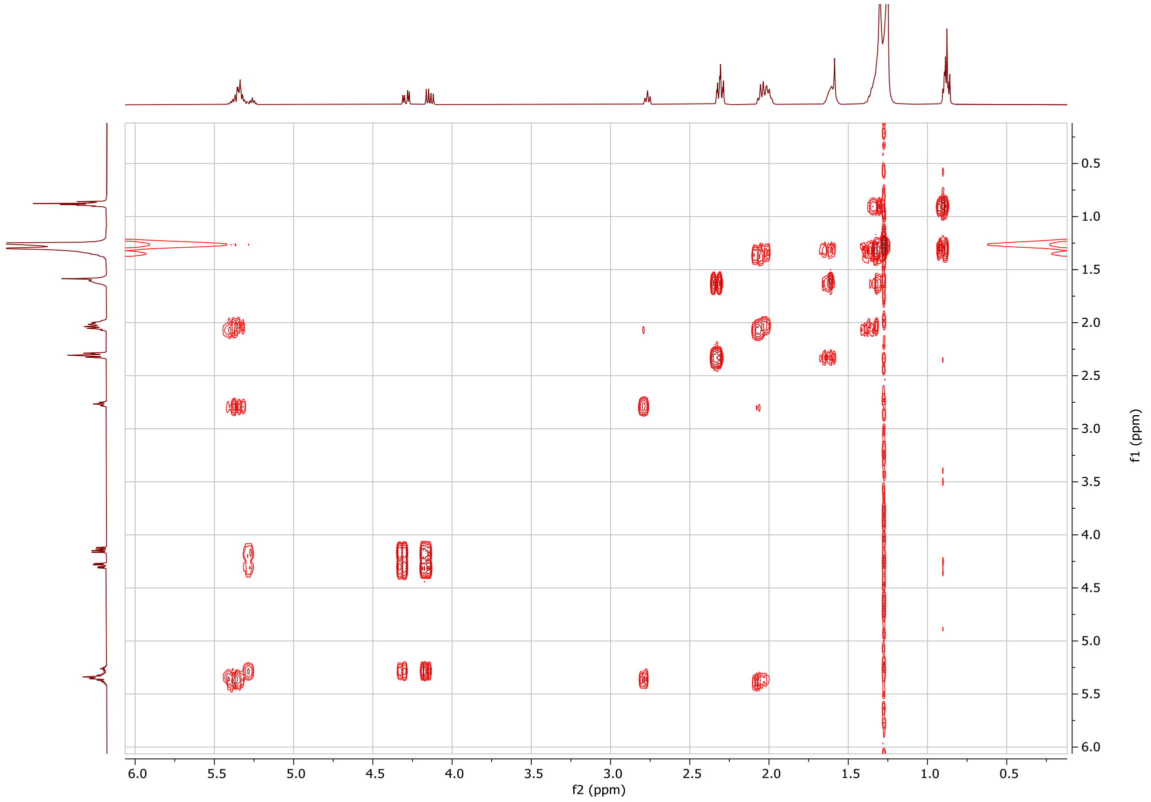

^{13}C-^{1}H HSQC spectrum of compound (*S*)-**2**

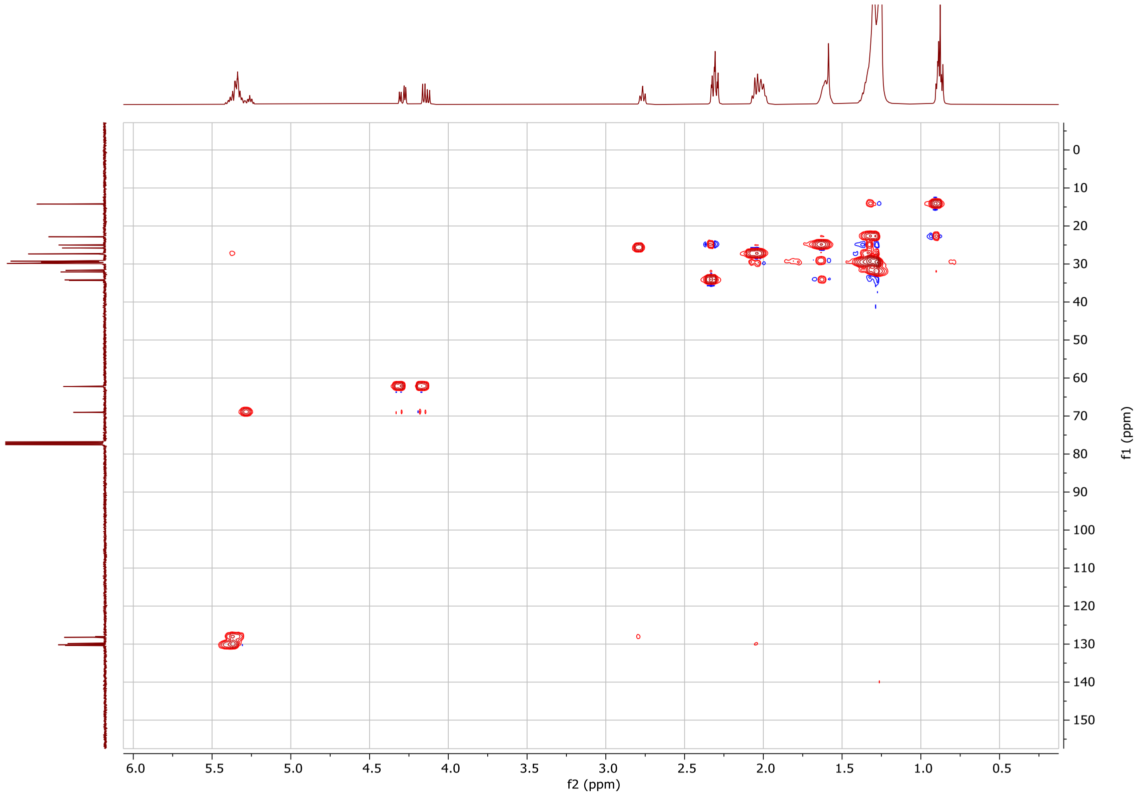

^{1}H NMR (400 MHz, $CDCl_3$) of compound (*R*)-**12a**

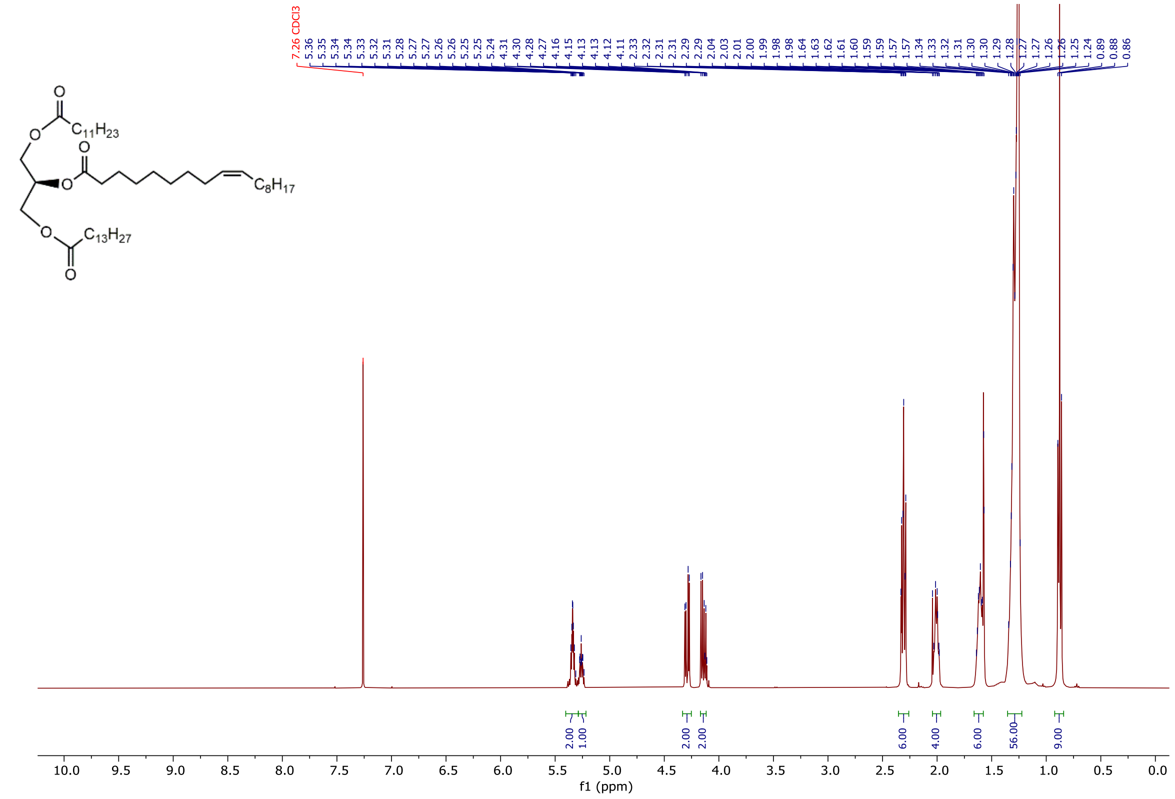

^{13}C{H} NMR (101 MHz, $CDCl_3$) of compound (*R*)-**12a**

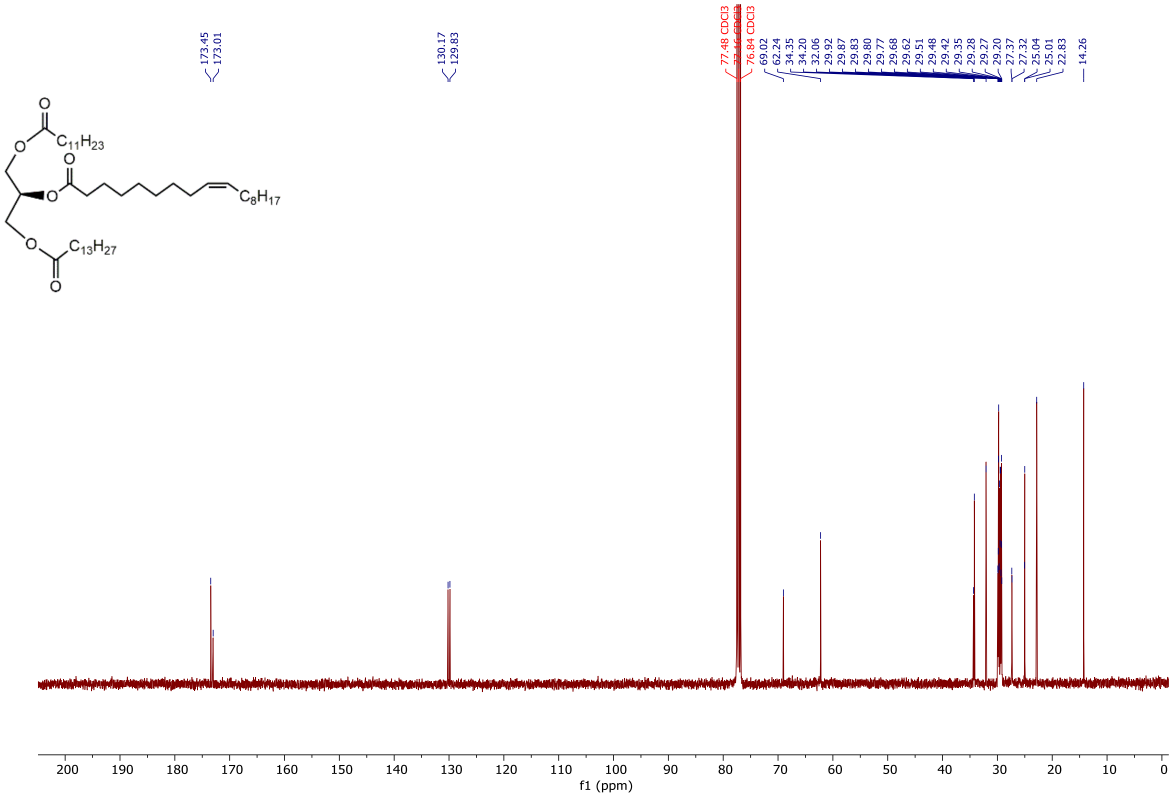

^{1}H-^{1}H COSY spectrum of compound (*R*)-**12a**

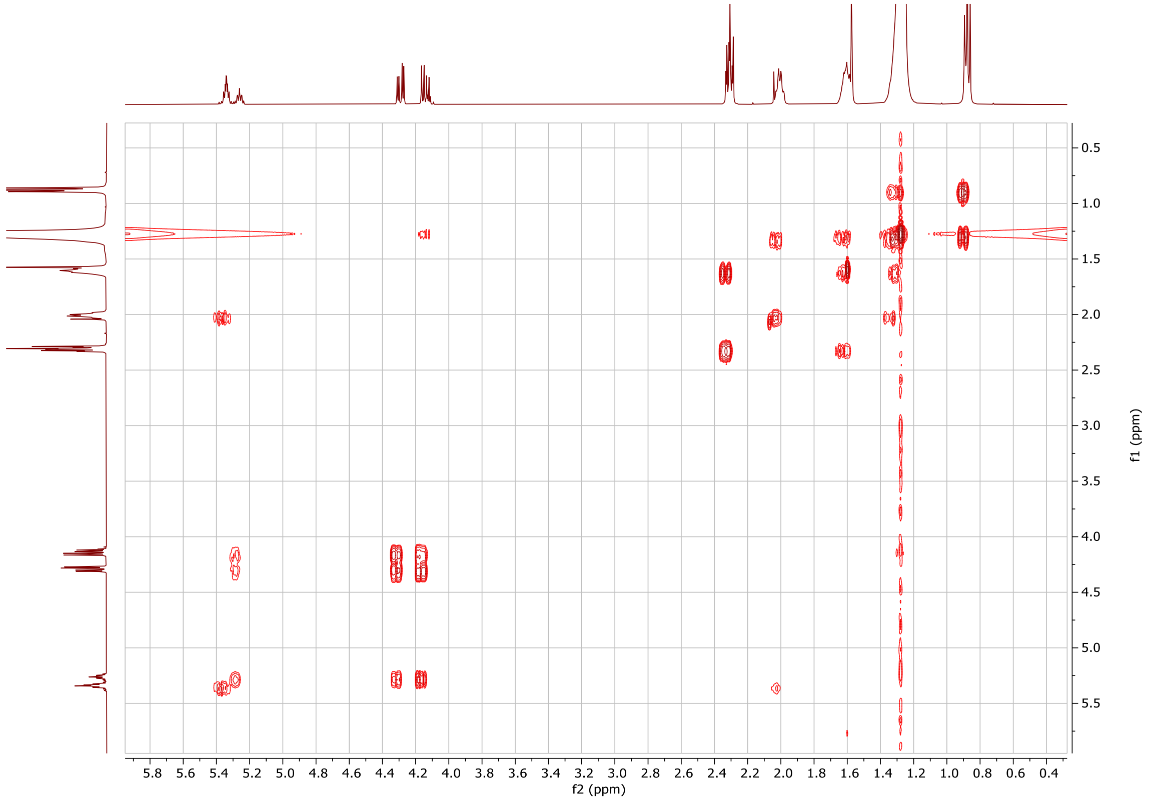

^{13}C-^{1}H HSQC spectrum of compound (*R*)-**12a**

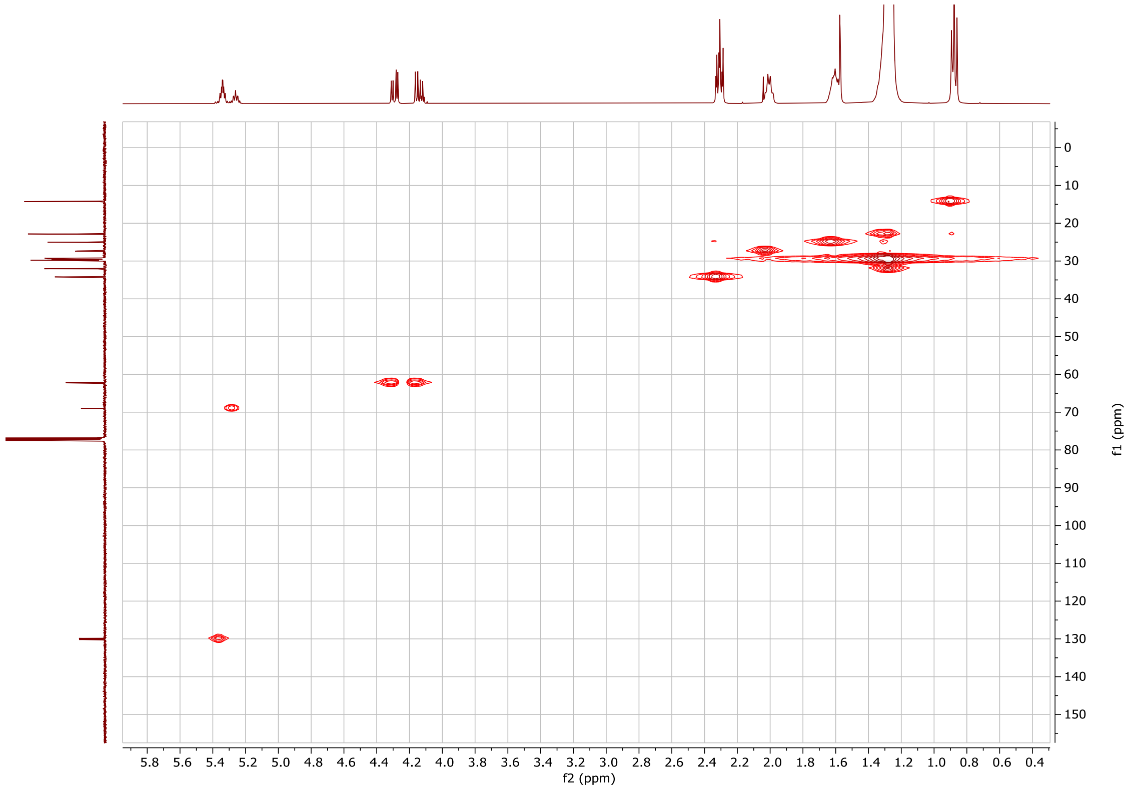

^{1}H NMR (400 MHz, $CDCl_3$) of compound (*R*)-**12b**

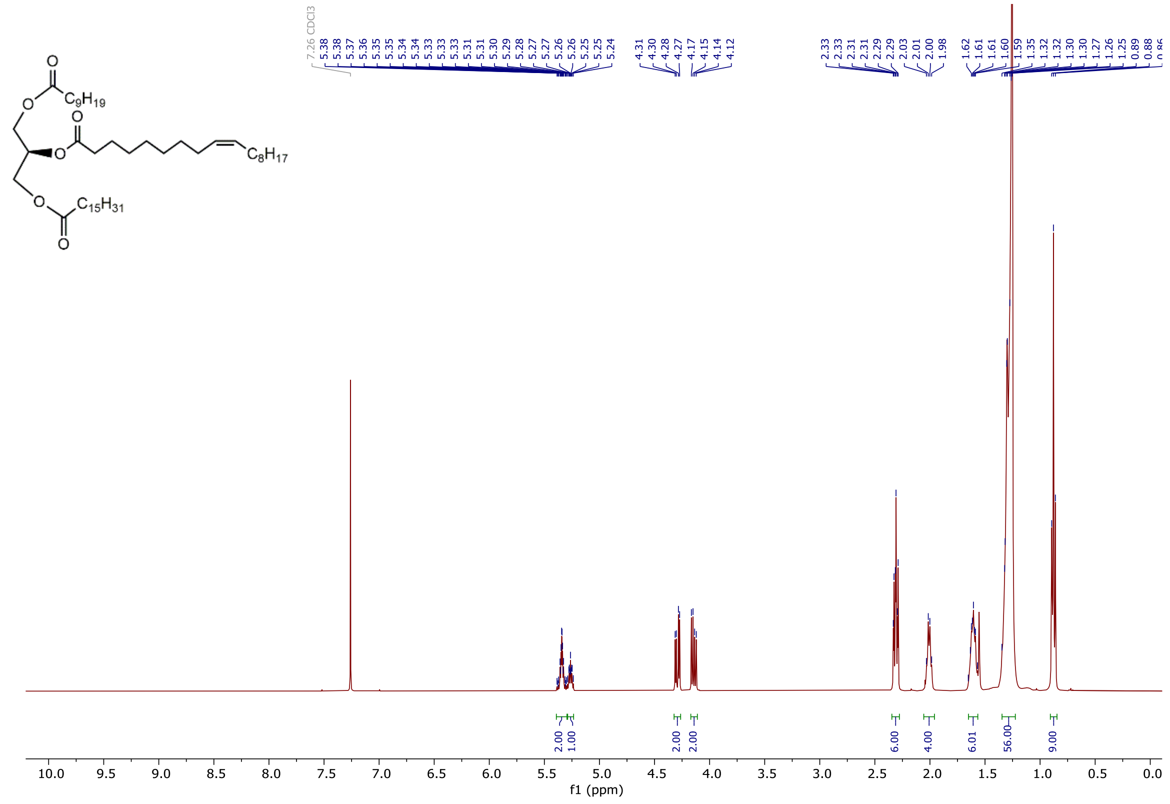

$^{13}C\{H\}$ NMR (101 MHz, $CDCl_3$) of compound (*R*)-**12b**

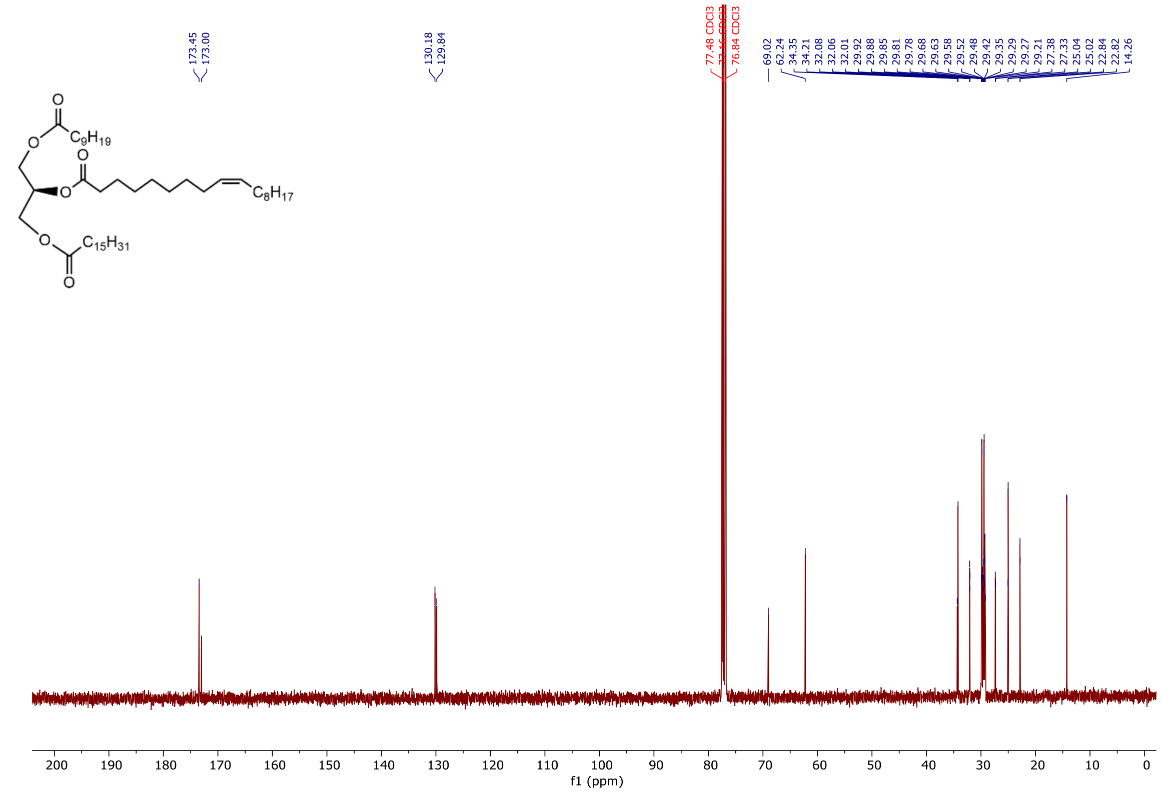

^{1}H-^{1}H COSY spectrum of compound (*R*)-**12b**

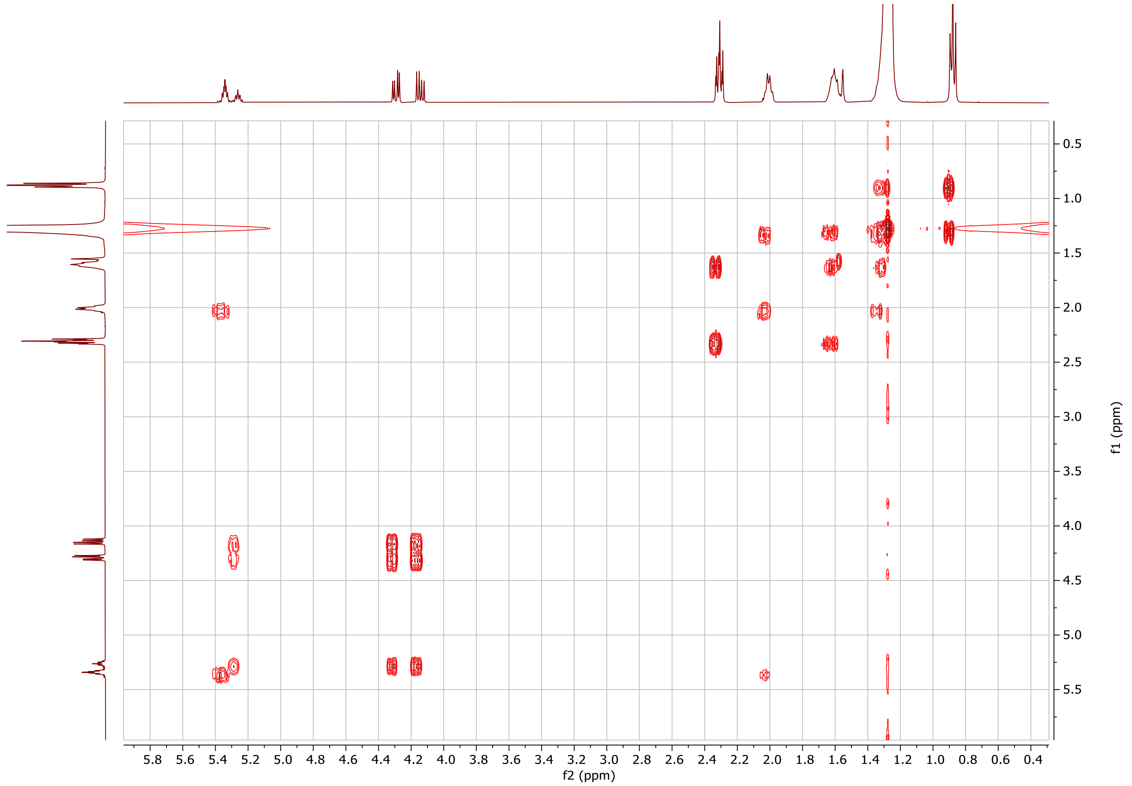

^{13}C-^{1}H HSQC spectrum of compound (*R*)-**12b**

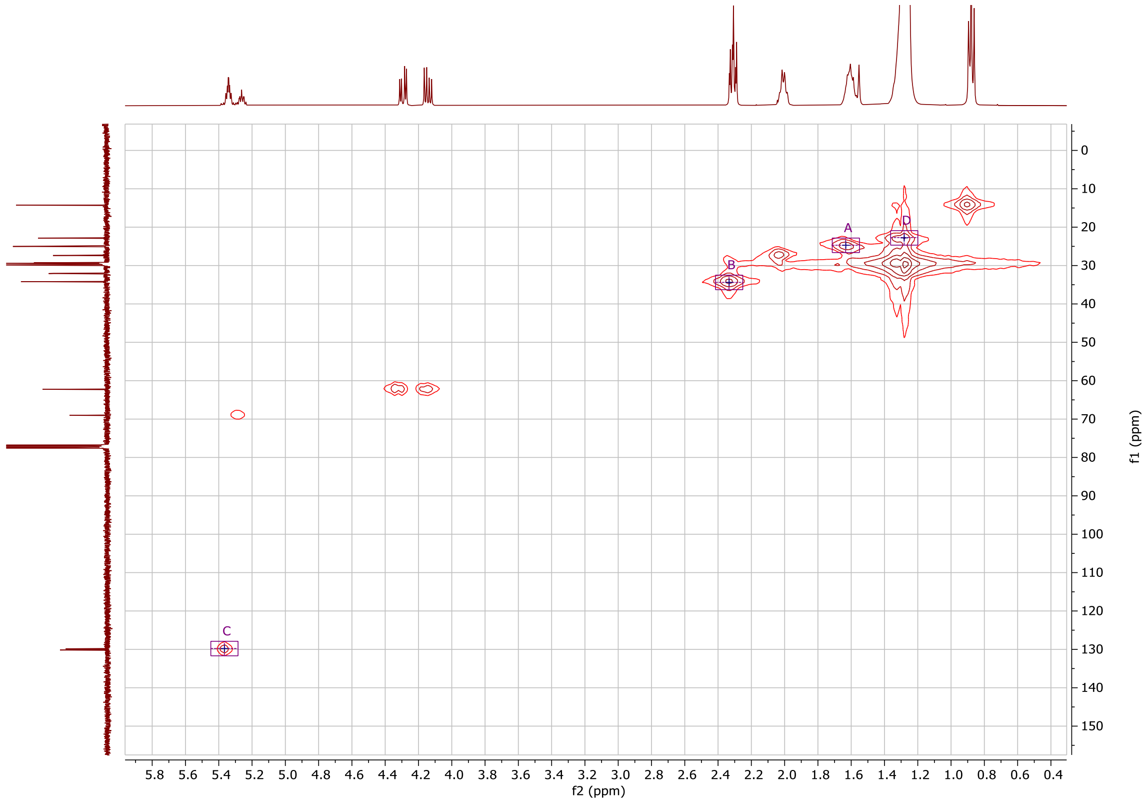

^{1}H NMR (400 MHz, $CDCl_3$) of compound (*R*)-**12c**

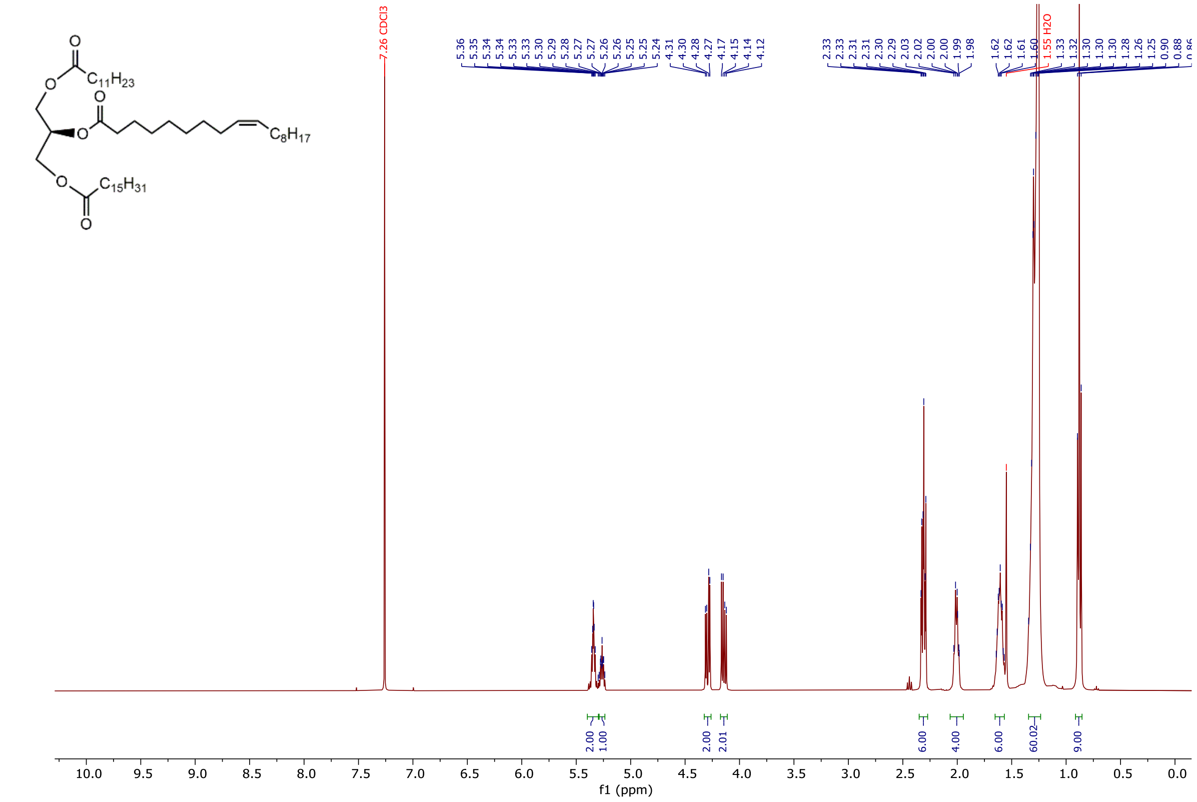

^{13}C{H} NMR (101 MHz, $CDCl_3$) of compound (*R*)-**12c**

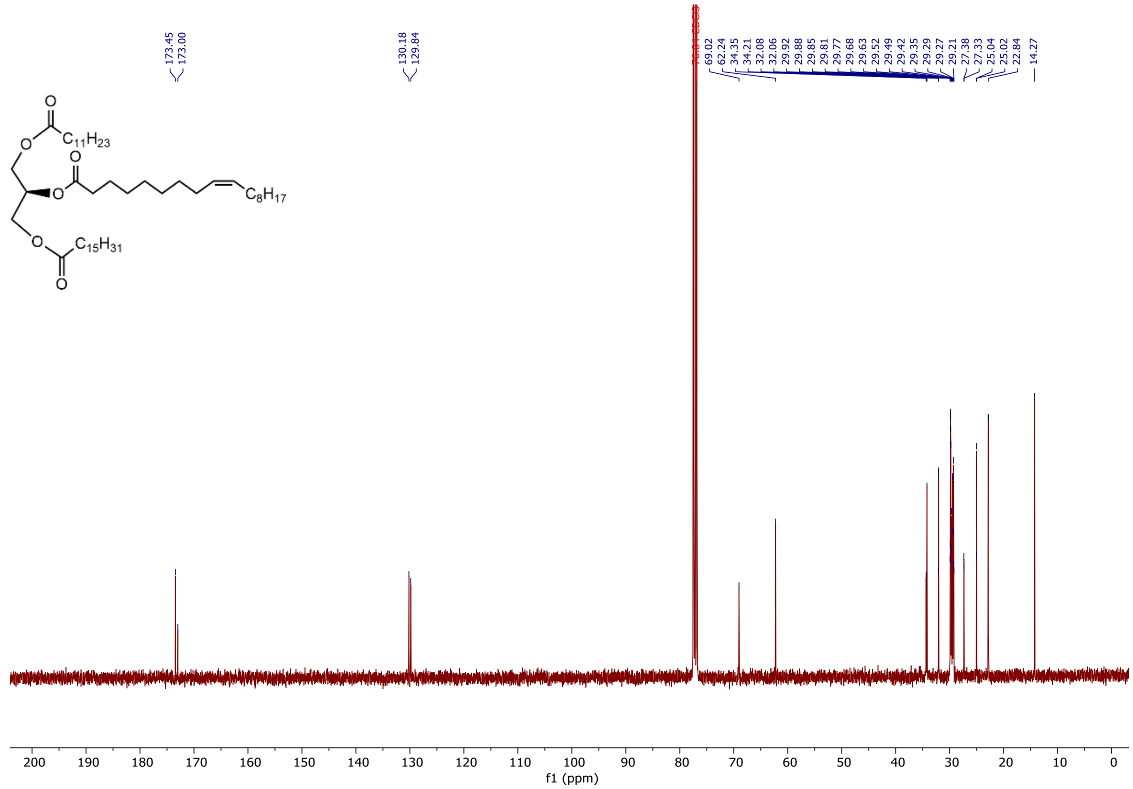

^{1}H-^{1}H COSY spectrum of compound (*R*)-**12c**

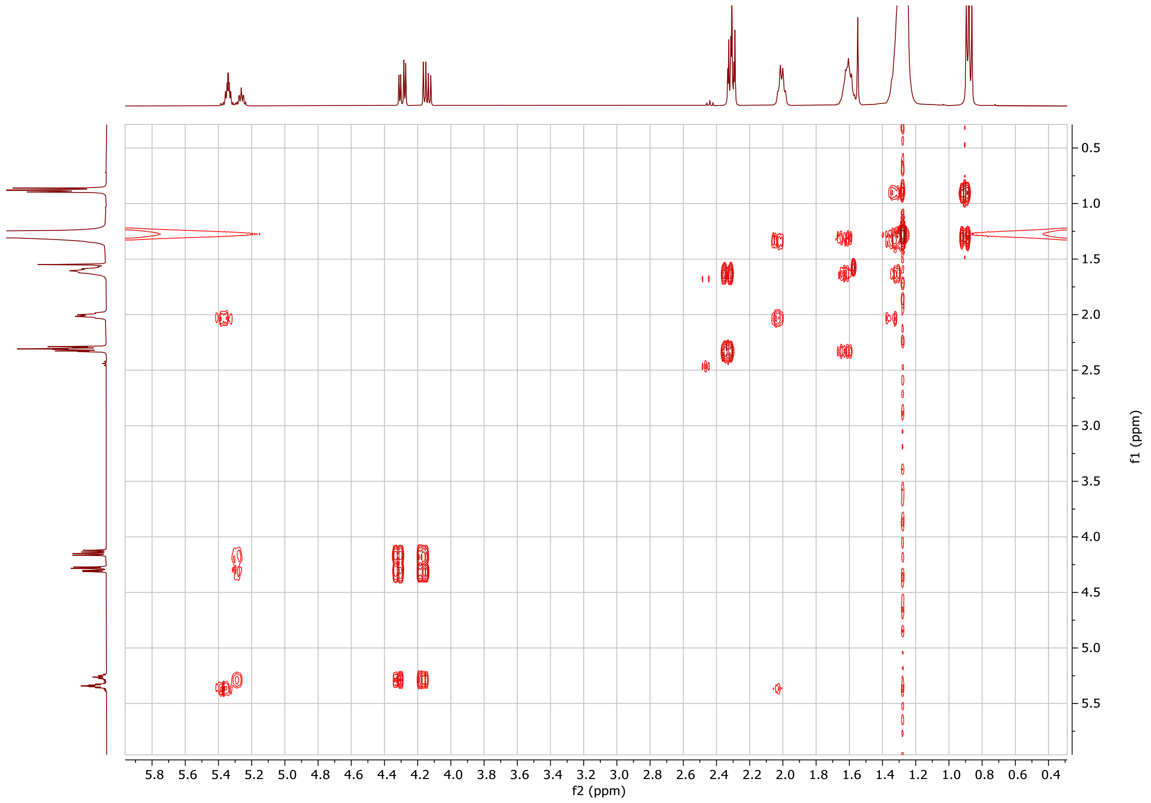

^{13}C-^{1}H HSQC spectrum of compound (*R*)-**12c**

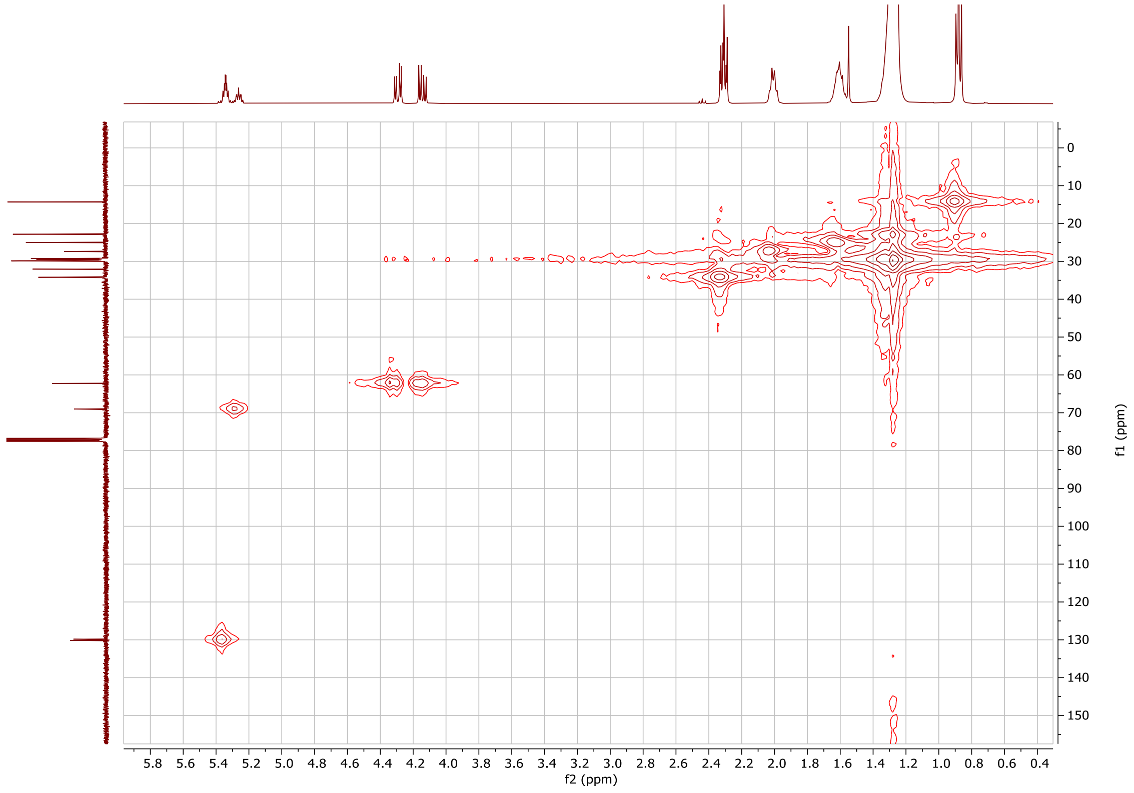

^{1}H NMR (400 MHz, $CDCl_3$) of compound (*R*)-**12d**

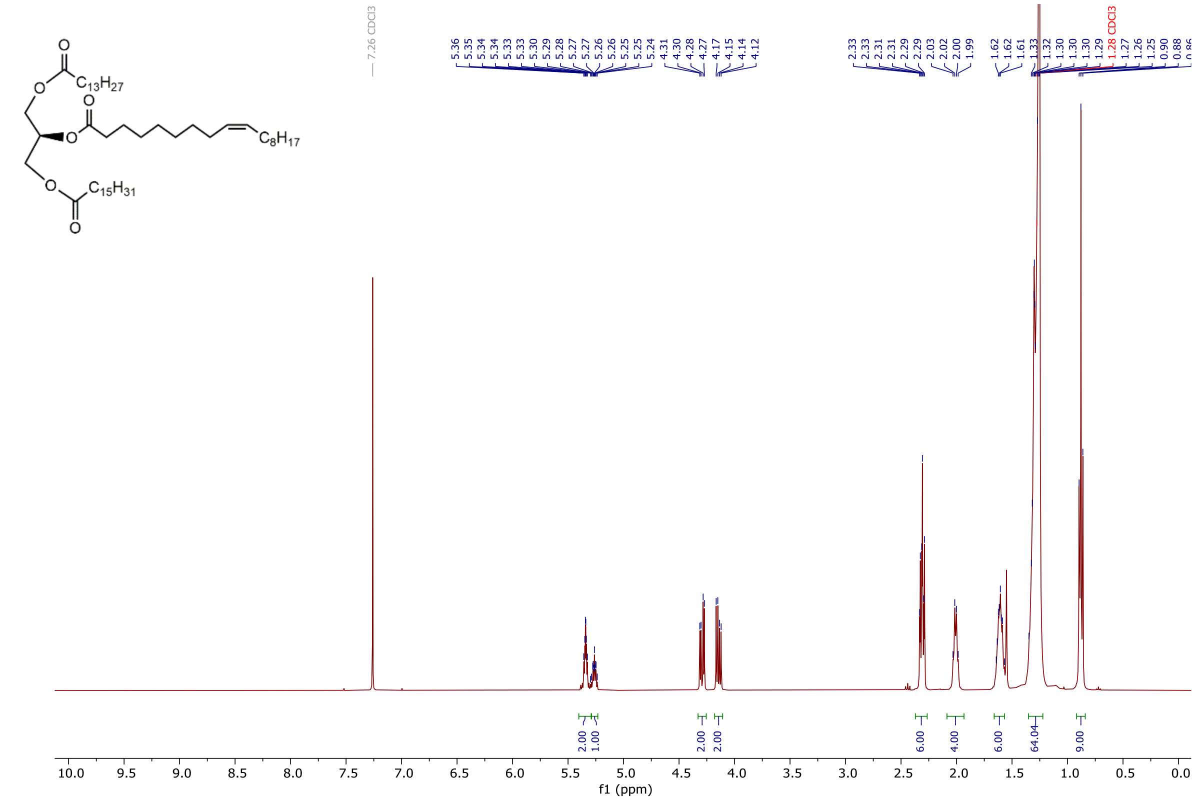

^{13}C{H} NMR (101 MHz, $CDCl_3$) of compound (*R*)-**12d**

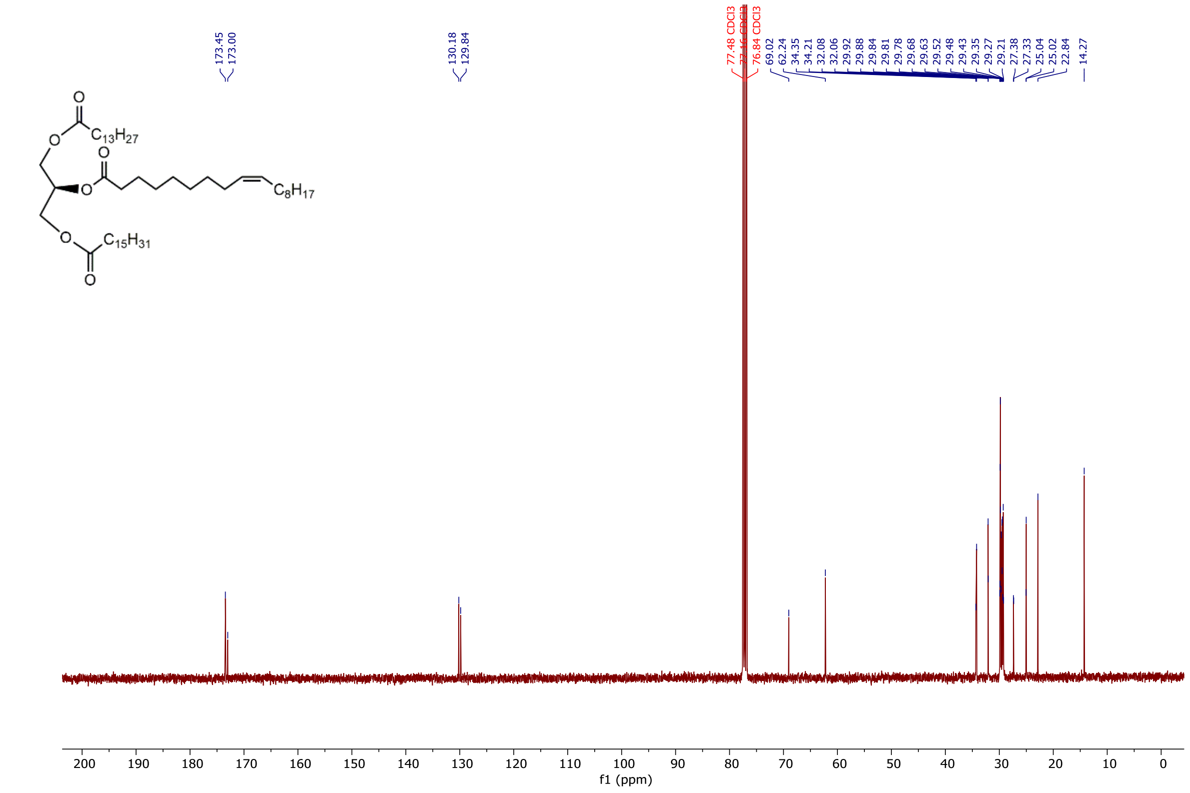

^{1}H-^{1}H COSY spectrum of compound (*R*)-**12d**

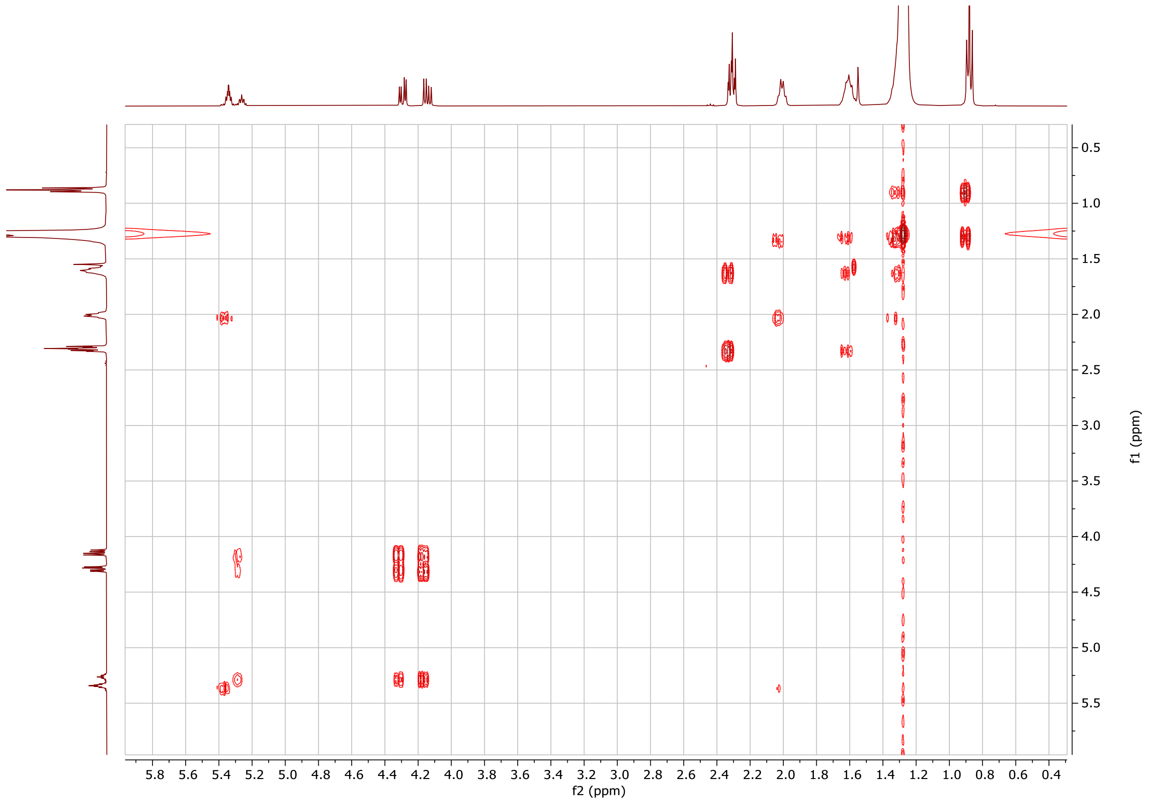

^{13}C-^{1}H HSQC spectrum of compound (*R*)-**12d**

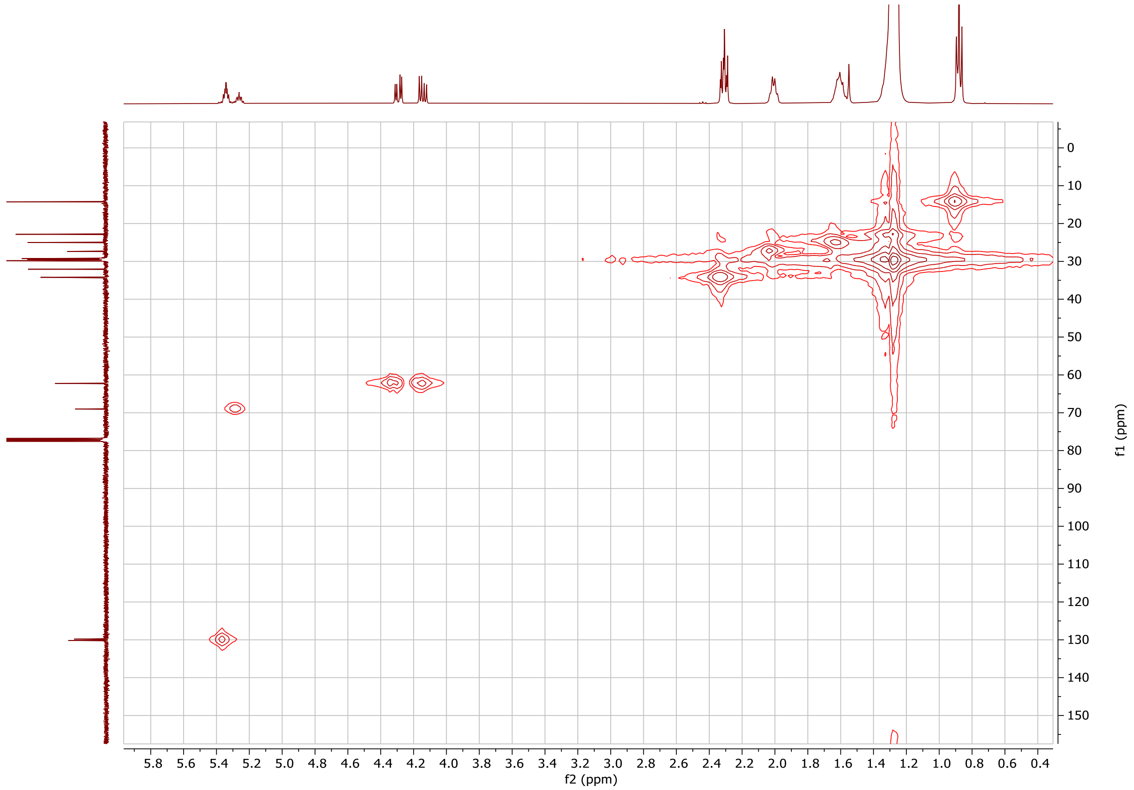

^{1}H NMR (400 MHz, $CDCl_3$) of compound (*S*)-**12e**

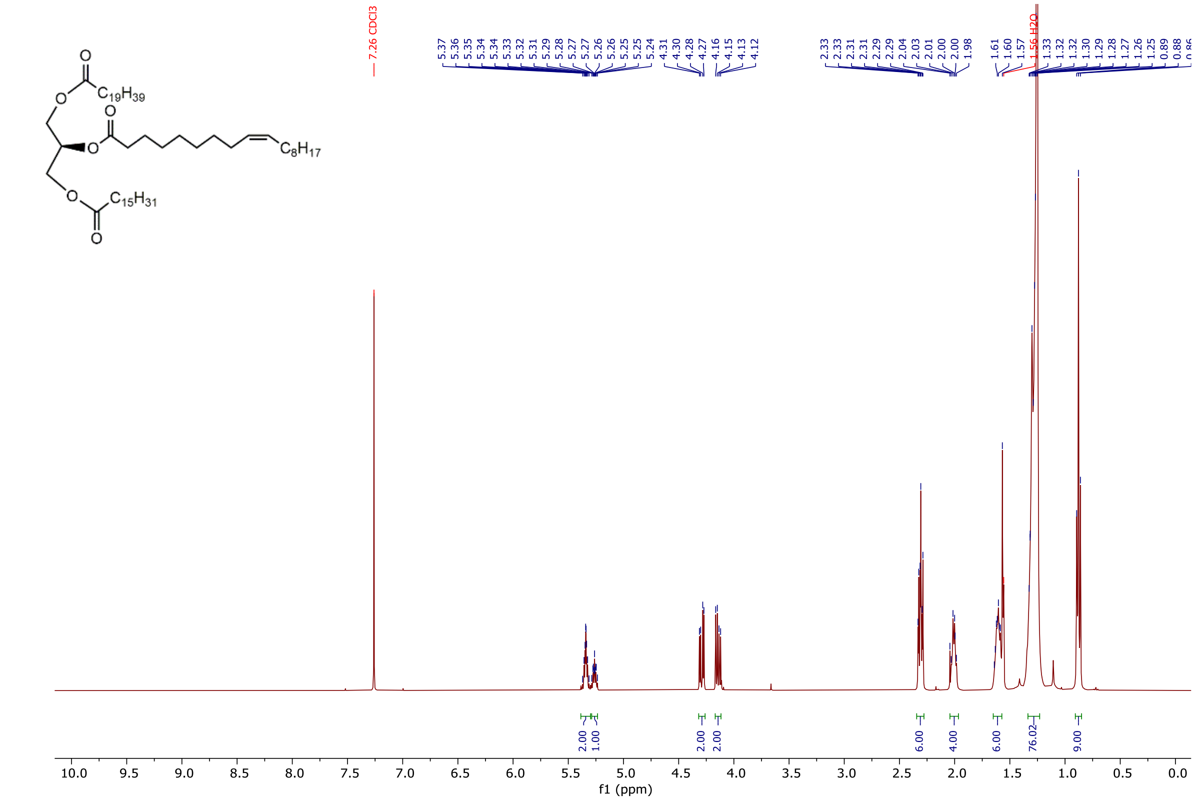

^{13}C{H} NMR (101 MHz, $CDCl_3$) of compound (*S*)-**12e**

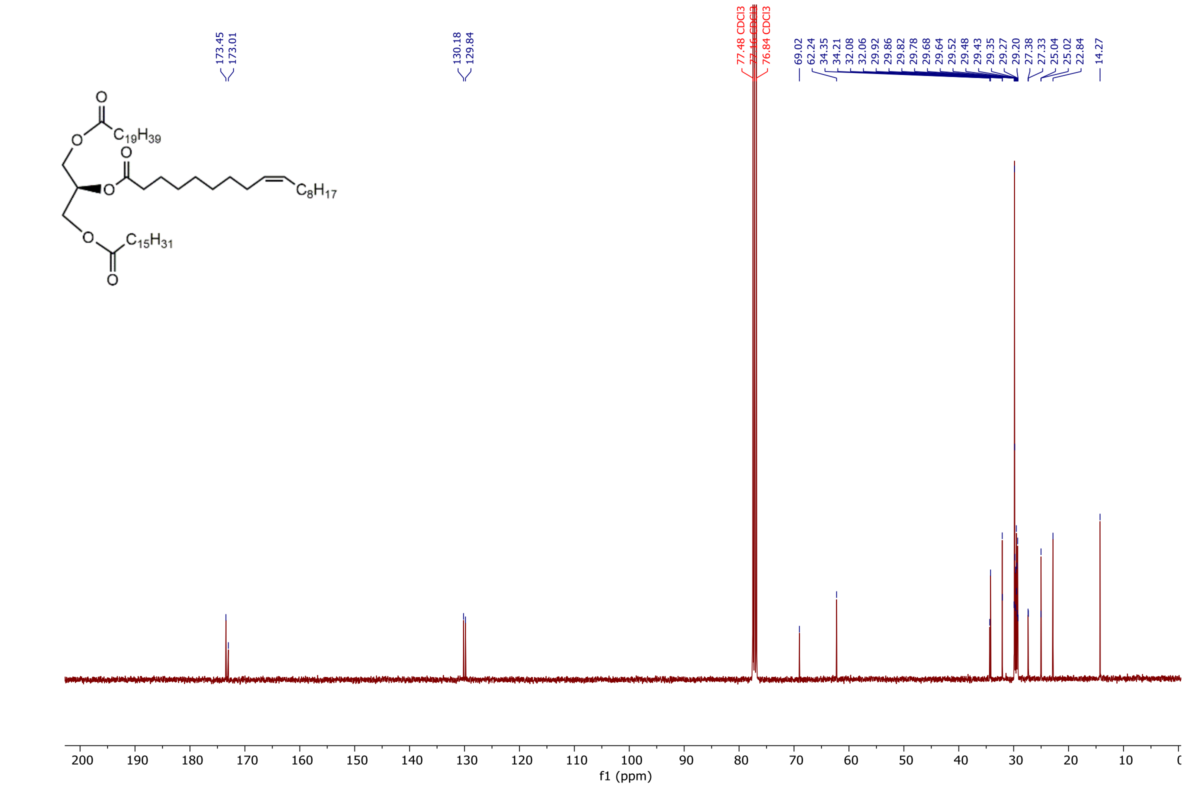

^{1}H-^{1}H COSY spectrum of compound (*S*)-**12e**

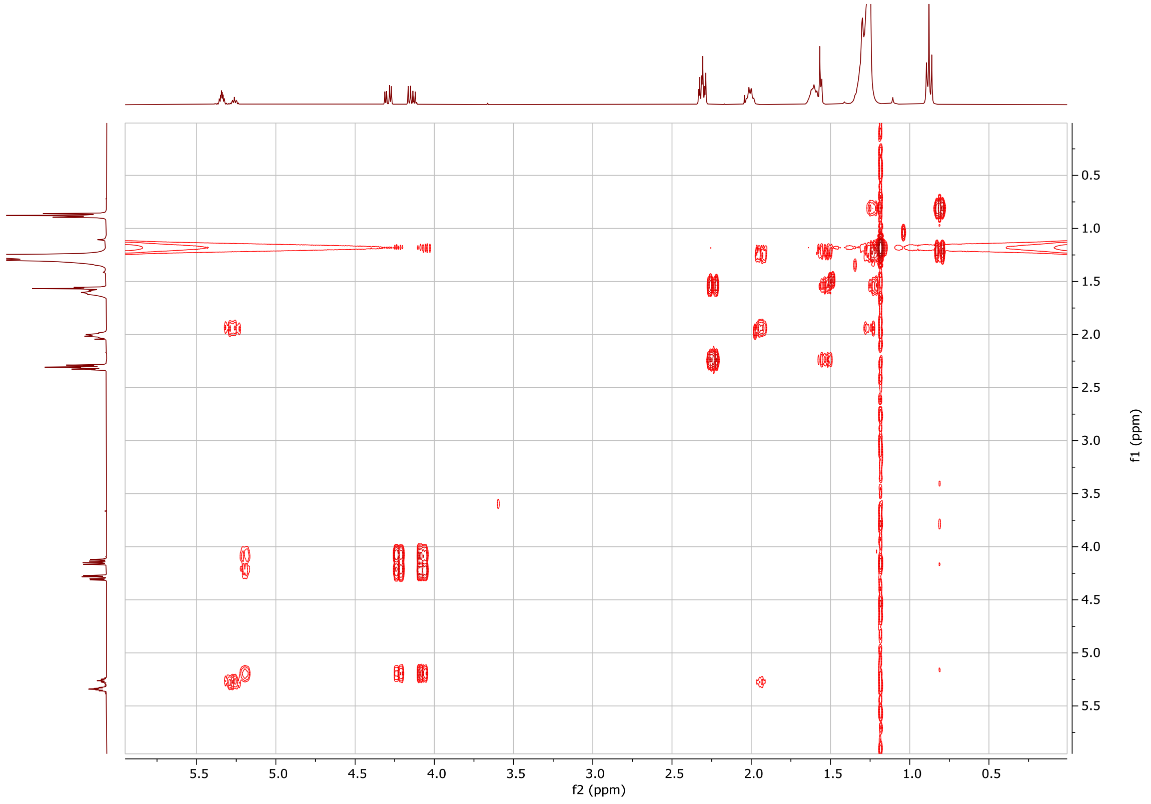

^{13}C-^{1}H HSQC spectrum of compound (*S*)-**12e**

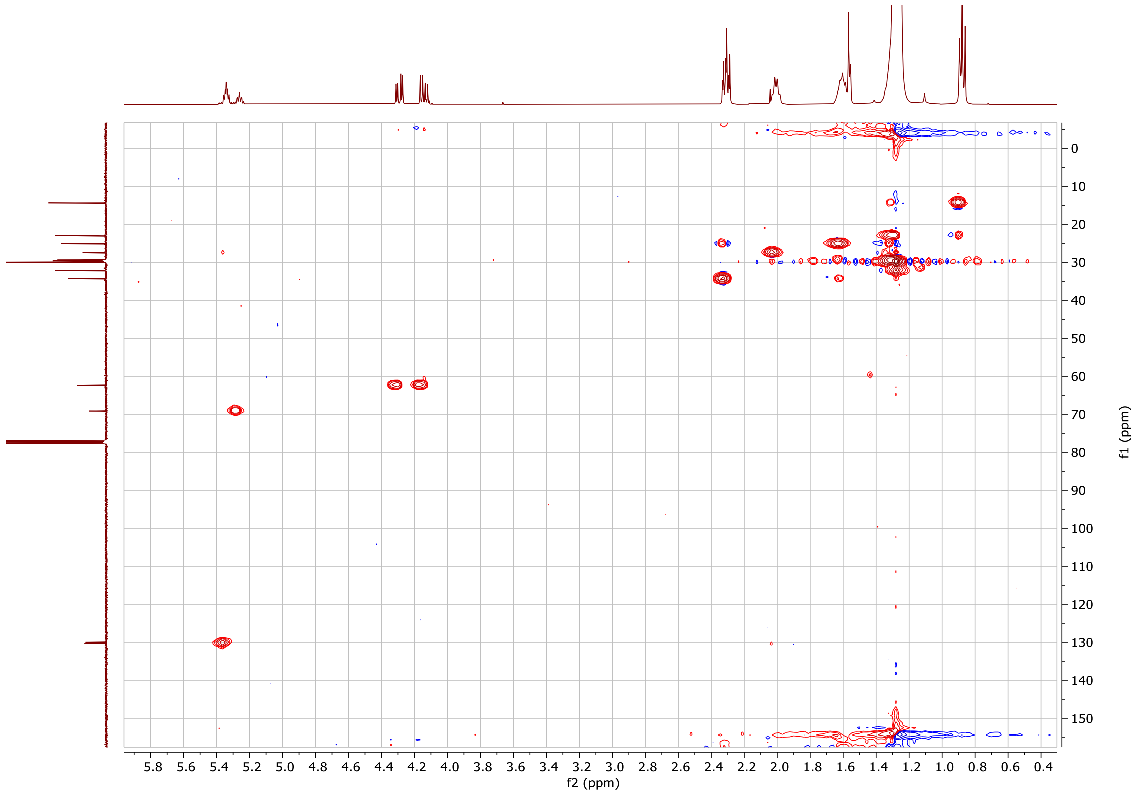

^{1}H NMR (400 MHz, $CDCl_3$) of compound (*R*)-**12f**

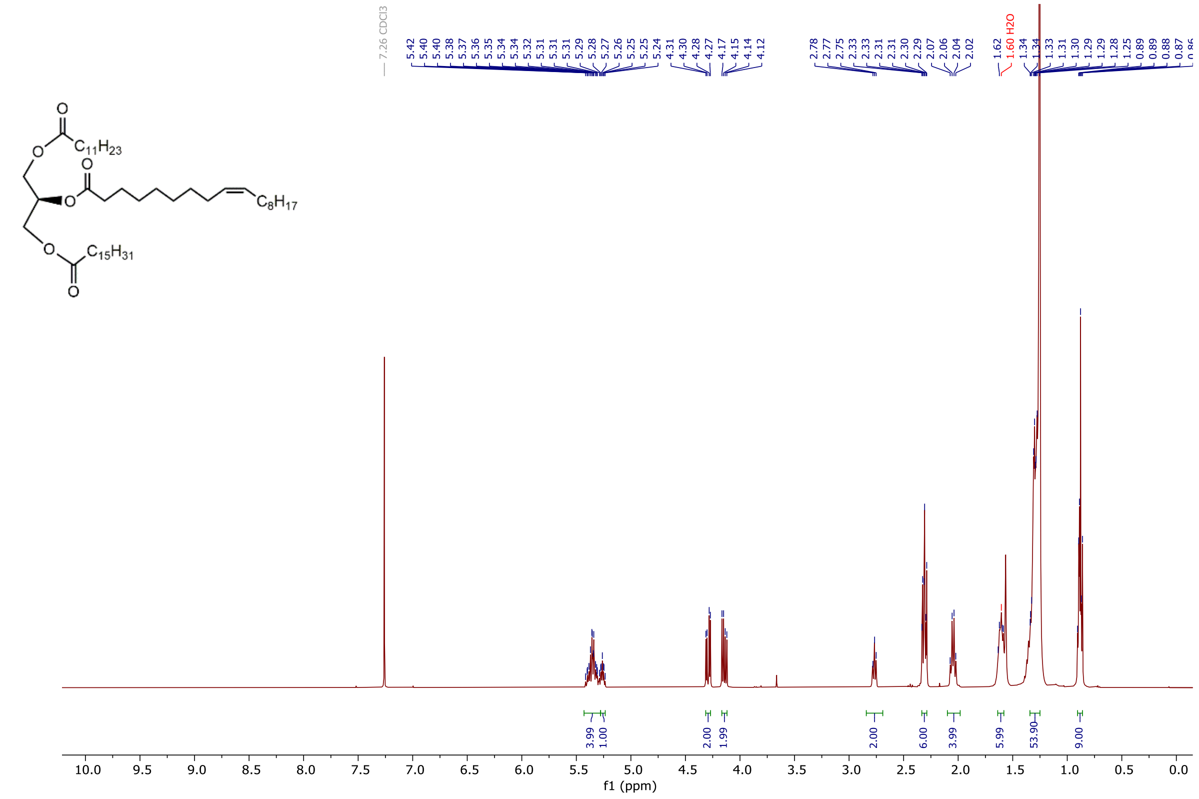

^{13}C{H} NMR (101 MHz, $CDCl_3$) of compound (*R*)-**12f**

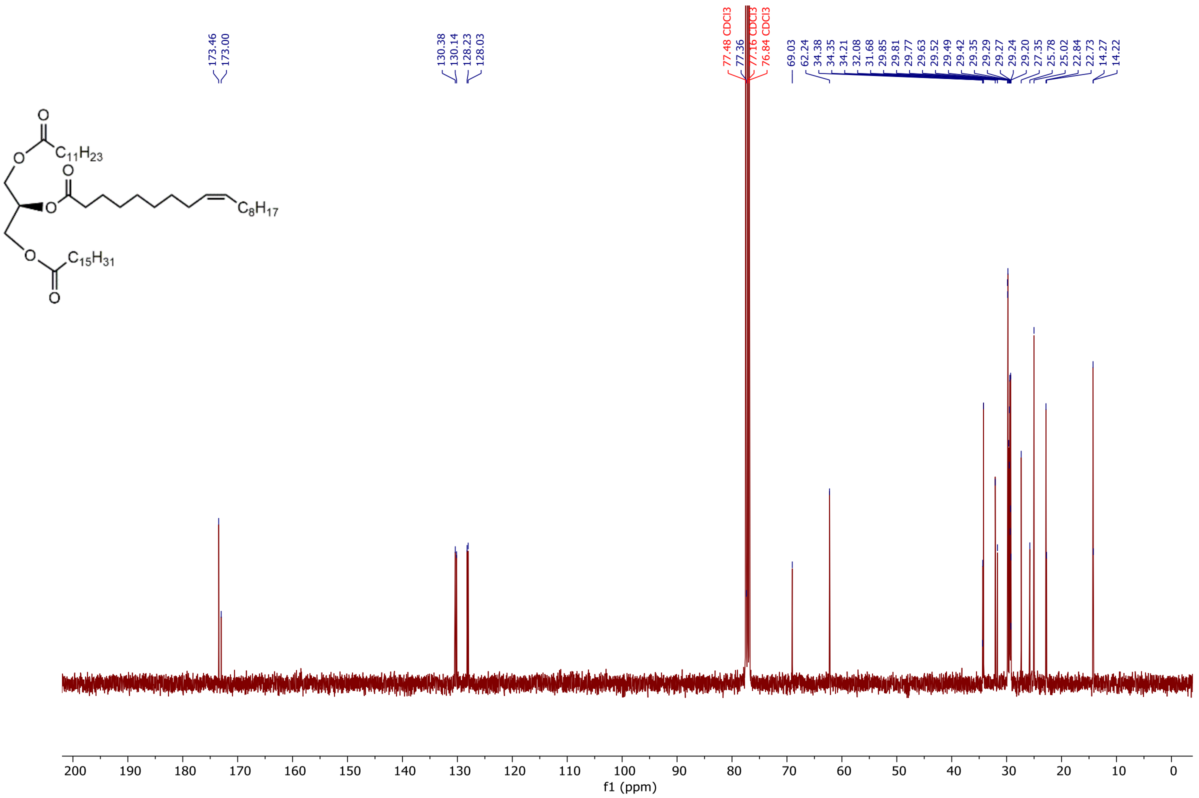

^{1}H-^{1}H COSY spectrum of compound (*R*)-**12f**

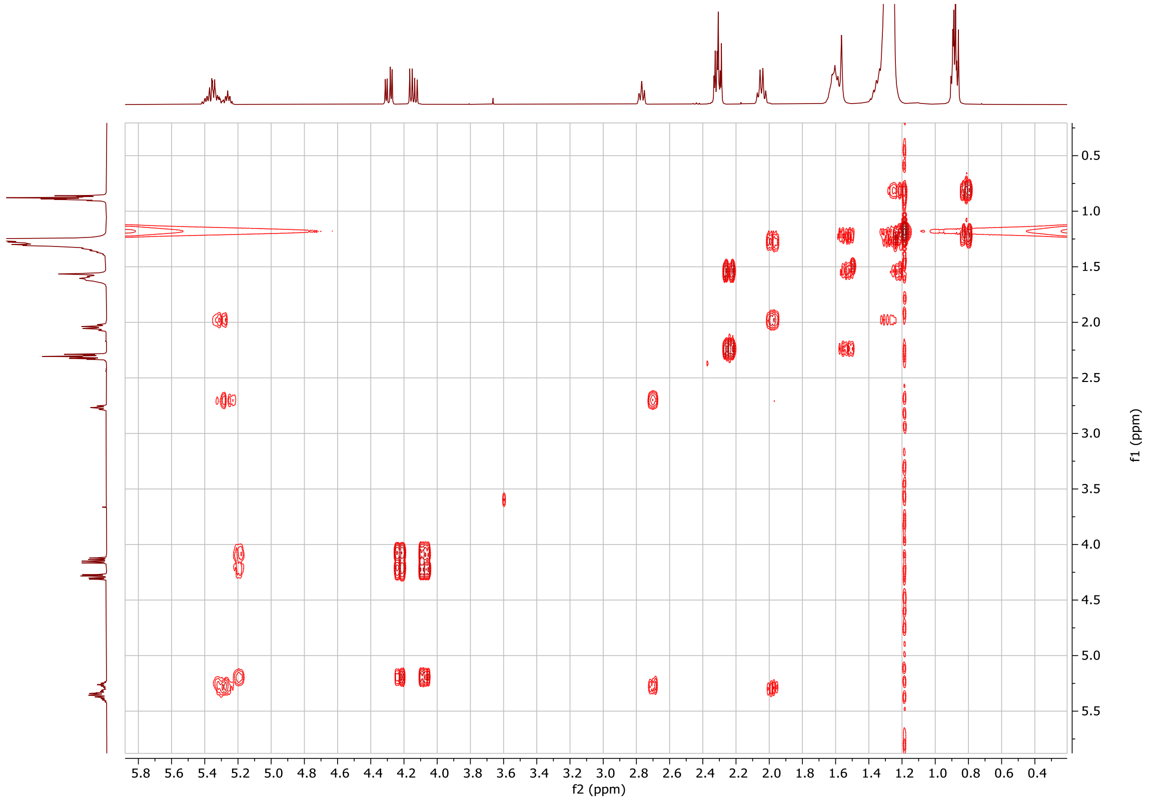

^{13}C-^{1}H HSQC spectrum of compound (*R*)-**12f**

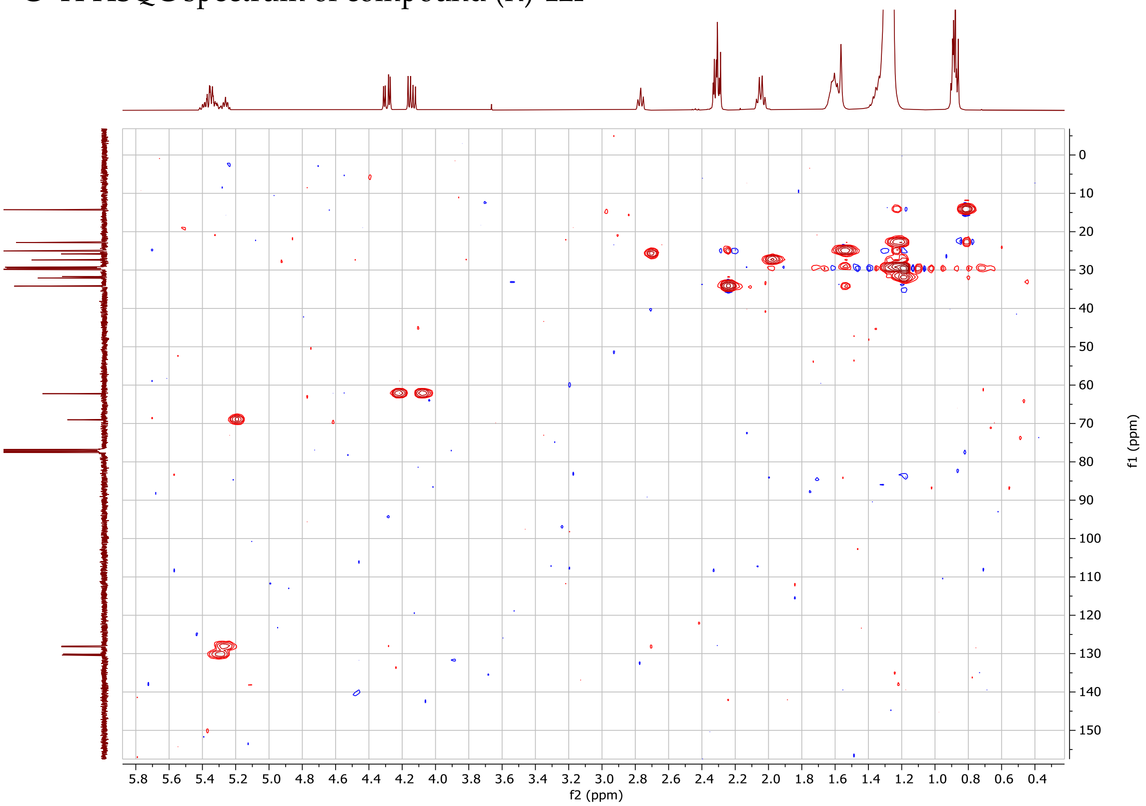

^{1}H NMR (400 MHz, $CDCl_3$) of compound **13**

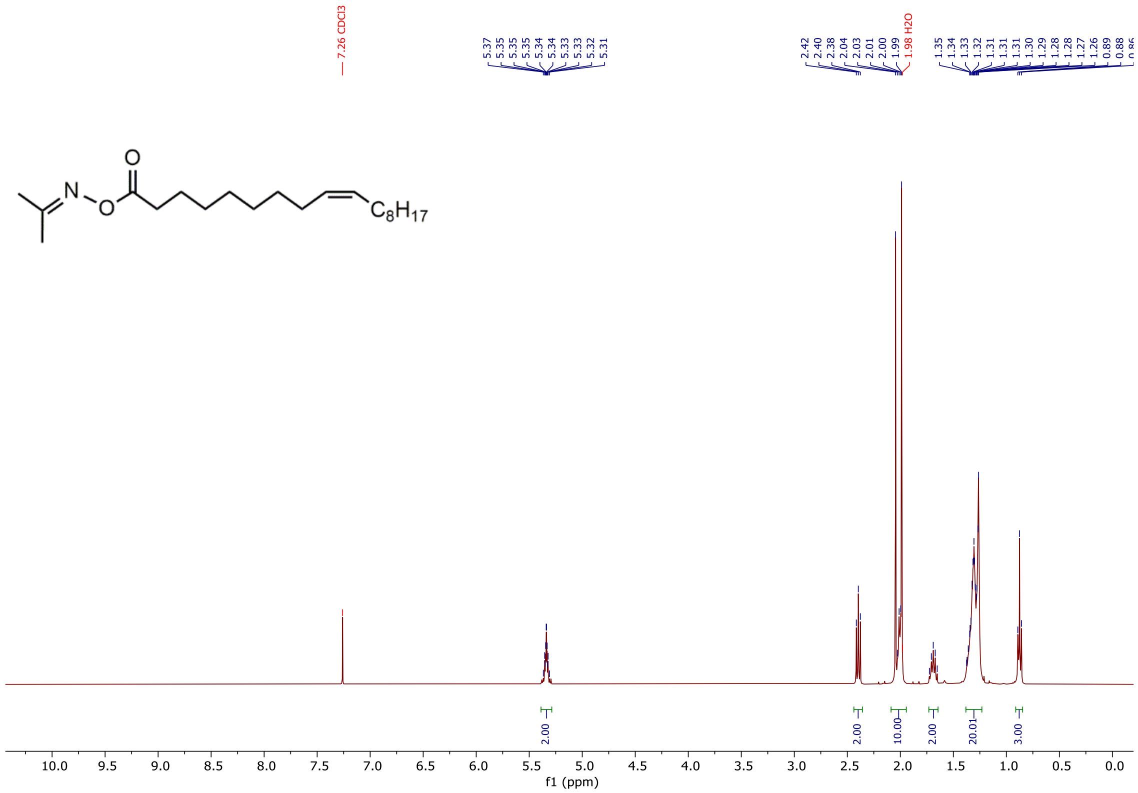

^{13}C{H} NMR (101 MHz, $CDCl_3$) of compound **13**

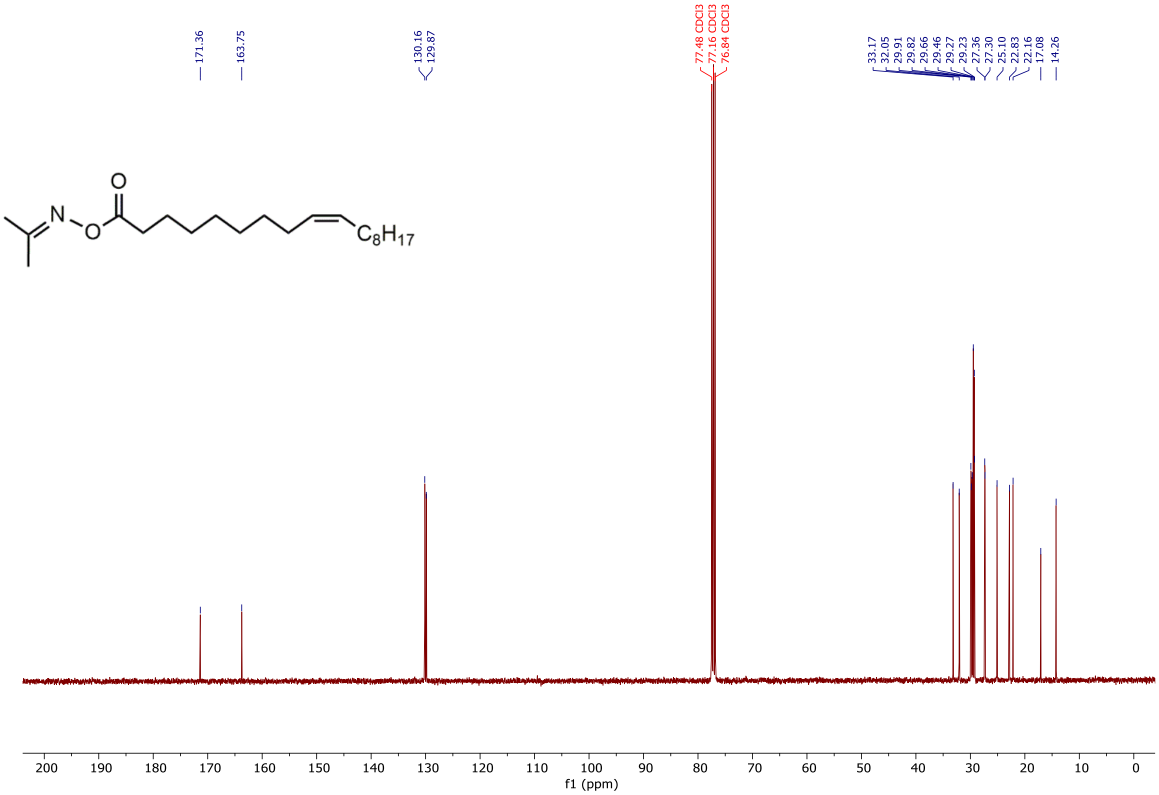

^{1}H-^{1}H COSY spectrum of compound **13**

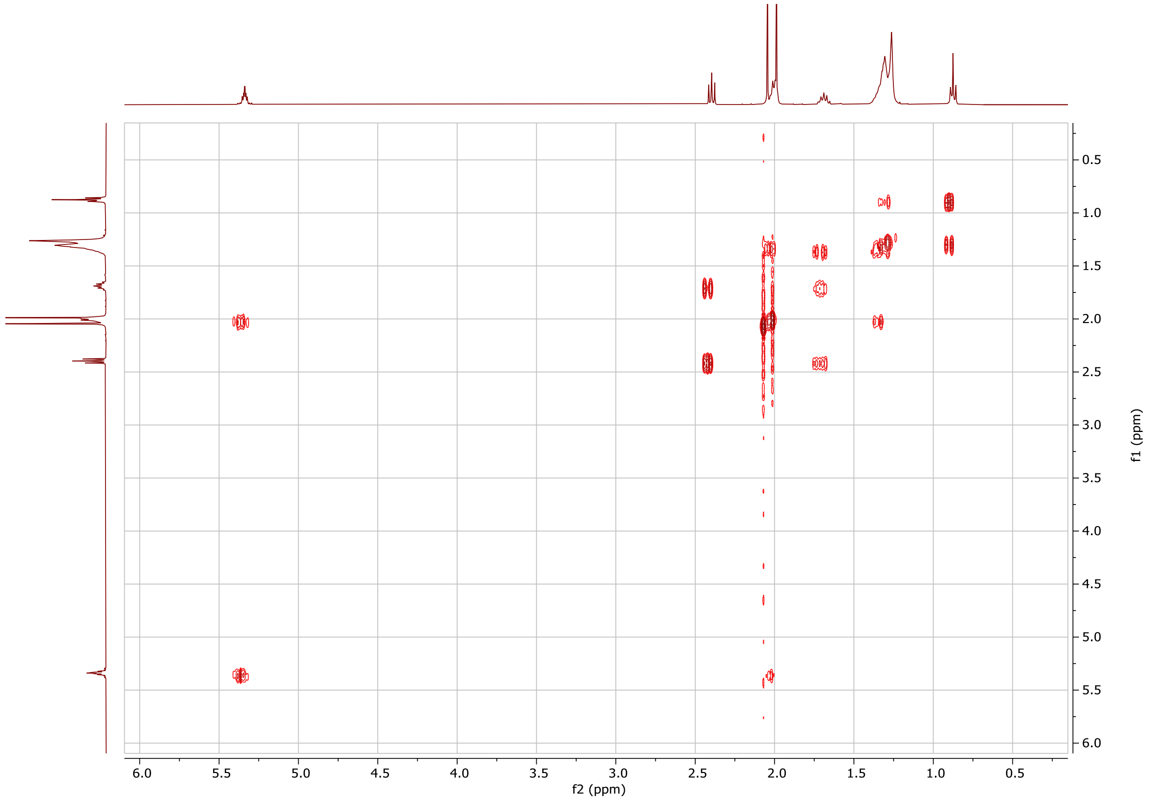

^{13}C-^{1}H HSQC spectrum of compound **13**

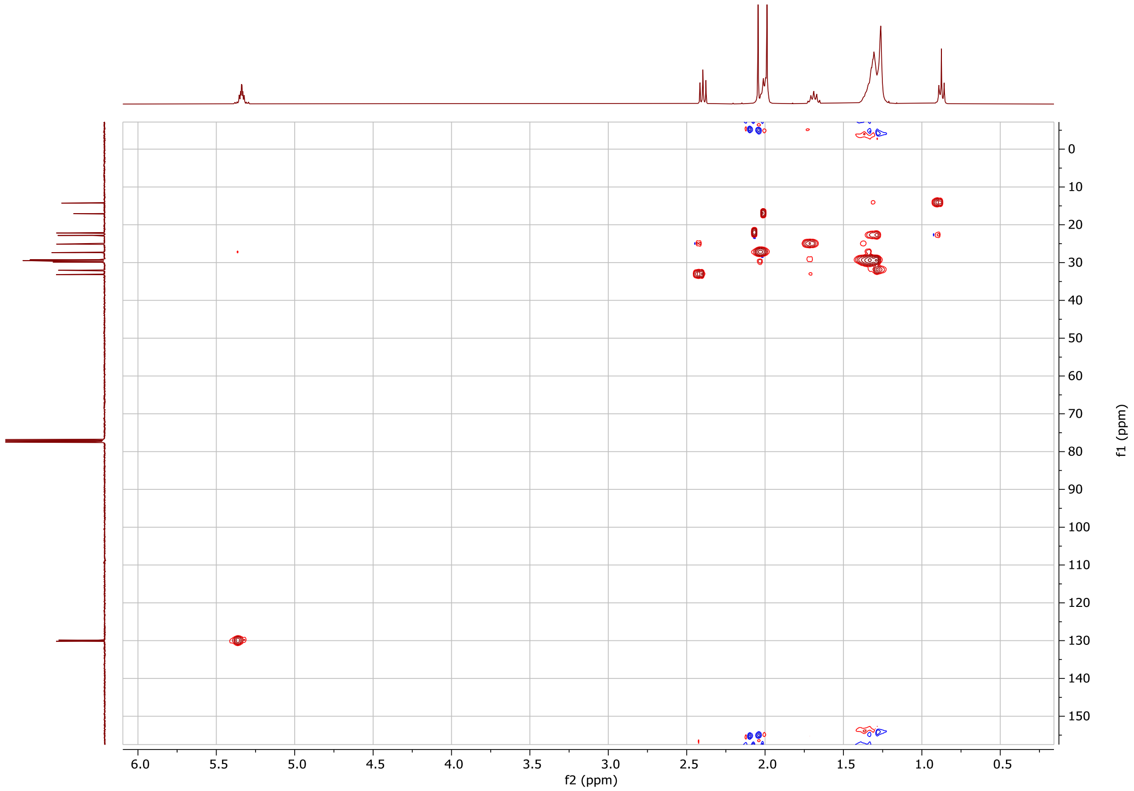

^{1}H NMR (400 MHz, $CDCl_3$) of compound (*R*)-**14**

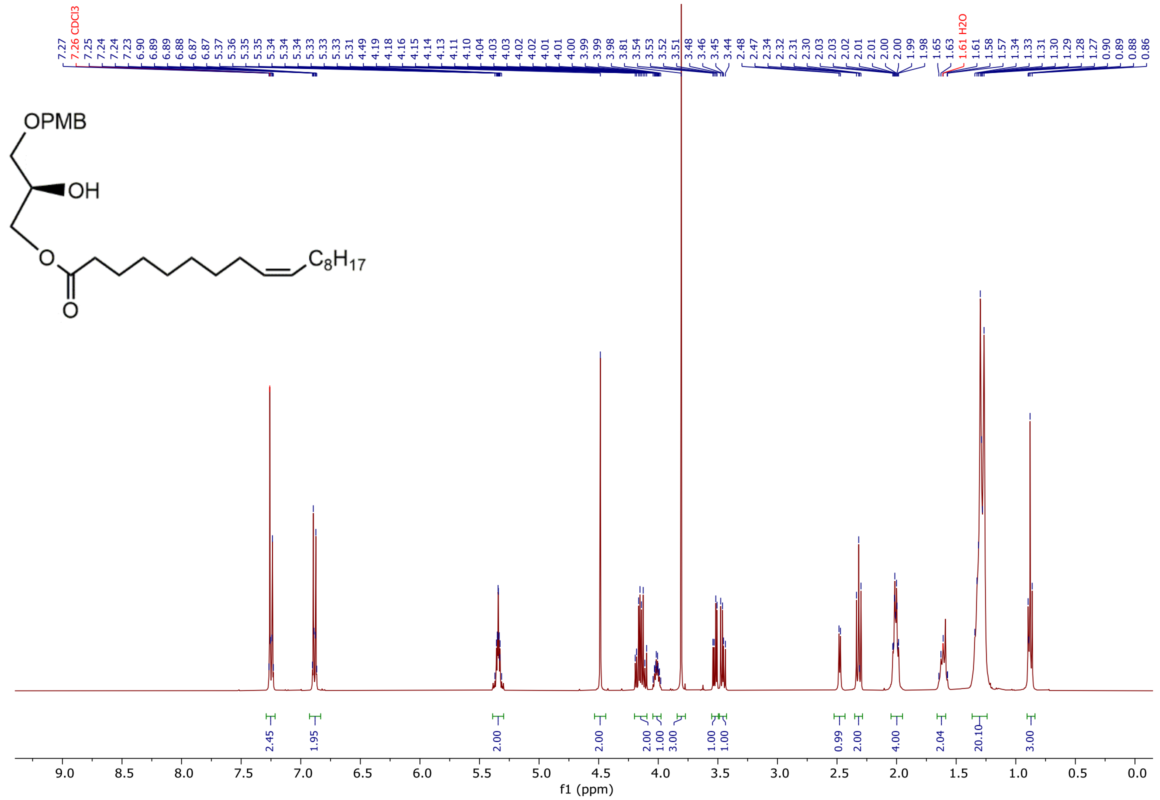

$^{13}C\{H\}$ NMR (101 MHz, $CDCl_3$) of compound (*R*)-**14**

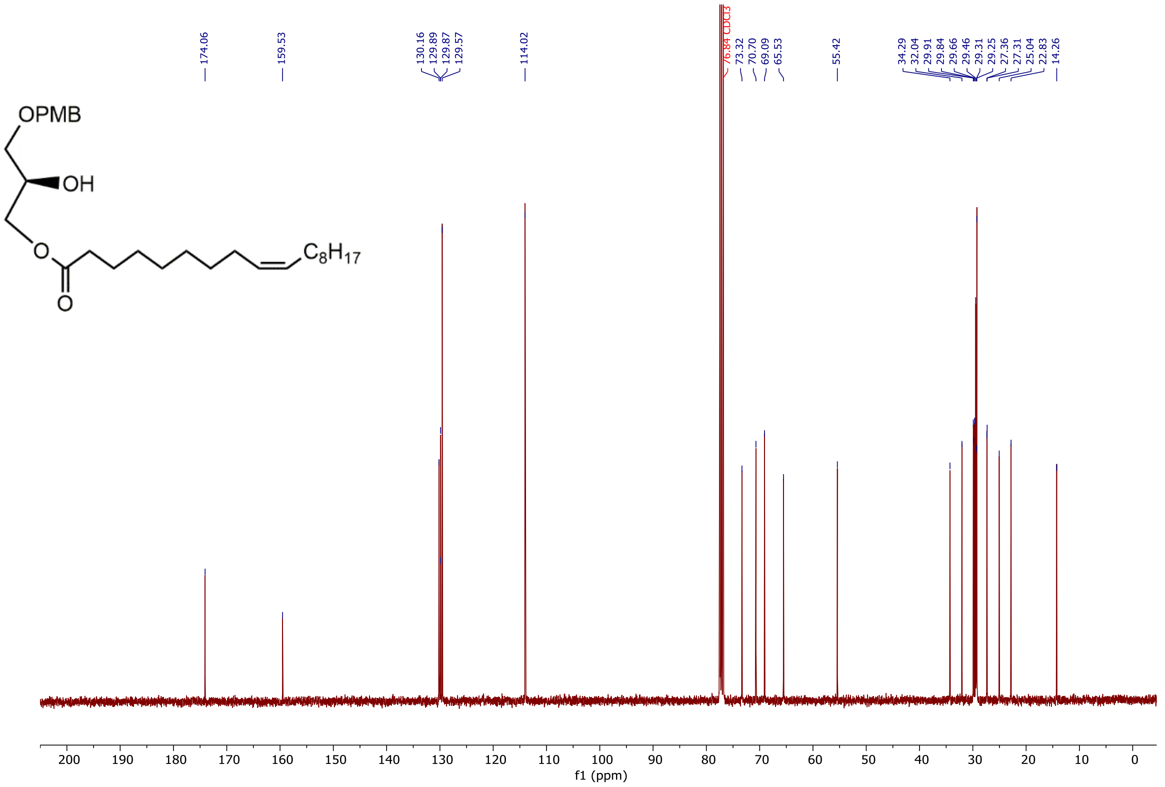

^{1}H-^{1}H COSY spectrum of compound (*R*)-**14**

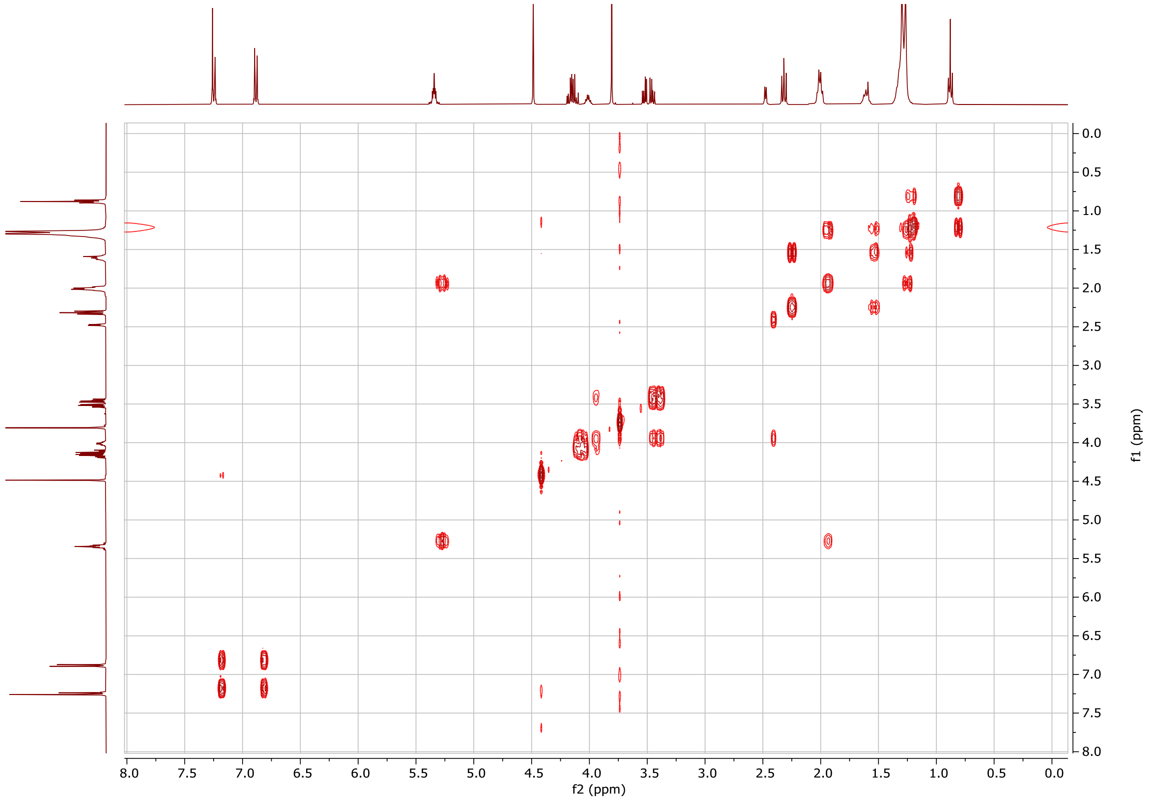

^{13}C-^{1}H HSQC spectrum of compound (*R*)-**14**

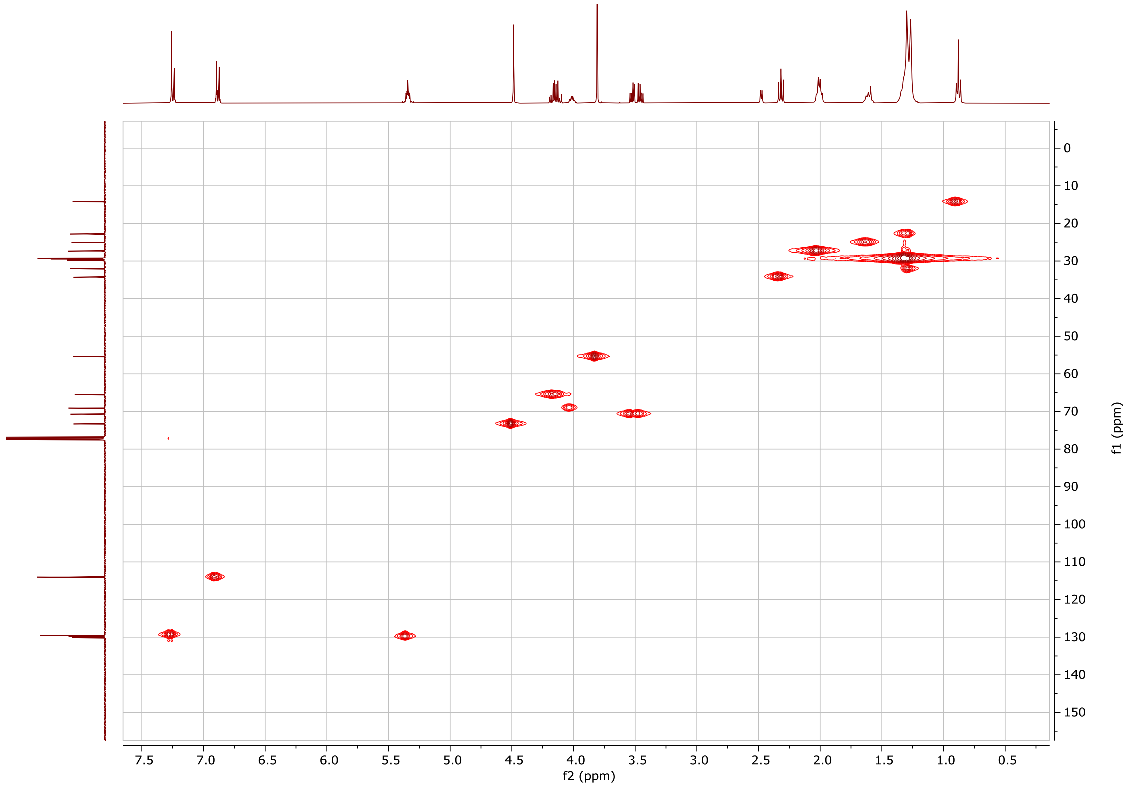

^{1}H NMR (400 MHz, $CDCl_3$) of compound (*R*)-**15a**

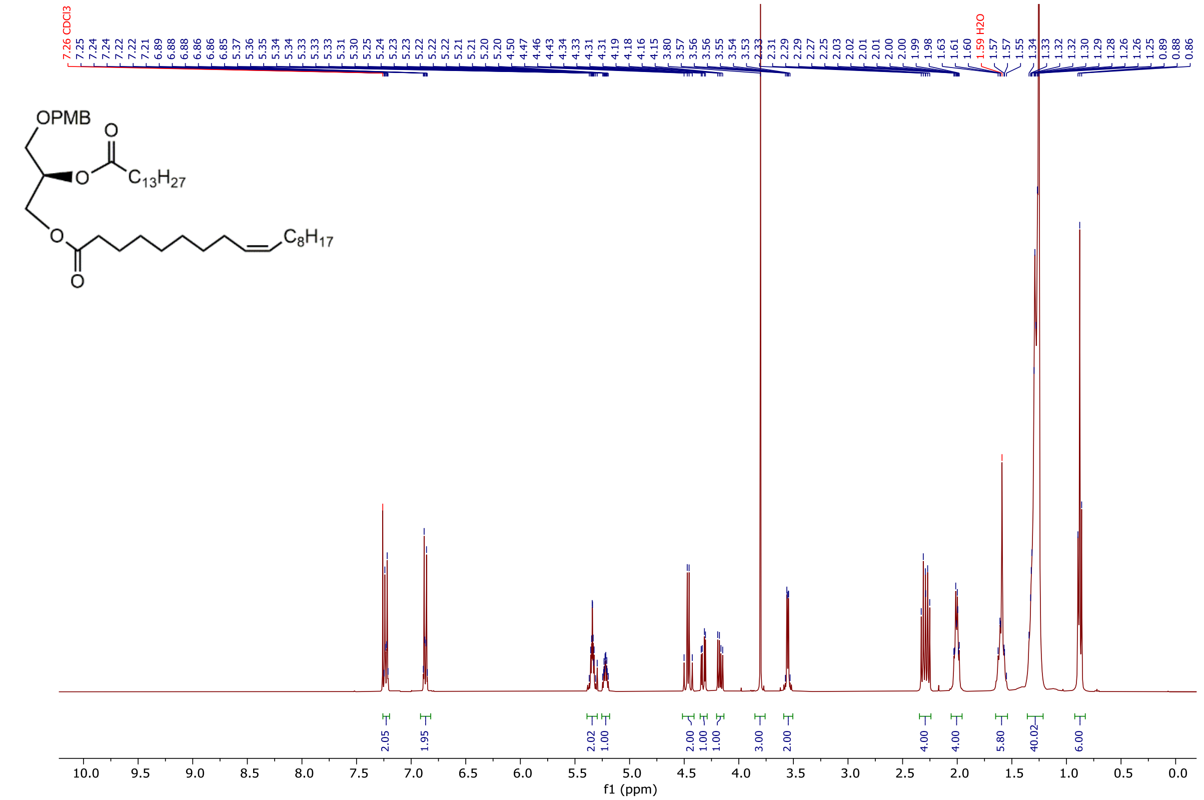

$^{13}C\{H\}$ NMR (101 MHz, $CDCl_3$) of compound (*R*)-**15a**

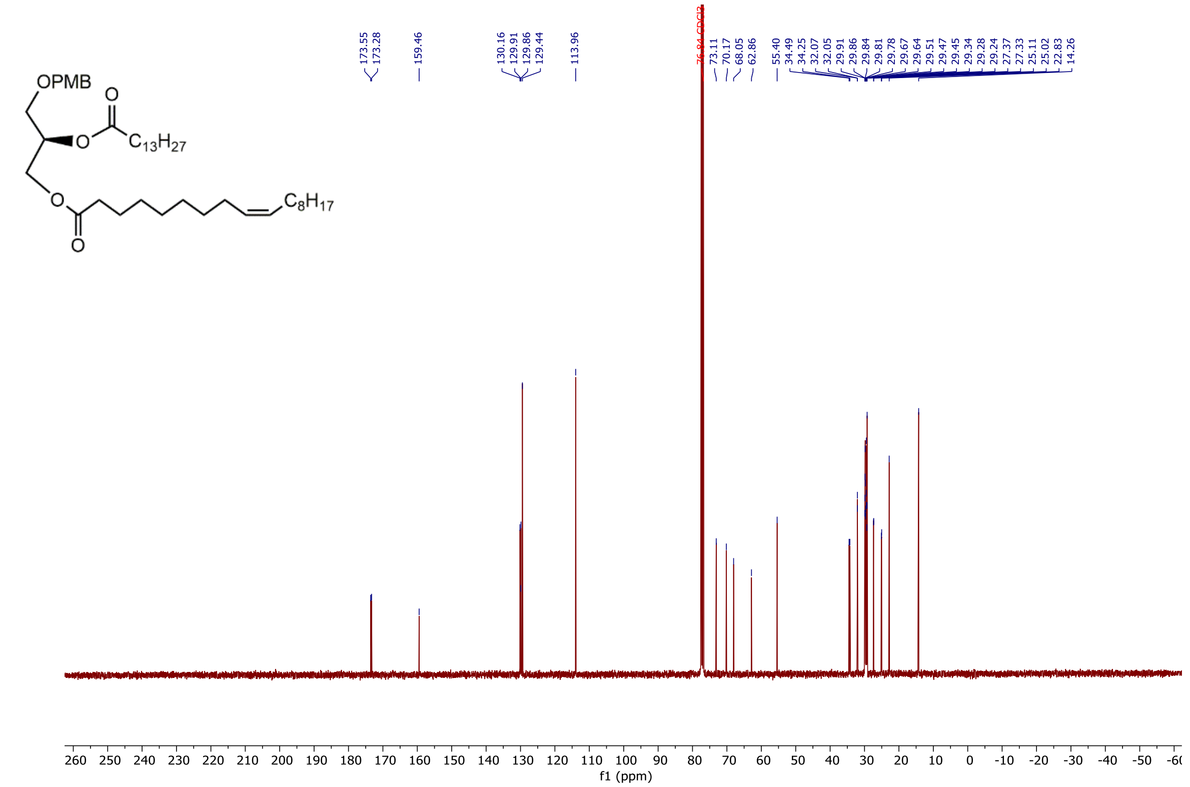

^{1}H-^{1}H COSY spectrum of compound (*R*)-**15a**

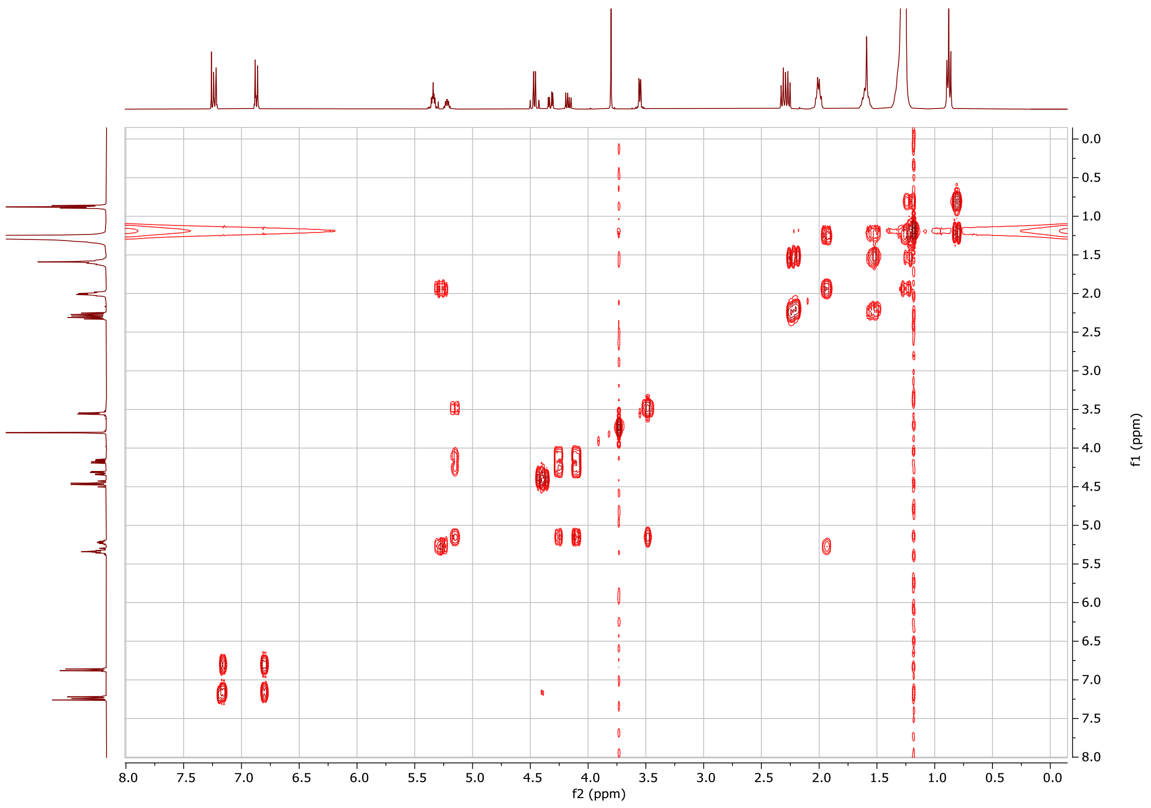

^{13}C-^{1}H HSQC spectrum of compound (*R*)-**15a**

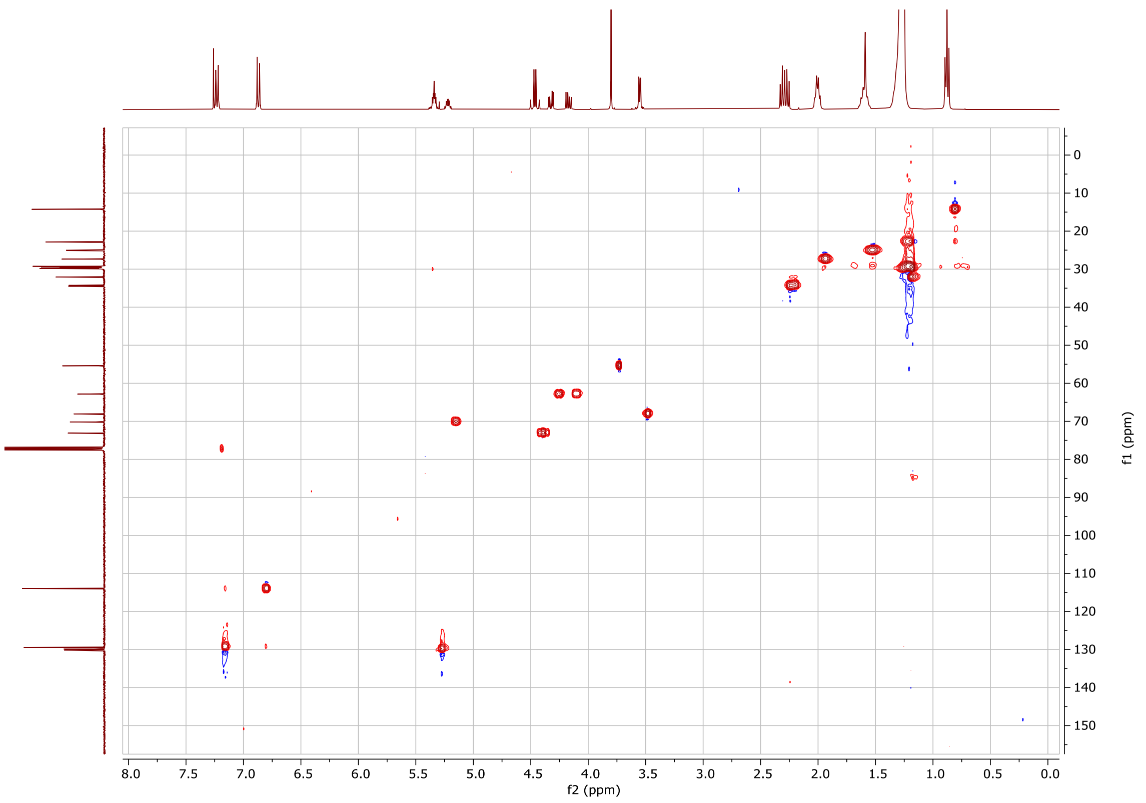

^{1}H NMR (400 MHz, $CDCl_3$) of compound (*R*)-**15b**

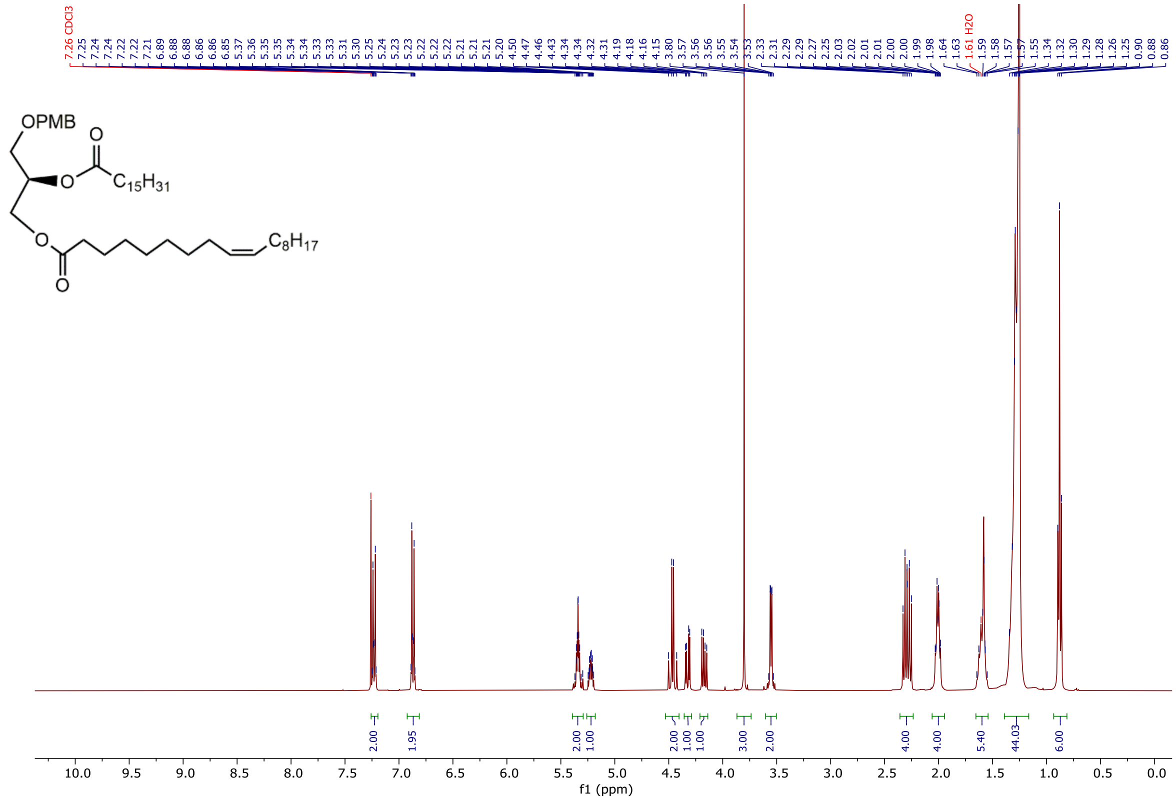

^{13}C{H} NMR (101 MHz, $CDCl_3$) of compound (*R*)-**15b**

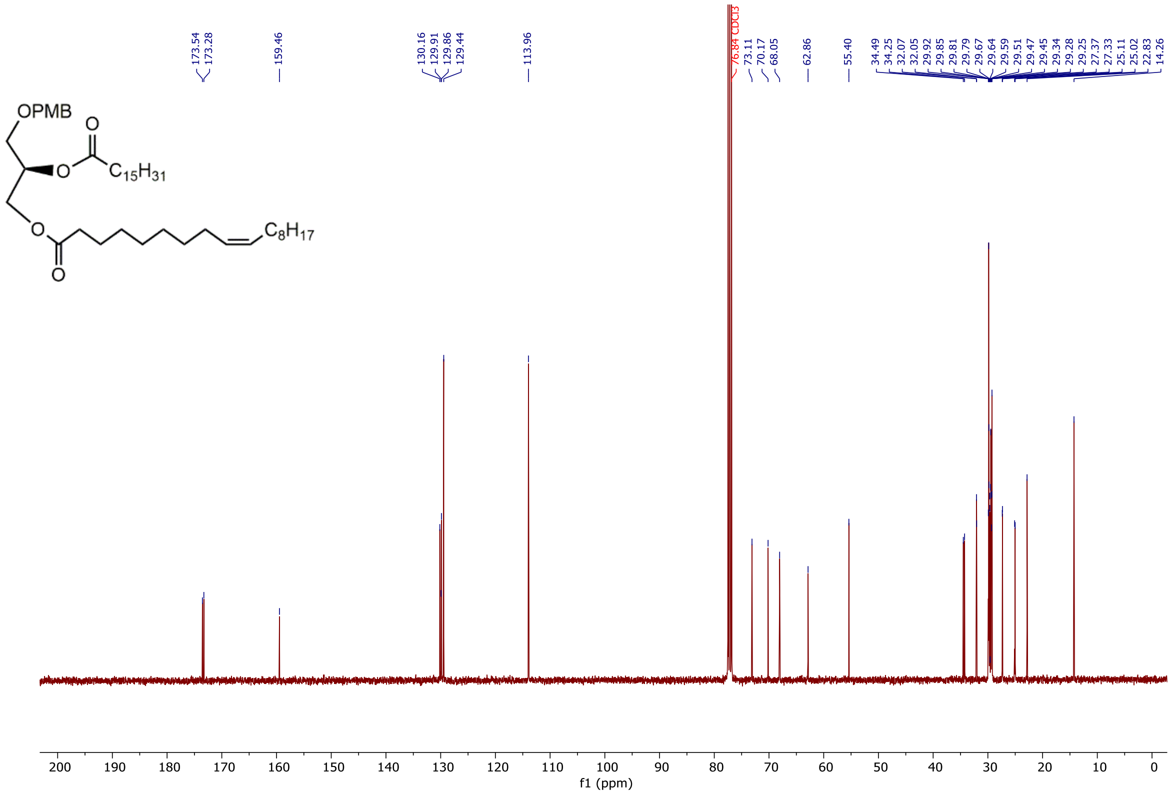

^{1}H-^{1}H COSY spectrum of compound (*R*)-**15b**

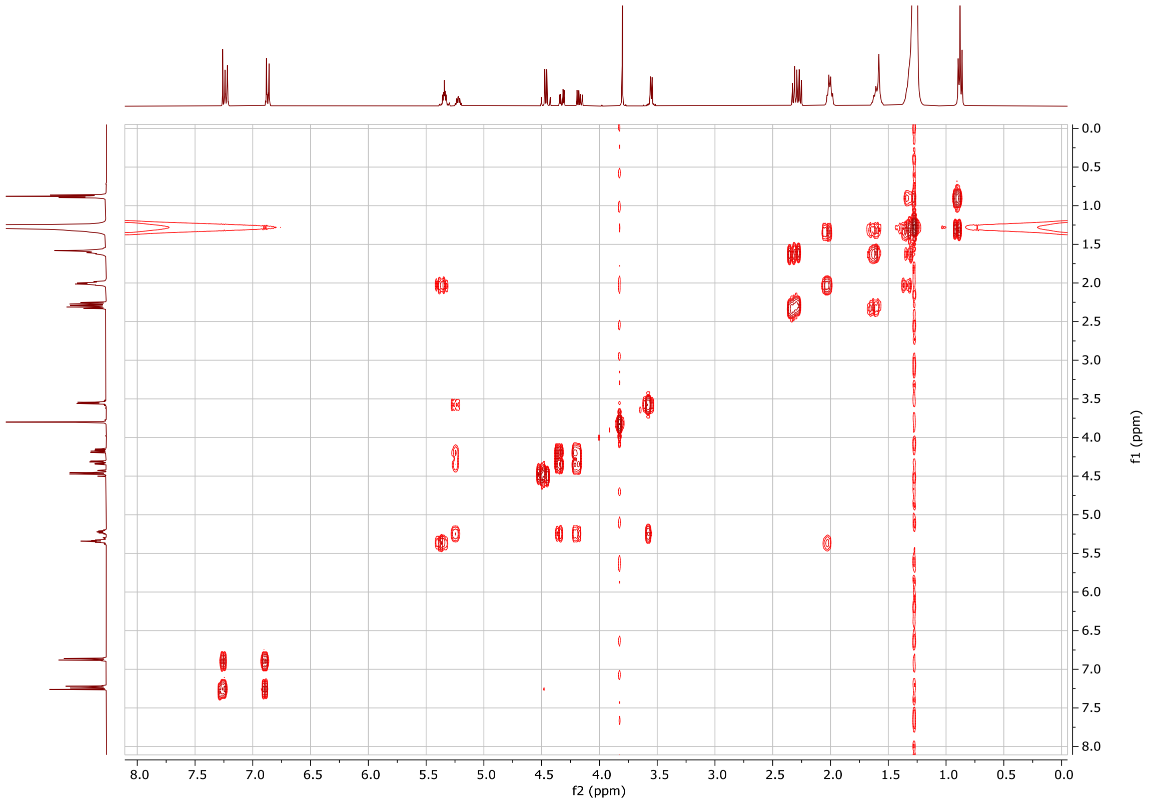

^{13}C-^{1}H HSQC spectrum of compound (*R*)-**15b**

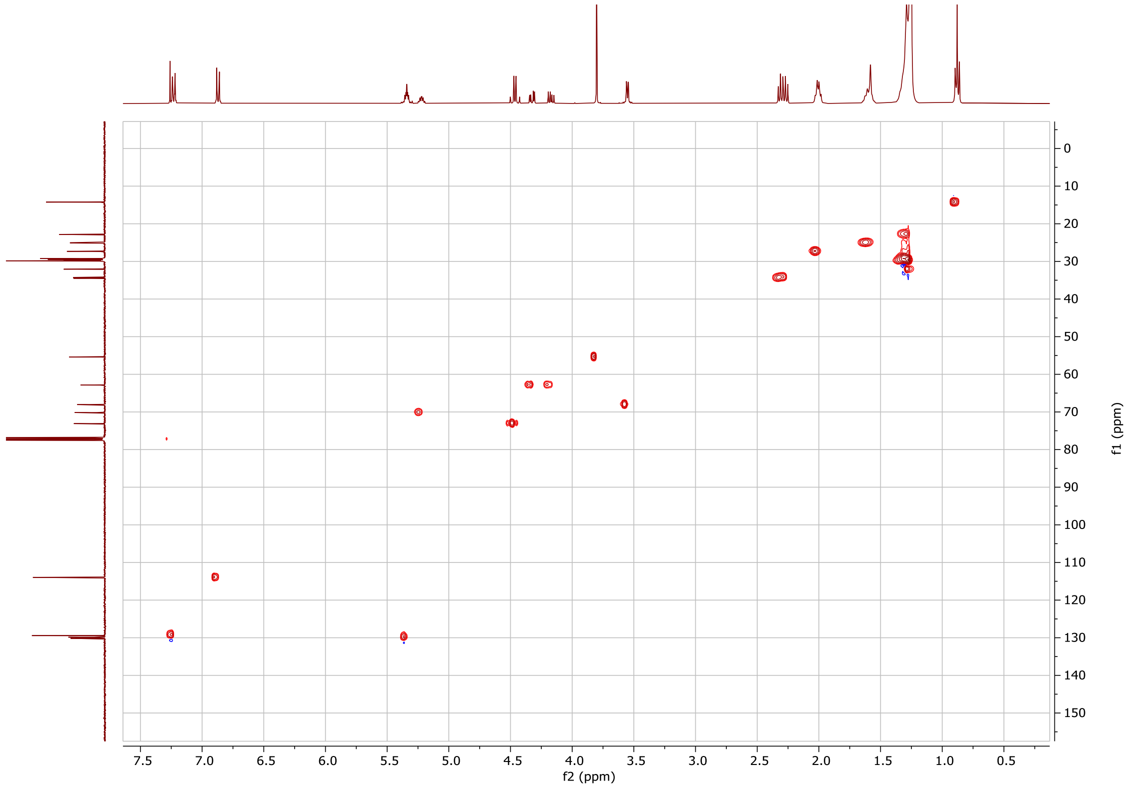

^{1}H NMR (400 MHz, $CDCl_3$) of compound (*R*)-**16a**

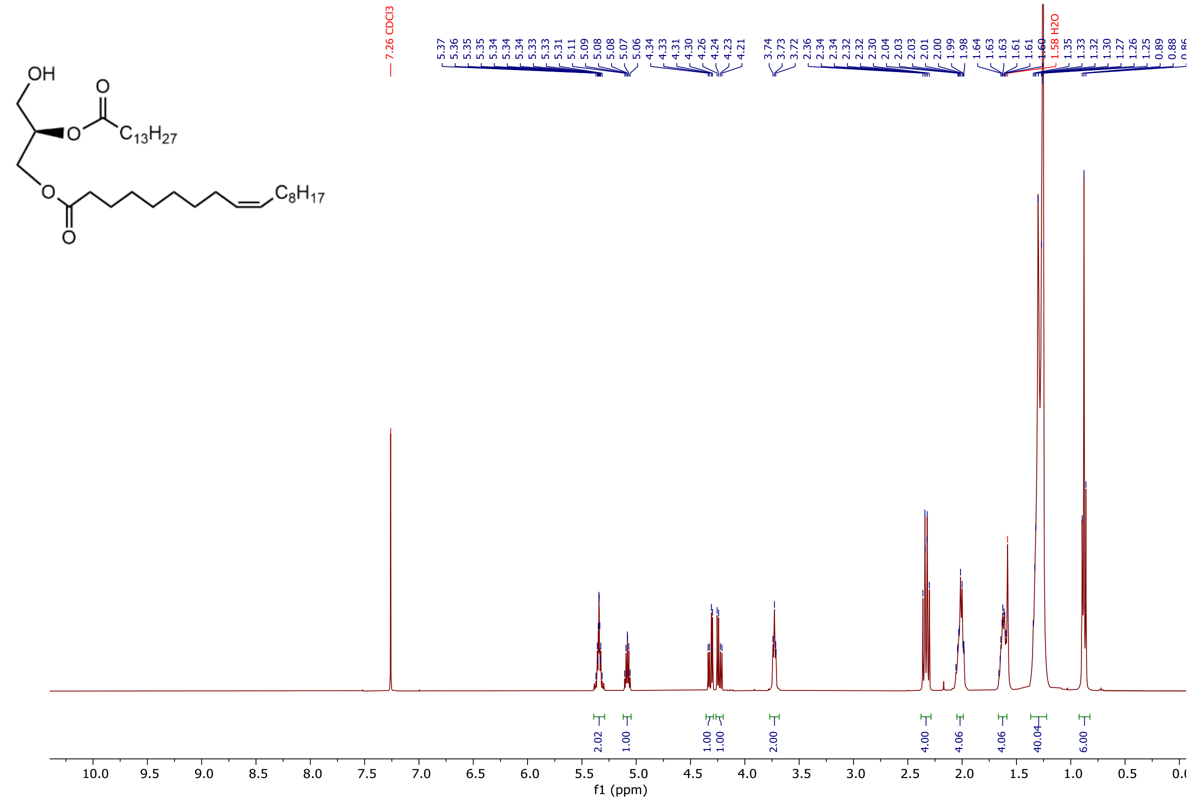

^{13}C{H} NMR (101 MHz, $CDCl_3$) of compound (*R*)-**16a**

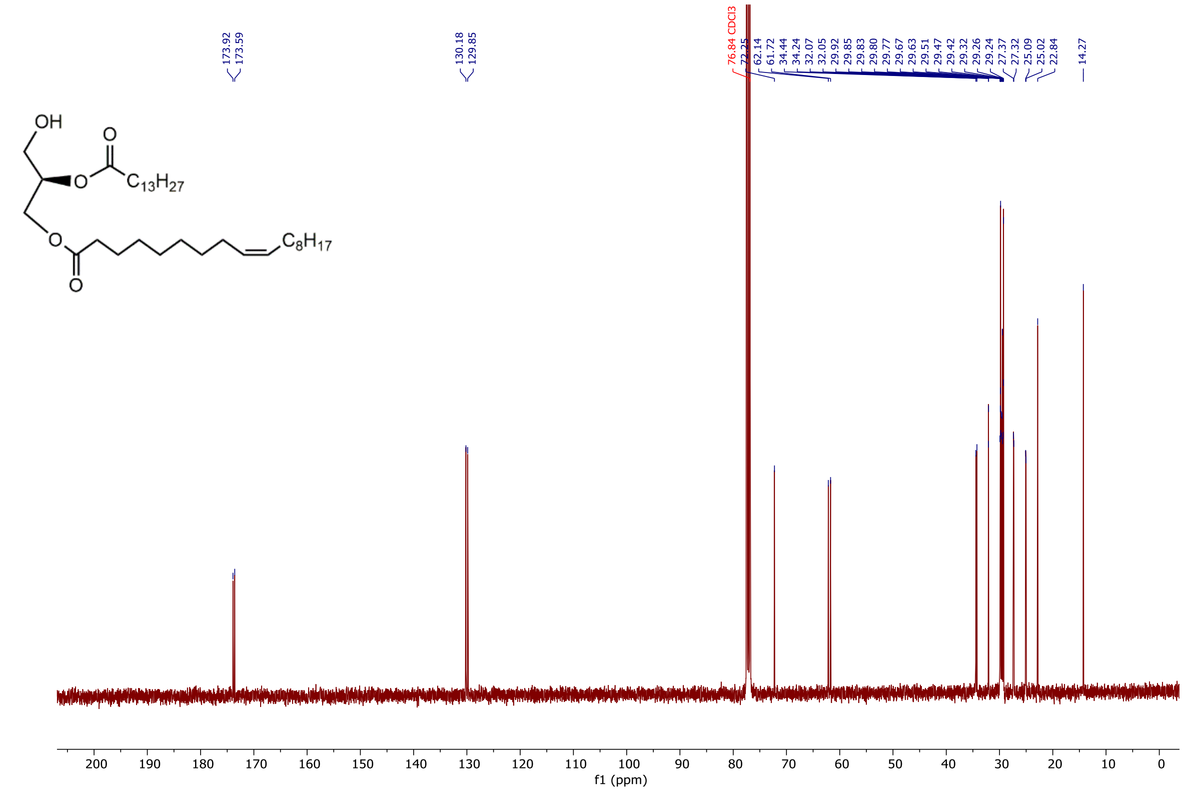

¹H-¹H COSY spectrum of compound (*R*)-**16a**

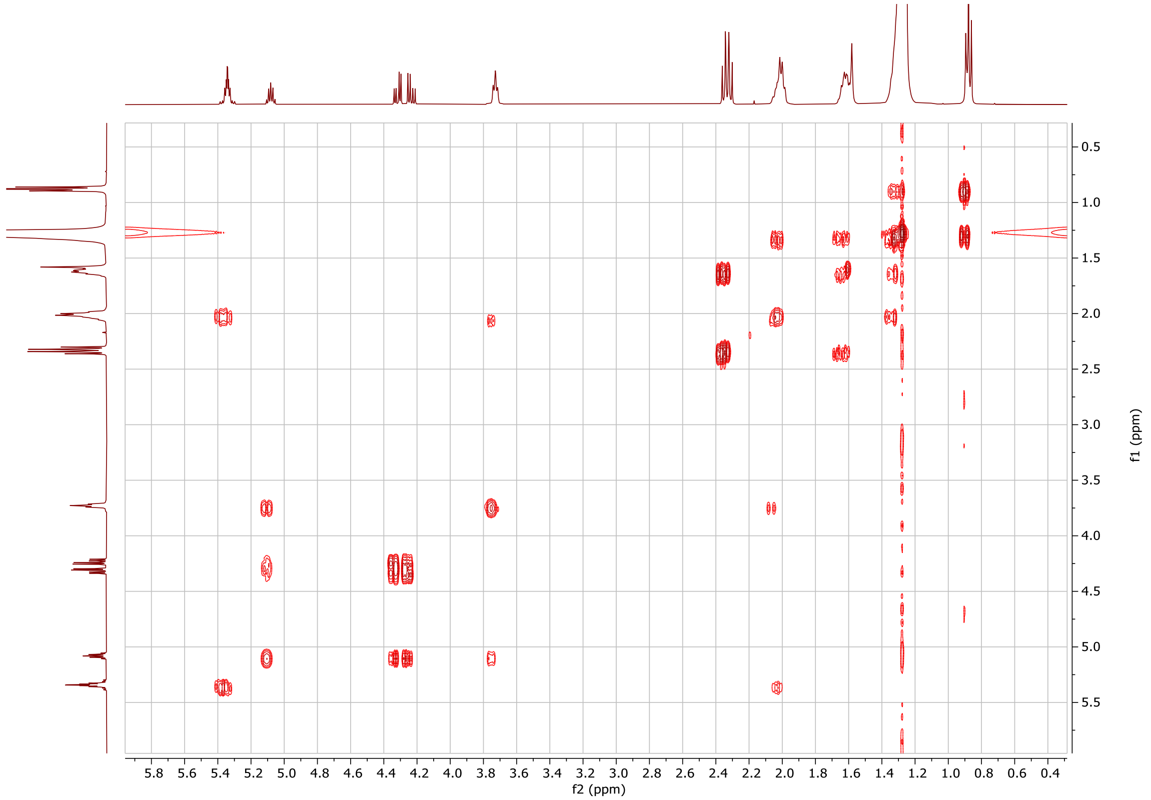

¹³C-¹H HSQC spectrum of compound (*R*)-**16a**

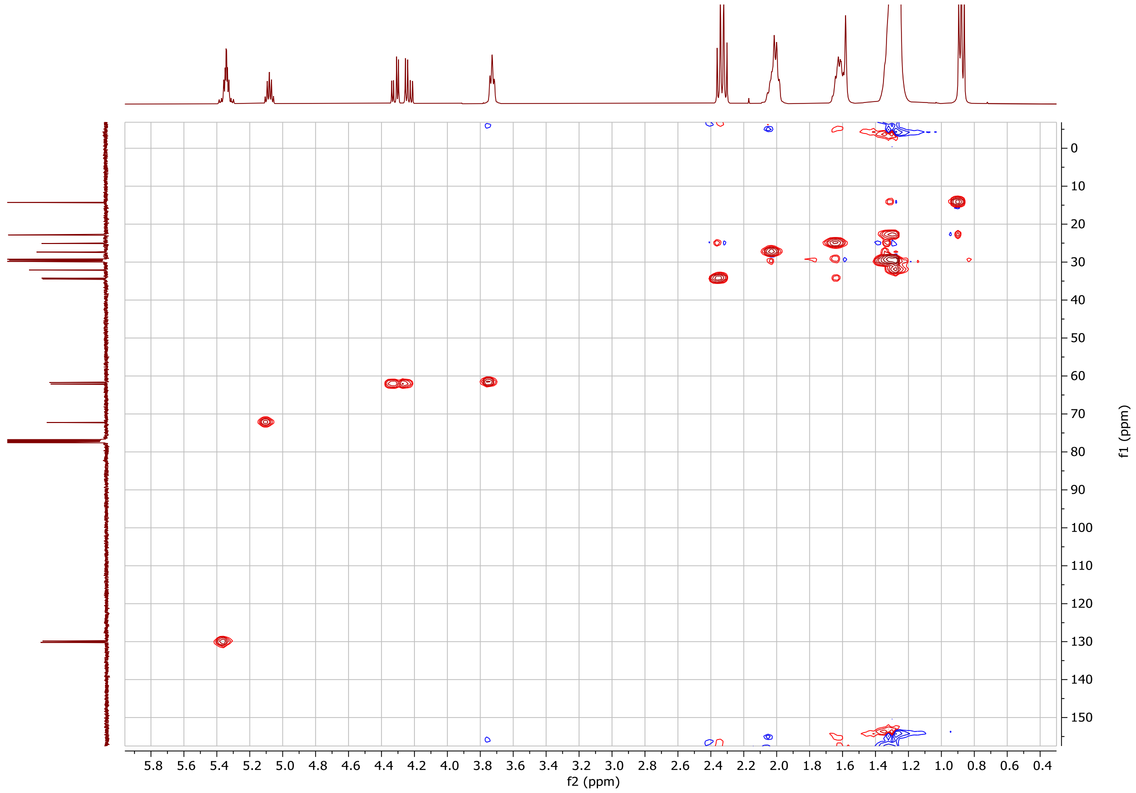

^{1}H NMR (400 MHz, $CDCl_3$) of compound (*R*)-**16b**

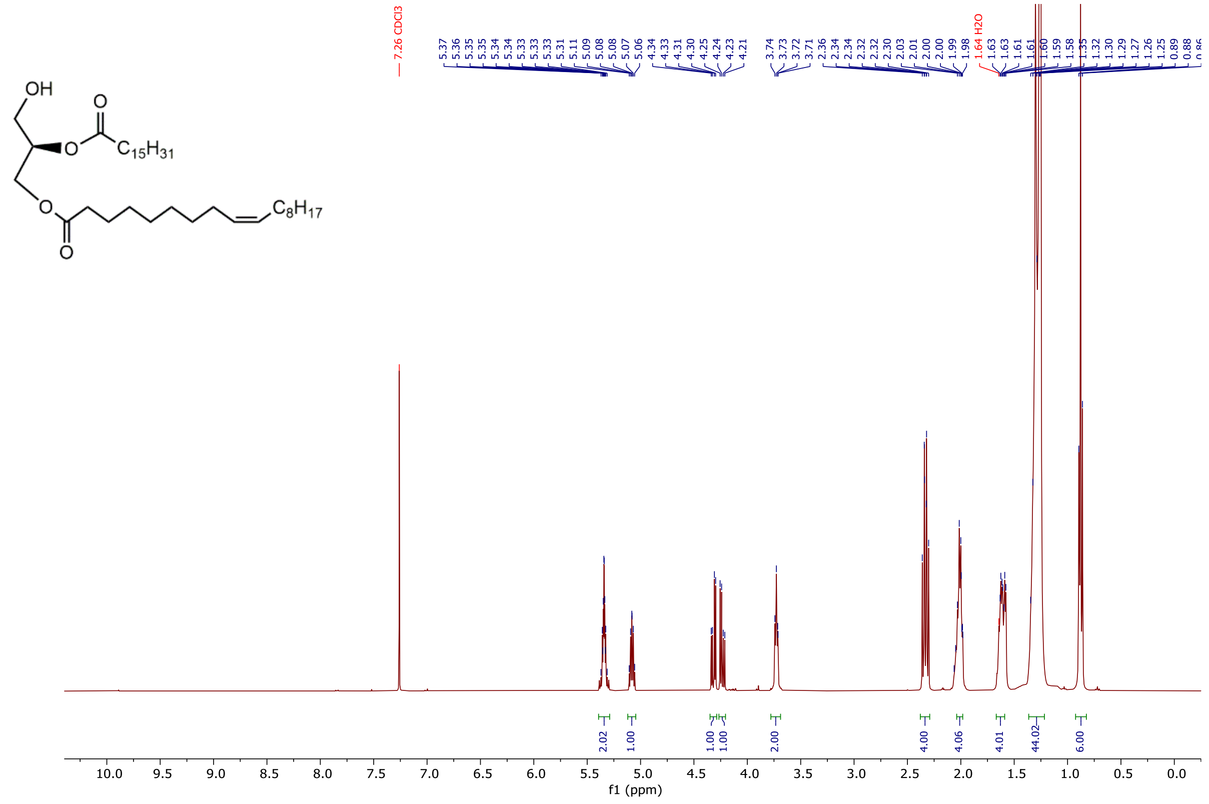

$^{13}C\{H\}$ NMR (101 MHz, $CDCl_3$) of compound (*R*)-**16b**

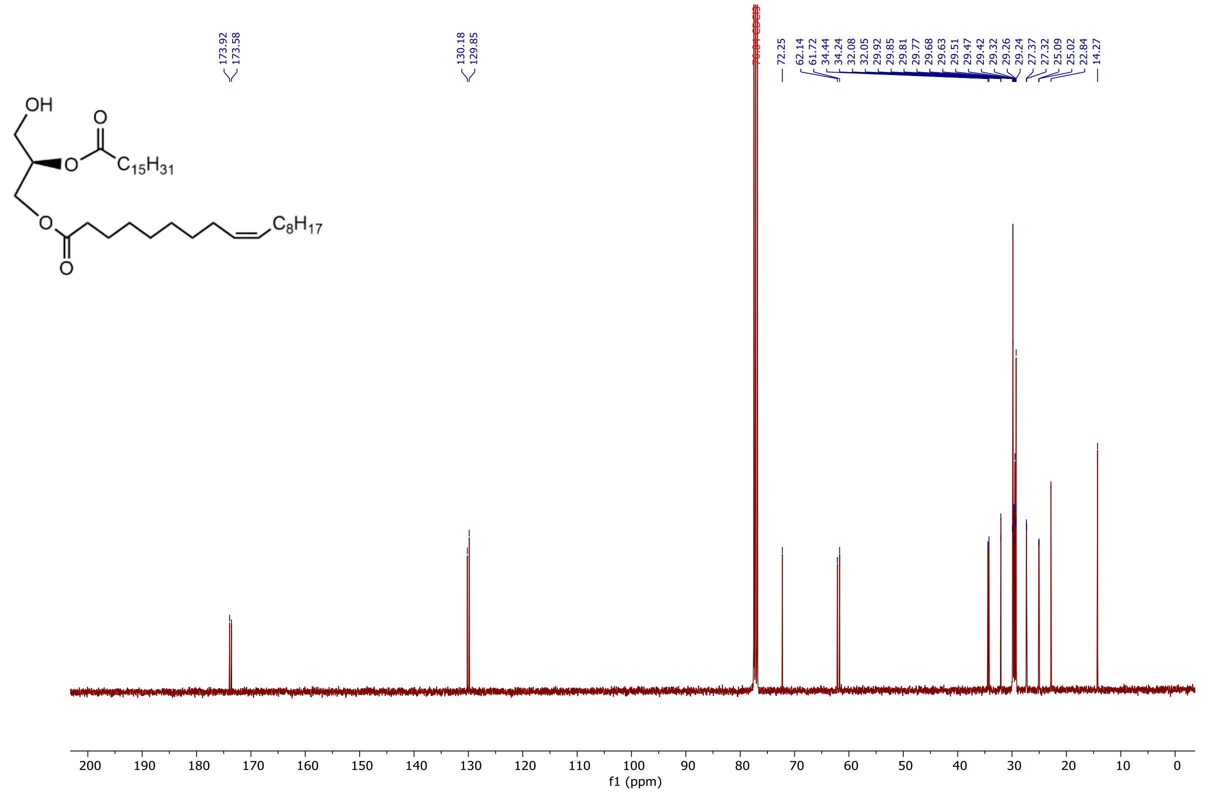

^{1}H-^{1}H COSY spectrum of compound (*R*)-**16b**

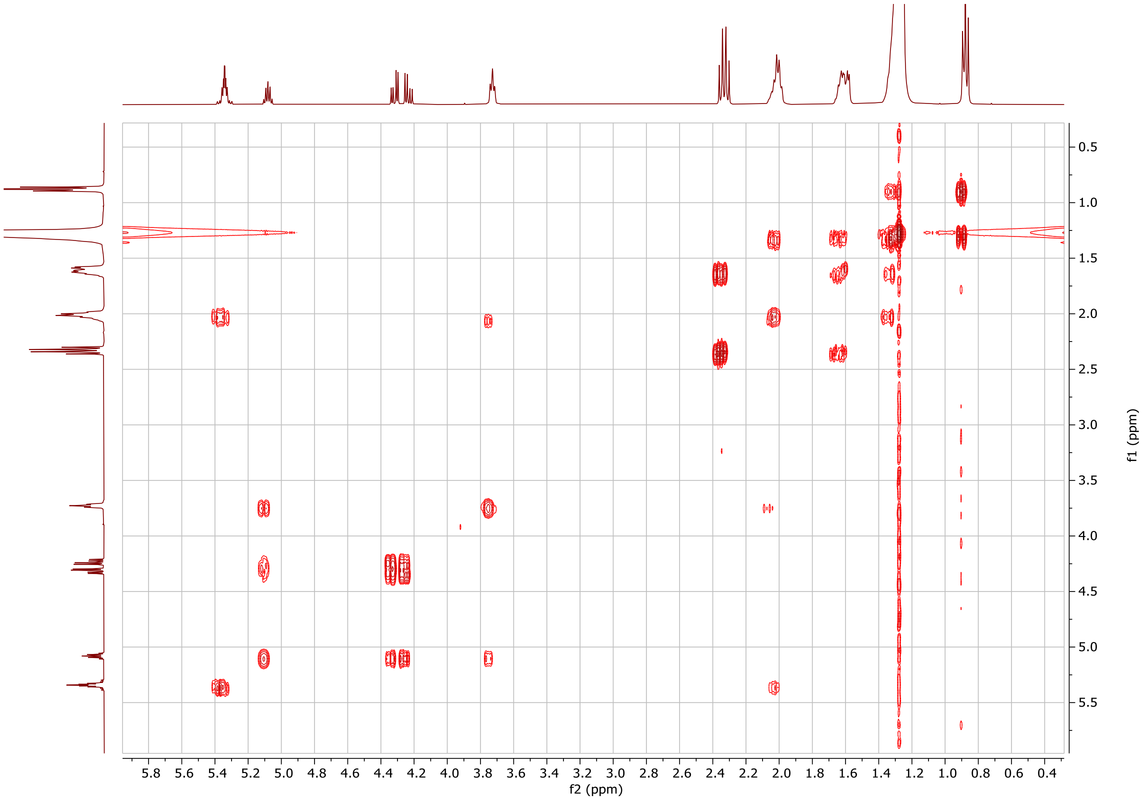

^{13}C-^{1}H HSQC spectrum of compound (*R*)-**16b**

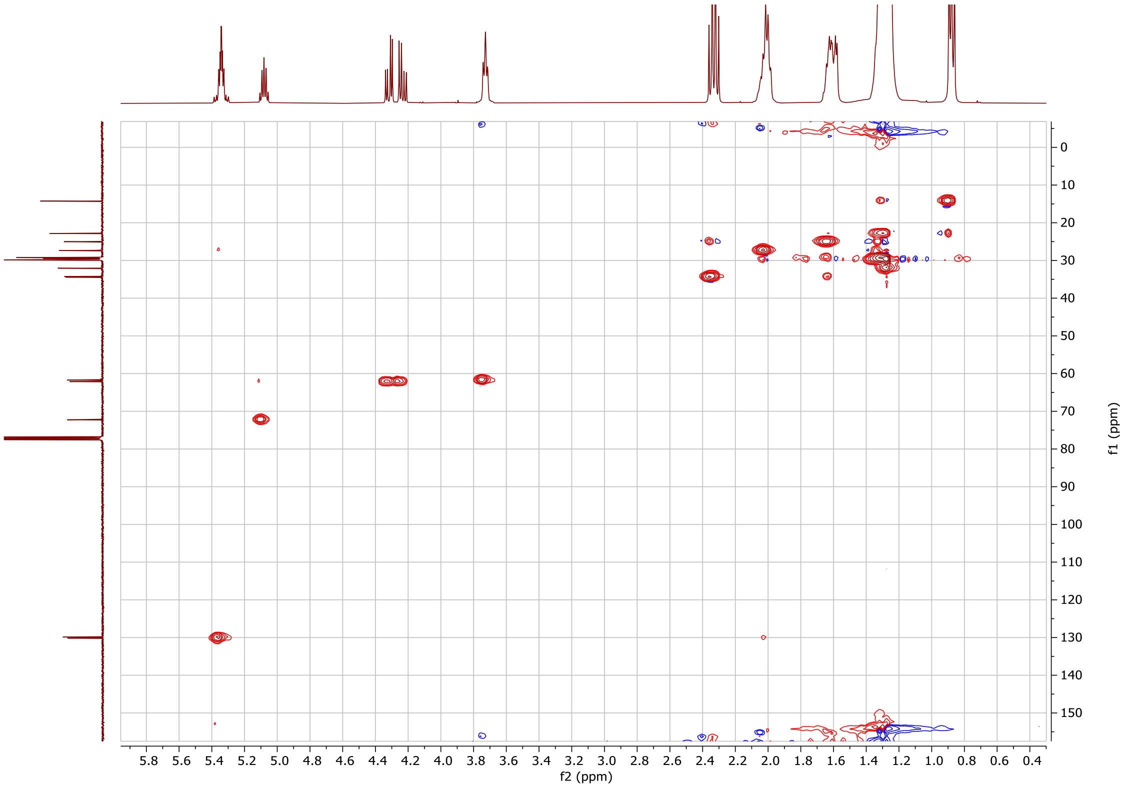

^{1}H NMR (400 MHz, CDCl3) of compound (*S*)-**5**

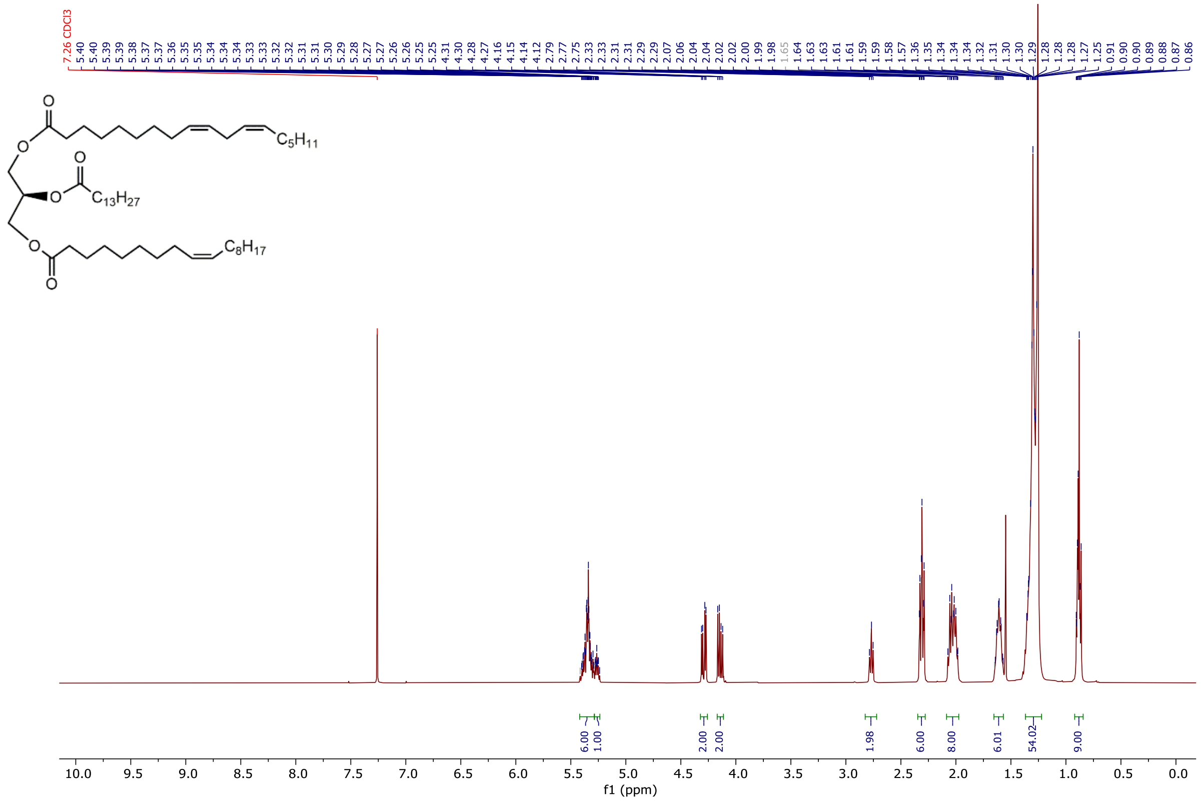

^{13}C{H} NMR (101 MHz, CDCl3) of compound (*S*)-**5**

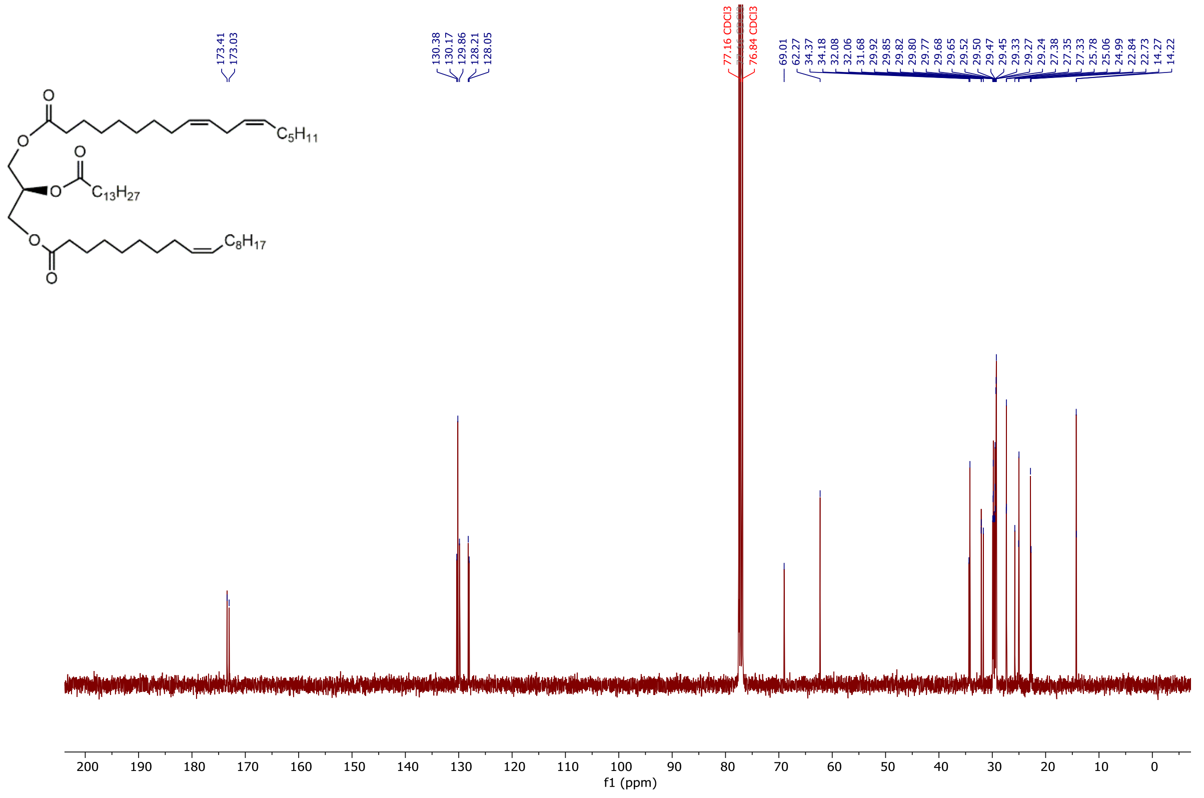

^{1}H-^{1}H COSY spectrum of compound (*S*)-**5**

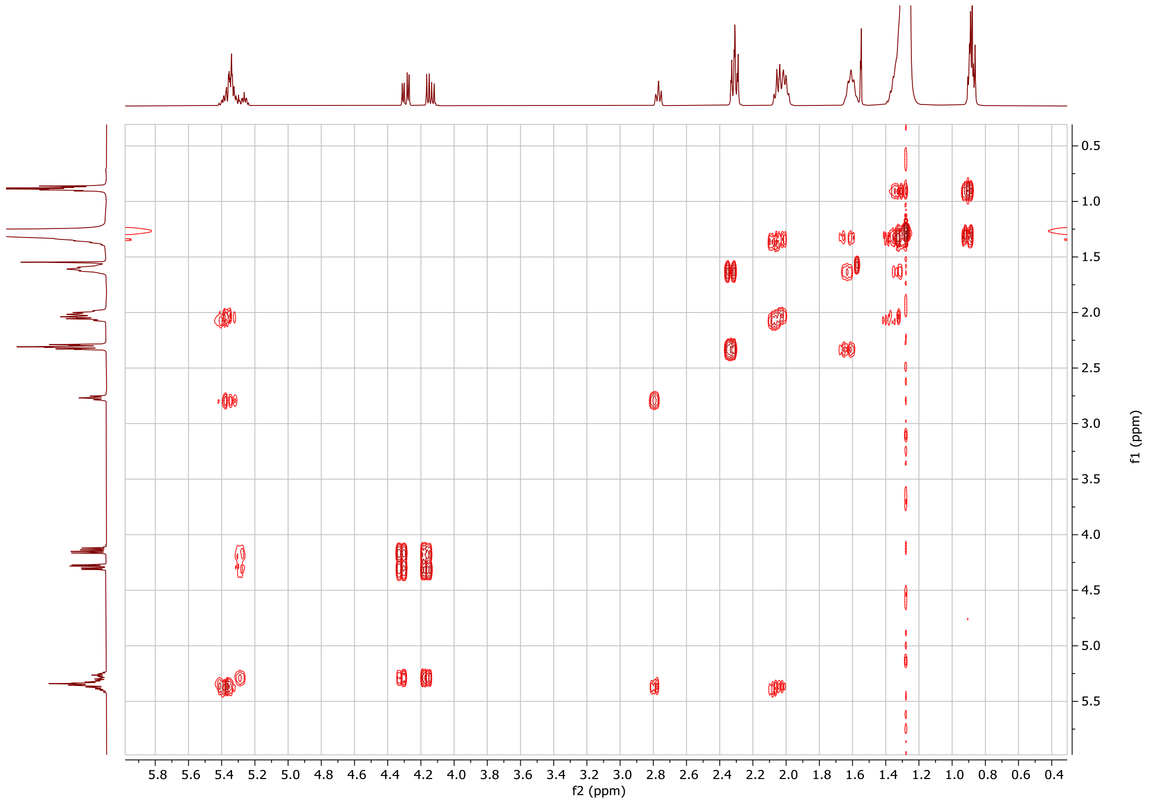

^{13}C-^{1}H HSQC spectrum of compound (*S*)-**5**

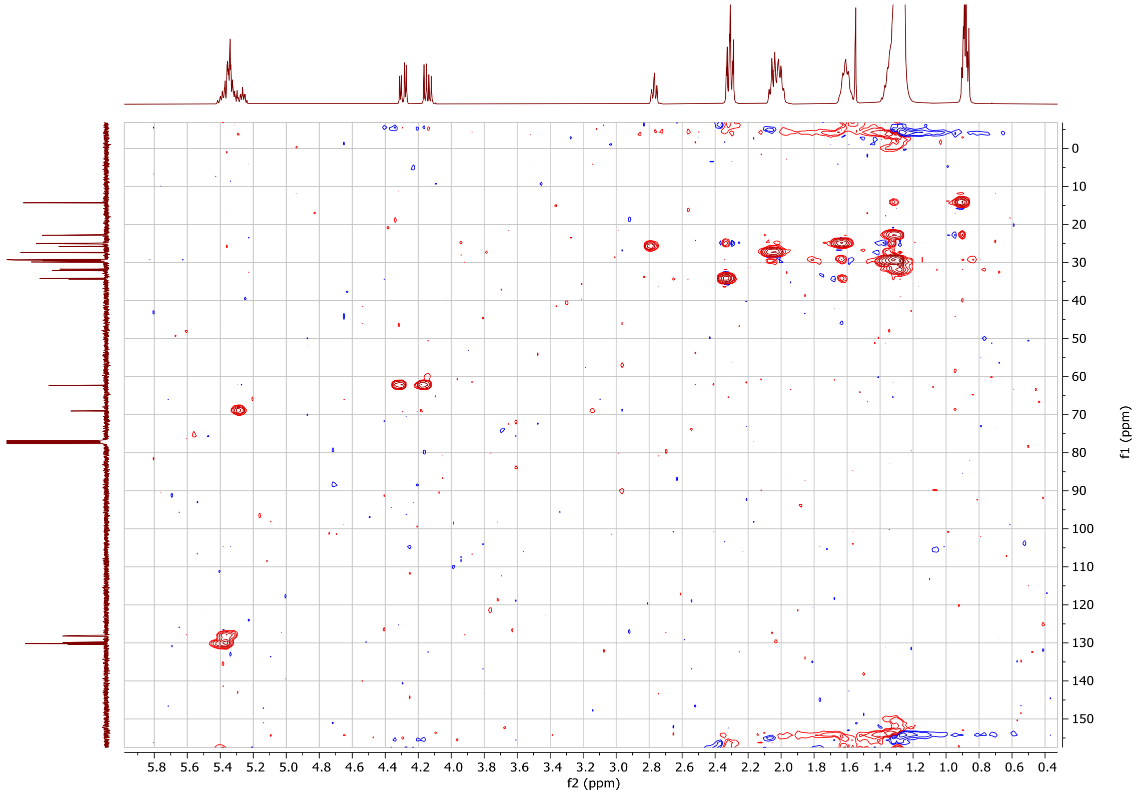

^{1}H NMR (400 MHz, $CDCl_3$) of compound (*S*)-**6**

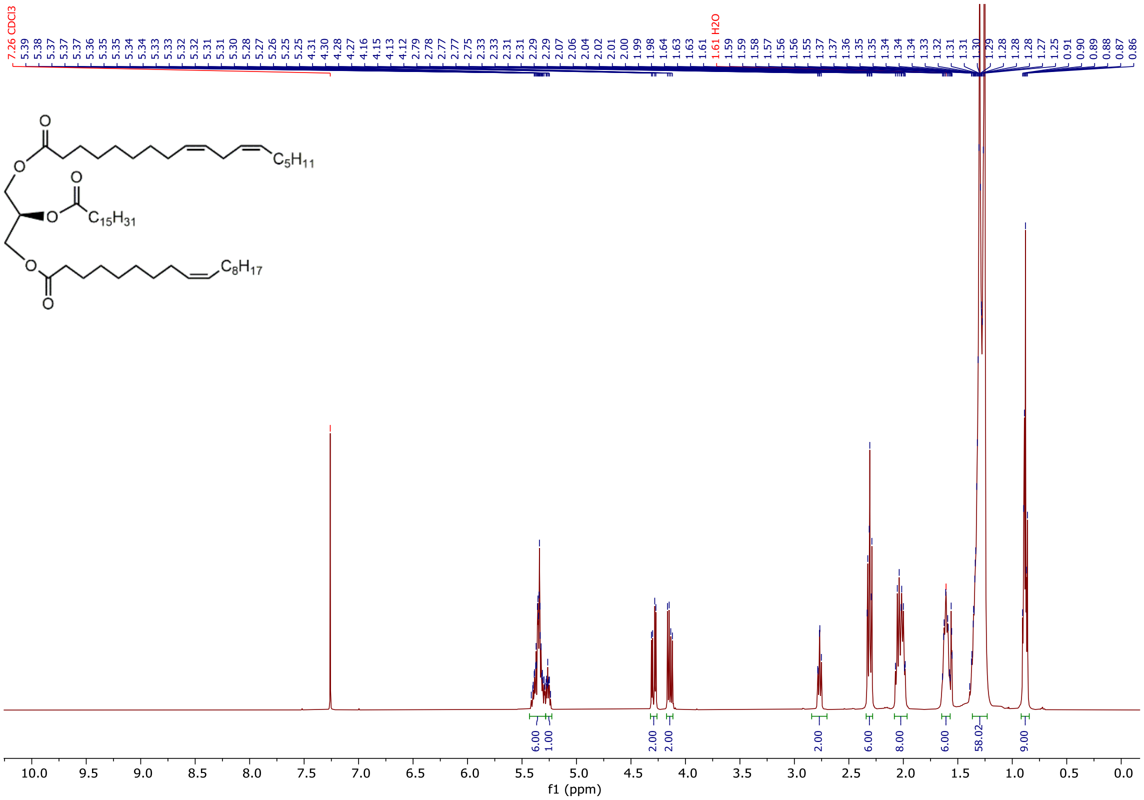

$^{13}C\{H\}$ NMR (101 MHz, $CDCl_3$) of compound (*S*)-**6**

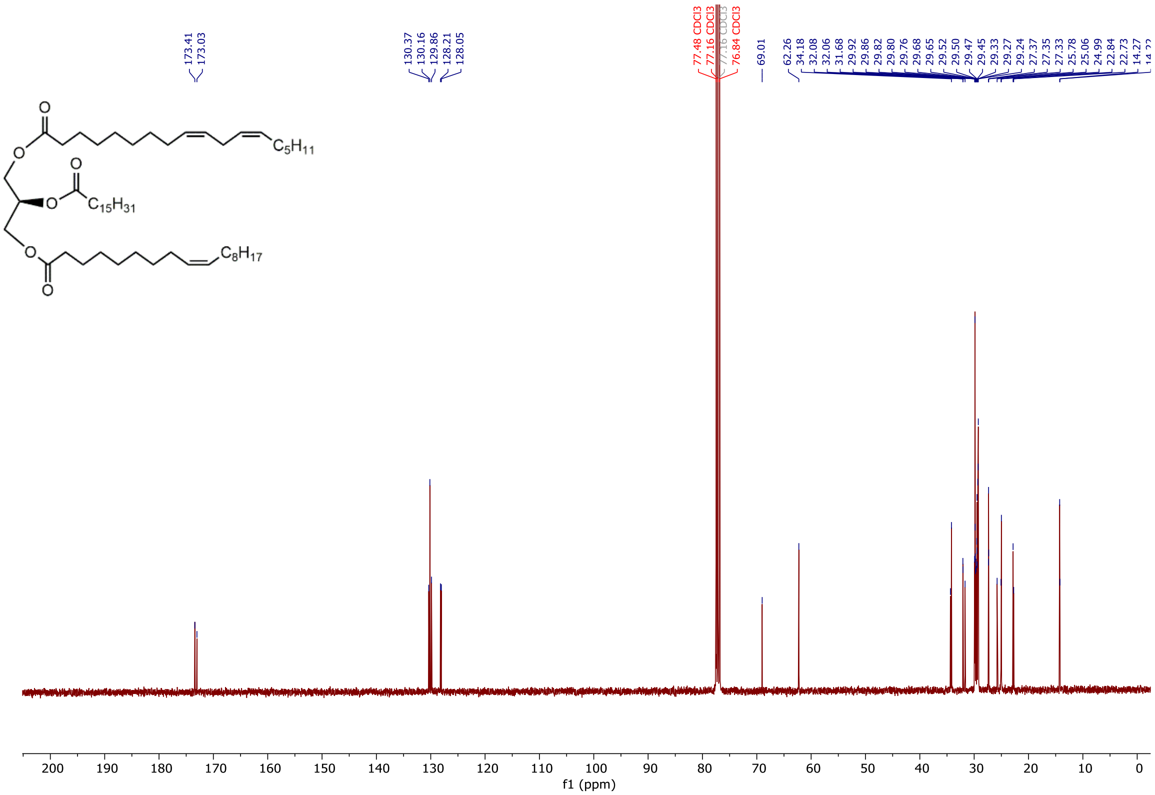

^{1}H-^{1}H COSY spectrum of compound (*S*)-**6**

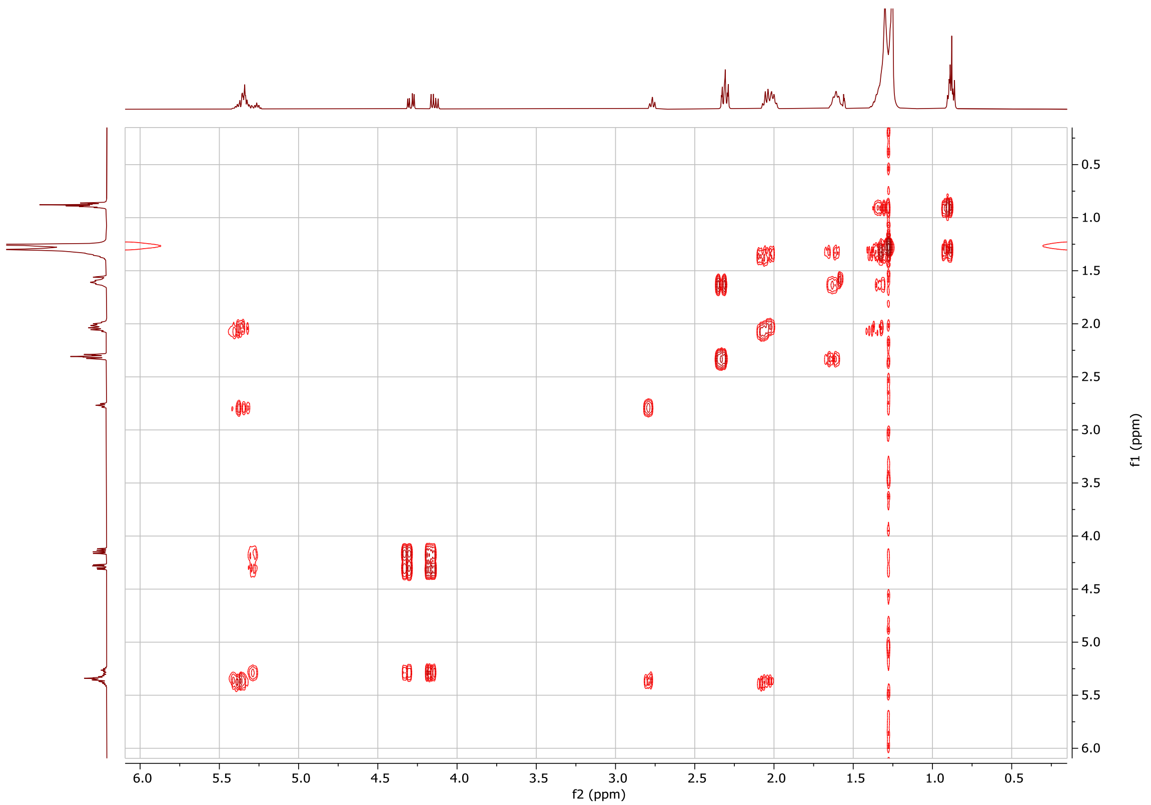

^{13}C-^{1}H HSQC spectrum of compound (*S*)-**6**

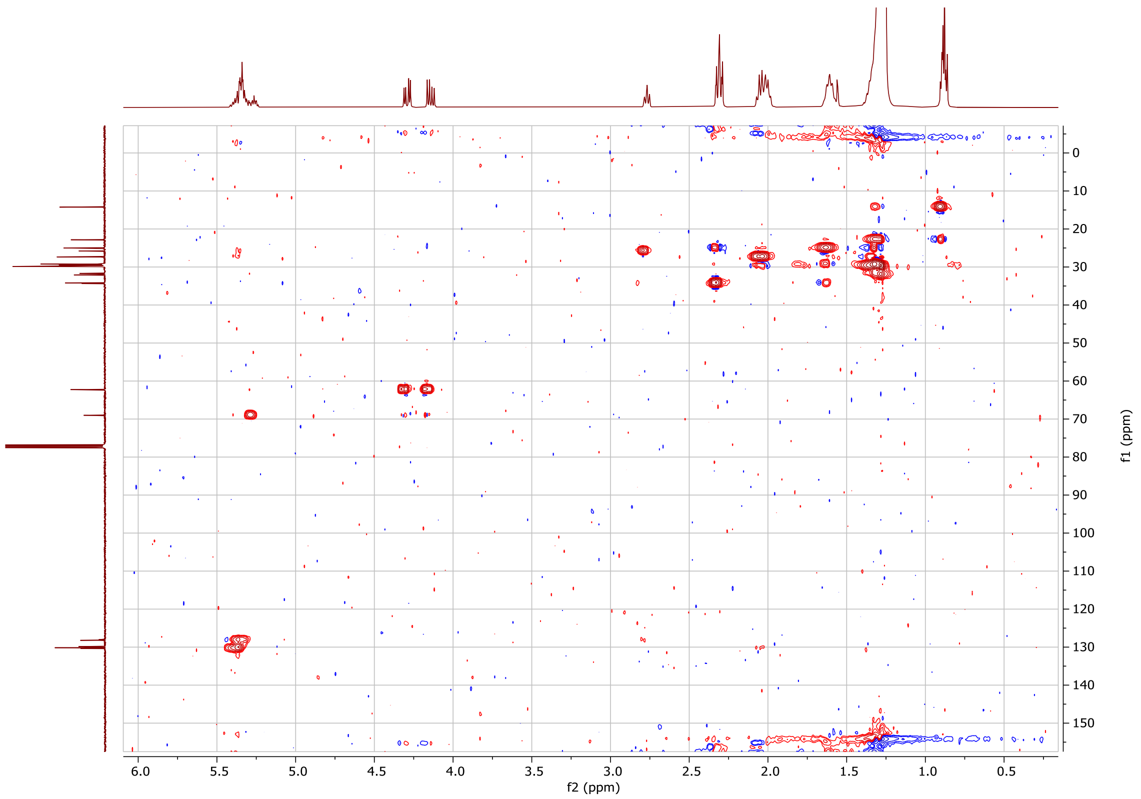

^{1}H NMR (400 MHz, $CDCl_3$) of compound (*R*)-**17**

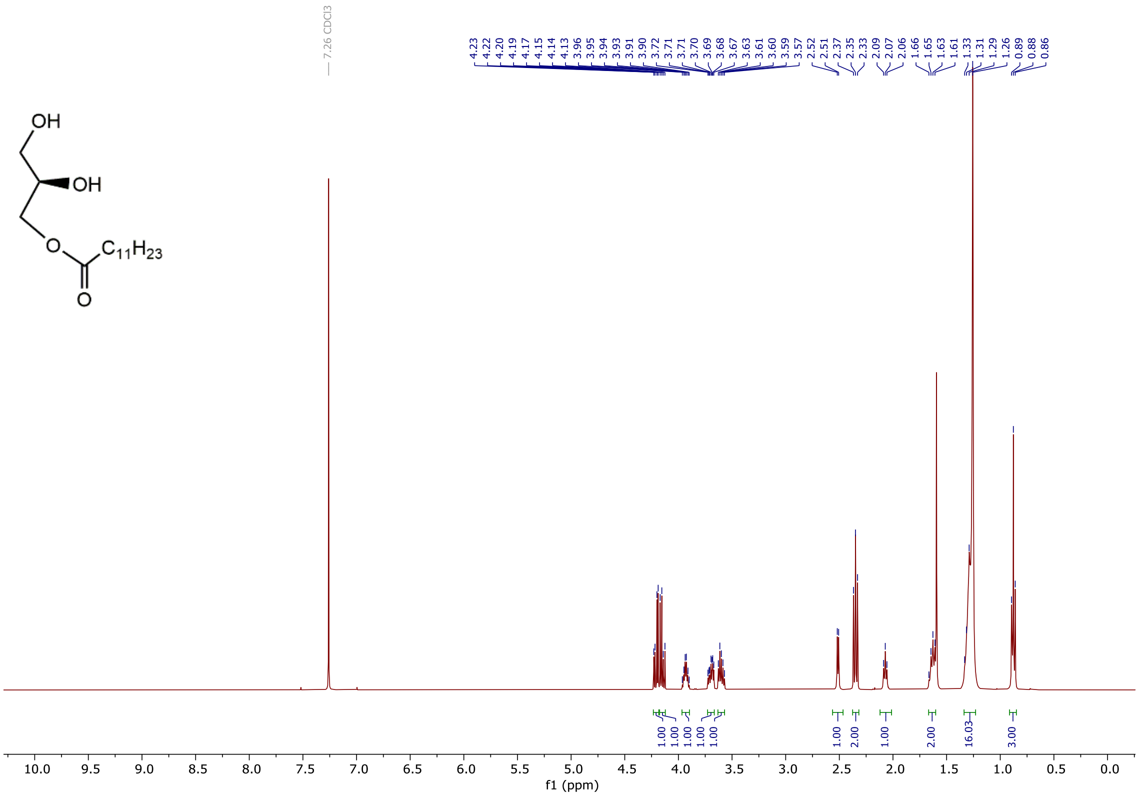

$^{13}C\{H\}$ NMR (101 MHz, $CDCl_3$) of compound (*R*)-**17**

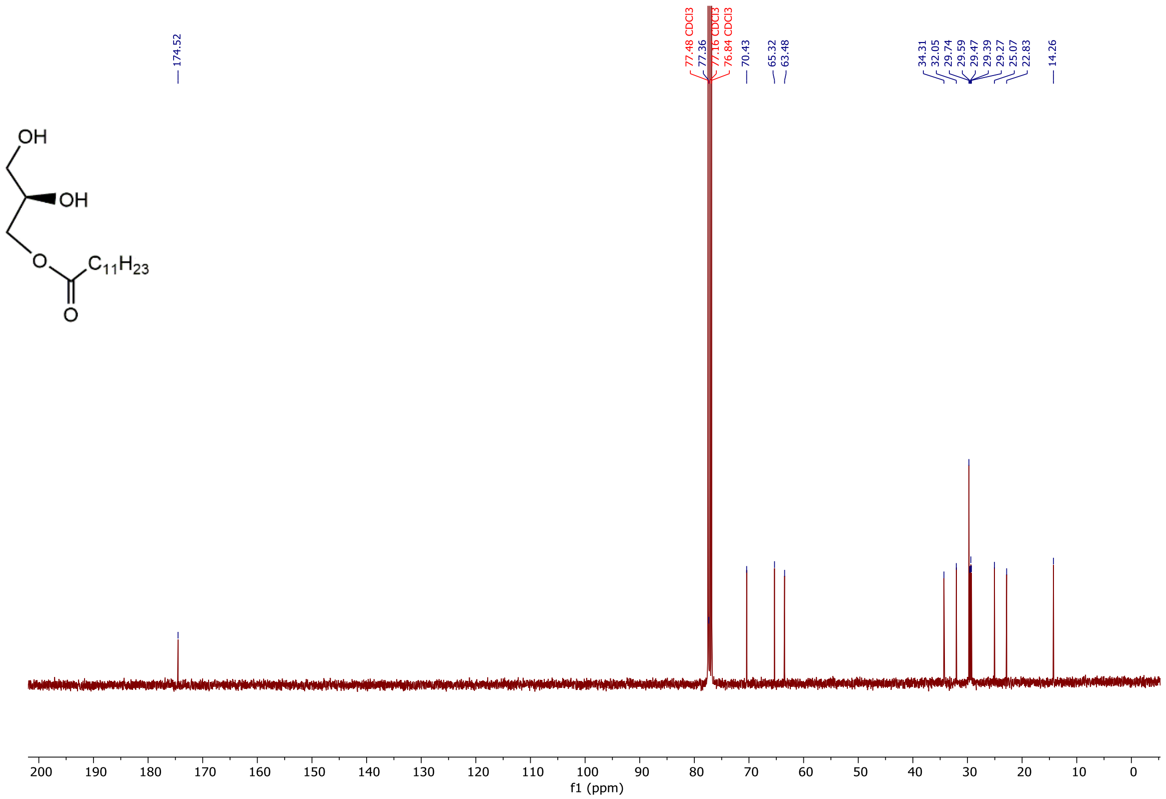

^{1}H-^{1}H COSY spectrum of compound (*R*)-**17**

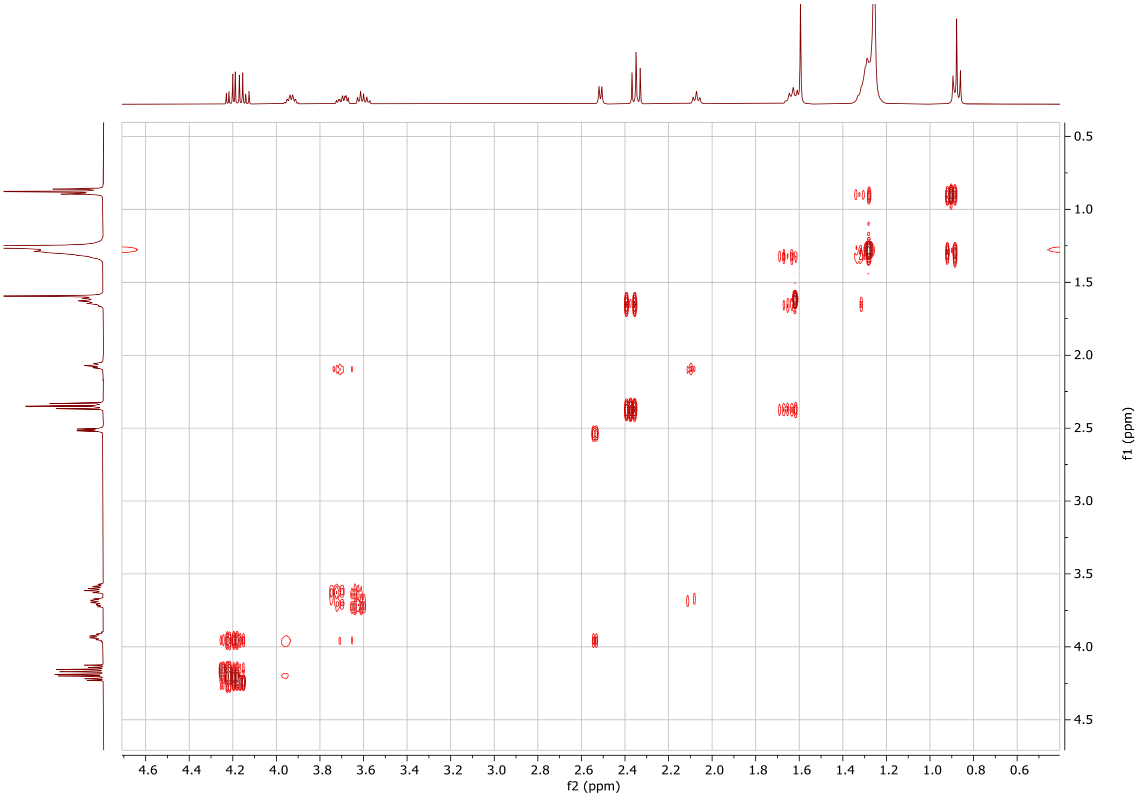

^{13}C-^{1}H HSQC spectrum of compound (*R*)-**17**

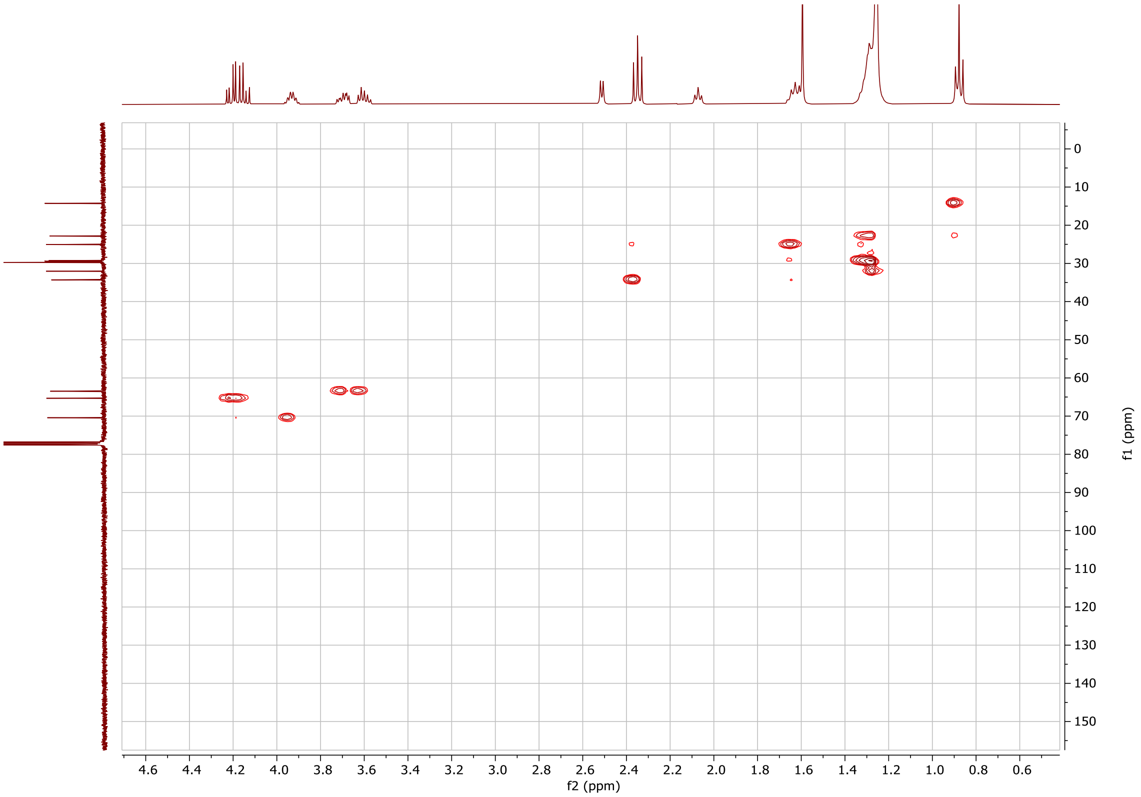

¹H NMR (400 MHz, $CDCl_3$) of compound (*R*)-**18**

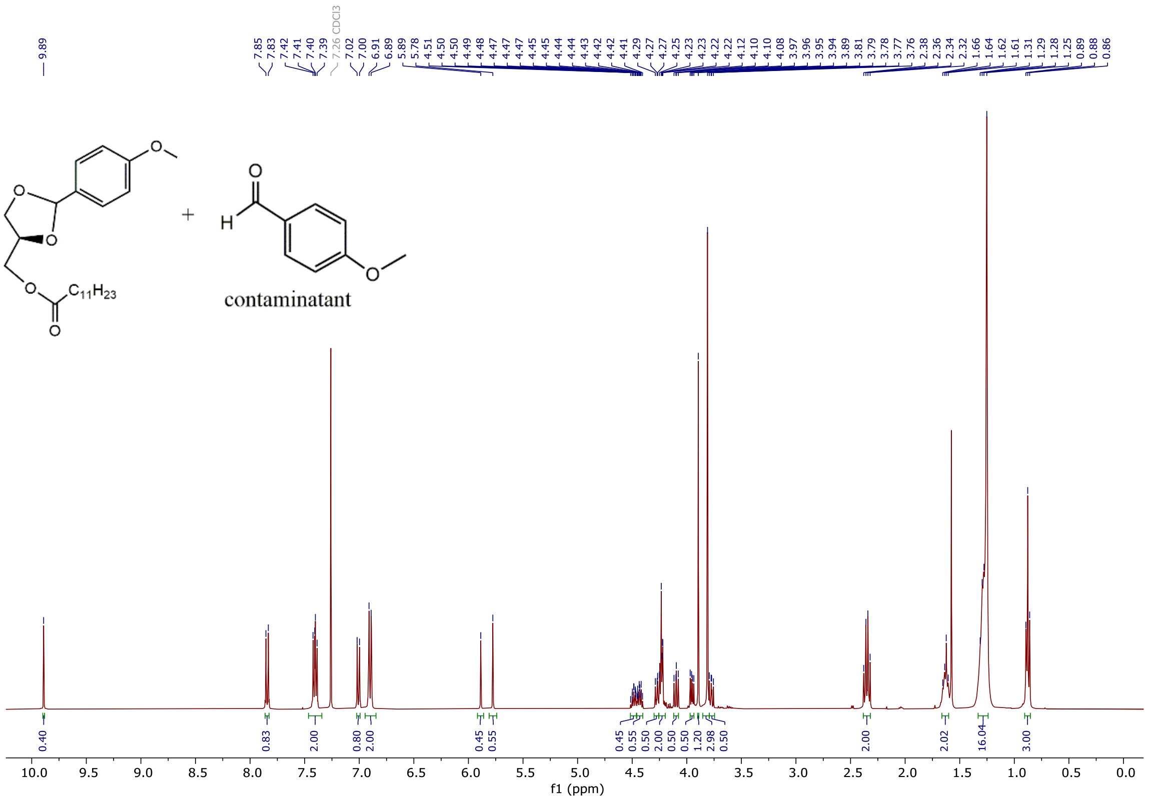

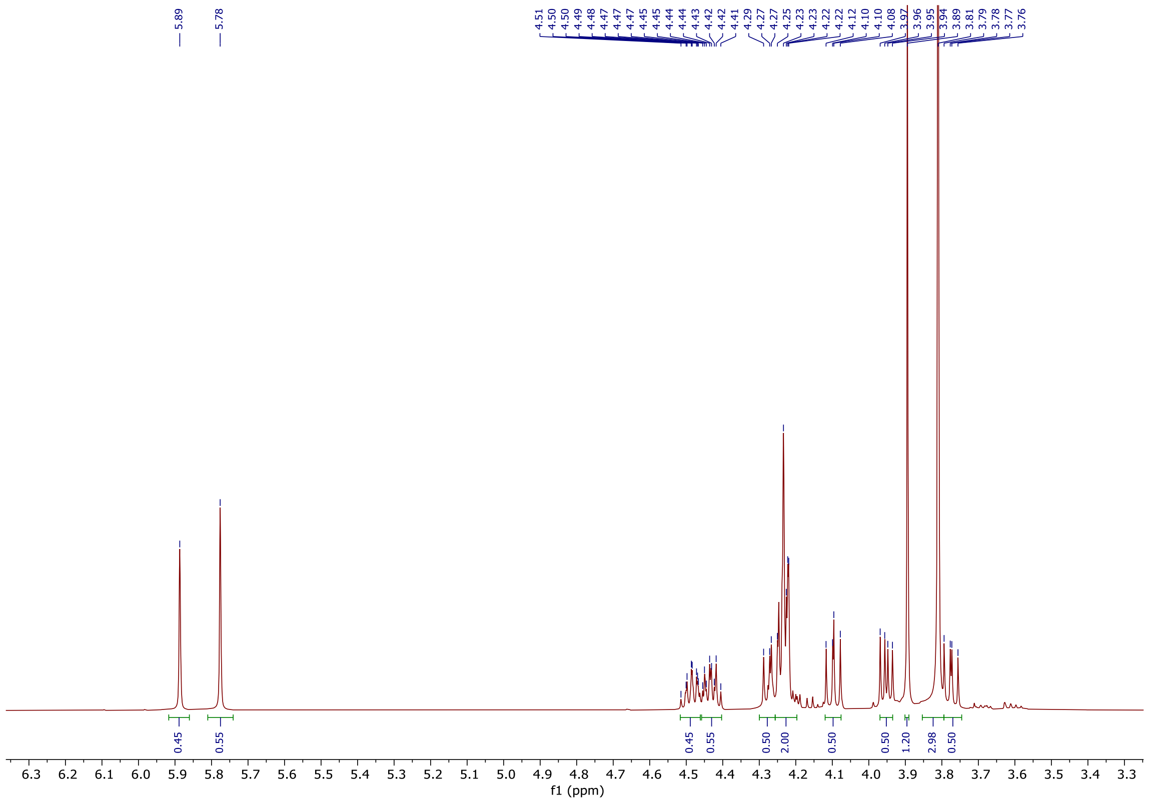

$^{13}C\{H\}$ NMR (101 MHz, $CDCl_3$) of compound (*R*)-**18**

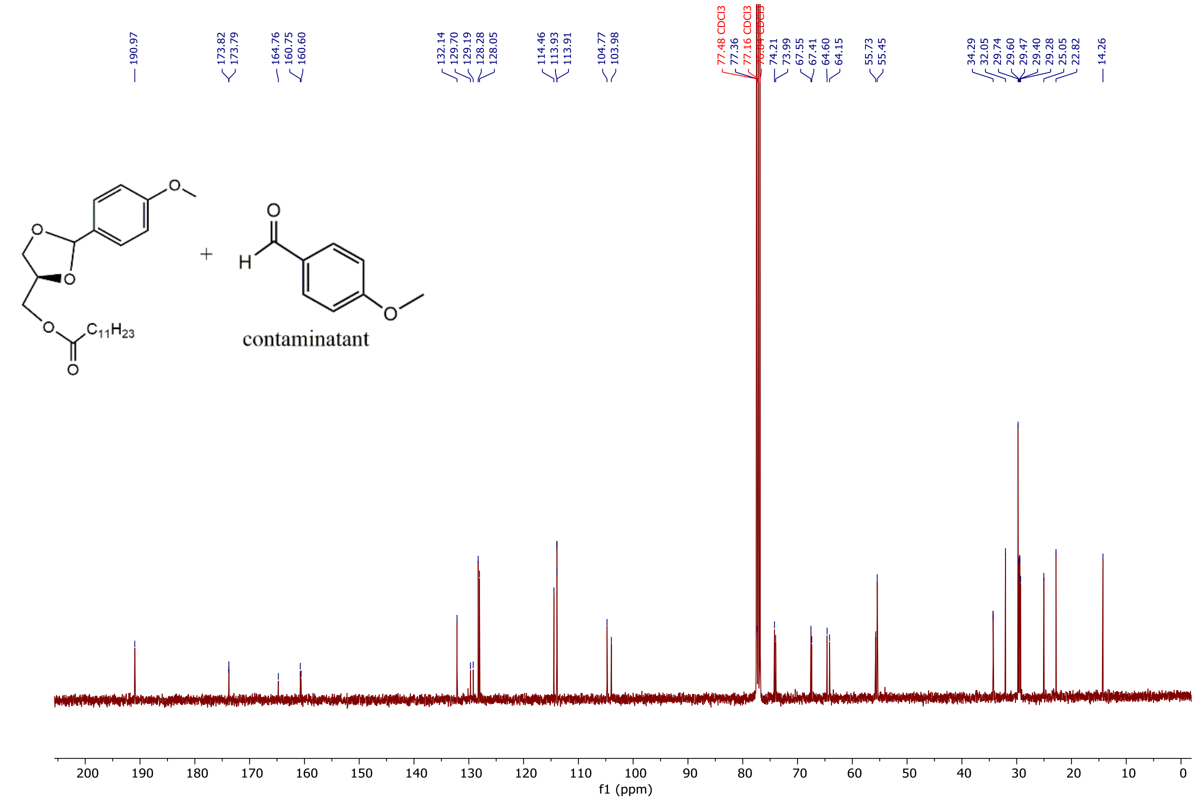

^1H-^1H COSY spectrum of compound (*R*)-**18**

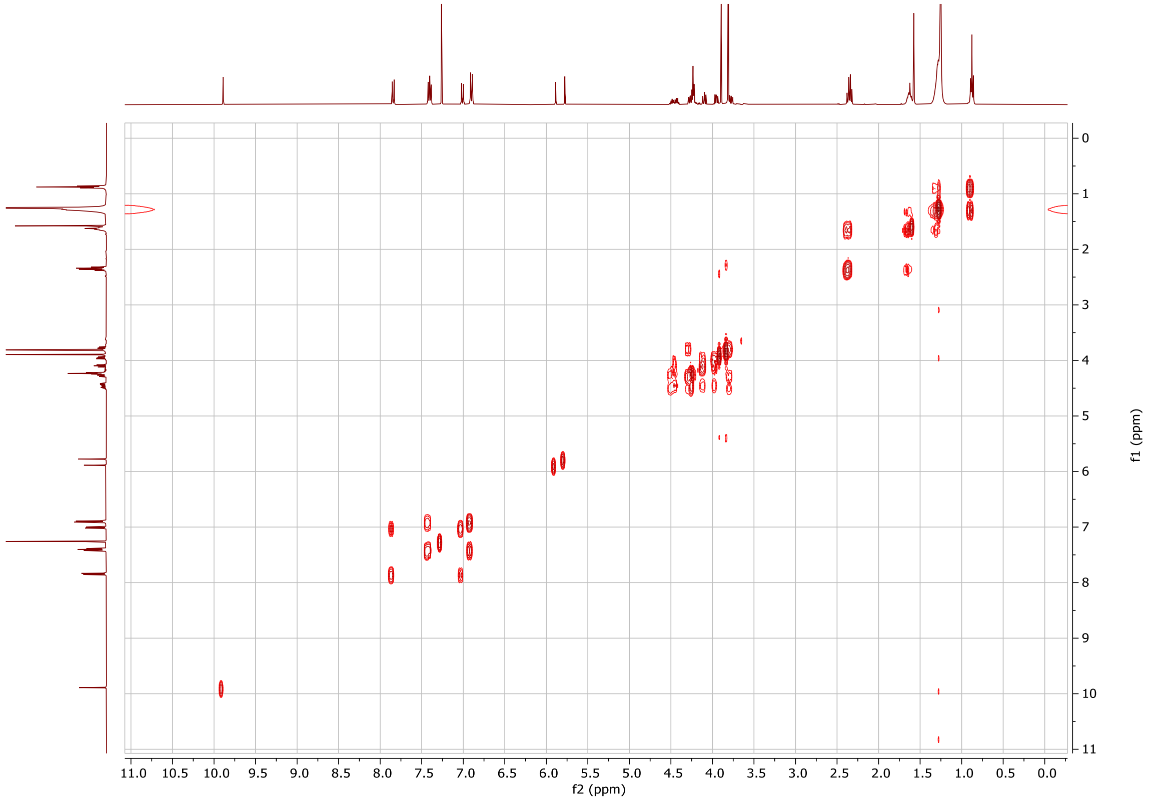

^{13}C-^{1}H HSQC spectrum of compound (*R*)-**18**

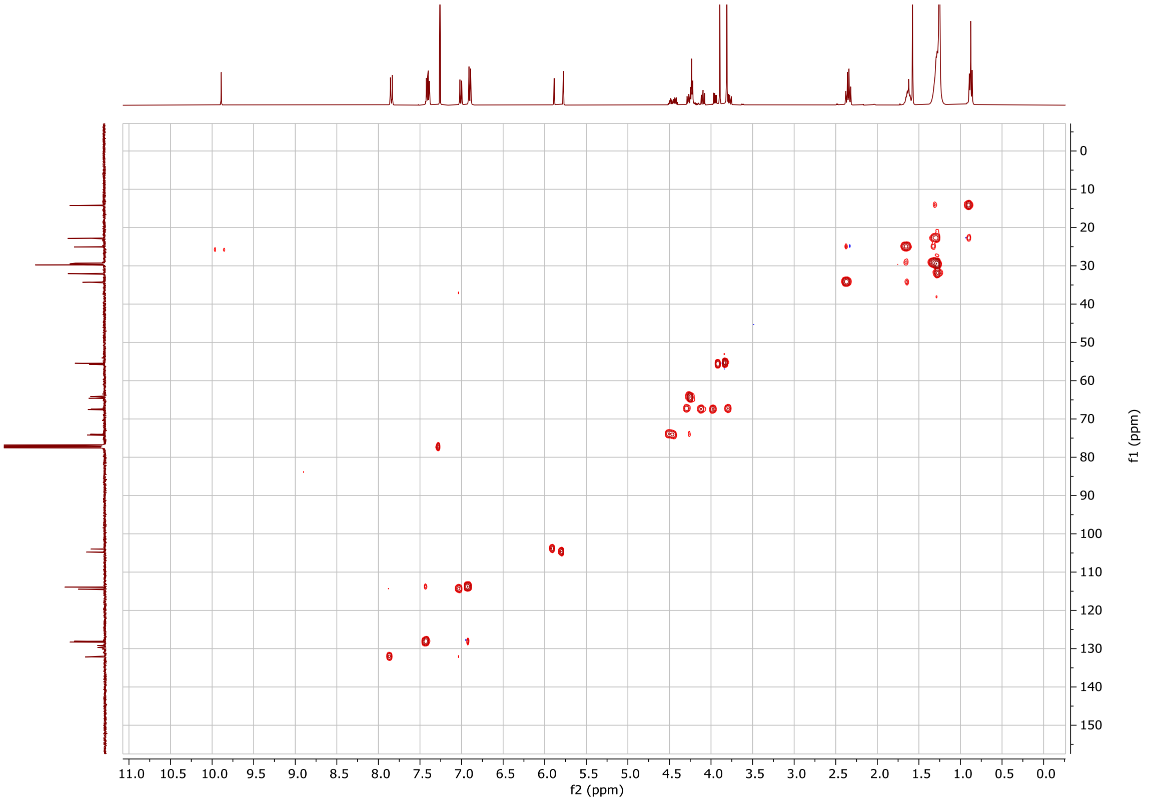

^{1}H NMR (400 MHz, $CDCl_3$) of compound (*S*)-**7**

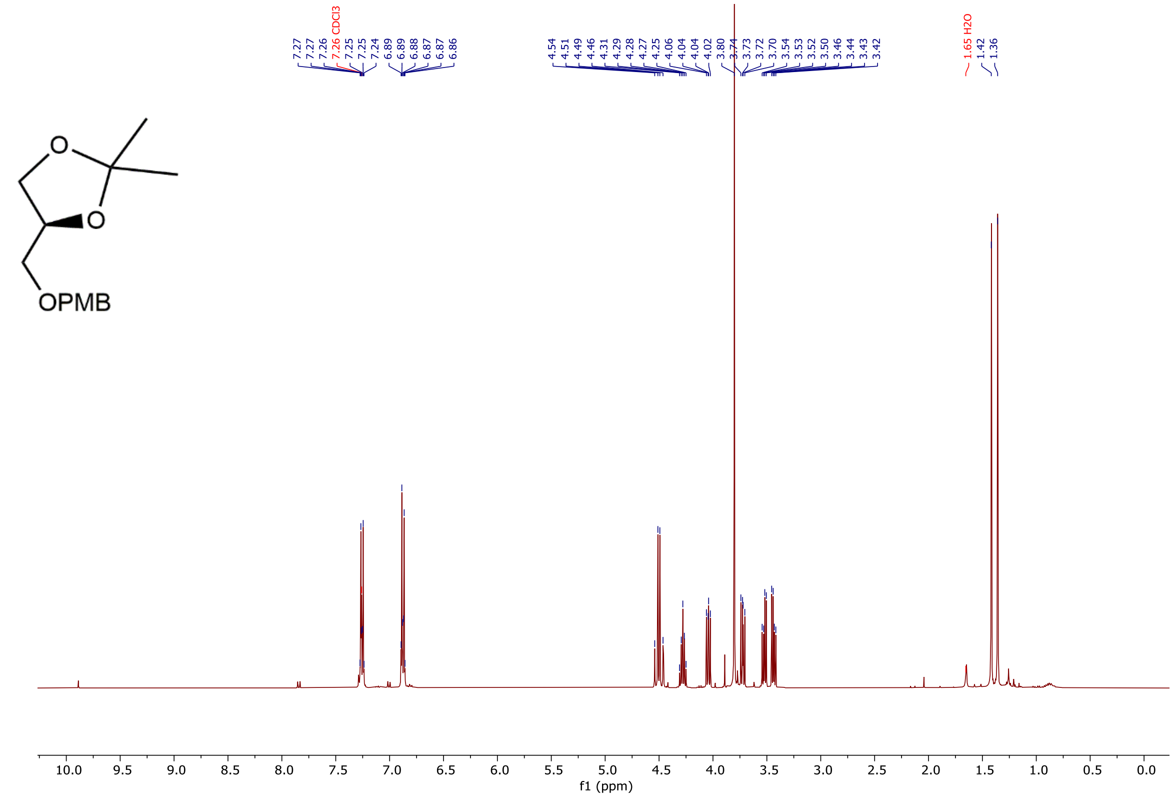

^{13}C{H} NMR (101 MHz, $CDCl_3$) of compound (*S*)-**7**

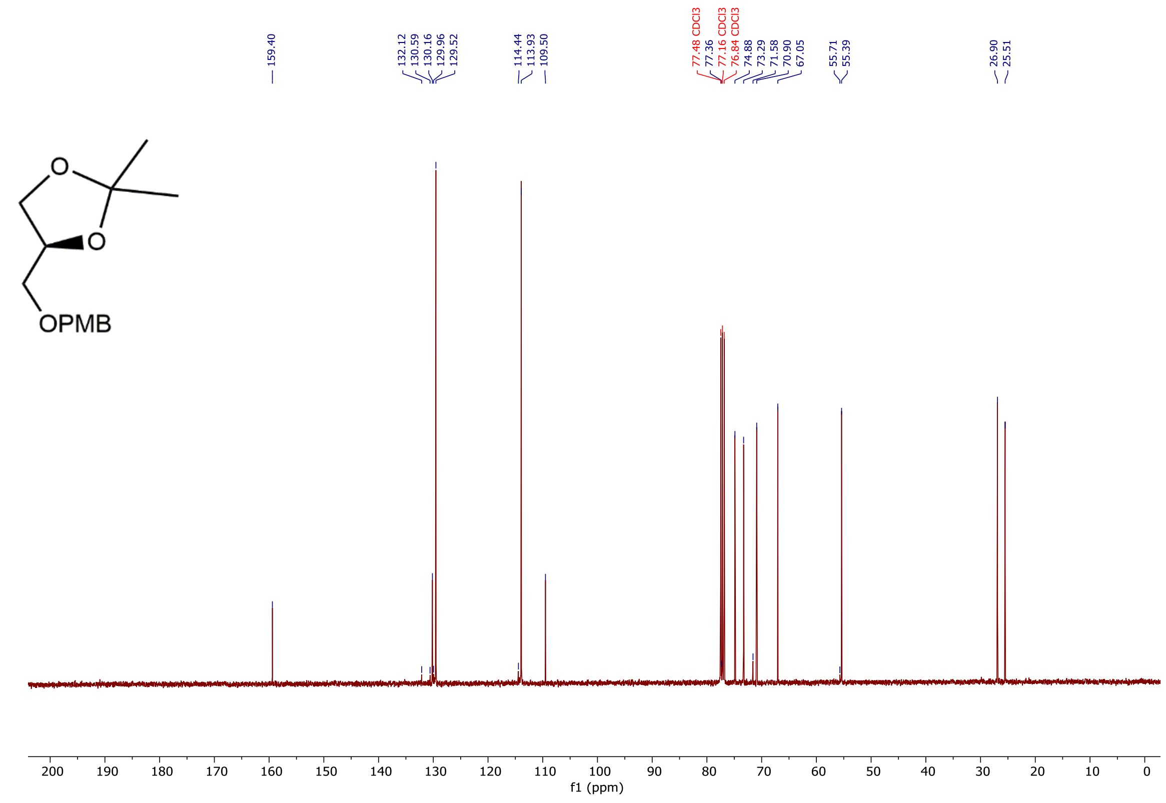

^1H-^1H COSY spectrum of compound (*S*)-**7**

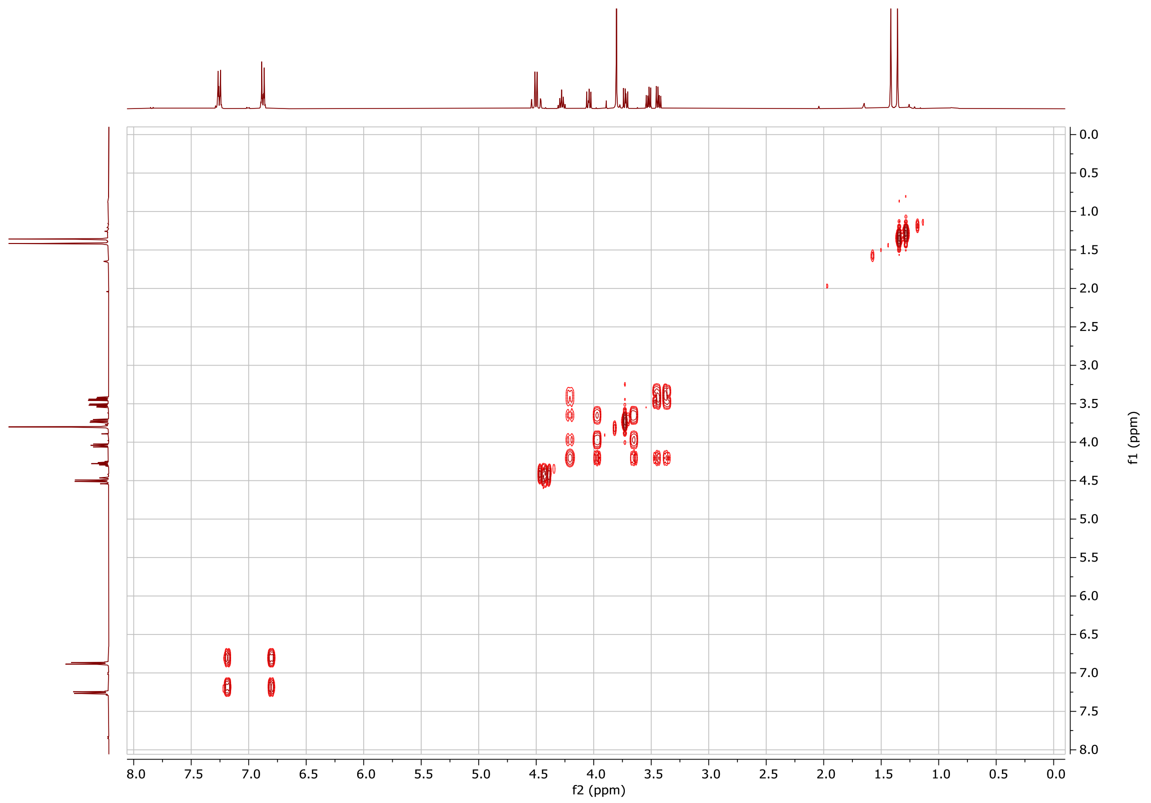

^{13}C-^1H HSQC spectrum of compound (*S*)-**7**

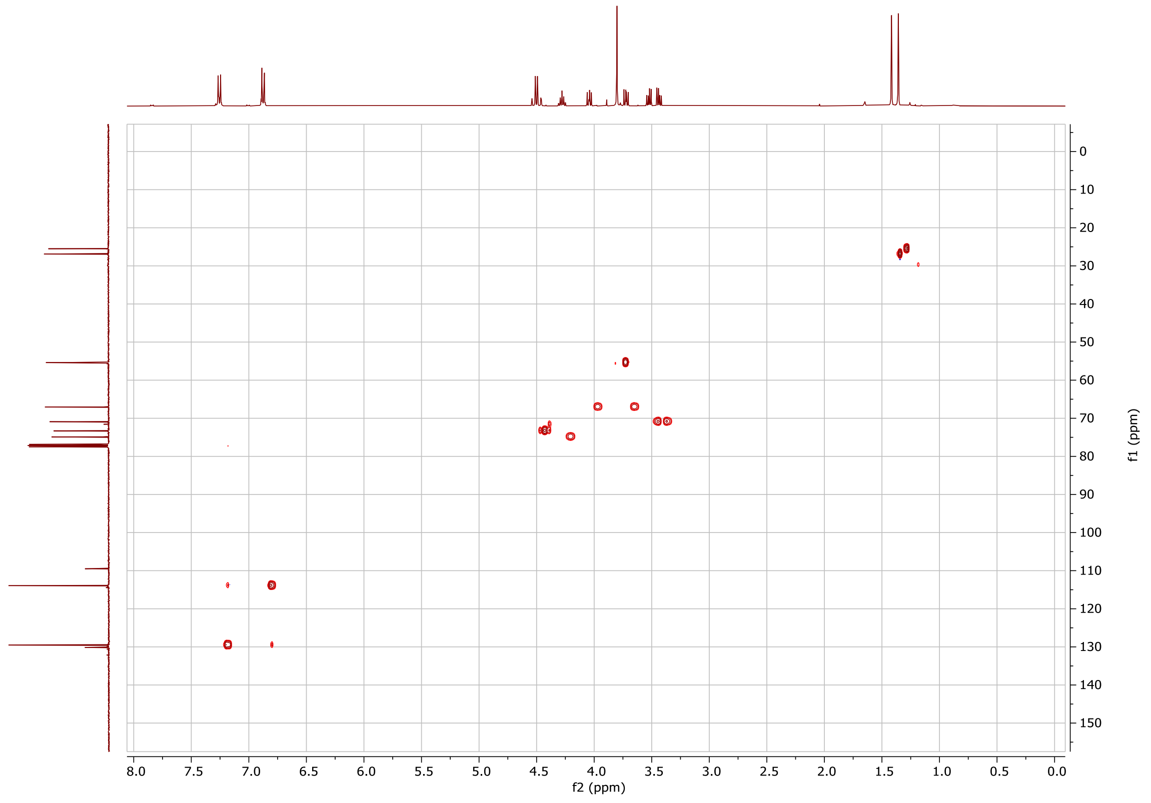

^{1}H NMR (400 MHz, $CDCl_3$) of compound (*R*)-**8**

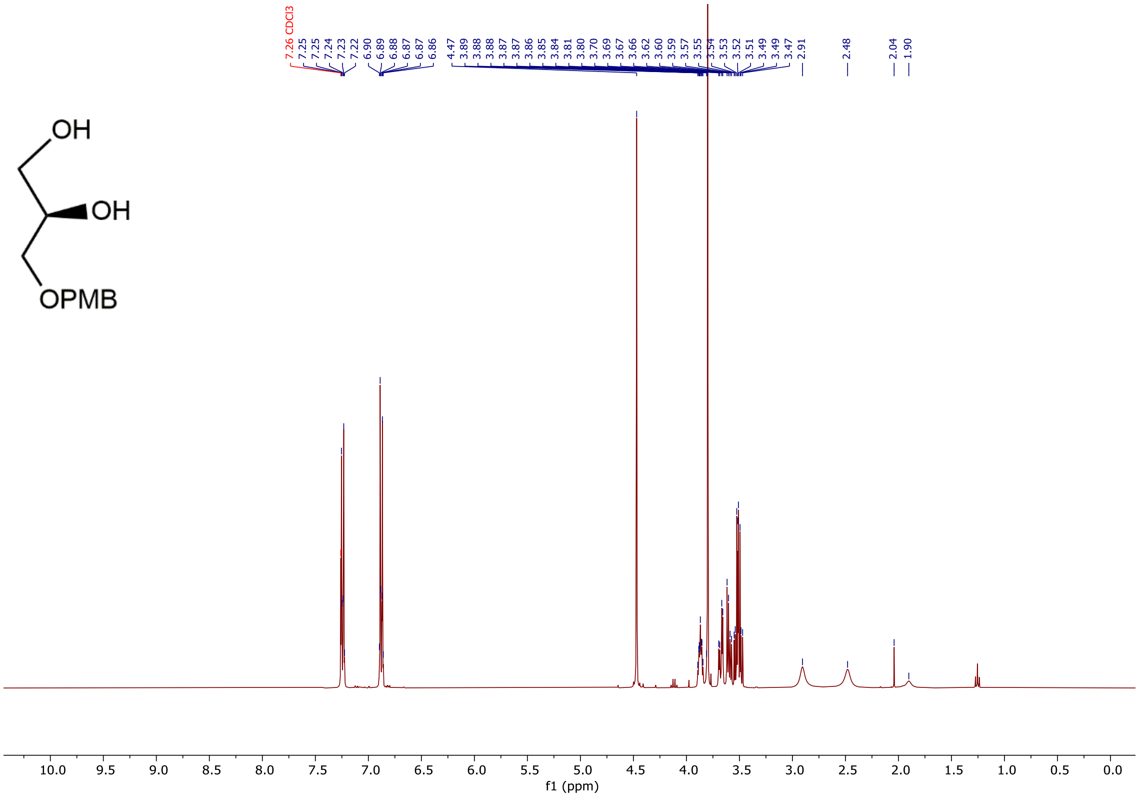

^{13}C{H} NMR (101 MHz, $CDCl_3$) of compound (*R*)-**8**

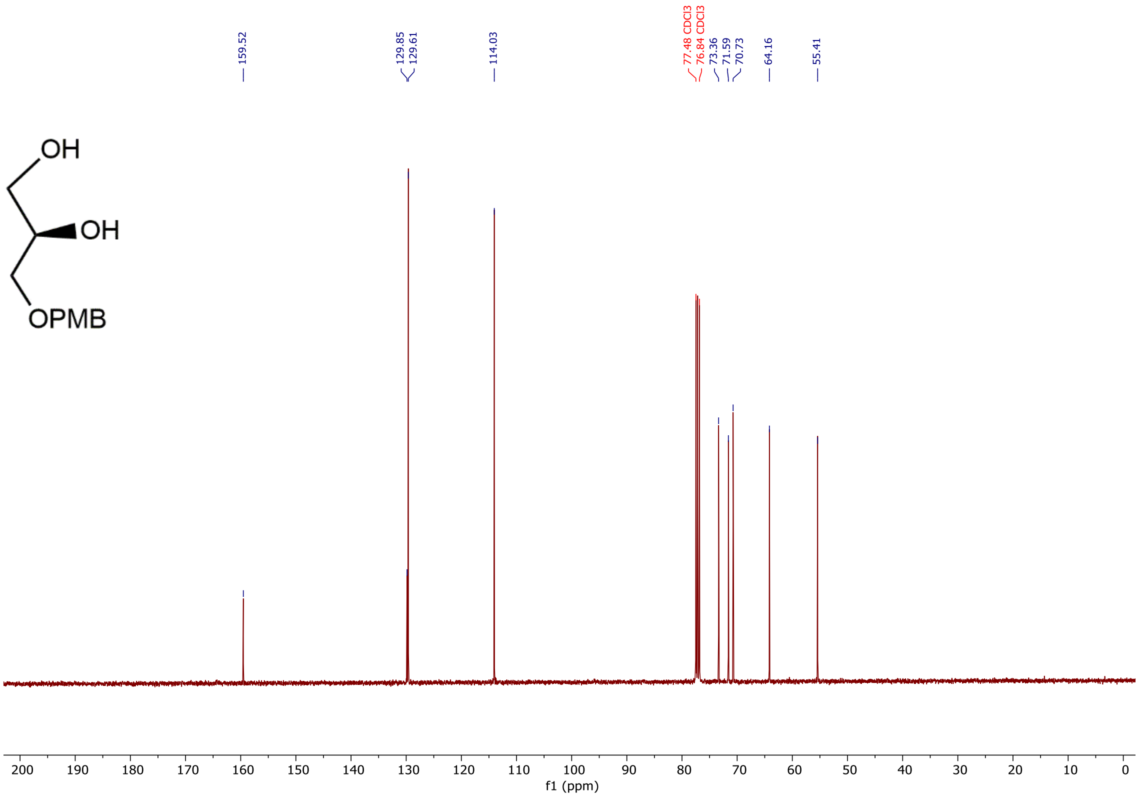

^{1}H-^{1}H COSY spectrum of compound (*R*)-**8**

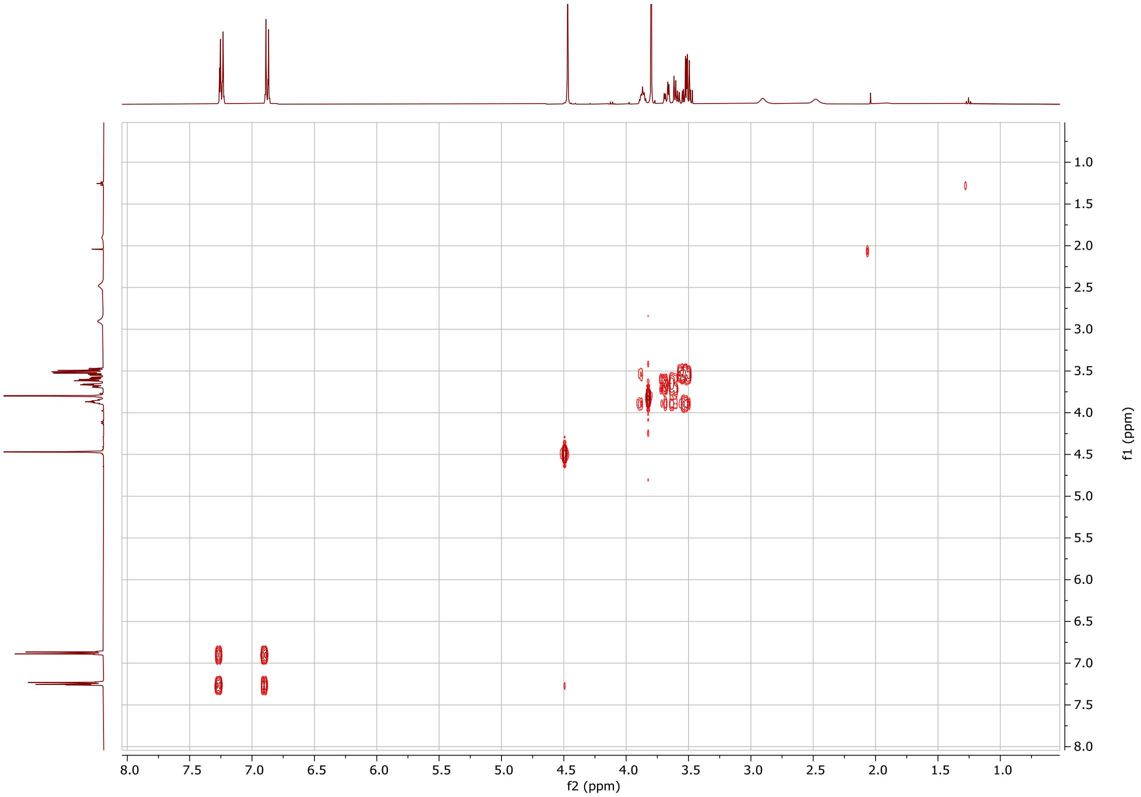

^{13}C-^{1}H HSQC spectrum of compound (*R*)-**8**

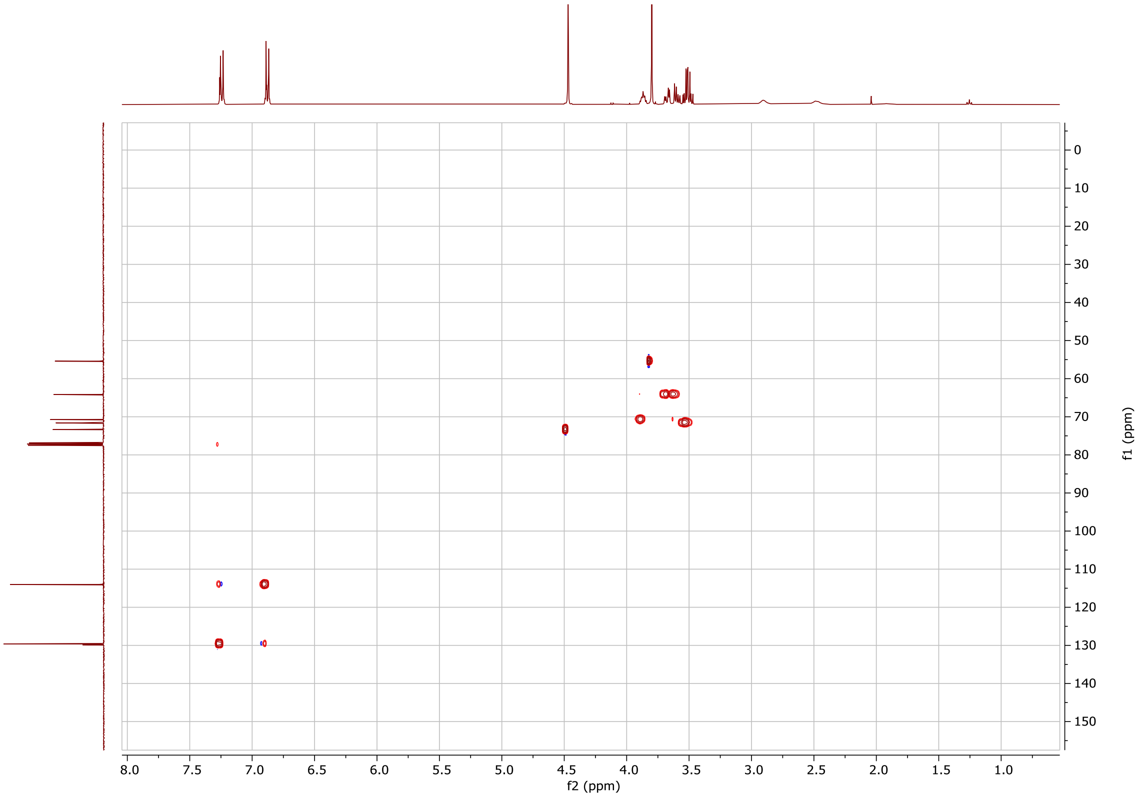

^{1}H NMR (400 MHz, $CDCl_3$) of compound (*S*)-**14**

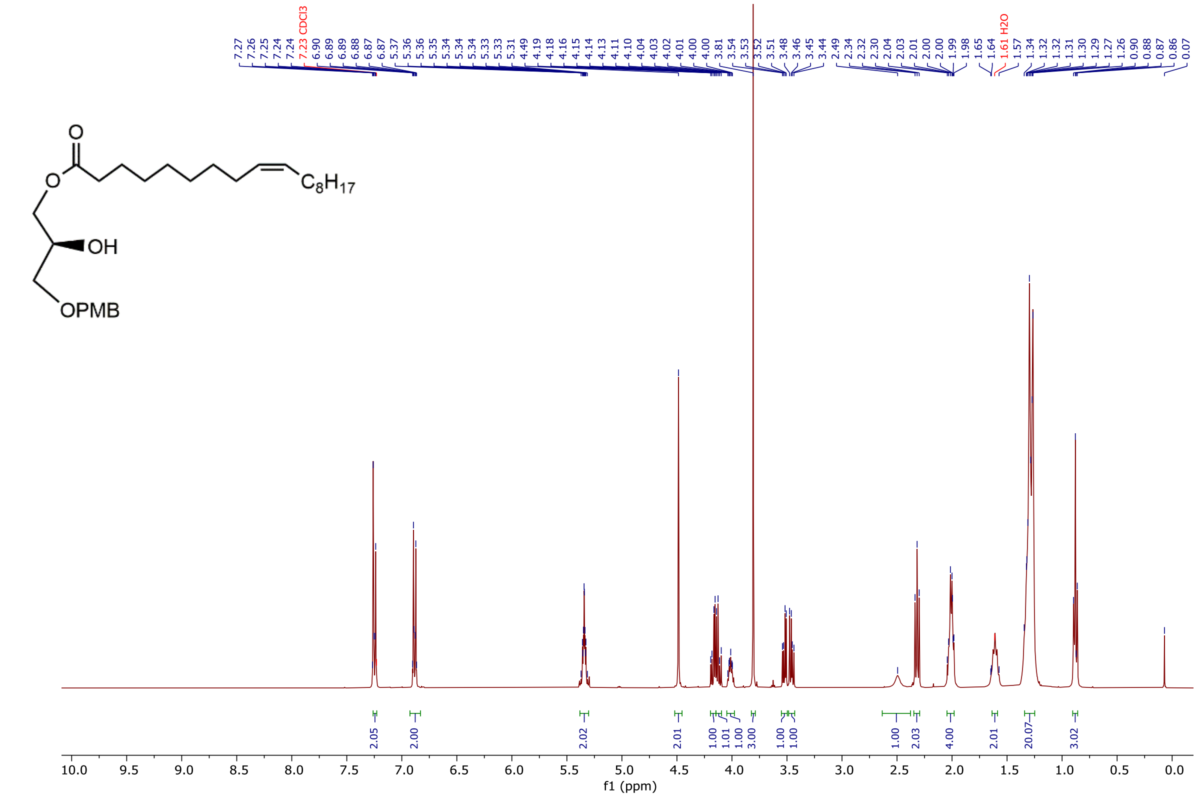

$^{13}C\{H\}$ NMR (101 MHz, $CDCl_3$) of compound (*S*)-**14**

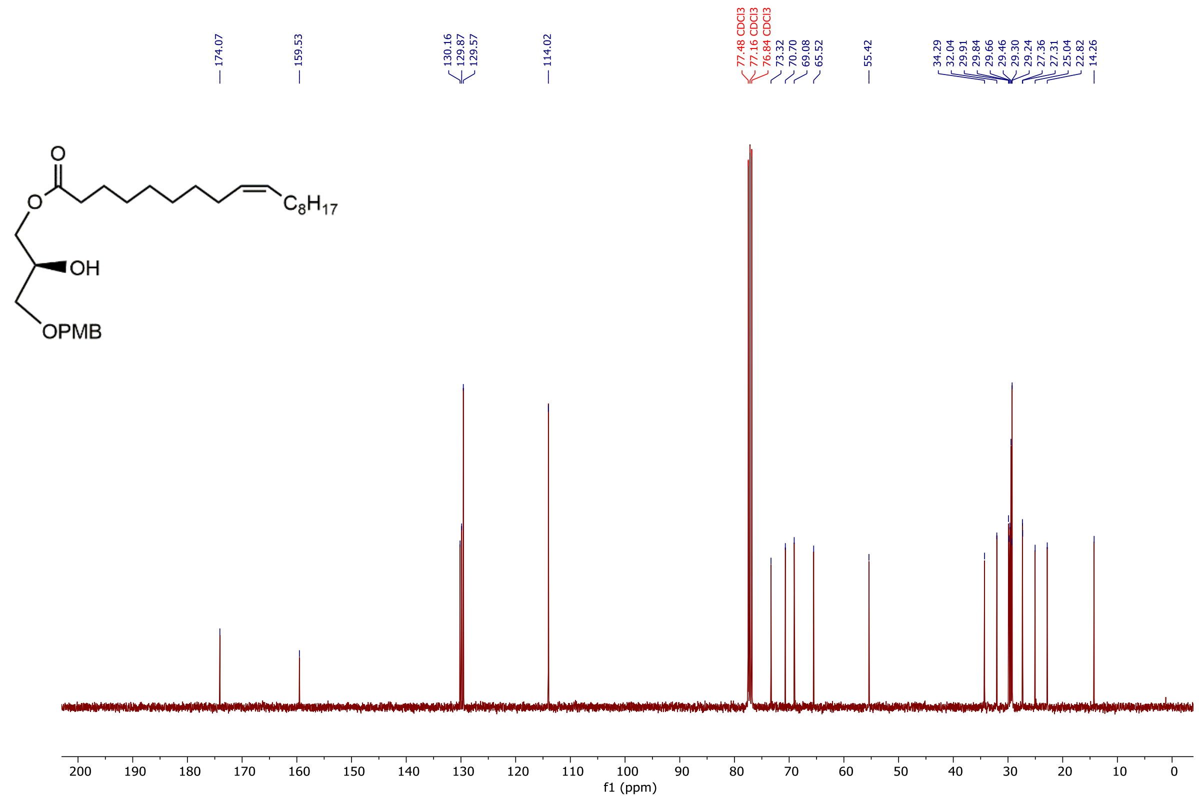

^{1}H-^{1}H COSY spectrum of compound (*S*)-**14**

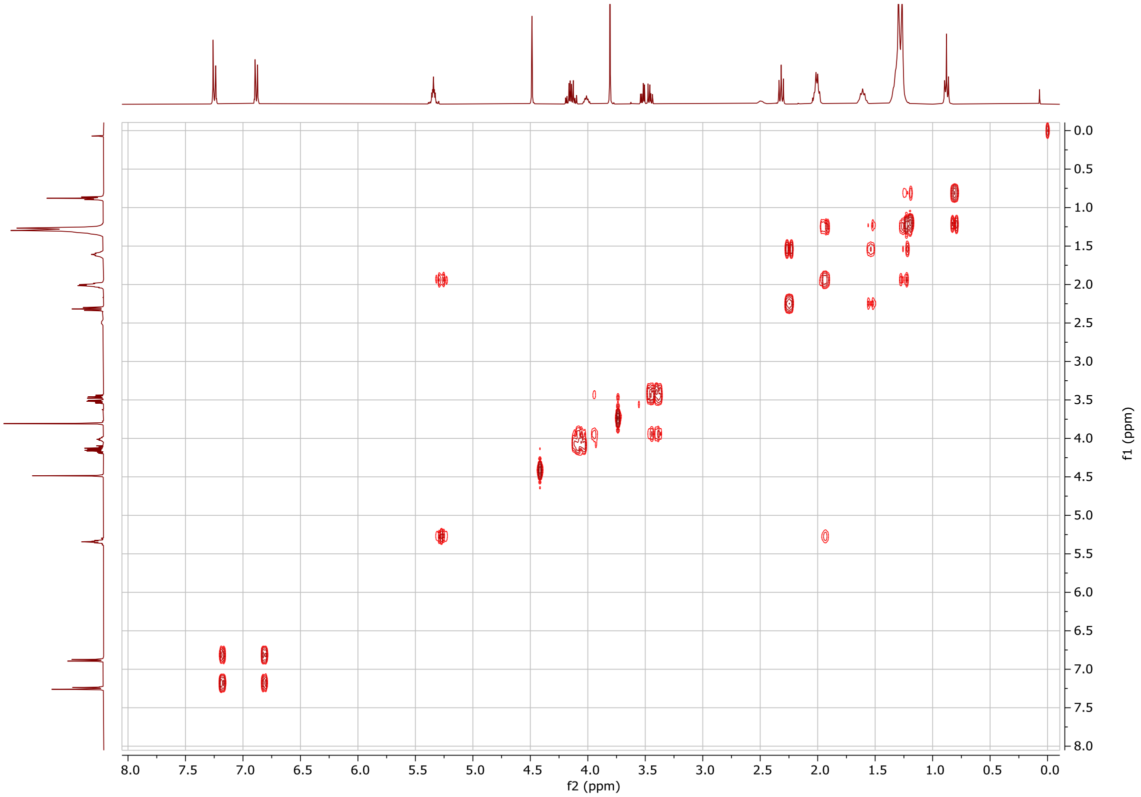

^{13}C-^{1}H HSQC spectrum of compound (*S*)-**14**

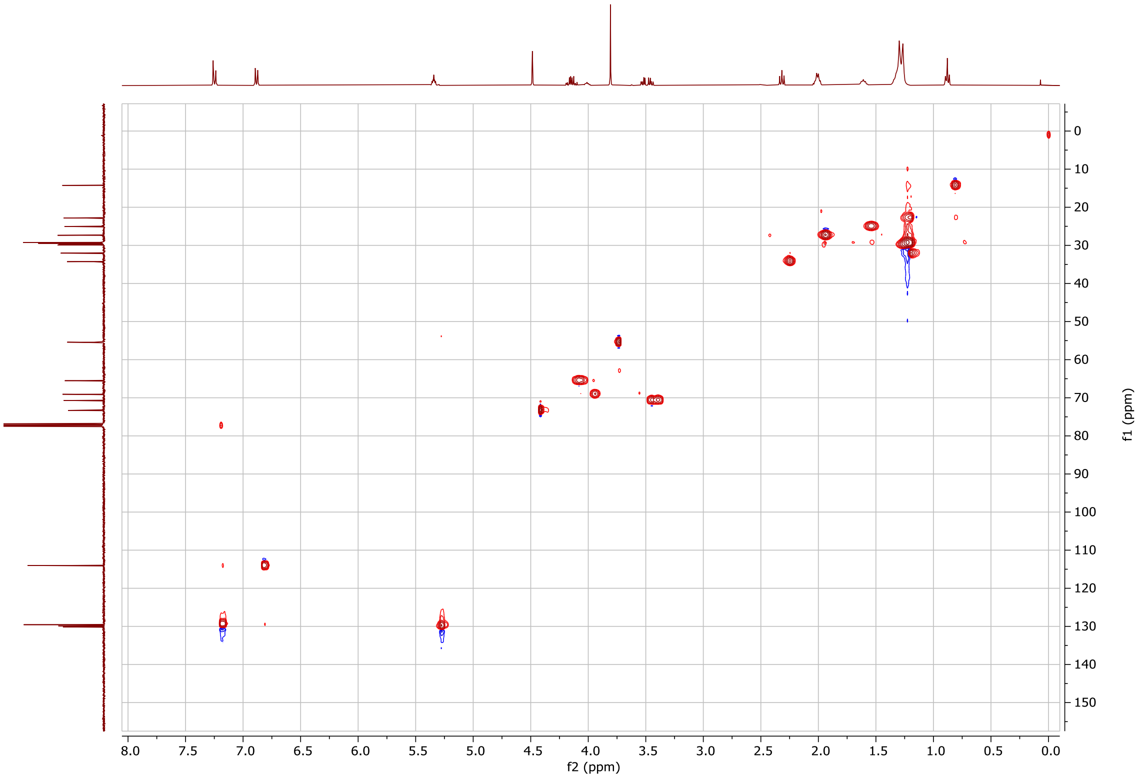

^{1}H NMR (400 MHz, $CDCl_3$) of compound (*S*)-**19**

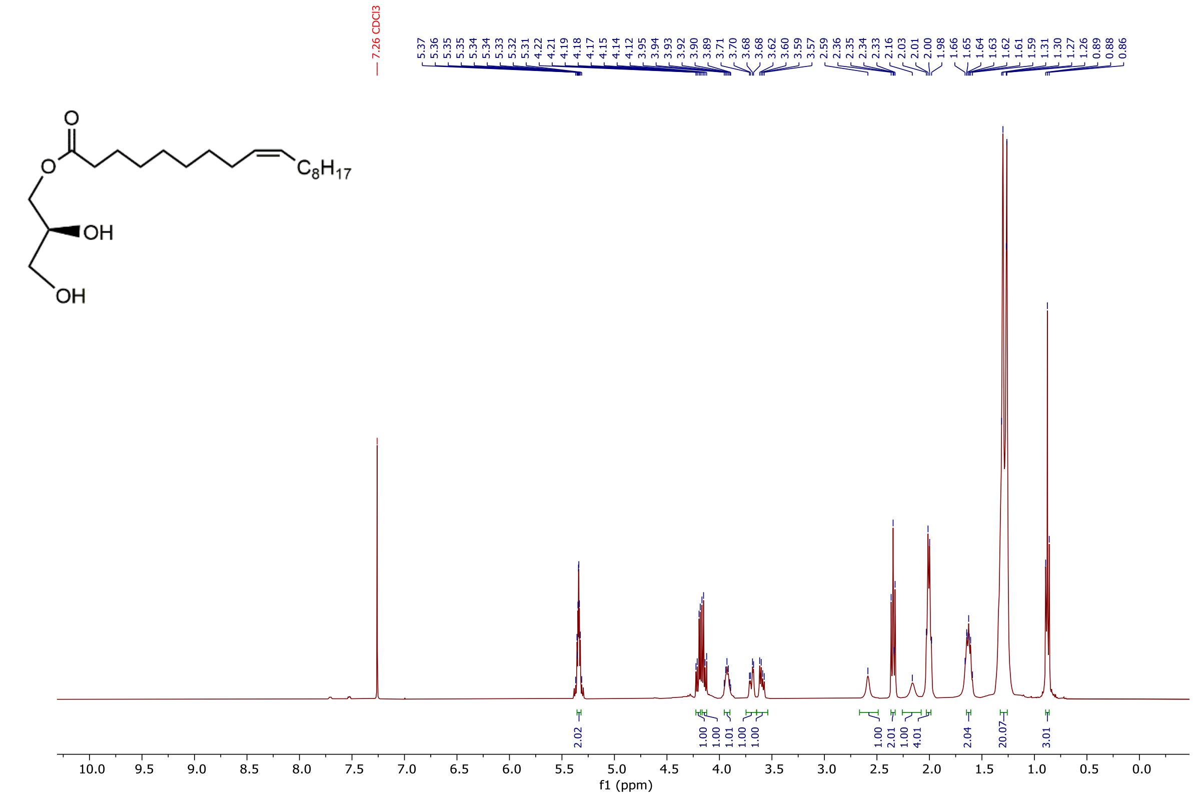

$^{13}C\{H\}$ NMR (101 MHz, $CDCl_3$) of compound (*S*)-**19**

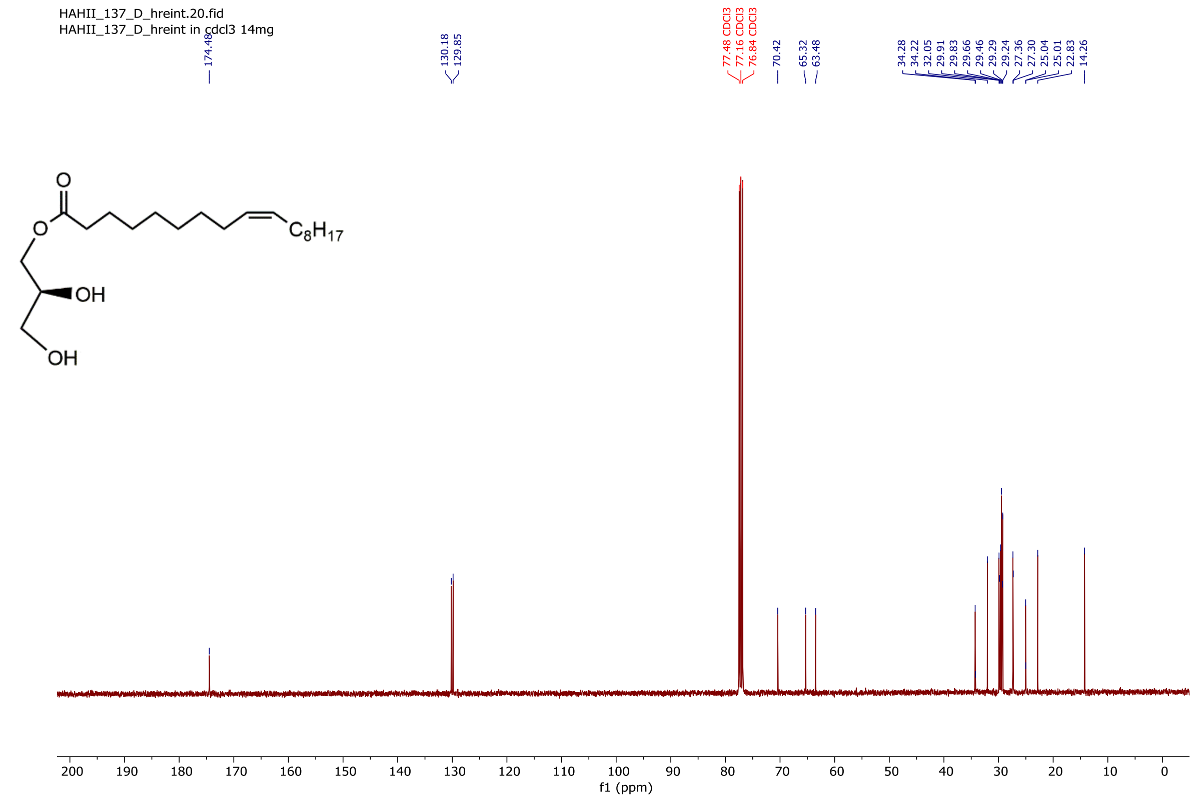

^{1}H-^{1}H COSY spectrum of compound (*S*)-**19**

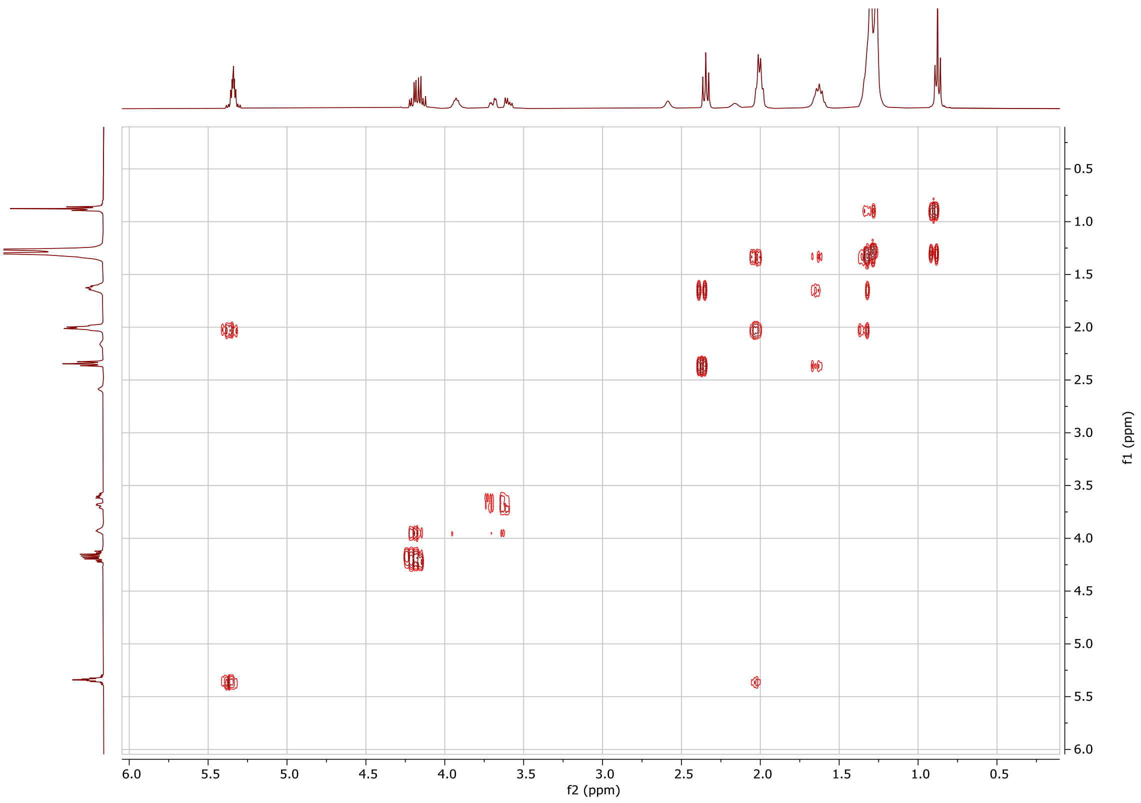

^{13}C-^{1}H HSQC spectrum of compound (*S*)-**19**

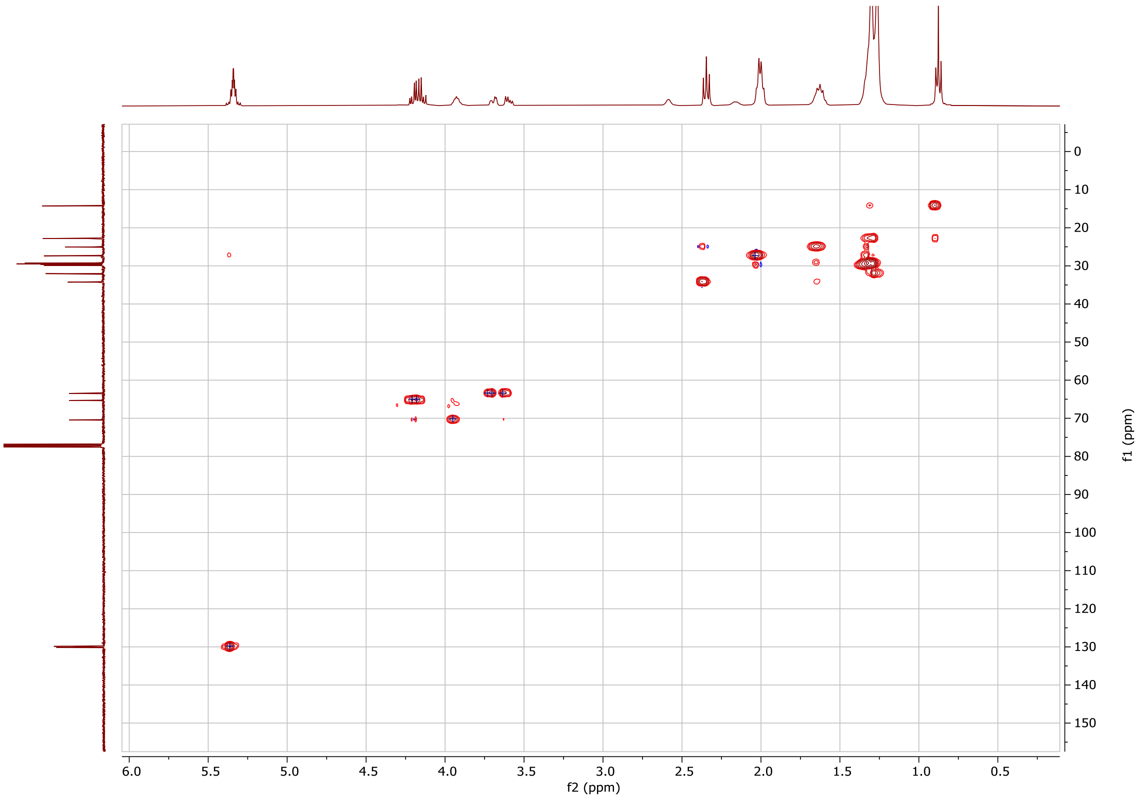

^{1}H NMR (400 MHz, $CDCl_3$) of compound (*S*)-**20a**

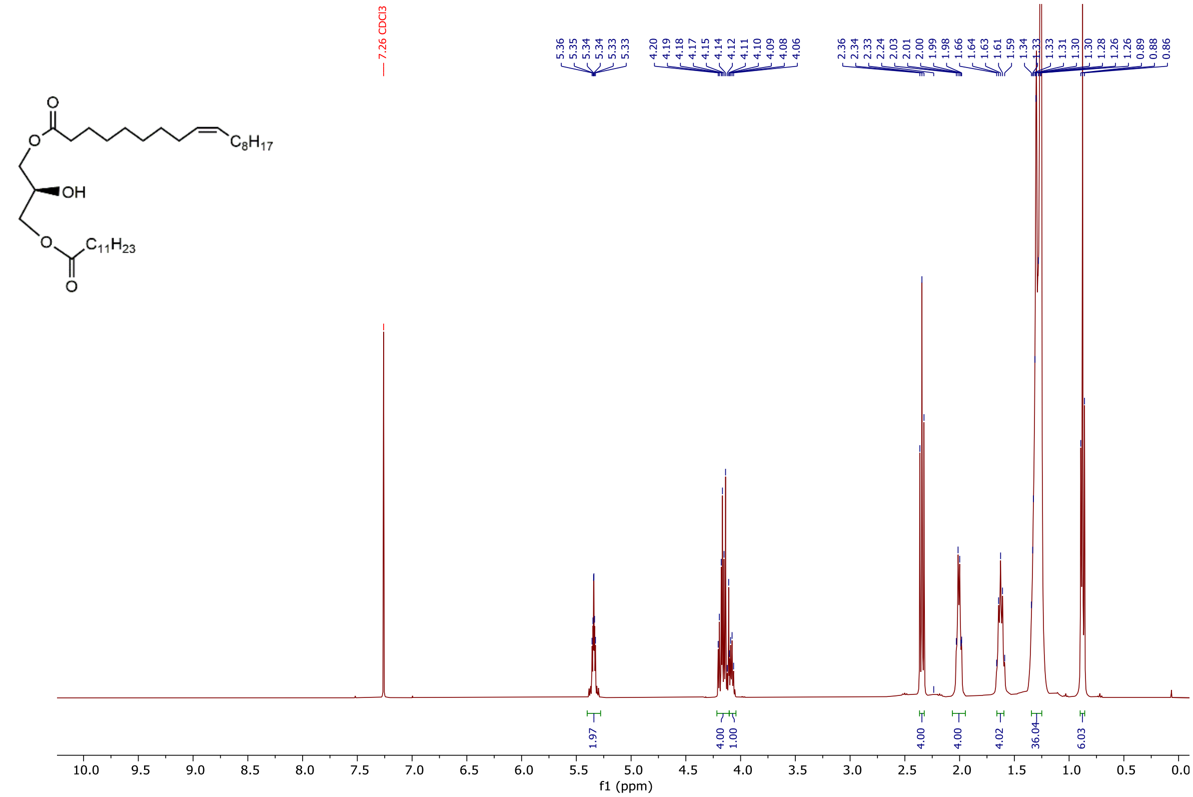

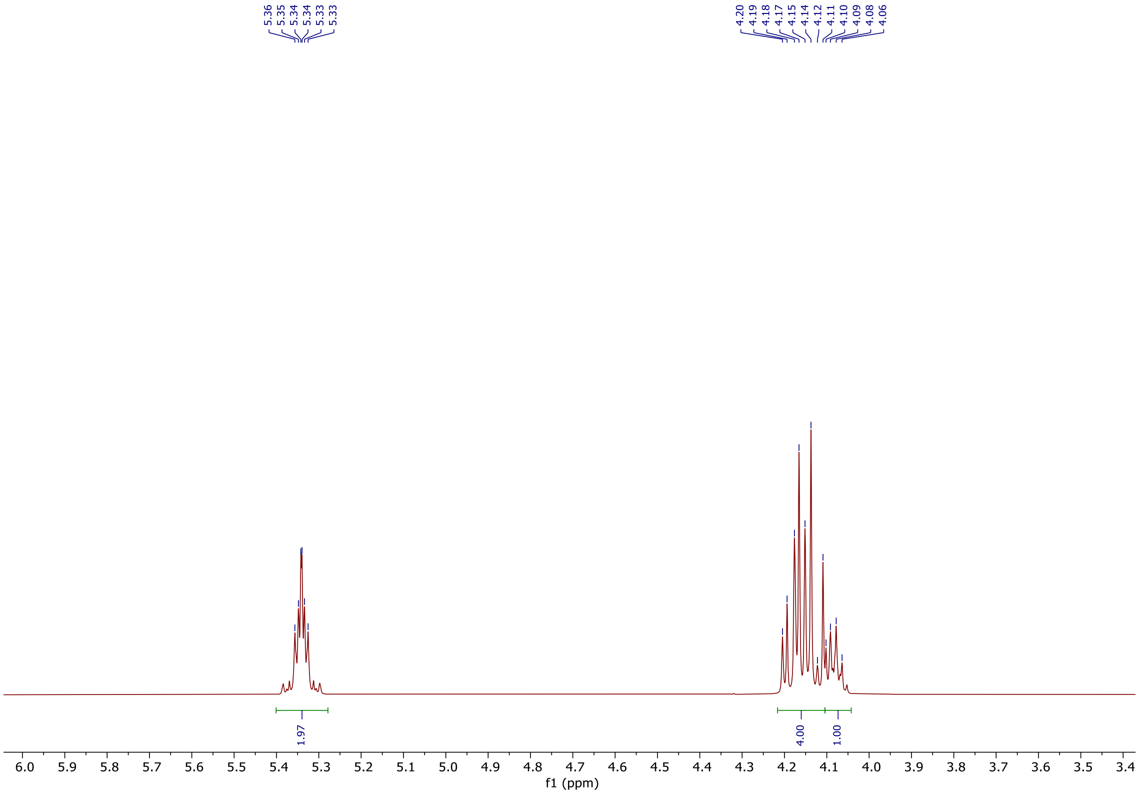

^{13}C{H} NMR (101 MHz, $CDCl_3$) of compound (*S*)-**20a**

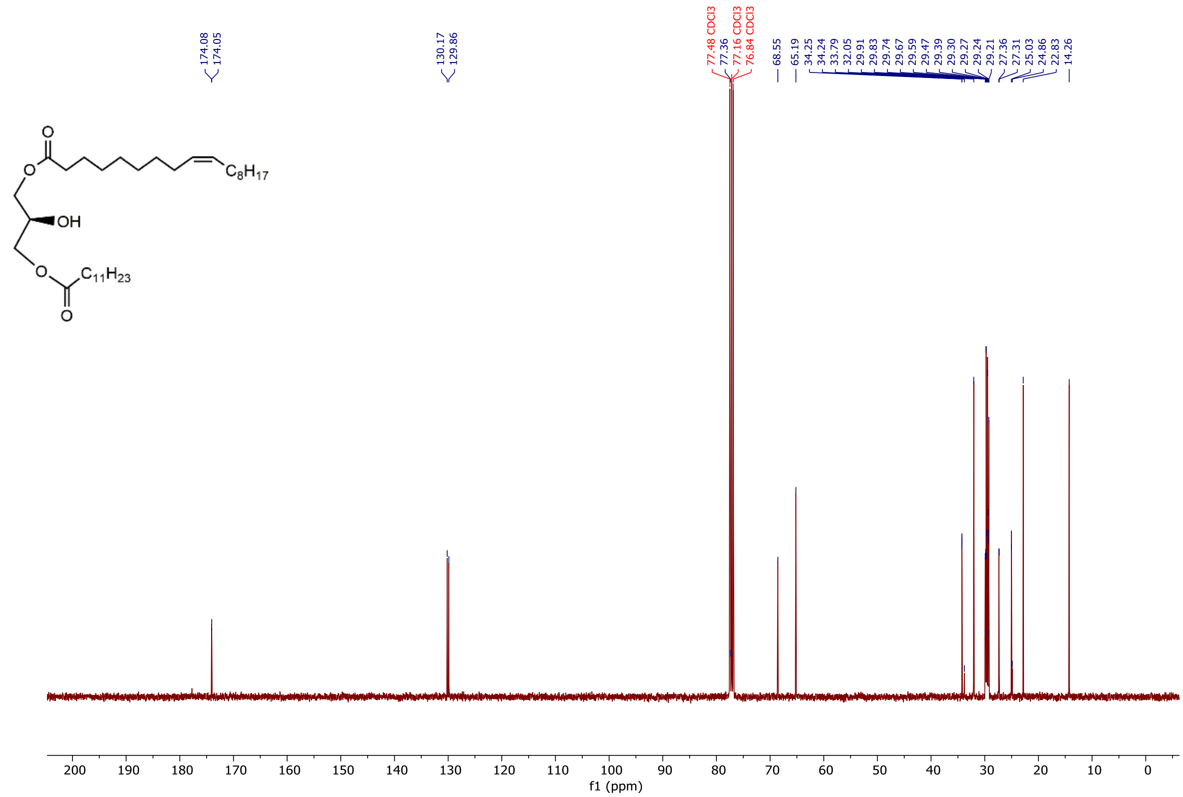

^{1}H-^{1}H COSY spectrum of compound (*S*)-**20a**

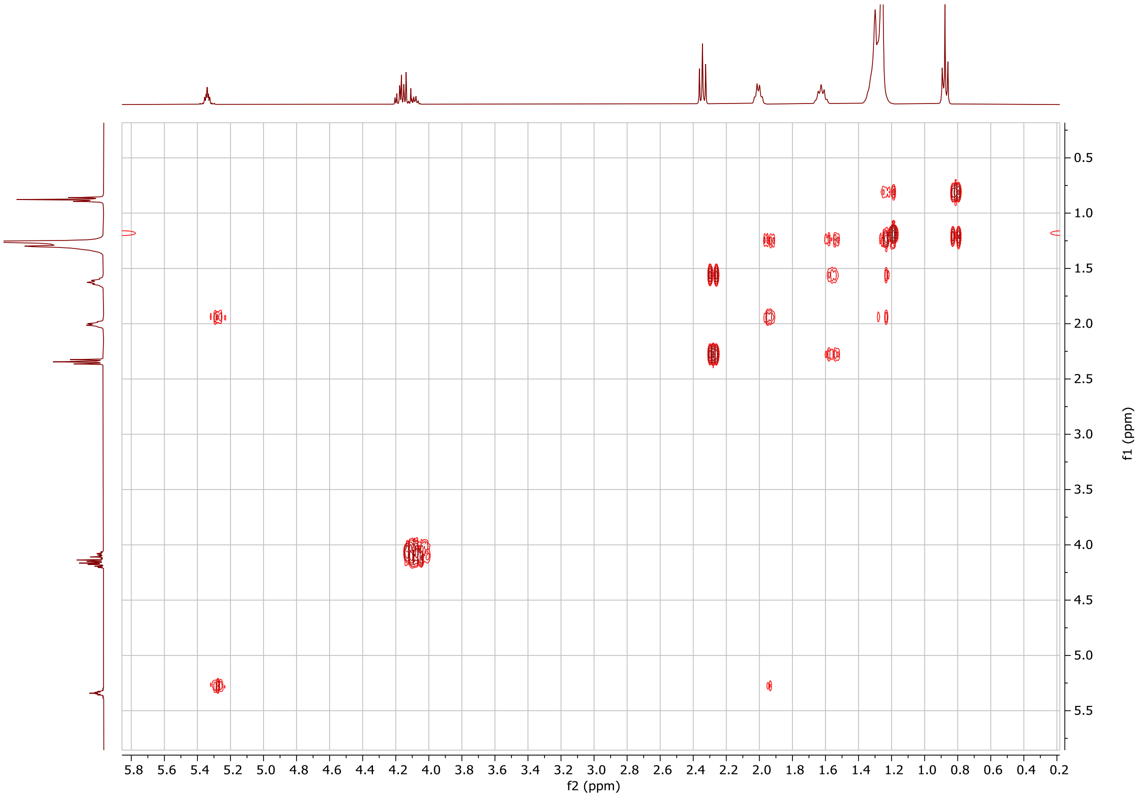

^{13}C-^{1}H HSQC spectrum of compound (*S*)-**20a**

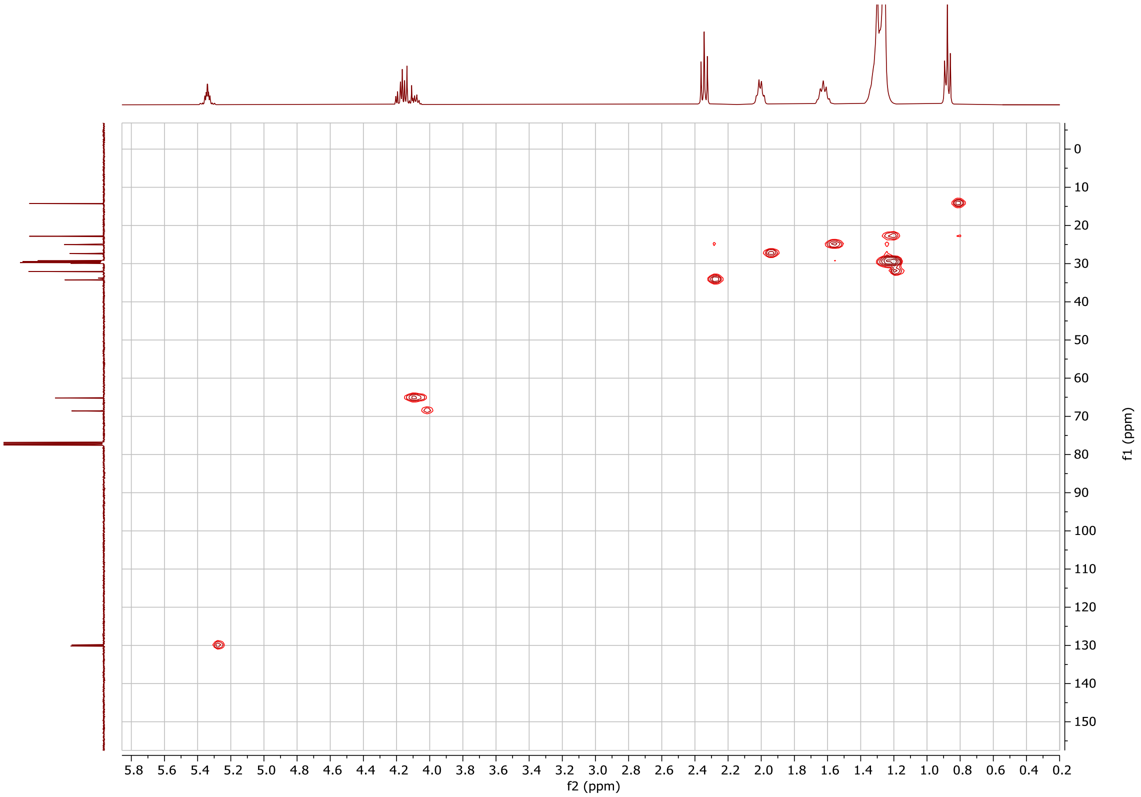

^{1}H NMR (400 MHz, $CDCl_3$) of compound (*S*)-**20b**

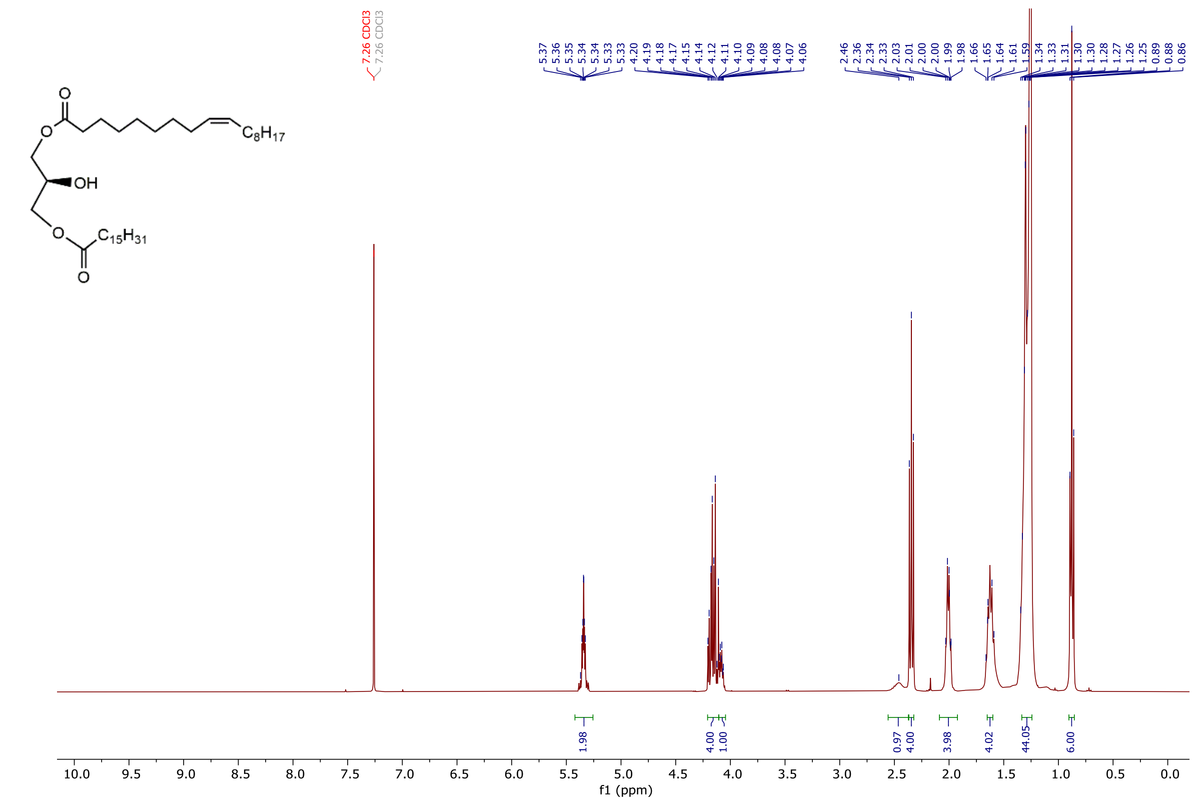

^{13}C{H} NMR (101 MHz, $CDCl_3$) of compound (*S*)-**20b**

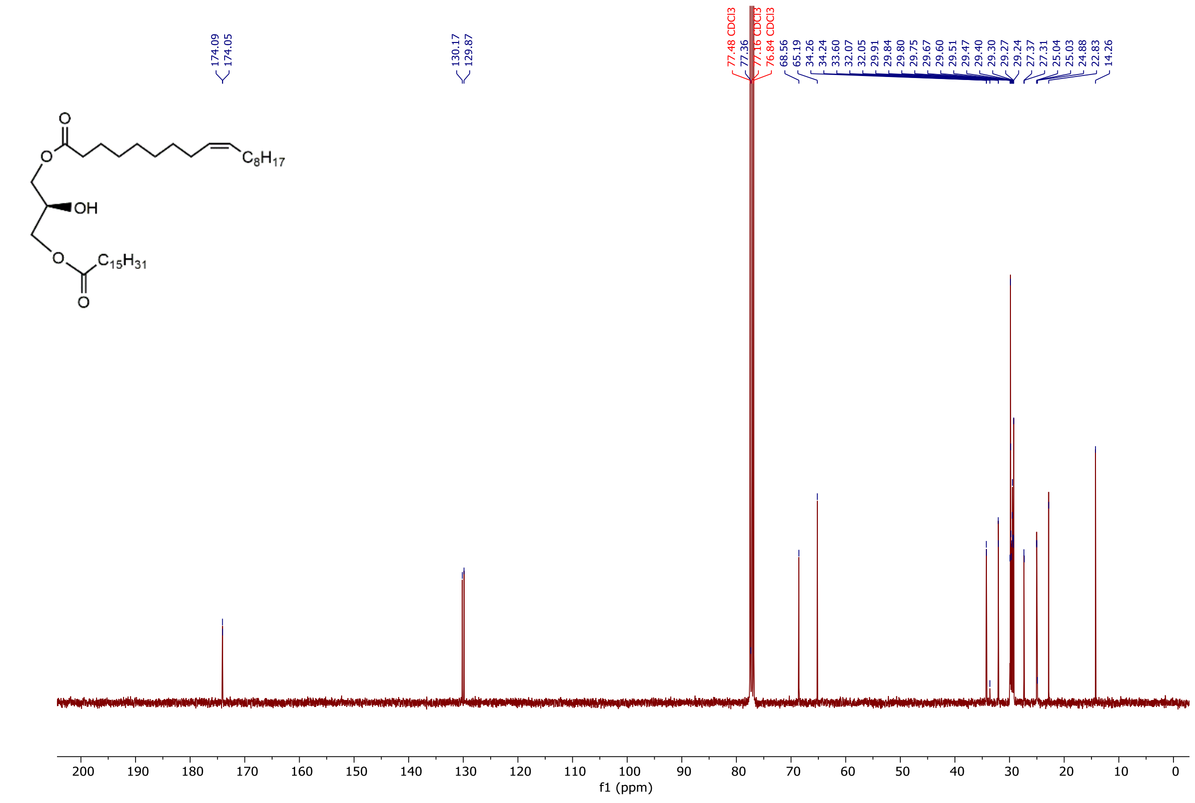

^{1}H-^{1}H COSY spectrum of compound (*S*)-**20b**

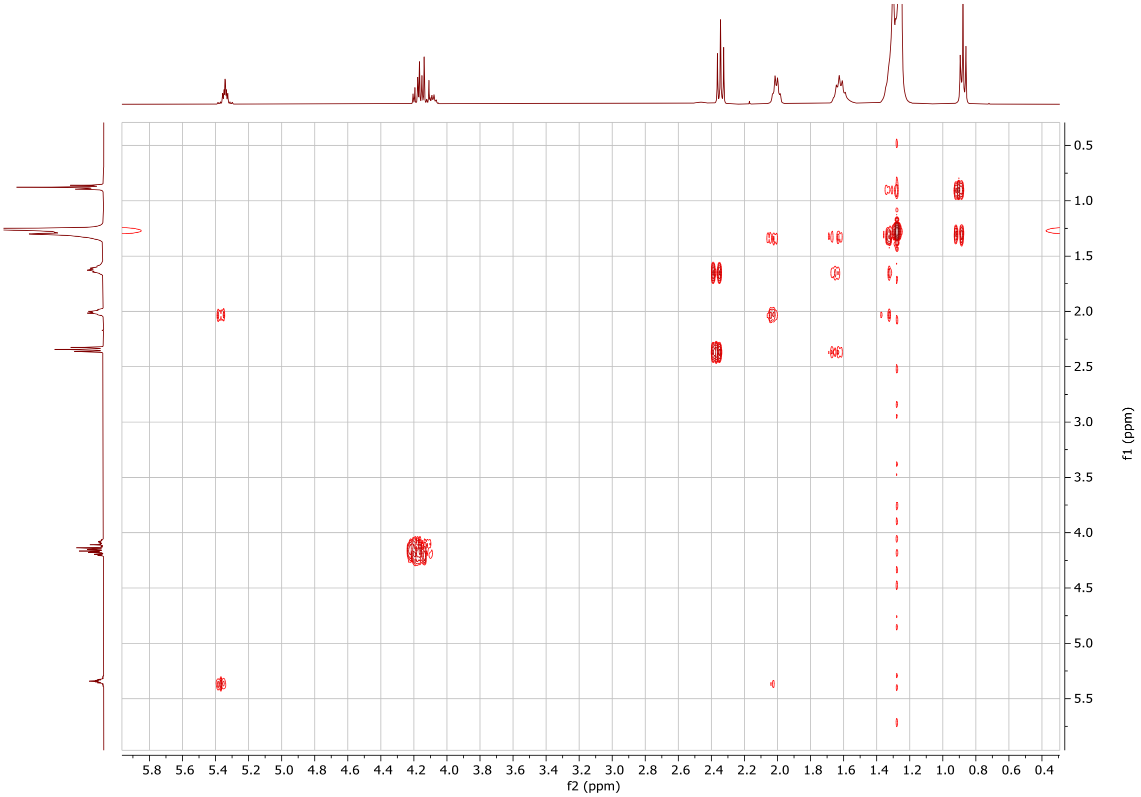

^{13}C-^{1}H HSQC spectrum of compound (*S*)-**20b**

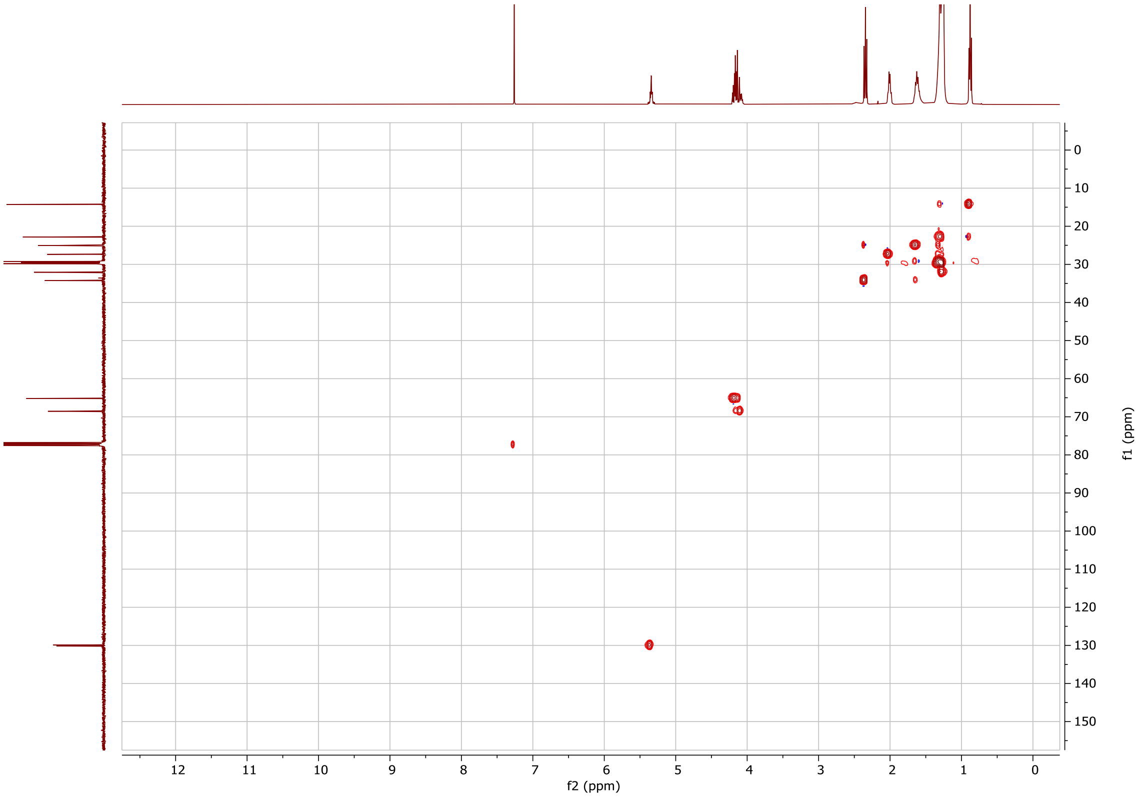

^{1}H NMR (400 MHz, $CDCl_3$) of compound (*S*)-**3**

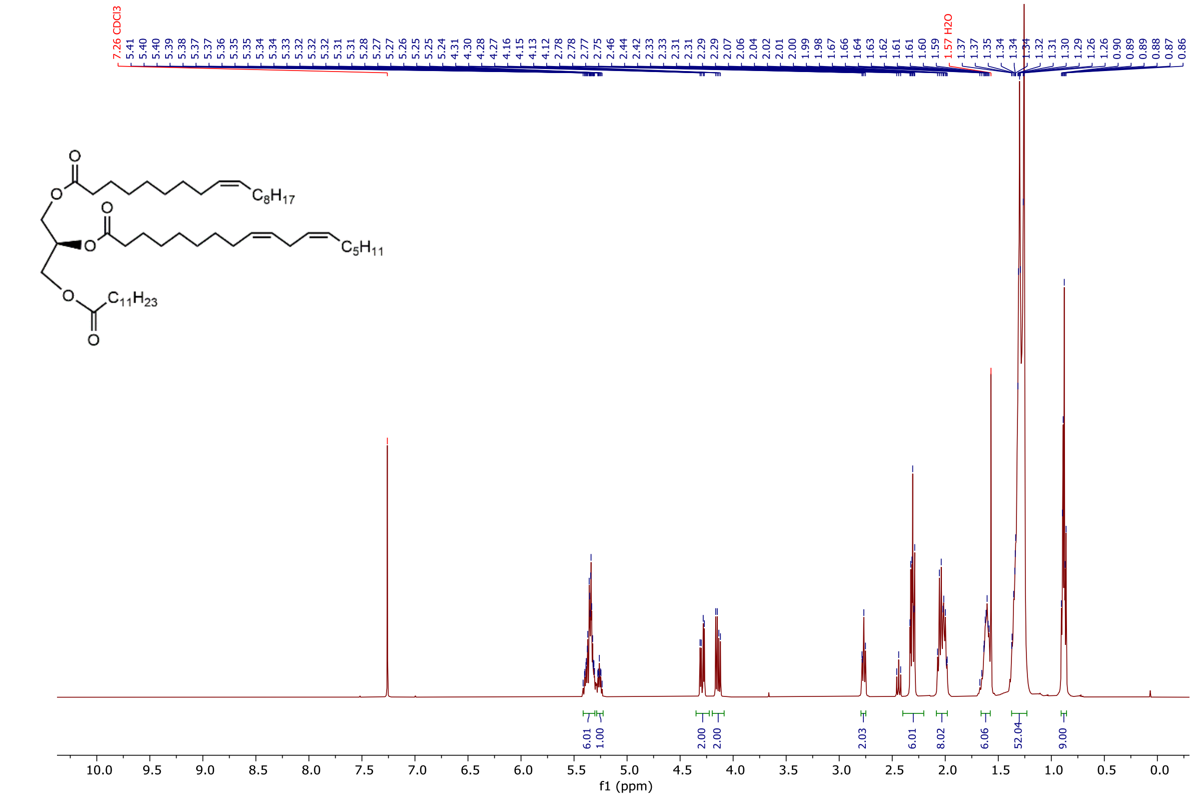

^{13}C{H} NMR (101 MHz, $CDCl_3$) of compound (*S*)-**3**

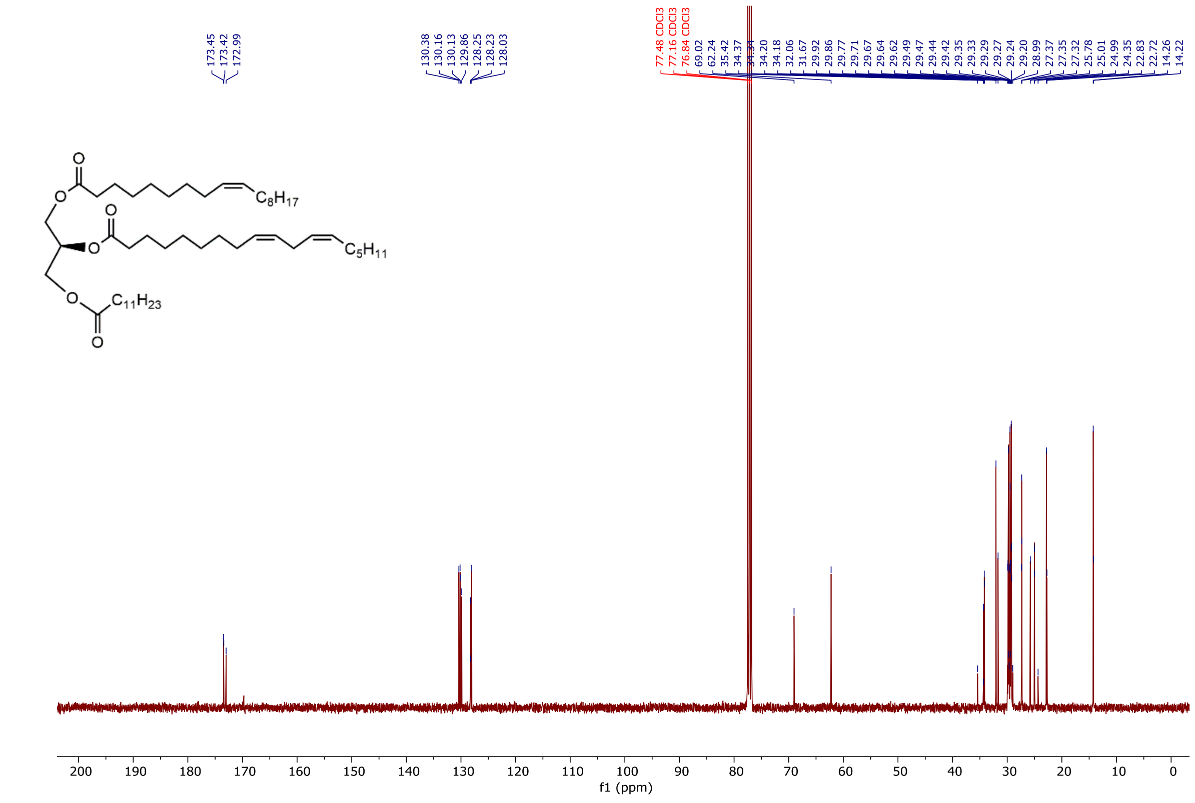

^{1}H-^{1}H COSY spectrum of compound (*S*)-**3**

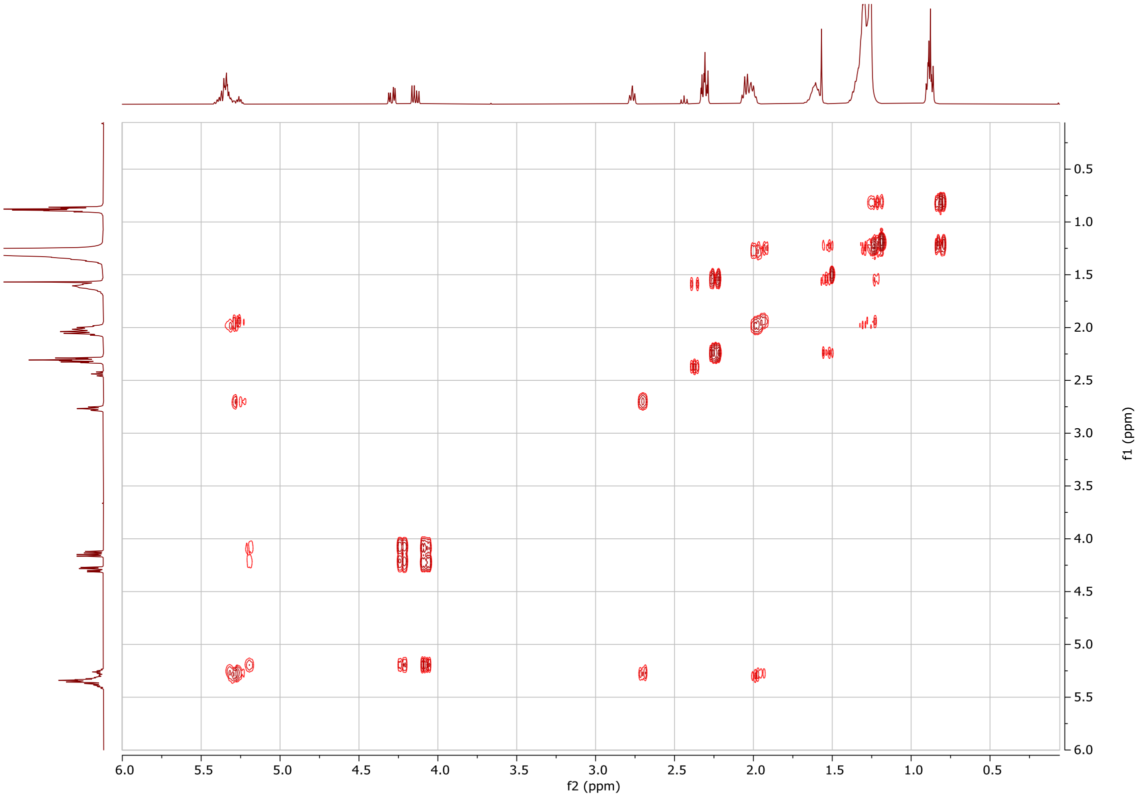

^{13}C-^{1}H HSQC spectrum of compound (*S*)-**3**

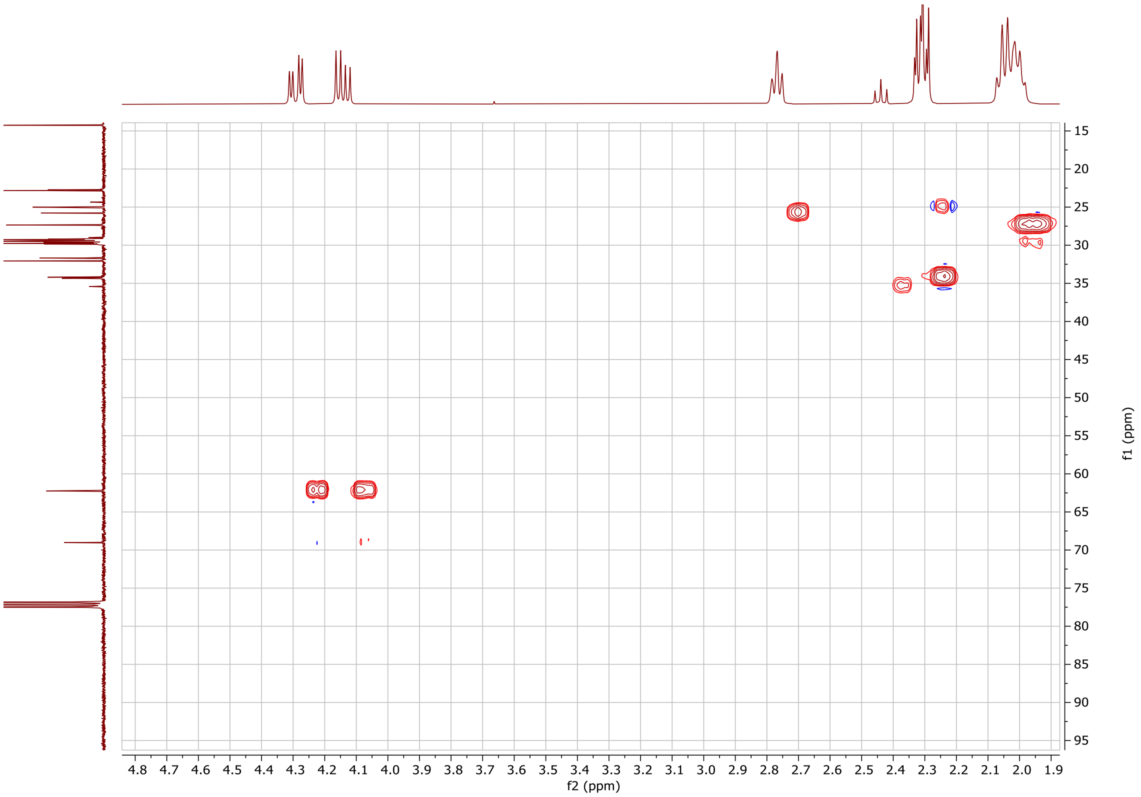

^{1}H NMR (400 MHz, $CDCl_3$) of compound (*S*)-**4**

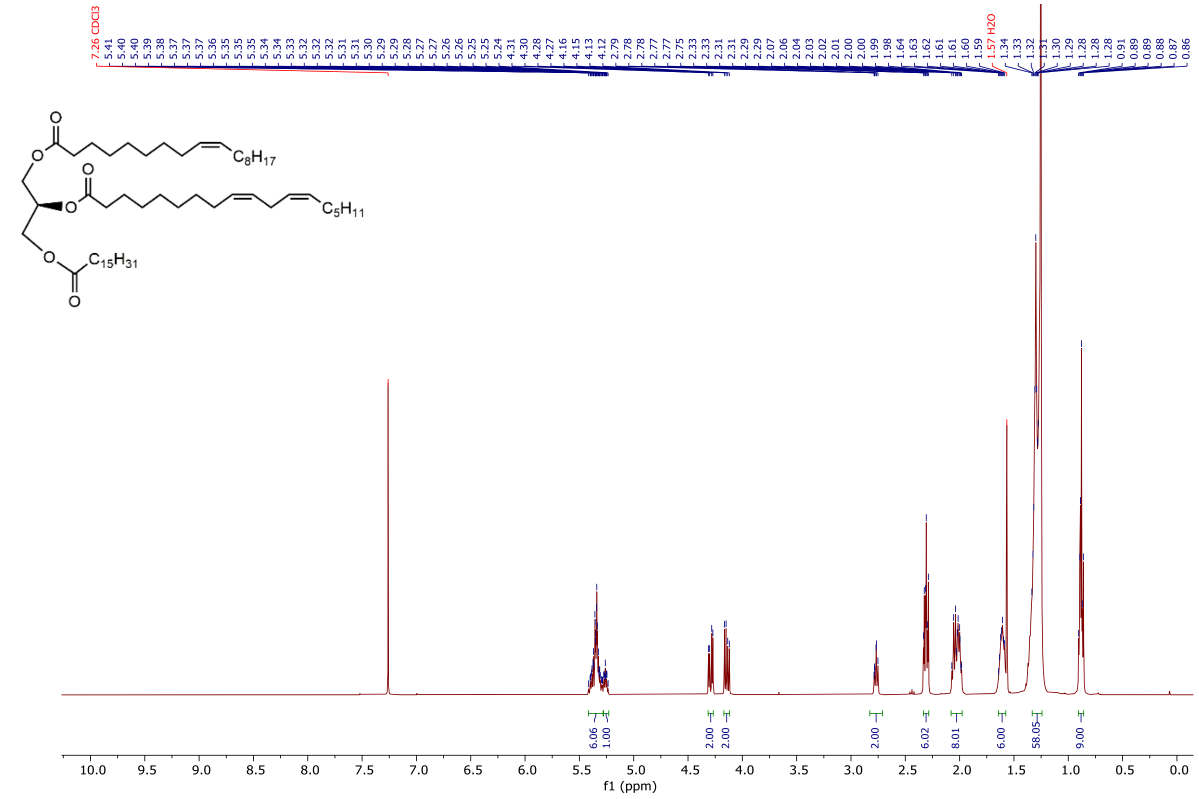

$^{13}C\{H\}$ NMR (101 MHz, $CDCl_3$) of compound (*S*)-**4**

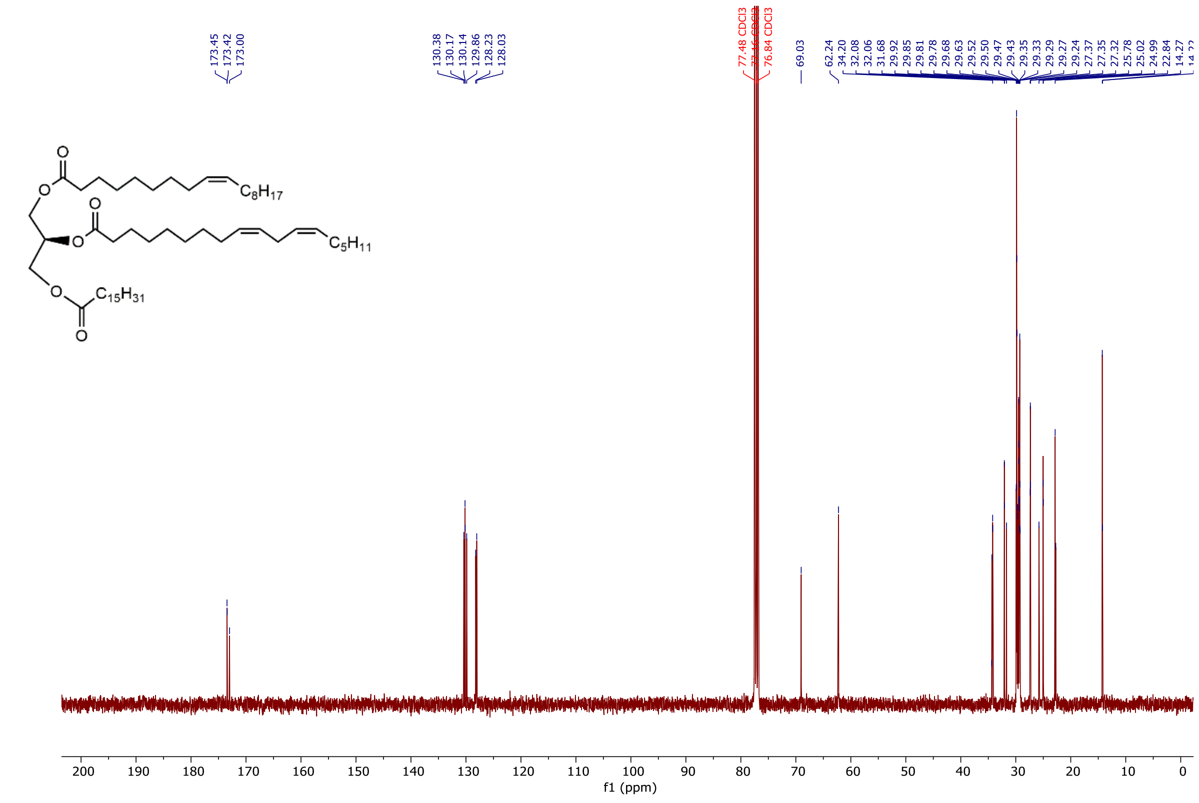

^{1}H-^{1}H COSY spectrum of compound (*S*)-**4**

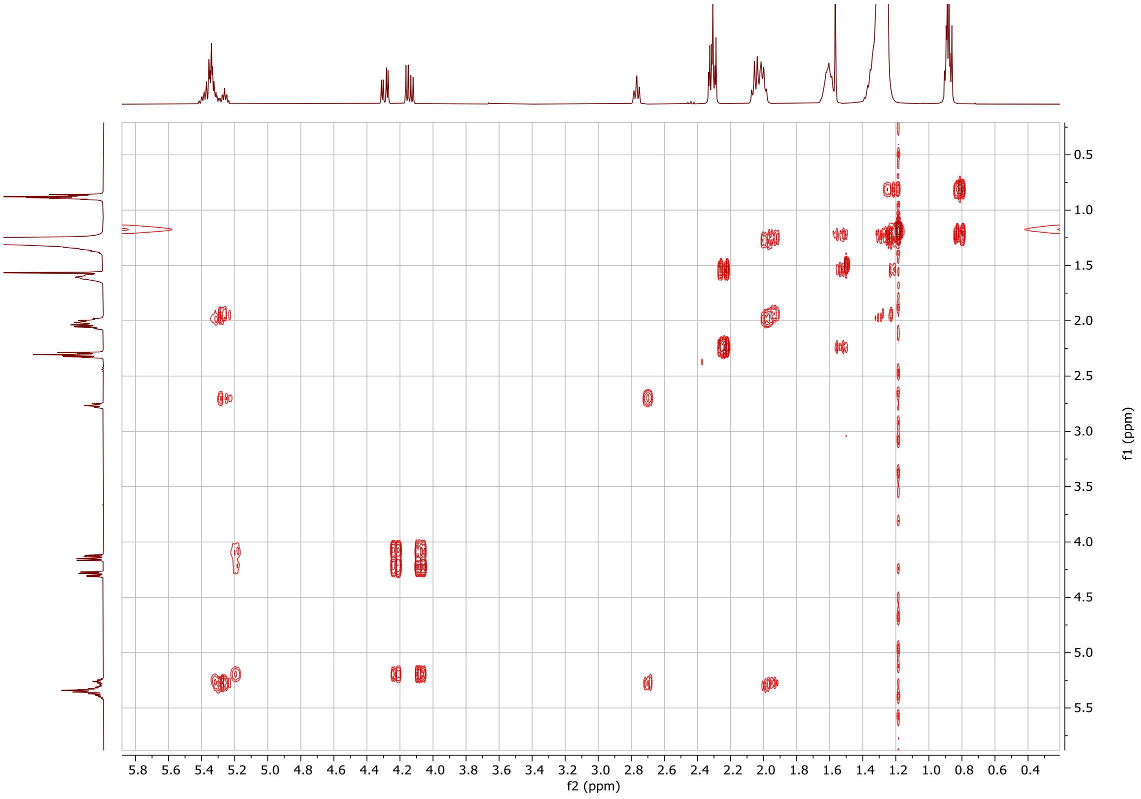

^{13}C-^{1}H HSQC spectrum of compound (*S*)-**4**

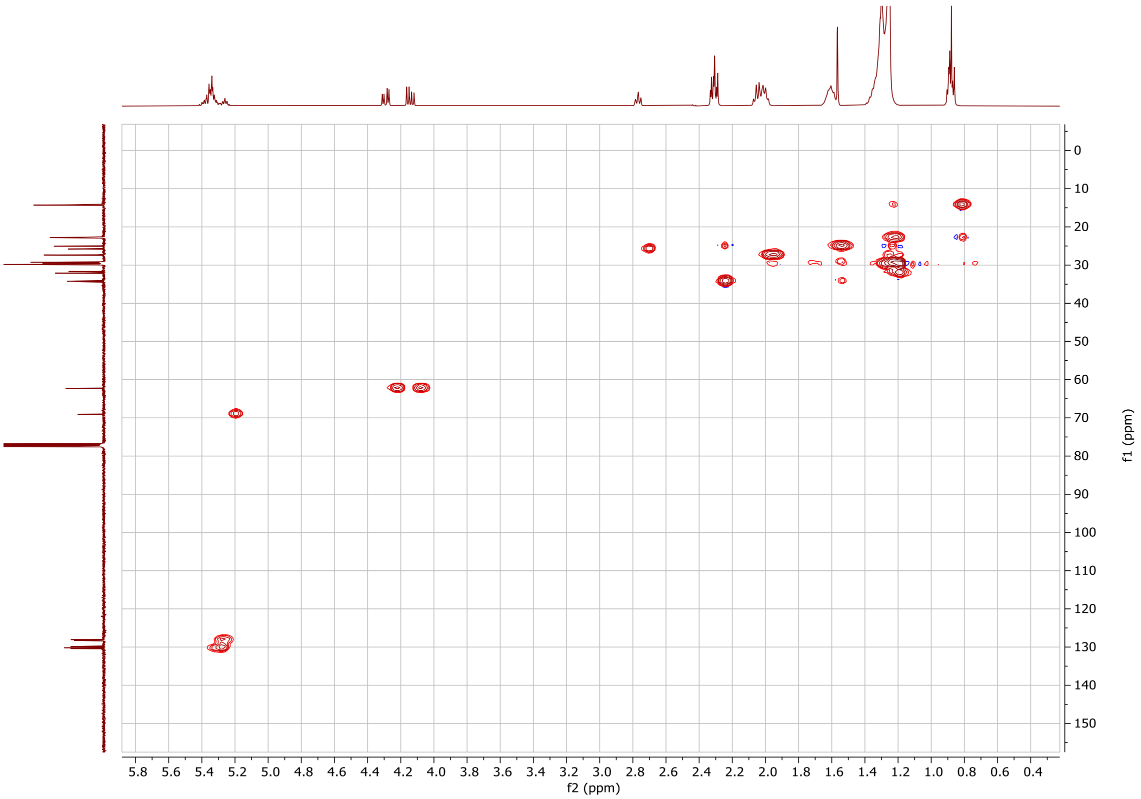

Supplement: Supplementary file 1 [file molecules-29-01633-s001.zip › molecules-2940333-supplementary.pdf]
